# Dynamic Au–C σ-Bonds Leading to an Efficient Synthesis of [*n*]Cycloparaphenylenes (*n* = 9–15) by Self-Assembly

**DOI:** 10.1021/jacsau.2c00194

**Published:** 2022-07-11

**Authors:** Yusuke Yoshigoe, Yohei Tanji, Yusei Hata, Kohtaro Osakada, Shinichi Saito, Eiichi Kayahara, Shigeru Yamago, Yoshitaka Tsuchido, Hidetoshi Kawai

**Affiliations:** † Department of Chemistry, Faculty of Science, Tokyo University of Science, 1-3 Kagurazaka,Shinjuku-ku, Tokyo 162-8601, Japan; ‡ Laboratory for Chemistry and Life Science, Institute of Innovative Research, Tokyo Institute of Technology, 4259, Nagatsuta, Midori-ku, Yokohama 226-8503, Japan; § Institute for Chemical Research, Kyoto University, Uji, Kyoto 611-0011, Japan

**Keywords:** dynamic covalent bond, cycloparaphenylenes, Au(I) complex, Au(I)−C σ-bond, kinetics, self-assembly

## Abstract

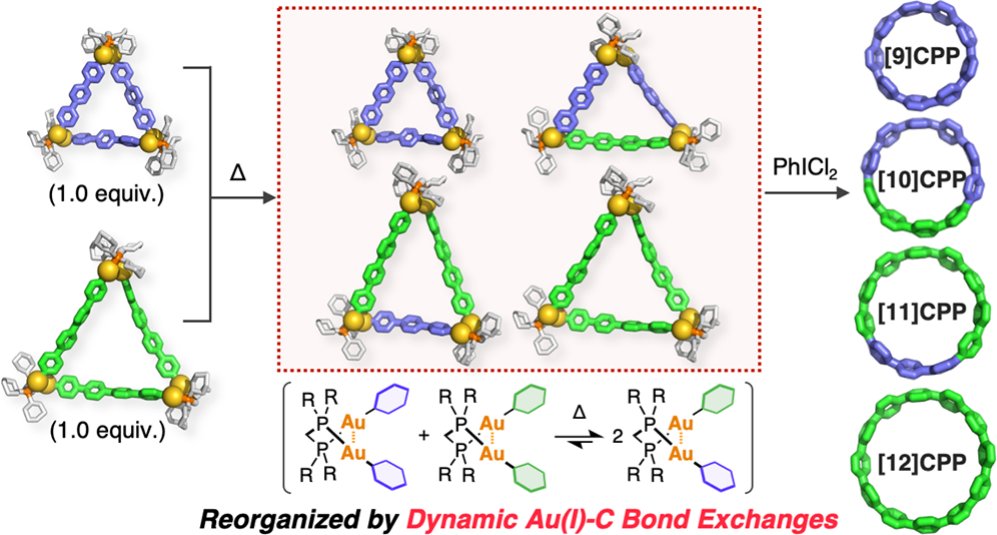

The transmetalation of the digold(I) complex [Au_2_Cl_2_(dcpm)] (**1**) (dcpm = bis(dicyclohexylphosphino)methane) with oligophenylene diboronic acids gave the triangular macrocyclic complexes [Au_2_(C_6_H_4_)_*x*_(dcpm)]_3_ (*x* = 3, 4, 5) with yields of over 70%. On the other hand, when the other digold(I) complex [Au_2_Cl_2_(dppm)] (**1′**) (dppm = bis(diphenylphosphino)methane) was used, only a negligible amount of the triangular complex was obtained. The control experiments revealed that the dcpm ligand accelerated an intermolecular Au(I)–C σ-bond-exchange reaction and that this high reversibility is the origin of the selective formation of the triangular complexes. Structural analyses and theoretical calculations indicate that the dcpm ligand increases the electrophilicity of the Au atom in the complex, thus facilitating the exchange reaction, although the cyclohexyl group is an electron-donating group. Furthermore, the oxidative chlorination of the macrocyclic gold complexes afforded a series of [*n*]cycloparaphenylenes (*n* = 9, 12, 15) in 78–88% isolated yields. The reorganization of two different macrocyclic Au complexes gave a mixture of macrocyclic complexes incorporating different oligophenylene linkers, from which a mixture of [*n*]cycloparaphenylenes with various numbers of phenylene units was obtained in good yields.

## Introduction

Self-assembly of organic ligands and metals, as exemplified in the metal–organic framework, has been recognized as a promising method to synthesize structurally controlled caged molecules and porous materials with controlled size and functions.^1−4^ Furthermore, the use of a covalent bond instead of the noncovalent bond has also been actively developed as, for example, the covalent organic framework (COF).^5,6^ However, the latter examples are still limited compared to those using noncovalent bonds.^7^ This is mainly because of the lower reversibility of covalent bonds than that of noncovalent bonds, necessitating a long reaction time and harsh conditions to reach an equilibrium structure. Therefore, the development of a new dynamic covalent bond would significantly impact the synthesis of COFs and related molecules by the self-assembly process.

**Figure 1:**
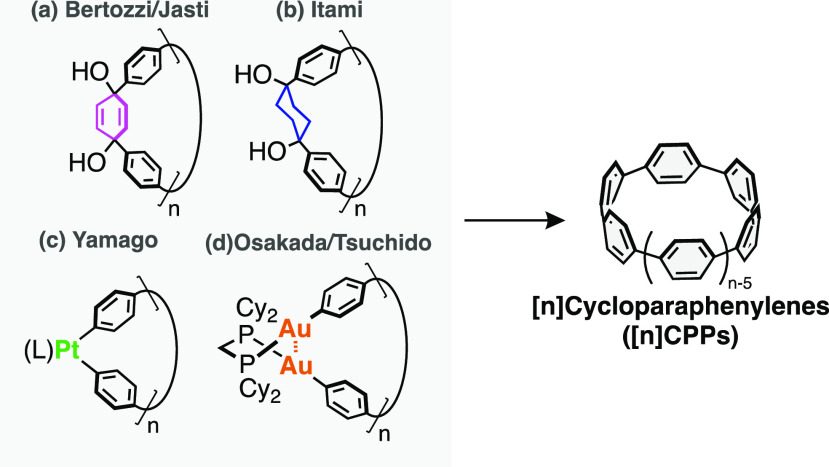
Synthetic routes to [*n*]cycloparaphenylenes reported by (a) Bertozzi/Jasti, (b) Itami, (c) Yamago, and (d) Osakada/Tsuchido.

Cyclo[*n*]paraphenylenes ([*n*]CPPs), where *n* is the number of phenylene groups, are organic macrocycles that consist of 1,4-linked phenylene units.^7−11^ While CPPs were imaginary molecules until 2009, CPPs with various sizes (*n* = 5–16, 18, 20, 21) are now being synthesized with the development of the innovative synthetic methods by Bertozzi/Jasti,^12^ Itami,^13^ Yamago,^14,15^ and Osakada/Tsuchido^16^ (Figure 1). Furthermore, these works unveiled unique size-dependent physical properties^17^ and applications of CPPs, that is, circularly polarized luminescent materials,^18−21^ biological fluorophores,^22,23^ gas-adsorption materials,^24−26^ and electron-transport materials.^27^ In addition, CPPs have also been used to construct unique molecular architectures,^28−30^ such as supramolecular host–guest molecules,^31−37^ mechanically interlocked molecules,^38−40^ and building blocks for tubular nanostructures.^41−44^ These results indicate the necessity for a size-selective and high-yielding synthetic method for CPPs.^45^

The synthesis of CPPs can be classified into two methods depending on the precursor to induce curvature; one employs masked benzene units (Figure 1a,b), and the other uses transition-metal complexes (Figure 1c,d). The latter method resembles COF synthesis as it relies on the assembly of a linear paraphenylene unit by a transition-metal complex, that is, Pt or Au, forming covalent metal (M)–carbon (C) bonds. The presence of equilibrium in the formation of the Pt-complexes has already been reported in the random synthesis of [*n*]CPPs.^46^ However, the control of the rate of equilibrium and the size of the Pt-macrocycles has never been achieved so far.

**Scheme 1:**
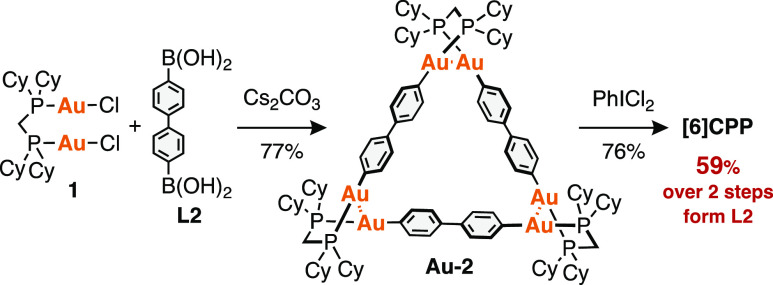
Synthesis of [6]CPP by an Au-Mediated Method (Our Previous Study)^16^

In 2020, some of the authors of this paper reported the synthesis of [6]CPP from a macrocyclic Au complex (Scheme 1).^16^ The reaction of 4,4′-diphenyldiboronic acid (**L2**) with [Au_2_Cl_2_(dcpm)] (**1**) (dcpm = bis(dicyclohexylphosphino)methane) produced the triangular hexagold(I) complex [Au_2_(C_6_H_4_)_2_(dcpm)]_3_ (**Au-2**) isolated in 77% yield. The oxidative chlorination^47−51^ of **Au-2** by PhICl_2_ afforded [6]CPP in a total yield of 59% (over two steps from **1**). Thus, this synthetic method has the advantage of highly efficient synthesis of CPPs and related nanohoops from three arylene units.^52−56^ However, the scope of this synthetic method and the mechanism of the efficient formation of the triangular Au complexes remain to be examined.

Herein, we report the selective and random synthesis of [*n*]CPPs (*n* = 9–15) in high overall yield through macrocyclic Au complexes with oligophenylene linkers. We further clarified the crucial role of the electron-donating tetra-cyclohexyl groups in ligand **1**, which significantly promotes the dynamic exchange reaction of Au–C bonds, giving desired cyclic structures under mild conditions. In addition, a detailed mechanism of the exchange reaction is proposed based on kinetic studies and structural analyses of the Au complexes. We believe that the current finding is not only useful for the synthesis of CPPs and related cyclic π-conjugated molecules but also for the development of new COFs.

## Results and Discussion

### Synthesis and Characterization of Macrocyclic Au Complexes and [*n*]CPPs (*n* = 9, 12, 15)

**Scheme 2:**
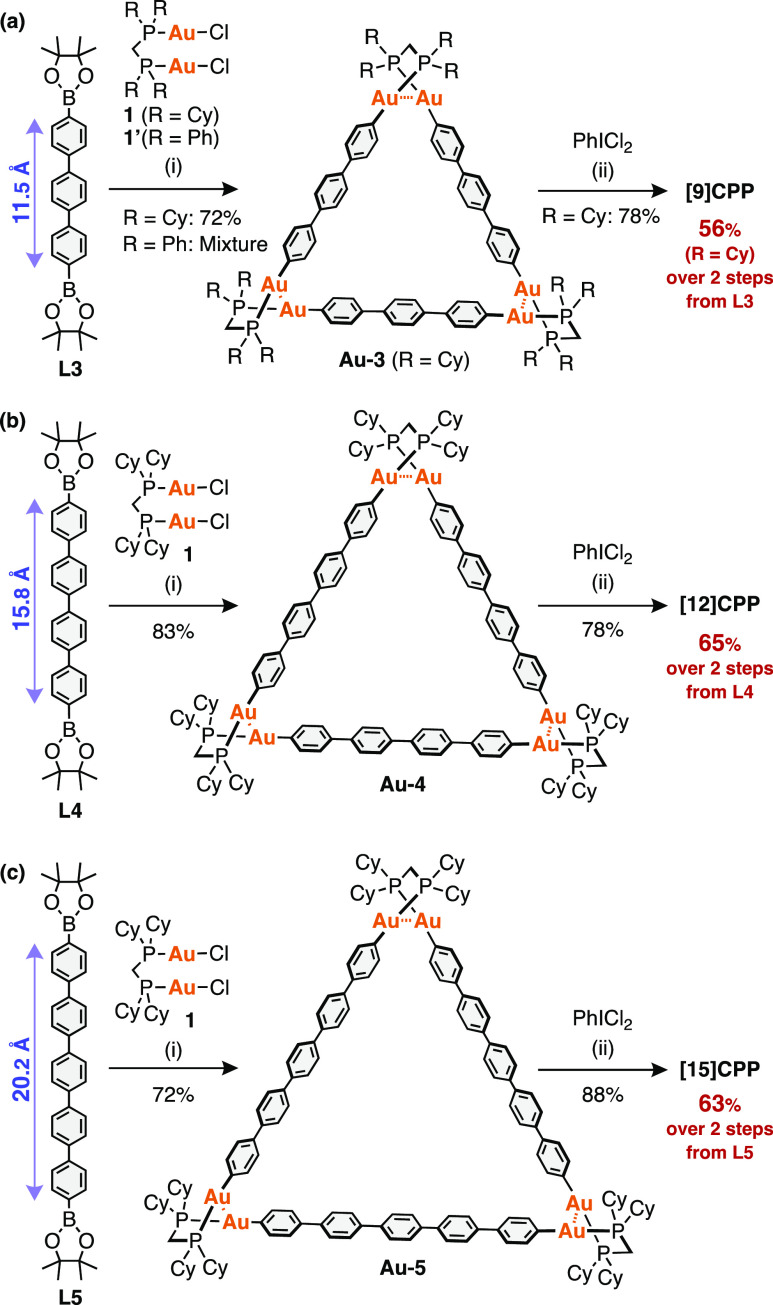
Synthesis of [*n*]CPPs by the Au-Mediated Method; (a) [9]CPP, (b) [12]CPP, and (c) [15]CPP Reagents and conditions: (i) [AuCl_2_(R_2_PCH_2_PR_2_)] (**1**: R = Cy (dcpm), **1′**: R = Ph (dppm)) (1.0 equiv), Cs_2_CO_3_ (6.0 equiv), toluene/ethanol/water (4:1:1), 50 °C, overnight; (ii) PhICl_2_ (3.0 equiv), DMF, −60 °C, 0.5 h, and then r.t., overnight. Linker lengths were determined from the molecular structures of oligophenylene simulated using MMFF calculations.

The transmetalation of [Au_2_Cl_2_(dcpm)] (**1**) with an equimolar amount of 4,4″-terphenyldiboronic acid pinacol ester (**L3**) was conducted in the presence of Cs_2_CO_3_ in toluene/ethanol/water at 50 °C. After stirring overnight, the resulting white solid was collected by filtration and characterized as the triangular macrocyclic complex [Au_2_(C_6_H_4_)_3_(dcpm)]_3_ (**Au-3**) in a 72% yield (Scheme 2a, reaction i). In contrast, the use of [Au_2_Cl_2_(dppm)] (**1′**) (dppm = bis(diphenylphosphino)methane) instead of **1** under the otherwise identical conditions gave a purple solid, which was partially soluble in (Cl_2_CD)_2_ and had broad signals in the ^1^H NMR spectrum (see Figure S9), suggesting the formation of linear and/or undesired macrocyclic oligomers. The results indicate the importance of the dcpm ligand of **1** over dppm to increase the dynamicity of the Au–C bond-exchange reaction, as discussed below.

By using **1** as a Au complex, oligoparaphenyl diboronic acid pinacol ester with a quaterphenylene group (**L4**) or a quinquephenylene group (**L5**) under otherwise identical reaction conditions afforded the corresponding Au complexes, [Au_2_(C_6_H_4_)_*x*_(dcpm)]_3_ (*x* = 4 from **Au-4**, 5 from **Au-5**) in 83 and 72% yields, respectively (Scheme 2b,c, reaction i). It is worth noting that the triangular complexes were obtained as a sole product in all cases.

**Figure 2:**
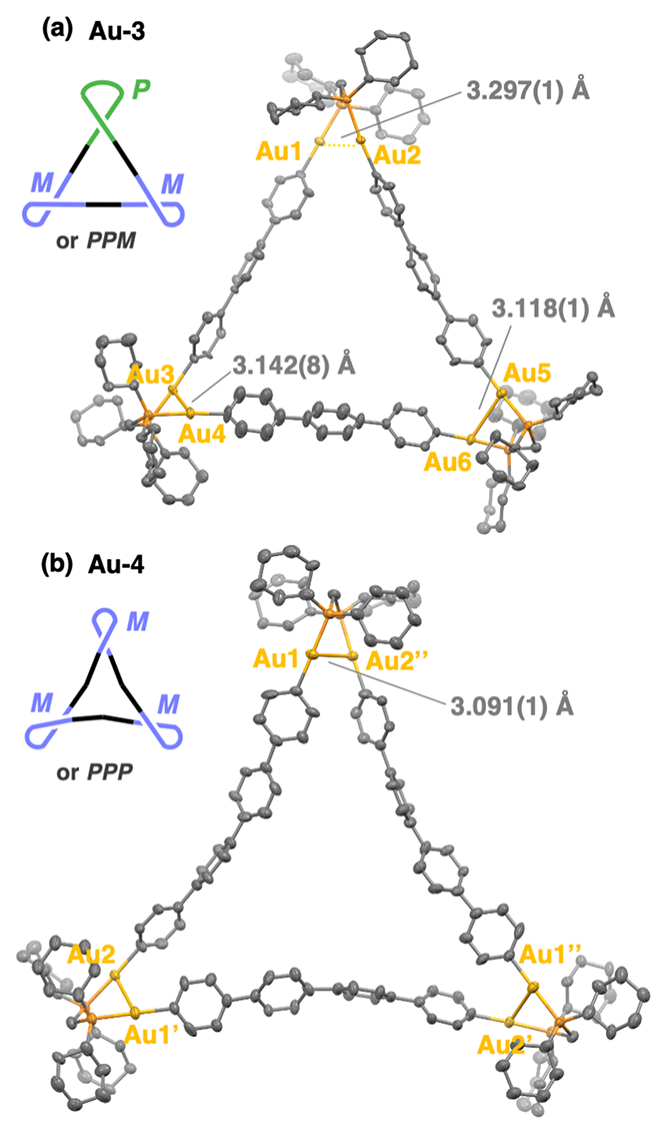
Molecular structures of (a) **Au-3** (*C*_2_-symmetry) and (b) **Au-4** (*D*_3_-symmetry) with thermal ellipsoids at 30% probability. Hydrogen atoms and solvent molecules are omitted for clarity.

Single crystals of **Au-3** (Figure 2a) and **Au-4** (Figures 2b and S95) suitable for X-ray crystallography were obtained via the vapor diffusion of CH_3_CN into (Cl_2_CH)_2_ solutions of each complex. Both molecules adopt a triangular molecular structure similar to that of **Au-2**,^16^ consisting of three oligophenylene linkers and three Au_2_(dcpm) units. The complex with the terphenylene linker, **Au-3**, adopts a pseudo-*C*_2_-symmetrical structure with *PMM*- or *PPM*-helical Au_2_P_2_C units at the three corners. **Au-4** gave polymorphic crystals with a *D*_3_-symmetrical structure and *PPP* or *MMM* helicity (Figure 2b) along with the *C*_2_-symmetrical structure (Figure S95).^57^ These triangular molecular structures were stabilized by aurophilic interactions^58−61^ between the two Au(I) centers in each corner. In the *C*_2_-symmetrical structure of **Au-3** (Figure 2a), the distance between the two neighboring gold atoms in two helical corners (3.142(8), 3.118(1) Å) is shorter than that of the other corner (3.297(1) Å). The same phenomenon was observed in the X-ray structures of **Au-2**(16) and **Au-4** (see Figure S95) with *C*_2_-symmetry. On the other hand, the X-ray structure of **Au-4** with *D*_3_-symmetry (Figure 2b) exhibits Au–Au distances of 3.091(1) Å, which are shorter than those of the *C*_2_ isomer. These results indicate stronger aurophilic interactions in the *D*_3_ isomer compared to those in the *C*_2_ isomer. Additionally, the phenylene linkers adopt a bent conformation in the *D*_3_ symmetry, which would be difficult to be adopted in **Au-3** and **Au-4** form with short oligophenylene linkers.

The oxidative chlorination^45^ of **Au-3** occurred upon the addition of 3 equiv of PhICl_2_ in DMF at −60 °C. The C–C bond formation between two phenylene linkers via reductive elimination gave [9]CPP when the reaction temperature was raised to 25 °C (Scheme 2a, reaction ii). The ^1^H NMR spectrum of the crude product showed only one singlet aromatic signal at 7.52 ppm (CDCl_3_, 25 °C), which was assigned to [9]CPP based on the literature.^17^ Purification of the reaction mixture using column chromatography on silica gel afforded the desired product in good yield (78%), along with the Au complex **1**, which was also obtained in 78% yield. [*n*]CPPs (*n* = 12 for **Au-4**, 15 for **Au-5**) were obtained from the corresponding Au complexes in 78% and 88% yields, respectively (Scheme 2b,c, reactions ii). [*n*]CPPs (*n* = 9, 12, 15) have successfully been obtained from oligophenylene diboronates, **L3**–**5**, in higher overall yields than other methods (see Tables S1–S3).^62^

### Kinetic Studies of the Au–C σ-Bond-Exchange Reaction

To clarify the origin of the unique ligand effect (**1** vs **1′**) for the formation of Au complexes, we next examined the dynamics of the Au–C bond-exchange reactions using model complexes. There were several reports on the exchange reaction of M–C σ-bonds (M = Pt(II), Pd(II), Au(I), etc.) (see supporting Scheme S3b),^63−69^ and the reactions were generally regarded as a slow process compared to those of the noncovalent metal–ligand bonds, that is, Pd–N^1^ and Pt–N^2^ bonds (see Scheme S3a). However, the exchange reactions involving aryl–Au(I) complexes have never been reported to date.^70−74^

**Figure 3:**
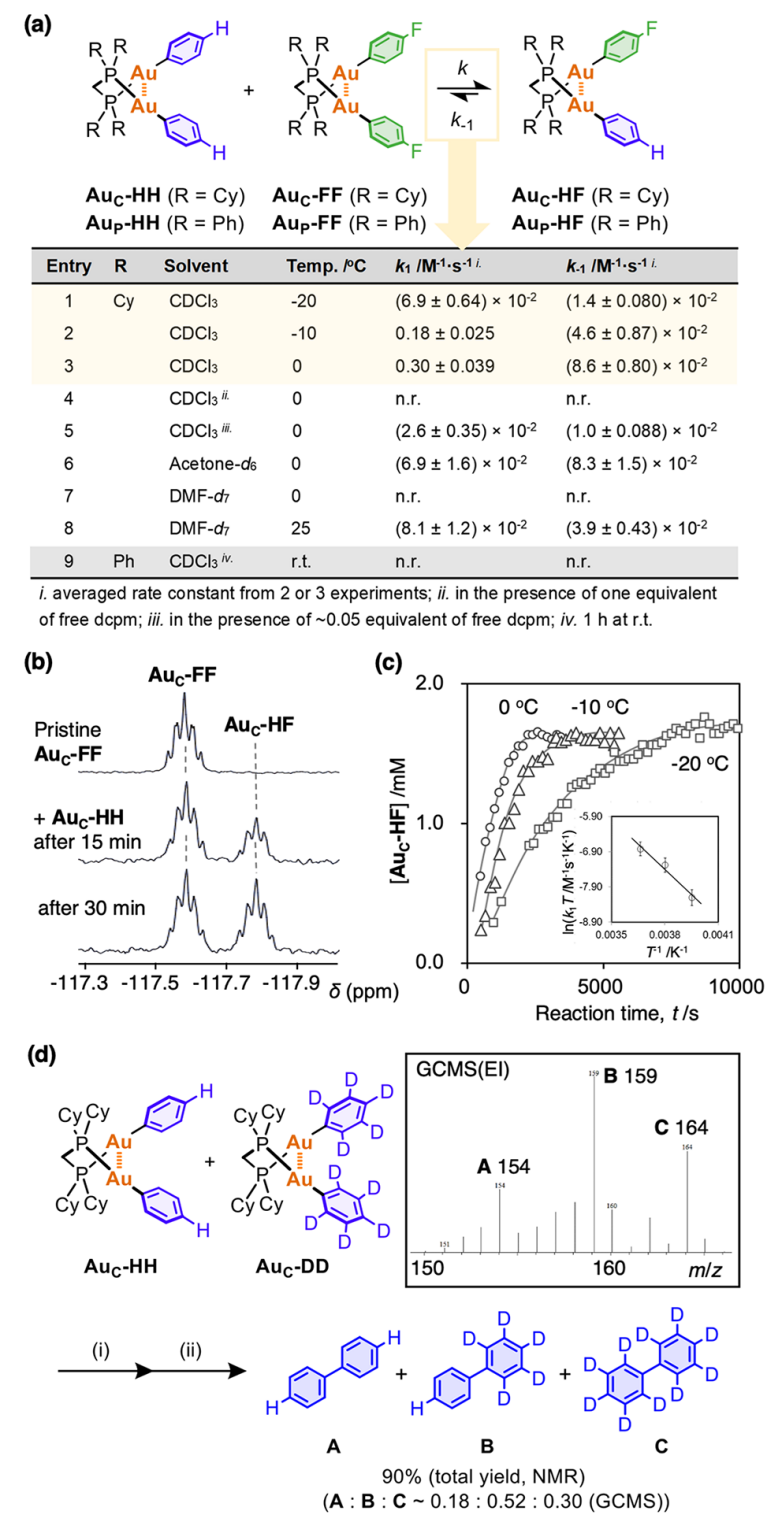
(a) Kinetic rates of comproportionation of (aryl)_2_Au_2_ complexes (**Au**_**C**_**-HH** and **Au**_**C**_**-FF**; **Au**_**P**_**-HH** and **Au**_**P**_**-FF**). (b) ^19^F NMR spectra (376 MHz, CDCl_3_, 23 °C): pristine **Au**_**C**_**-FF** (top), a mixture of **Au**_**C**_**-HH** and **Au**_**C**_**-FF** 15 min after its preparation (center), and the mixture after 30 min (bottom). (c) Reaction profile of the comproportionation at 0 (circles), −10 (triangles), and −20 (squares) °C (inset: Eyring plot); (d) comproportionation of **Au**_**C**_**-HH** and **Au**_**C**_**-DD**, followed by reductive elimination to yield biphenyls **A**, **B**, and **C** (inset: GC–MS spectra). Reagents and conditions: (i) CH_2_Cl_2_, r.t., 2 h; (ii) PhICl_2_ (2.0 equiv), CH_2_Cl_2_, −60 °C, 0.5 h, then r.t., overnight.

At first, two acyclic dinuclear Au(I) complexes having the dcpm ligand, [Au_2_Ph_2_(dcpm)] (**Au**_**C**_**-HH**), and [Au_2_(C_6_H_4_-4-F)_2_(dcpm)] (**Au**_**C**_**-FF**), were synthesized, and the formation of the unsymmetrical Au(I) complex, [Au_2_Ph(C_6_H_4_-4-F) (dcpm)] (**Au**_**C**_–**HF**), was observed upon mixing equimolar amounts (1.7 mM each) of **Au**_**C**_**-HH** and **Au**_**C**_**-FF** in CDCl_3_ (Figure 3a). The ^19^F NMR spectroscopic analysis indicated that the reaction reached equilibrium after 30 min at 25 °C (Figure 3b).^75^ To our surprise, rapid bond exchange of the Au–C σ-bonds was clearly observed even at or below room temperature. The rate constants of the comproportionation (*k*_1_) and disproportionation (*k*_–1_) employing a mixture of **Au**_**C**_**-HH** and **Au**_**C**_**-FF** at −20 °C were determined to be *k*_1_ = (6.9 ± 0.64) × 10^–2^ M^–1^·s^–1^ and *k*_–1_ = (1.4 ± 0.08) × 10^–2^ M^–1^·s^–1^ (Figure 3c). The values are in good agreement with the reversible second-order reaction model.^76^ The temperature dependence of the rate of comproportionation in CDCl_3_ was obtained to be given in *k*_1_ = 0.30 ± 0.039 M^–1^·s^–1^, *k*_–1_ = (8.6 ± 0.80) × 10^–2^ M^–1^·s^–1^ (at 0 °C), *k*_1_ = 0.18 ± 0.025 M^–1^·s^–1^, (4.6 ± 0.87) × 10^–2^ M^–1^·s^–1^ (at −10 °C) (Figure 3a; entry 2, 3). By adding 1 equiv of the ancillary ligand, dcpm, to a mixture of **Au**_**C**_**-HH** and **Au**_**C**_**-FF** in CDCl_3_ at 0 °C, two doublet peaks of ^31^P{^1^H} NMR at δ 45.8 and −10.4 (^2^*J*_P–P_ = 80 Hz), corresponding to mononuclear Au complexes,^77,78^ [AuAr(dcpm)] (Ar = C_6_H_5_, or C_6_H_4_-4-F), were obtained, and the reaction was in a resting state (Figure 3a, entry 4). Even in the presence of a catalytic amount (∼0.05 equiv) of free dcpm, the distinct reduction in the rate of comproportionation of the Au complexes in CDCl_3_ at 0 °C was observed with the constants of *k*_1_ = (2.6 ± 0.35) × 10^–2^ M^–1^·s^–1^ and *k*_–1_ = (1.0 ± 0.088) × 10^–2^ M^–1^·s^–1^ (Figure 3a, Entry 5). Coordinative solvents, such as acetone and DMF, tend to decelerate the aryl exchange (Figure 3a, entry 6–8). The kinetic rates of the reaction using acetone-*d*_6_ at 0 °C were *k*_1_ = (6.9 ± 1.6) × 10^–2^ M^–1^·s^–1^ and *k*_–1_ = (8.3 ± 1.5) × 10^–2^ M^–1^·s^–1^, which were one-tenth as in CDCl_3_ at the same temperature. In a DMF solution at 0 °C, the desired aryl exchange reaction did not proceed; thus, the reaction mixture was warmed up to 25 °C to proceed with the reaction with rate constants of *k*_1_ = (8.1 ± 1.2) × 10^–2^ M^–1^·s^–1^ and *k*_–1_ = (3.9 ± 0.43) × 10^–2^ M^–1^·s^–1^. We believe that these solvents would coordinate with the Au complexes to reconstruct the less reactive compounds, such as [AuArL] (L = coordinative solvent) which is similar to [AuAr(dcpm)]. The putative mechanism is discussed in a later section.

To verify the effect of the electronic nature of the aryl groups, we conducted NMR studies of the ligand-exchange reaction using an equimolar mixture of [Au_2_Ph_2_(dcpm)] (**Au**_**C**_**-HH**) and [Au_2_(C_6_D_5_)_2_(dcpm)] (**Au**_**C**_**-DD**) in CDCl_3_ at −20 °C. However, the isomeric shifts of the deuterated phenyl groups were too small to distinguish deuterated groups from nondeuterated groups or follow the reaction rates determined by NMR. The chemical oxidation of the reaction mixture after 2 h caused the formation of a mixture of C_6_H_5_–C_6_H_5_, C_6_H_5_–C_6_D_5_, and C_6_D_5_–C_6_D_5_ in a 0.18:0.52:0.30 molar ratio, which was determined by GC–MS analysis (Figure 3d). The results indicated that scrambling of the phenyl group was completed within a short time, similar to the corresponding reactions of **Au**_**C**_**-HH** and **Au**_**C**_**-FF**.

Moreover, the comproportionation of Au complexes having the dppm ligand, **Au**_**P**_**-HH**, and **Au**_**P**_**-FF** did not occur at all even after 1 h at room temperature (Figure 3a, entry 9). The results clearly suggest that the cyclohexyl groups on the phosphines of the dcpm ligands are essential for the progress of the reaction.

**Figure 4:**
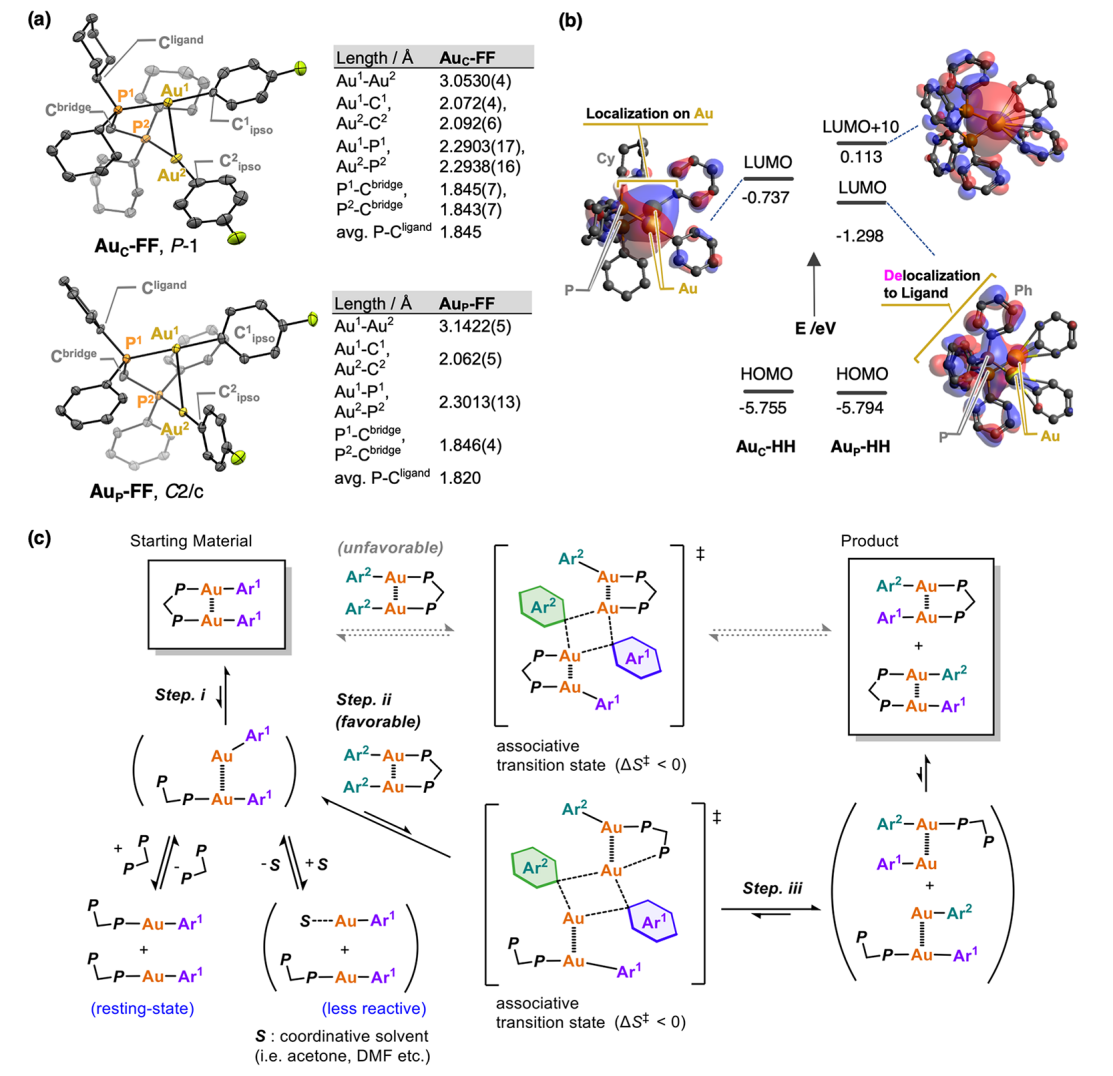
(a) ORTEP drawings of **Au**_**C**_**-FF** and **Au**_**P**_**-FF** with thermal ellipsoids at 50% probability; hydrogen atoms are omitted for clarity. Selected bond distances (Å) are summarized in the inserted table. (b) Energy levels of the frontier orbitals of **Au**_**C**_**-HH** and **Au**_**P**_**-HH** and a depiction of their LUMOs (M06/6-31G for C, H, P, LANL2TZ(f) for Au, iso value = 0.025); hydrogen atoms are omitted for clarity; (c) putative mechanism for the Au–C bond-exchange reaction between (aryl)_2_Au_2_ complexes.

The different dynamic behavior of the Au(I)–aryl complexes with dcpm and dppm can be explained by the structural change of the Au(I) complex due to the electronic of the substituent as estimated from their crystal structures of **Au**_**C**_**-FF** and **Au**_**P**_**-FF** (Figure 4a). The Au–P bonds of **Au**_**C**_**-FF** (2.2903(17) and 2.2938(16) Å) are shorter than those of **Au**_**P**_**-FF** (2.3013(13) Å) due to the electron-donating effect of the cyclohexyl-substituted phosphine compared to that of the phenyl-substituted one. Then, the strong σ-donation effect leads to a higher trans effect of dcpm compared to that of dppm to place aryl groups opposite to each other. Therefore, the lengths of the two Au–C_ipso_ bonds of **Au**_**C**_**-FF** (2.072(4) and 2.092(5) Å) are slightly longer than those of **Au**_**P**_**-FF** (2.062(5) Å). Namely, the above results suggest that the Au(I)–C σ-bonds in **Au**_**C**_**-FF** are weaker than those in **Au**_**P**_**-FF**.

Theoretical calculations further suggested the importance of the cyclohexyl group on the reactivities of the Au–aryl complexes. Thus, the lowest unoccupied molecular orbital (LUMO) of **Au**_**C**_**-HH** is localized at the apical position of the gold atom with *E* = −0.737 eV, while it is delocalized to the ancillary phosphine ligand in the case of **Au**_**P**_**-HH** (Figure 4b). In contrast, the lowest unoccupied, localized orbital on the gold of **Au**_**P**_**-HH** was observed at the LUMO + 10 level with *E* = 0.113 eV. The results indicate that the dcpm ligand enhances the electrophilic reactivity of Au atoms in **Au**_**C**_**-HH** by localizing the electrophilic orbital of the Au atoms and lowering its energy level as compared to the dppm ligand. Since the cyclohexyl group is an electron-donating group, the calculation results that the electrophilicity of the Au atom increases with the dcpm ligand cannot be explained by a simple electronic effect only. Therefore, this seemingly contradictory result is most likely due to the structural difference between **Au**_**C**_ and **Au**_**P**_ complexes caused by the trans effect, as discussed in the previous section. These results also suggest the mechanism of the Au–aryl exchange reaction, which involves the nucleophilic attack from a reactive Au–C_aryl_ bond to the electrophilic LUMO orbital on the gold atom.

The activation parameters for the comproportionation between **Au**_**C**_**-HH** and **Au**_**C**_**-FF** were determined to be Δ*G*^⧧^ = 17 kcal mol^–1^, Δ*H*^⧧^ = 9.5 kcal mol^–1^, and Δ*S*^⧧^ = −26 cal mol^–1^ K^–1^ (at 25 °C) based on the Eyring plot (Figure 3c, inset). The negative value of Δ*S*^⧧^ implies that the bond-exchange process proceeds *via* an associative mechanism.^63,64,77,79−81^ Considering the deceleration of the aryl exchange reaction upon the addition of catalytic amounts of dcpm in the former section, it is plausible that a ligand dissociation precedes an associative transition state. To combine the above results, we could propose the putative mechanism that the bond exchange or metathesis between the metal centers and their organic ligands proceeded *via* a mechanism with three steps: (i) a ligand dissociation to form reactive Au species [i.e., AuAr or ArAu–AuAr(dcpm)]; (ii) formation of an associative transition state;^65,66,68−70,73,74^ (iii) the Au–C_aryl_ bond exchange (Figure 4c). The complexes with bridging organic ligands, presumed to be the intermediate of organic ligand exchange, have been proposed in kinetic studies in which aryl–Cu(I) and aryl–Au(I) complexes caused trans–cis isomerization of Pd(II) complexes^70,72^ and exchange of their aryl and alkynyl ligands bound to Pd(II) and Rh(I) complexes.^71^

We compared the kinetic and thermodynamic parameters of the comproportionation of **Au**_**C**_**-HH** and **Au**_**C**_**-FF** and those of the reaction of [PtPh_2_(cod)] and [Pt(C_6_H_4_-4-F)_2_(cod)], which we have reported previously (see supporting Scheme S4).^65,82^ The comproportionation of Au_2_ complexes at 0 °C proceeded nearly 10^5^ times as fast as the reaction of [PtPh_2_(cod)] and [Pt(C_6_H_4_-4-F)_2_(cod)] even at 50 °C with rate constants of *k*_1_ = (6.4 ± 0.6) × 10^–6^ and *k*_–1_ = (2.0 ± 0.2) × 10^–6^ M^–1^ s^–1^. The thermodynamic parameters for the above Pt system are Δ*G*^⧧^ = 27 kcal mol^–1^, Δ*H*^⧧^ = 23 kcal mol^–1^, and Δ*S*^⧧^ = −11 cal mol^–1^ K^–1^. The Δ*H*^⧧^ for the Au_2_ system is smaller than that for the Pt system, suggesting that the Au(I)–C bond dissociation *via* the formation of the association complex of intermediate can be expected to occur more easily.

**Figure 5:**
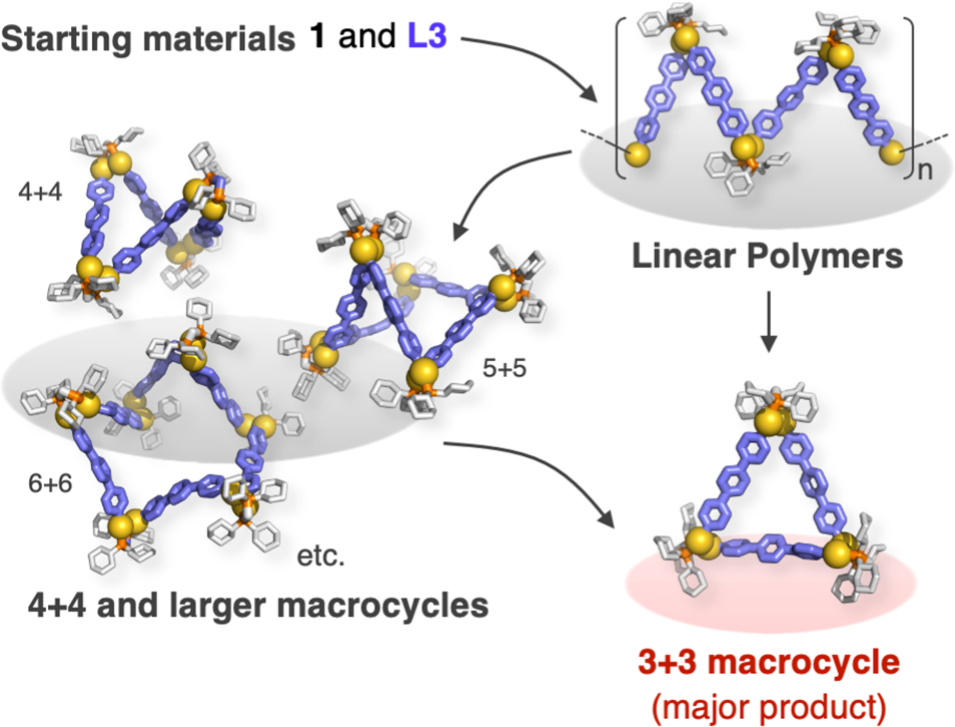
Illustration of a plausible mechanism for the formation of the triangular Au(I) complex **Au-3** via a self-assembly process between **1** and **L3**.

These results imply that the highly efficient macrocyclization in this study should be attributed to the highly dynamic, reversible intermolecular exchanges of Au(I)–C σ-bonds (Figure 5). In the early stage of the reaction between **1** and **L3**, a mixture of acyclic and cyclic oligomers can form under kinetic conditions. Then, the triangular complex becomes the major product via the dynamic bond-exchange reaction between these species. As the macrocyclization reaction proceeds in a heterogeneous mixture, the high thermodynamic stability and/or the high crystallinity of the triangular complex could account for the isolation of the triangular complex as the sole product.

### Synthesis of CPPs from Two Different Oligophenylene Linkers

**Figure 6:**
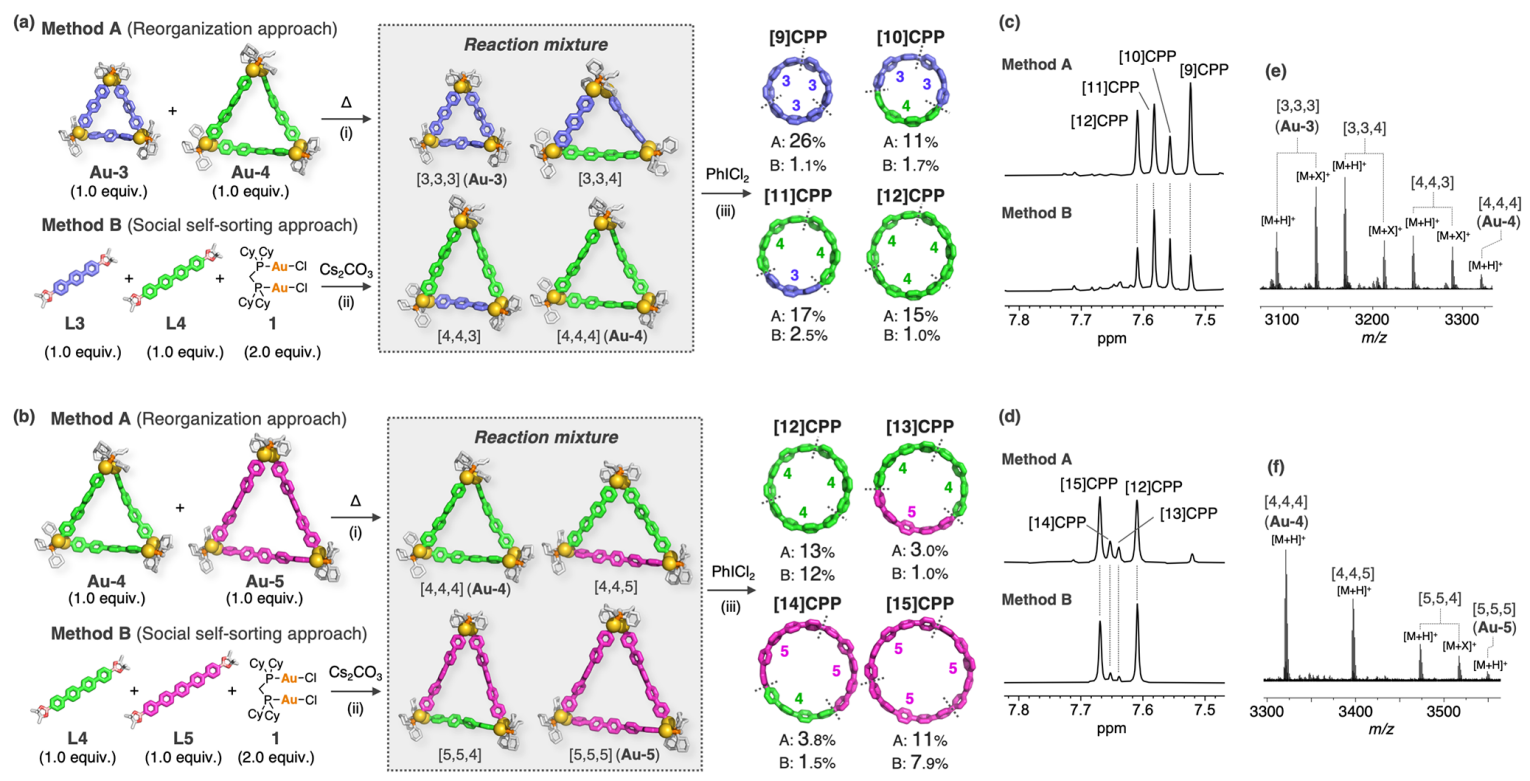
(a,b) Synthesis of [*n*]CPPs from two different macrocyclic Au complexes, **Au-*x*** and **Au-*y*** [(a) *x* = 3, *y* = 4 or (b) *x* = 4, *y* = 5] (method A), or two different oligophenyldiboronic acids, **Lx** and **Ly** [(a) *x* = 3, *y* = 4 or (b) *x* = 4, *y* = 5] with [AuCl_2_(dcpm)] (**1**) (method B). Reagents and conditions: (i) DMF, 50 °C, overnight; (ii) Cs_2_CO_3_ (6.0 equiv), toluene/ethanol/water (4:1:1), 50 °C, overnight; (iii) PhICl_2_ (3.0 equiv), DMF, −60 °C, 0.5 h, and then r.t., overnight. The yields of the CPPs over two steps were determined from the ^1^H NMR signal intensities relative to an internal standard (1,2,4,5-tetrabromobenzene, TBB). (c,d) ^1^H NMR spectra of the reaction mixtures after treatment with PhICl_2_ (400 MHz, CDCl_3_, 25 °C). The signals were assigned with reference to reports in the literature.^17,62^ (e,f) FT-ICR MALDI-TOF MS spectra of the mixture of different macrocyclic Au complexes, **Au-*x*** and **Au-*y*** [(e) *x* = 3, *y* = 4 or (f) *x* = 4, *y* = 5] after mixing at 50 °C in DMF (X = C_2_H_6_O or Me_2_NH).^83^

To explore the scope of the present dynamic aryl-group exchange reactions, two macrocyclic Au complexes (**Au-3**/**Au-4** or **Au-4**/**Au-5**) were mixed in DMF at 50 °C (Figure 6a, Method A). Then, the resulting reaction mixture was directly treated with PhICl_2_ without isolating the Au complexes. The ^1^H NMR spectrum of the products obtained from an equimolar mixture of **Au-3** and **Au-4** (Figure 6c) contained four singlet signals that were assigned to [9], [10], [11], and [12]CPPs. The formation of the [10] and [11]CPPs indicates the formation of isosceles triangular macrocyclic complexes in which two different oligophenylene linkers were reorganized from **Au-3** and **Au-4** through the dynamic Au(I)–C bond-exchange process. These complexes were experimentally confirmed by Fourier transform ion cyclotron resonance (FT-ICR) MALDI-TOF MS measurement of the crude products after mixing at 50 °C in DMF (Figure 6e,f).^83^ The NMR yields of [10]CPP (11%) and [11]CPP (17%), which were CPPs derived from heteroleptic Au complexes with different linkers, were slightly lower than those of [9]CPP (26%) and [12]CPP (15%), which are CPPs derived from homoleptic Au complexes with identical linkers (Figure 6c). All of these four CPPs were successfully separated by preparative gel permeation chromatography (PGPC) (see supporting Figure S49). In the **Au-4**/**Au-5** system, the NMR yields of [12]CPP (13%) and [15]CPP (11%) were higher than those of [13]CPP (3.0%) and [14]CPP (3.8%) (Figure 6d), indicating a similar trend to that of the **Au-3**/**Au-4** system. However, the lower yields of [13] and [14]CPPs suggest that the formation of the heteroleptic macrocyclic Au complexes would be more difficult than the **Au-3**/**Au-4** system. We also conducted the transmetalation of [AuCl_2_(dcpm)] (**1**) with a 1:1 mixture of two different oligophenyldiboronic acids (**L3**/**L4** or **L4**/**L5**) (Figure 6a,b, Method B). Oxidation of the product by PhICl_2_ afforded a mixture of CPPs in much lower NMR yields than Method A. These results indicate that the intermolecular transmetalation based on the dynamic Au(I)–C σ bond exchange could occur between not only the acyclic complexes but also the macrocyclic complexes. The fact that the differences in the ratios of the CPP products depend on the linker length in both methods should be attributed to the thermodynamical stability of the corresponding precursor complexes. Our crystallographic study (Figure 2) revealed that the homoleptic Au complexes with long oligophenylene linkers can form *D*_3_-isomers with strong aurophilic interactions, resulting in higher thermodynamic stability than that of the heteroleptic Au complexes to regulate the outcome of self-sorting.^92−87^

In the Pt-mediated method for CPP synthesis (Figure 1c), the formation of CPPs from two different arylene linkers has been achieved by the transmetalation of [PtCl_2_(cod)] with a mixture of two different bis(trimethylstannyl)arylenes followed by the bromine-induced reductive elimination in 0.7–9.8% yields over two steps, which is a similar procedure to Method B in this study.^46,88^ On the other hand, the Au-mediated CPP synthesis outlined in this study represents a new way to an efficient synthesis of [*n*]CPPs with numbers of phenylene units other than multiples of three via mixing two “preorganized” macrocyclic Au complexes (Method A). The higher yields observed in the current Au-mediated method can be attributed to the higher reversibility of the intermolecular transmetalation of Au(I)–C bonds than that of Pt(II)–C bonds.

**Scheme 3:**
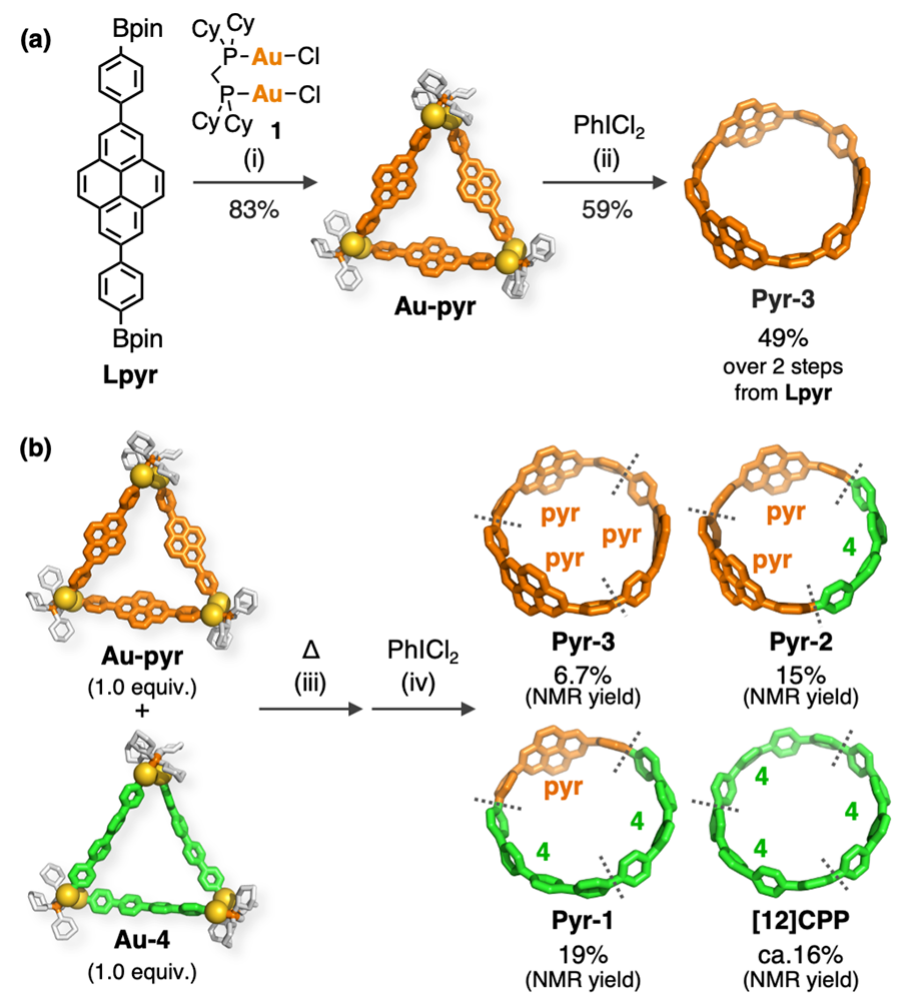
Synthesis of Pyrene-Containing Nanohoops from the Macrocyclic Au Complex; (a) Selective Synthesis of **Pyr-3**; (b) Synthesis of **Pyr-3**, **Pyr-2**, and **Pyr-1** by the Reorganization Process from **Au-pyr** and **Au-4** Reagents and conditions: (i) Cs_2_CO_3_ (6.0 equiv), toluene/ethanol/water (4:1:1), 50 °C, 18 h; (ii) PhICl_2_ (3.0 equiv), CH_2_Cl_2_, −80 °C, 20 min, then r.t., 14 h; (iii) CHCl_3_, 50 °C, 2 days; (iv) PhICl_2_ (3.0 equiv), CHCl_3_, −60 °C, 30 min, then r.t., 2 days; NMR yields of the CPP derivatives in the reorganization method were determined from the ^1^H NMR signal intensities relative to an internal standard (1,2,4,5-tetramethylbenzene, TMB). The NMR yield of [12]CPP is not precise because the signal overlaps with other CPP derivatives.

Finally, to demonstrate the substrate applicability of our synthetic method, we have synthesized new nanohoops with condensed aromatic rings. The transmetalation of pinB–C_6_H_4_–C_16_H_8_–C_6_H_4_–Bpin (**Lpyr**)^55^ and [Au_2_Cl_2_(dcpm)] (**1**) afforded macrocyclic Au complex (**Au-pyr**) in an 83% yield. The oxidative chlorination of **Au-pyr** with PhICl_2_ produced a nanohoop containing three 2,7-pyrenylene units (**Pyr-3**) in a 59% yield (Scheme 3a). The reorganization method between **Au-pyr** and **Au-4** gave three nanohoops with one to three 2,7-pyrenylene units (**Pyr-3**, **Pyr-2**, and **Pyr-1**) along with [12]CPP in 6.7, 15, 19, and ca. 16% NMR yields (Scheme 3b), respectively, which were isolated by PGPC. **Pyr-2** is a structural isomer of the CPP derivative reported by Itami,^90^ in which two 2,7-pyrenylene units are introduced on their opposite sides. These results indicate that the reorganization method developed in this study is effective not only for the synthesis of a series of [*n*]CPPs but also for the synthesis of a variety of CPP derivatives and nanohoops with functional groups introduced at various numbers of substitution and positions.

## Conclusions

We have demonstrated the highly efficient formation of triangular macrocyclic Au complexes, [Au_2_(C_6_H_4_)_*x*_dcpm]_3_ (*x* = 3, 4, 5) by the self-assembly of [Au_2_Cl_2_dcpm] and oligophenylene diboronic acids. The choice of the dcpm ligand instead of the dppm ligand is crucial for achieving the highly selective and high-yielding formation of the triangular complexes. Furthermore, the control experiments and theoretical calculation reveal the role of the dcpm ligand, which increases the Au–aryl exchange reaction by electrophilically activating the gold atom of the Au complex. This is the first example to observe the dynamics of the Au–aryl exchange reaction and its ligand effect. Furthermore, the chemical oxidation of the obtained complexes produced the corresponding [*n*]cycloparaphenylenes ([*n*]CPPs) (*n* = 9, 12, 15) in good overall yields. We have also demonstrated the synthesis of [*n*]CPPs (*n* = 10, 11, 13, 14) and pyrene-containing nanohoops by the reorganization of two different macrocyclic Au complexes. Applications of dynamic Au–C bonds, including the syntheses of a variety of functionalized CPPs and related nanohoops,^91^ are in progress and will be reported in due course.

## Methods

### General

All manipulations were carried out under an argon atmosphere using standard Schlenk techniques. Solvents and reagents were purchased from TCI Co. Ltd, Wako Pure Chemical Industries Ltd, Kanto Chemical Co. Inc., Sigma-Aldrich Co., and Tanaka Kikinzoku Kogyo K.K. NMR spectra were recorded on a Bruker Biospin AVANCE NEO 400 (400 MHz), Bruker Biospin AVANCE II DPX-400 (400 MHz), Bruker Ascend 400 (400 MHz), or JEOL ECZ400S (400 MHz). IR spectra were obtained using a JASCO FT/IR-4600 (ATR). Elemental analyses were performed using a J-science JM10. The high-resolution mass spectrometry (HRMS) spectra were measured in positive ion mode on a JEOL JMS-S3000 SpiralTOF (MALDI-TOF) or a Fourier transformation-ion cyclotron resonance-mass spectrometer, Bruker solariX (FT-ICR-MS), equipped with a 7 Tesla superconductive magnet by using a matrix-assisted laser desorption/ionization (MALDI) ion source. The single X-ray structure determination was performed on a Rigaku XtaLAB Synergy-DW diffractometer or a Bruker D8 QUEST diffractometer. All calculations were carried out using the Gaussian 16 Rev. B. Program package.

#### Synthesis of Oligophenyldiboronic Acids (**L3**, **L4**, and **L5**)

4,4″-Terphenyldiboronic acid pinacol ester (**L3**) was prepared by the Miyaura borylation of 4,4″-dibromoterphenyl with B_2_pin_2_. 4,4‴-Quaterphenyldiboronic acid pinacol ester (**L4**) and 4,4″″-quinquephenyldiboronic acid pinacol ester (**L5**) were synthesized *via* a ring extension from 4,4′-*p*-biphenyldiboronic acid (**L2**) and **L3** by the Suzuki coupling reaction with 2 equivalents of Br–C_6_H_4_–B(dan) (dan = 1,8-diaminonaphthalene), followed by the exchange of the protective groups under acidic conditions (see the Supporting Information for detailed procedures).

#### Synthesis of the Macrocyclic Au Complex, [Au_2_(C_6_H_4_)_*x*_(Cy_2_PCH_2_PCy_2_)]_3_ (X = 3: **Au-3**, *x* = 4: **Au-4**, *x* = 5, **Au-5**)

A mixture of oligophenyldiboronic acid pinacol ester, pinB–(C_6_H_4_)_*x*_–Bpin (*x* = 3: **L3**, *x* = 4: **L4**, *x* = 5, **L5**) (1.0 equiv), Cs_2_CO_3_ (6.0 equiv), and [Au_2_Cl_2_(dcpm)] (**1**) (1.0 equiv) in degassed toluene/H_2_O/EtOH was stirred overnight at 50 °C under an argon atmosphere. After the reaction mixture was allowed to cool to room temperature, the precipitates were collected by suction filtration, washed with EtOH, and then dried *in vacuo*. The Au complex, [Au_2_(C_6_H_4_)_*x*_(Cy_2_PCH_2_PCy_2_)]_3_ (*x* = 3: **Au-3**, *x* = 4: **Au-4**, *x* = 5, **Au-5**), was obtained as a white solid. **Au-3** (*x* = 3): 72% yield. ^1^H NMR (399 MHz, C_2_D_2_Cl_4_, r.t.): δ 7.65 (s, 12H, C_6_H_4_), 7.64–7.59 (m, 12 H, C_6_H_4_), 7.49 (d, 12H, *J* = 7.8 Hz, C_6_H_4_) 2.30–2.21 (br, 12H, C_6_H_11_), 2.14–2.07 (br, 18H, C_6_H_11_ and CH_2_), 1.97–1.91 (br, 24H, C_6_H_11_), 1.79–1.74 (br, 12H, C_6_H_11_), 1.70–1.59 (br, 24H, C_6_H_11,_ overlapping with the signal of H_2_O), 1.57–1.46 (br, 12H, C_6_H_11_), 1.38–1.31 (br, 36 H, C_6_H_11_). ^13^C{^1^H} NMR (100 MHz, C_2_D_2_Cl_4_, r.t.): δ 174.0 (t, *J* = 57.4 Hz), 140.3 (s), 139.9 (s), 136.6 (s), 126.9 (s), 125.2 (s), 35.2 (t, *J* = 14.9 Hz), 29.8 (s), 29.0 (s), 26.7 (d, *J* = 20.4 Hz), 25.8 (s). ^31^P{^1^H} NMR (161 MHz, C_2_D_2_Cl_4_, r.t.): δ 48.2 (s). IR (ATR): ν = 2922, 2848, 1442, 1355, 1006, 795, 756, 511 cm^–1^. Anal. Calcd for C_129_H_174_Au_6_P_6_: C, 50.10; H, 5.67. Found: C, 50.58; H, 5.77. HRMS (FT-ICR MALDI-TOF, DCTB) *m*/*z*: calcd. for C_129_H_174_Au_6_P_6_+H, 3093.0141 [*M* + H]^+^; found, 3093.0170. **Au-4** (*x* = 4): 83% yield. ^1^H NMR (399 MHz, C_2_D_2_Cl_4_, r.t.): δ 7.70 (s, 24H, C_6_H_4_), 7.67–7.61 (m, 12H, C_6_H_4_), 7.50 (d, 12 H, *J* = 7.6 Hz, C_6_H_4_), 2.31–2.20 (br, 12H, C_6_H_11_), 2.15–2.07 (br, 18H, C_6_H_11_ and CH_2_), 1.98–1.90 (br, 24H, C_6_H_11_), 1.82–1.75 (br, 12H, C_6_H_11_), 1.71–1.60 (br, 24H, C_6_H_11,_ overlapping with the signal of H_2_O), 1.59–1.46 (br, 12H, C_6_H_11_) 1.40–1.30 (br, 36H, C_6_H_11_). ^13^C{^1^H} NMR (100 MHz, C_2_D_2_Cl_4_, r.t.): δ 174.3 (t, *J* = 57.7 Hz), 140.7 (s), 140.3 (s), 138.5 (s), 136.4 (s), 127.1 (s), 127.0 (s), 125.2 (s), 35.2 (t, *J* = 13.7 Hz), 29.8 (s), 29.0 (s), 26.7 (d, *J* = 21.4 Hz), 25.8 (s). ^31^P{^1^H} NMR (161 MHz, C_2_D_2_Cl_4_, r.t.): δ 48.3 (s). IR (ATR): ν = 2924, 2849, 1447, 1218, 1160, 1004, 803, 760, 513 cm^–1^. Anal. Calcd for C_147_H_186_Au_6_P_6_ 6H_2_O: C, 51.39; H, 6.01. Found: C, 51.48; H, 6.13. HRMS (FT-ICR MALDI-TOF, DCTB) *m*/*z*: calcd for C_147_H_186_Au_6_P_6_+H, 3321.1080 [*M* + H]^+^; found, 3321.1061. **Au-5** (*x* = 5): 72% yield. ^1^H NMR (399 MHz, C_2_D_2_Cl_4_, r.t.): δ 7.76–7.69 (br, 36H, C_6_H_4_), 7.67–7.59 (br, 12H, C_6_H_4_), 7.55–7.48 (br, 12 H, C_6_H_4_), 2.21–2.02 (br, 30H, C_6_H_11_, CH_2_), 1.98–1.91 (br, 24H, C_6_H_11_), 1.81–1.75 (br, 12H, C_6_H_11_), 1.68–1.56 (br, 24H, C_6_H_11,_ overlapping with signal of H_2_O), 1.40–1.29 (br, 48H, C_6_H_11_). ^31^P{^1^H} NMR (161 MHz, C_2_D_2_Cl_4_, r.t.): δ 48.3 (s). IR (ATR): ν 2924, 2850, 1480, 1446, 1006, 801, 756, 512 cm^–1^. Anal. Calcd for C_165_H_198_Au_6_P_6_ 3H_2_O: C, 55.00; H, 5.71. Found: C, 55.12; H, 6.04. HRMS (FT-ICR MALDI-TOF, DCTB) *m*/*z*: calcd for C_165_H_198_Au_6_P_6_+H, 3549.2019 [*M* + H]^+^; found, 3549.2084.

#### Synthesis of [*n*]CPP (*n* = 9, 12, 15)

To a suspension of the Au complex, [Au_2_(C_6_H_4_)_*x*_(Cy_2_PCH_2_PCy_2_)]_3_ (*x* = 3: **Au-3**, *x* = 4: **Au-4**, *x* = 5, **Au-5**), (1.0 equiv) in degassed DMF was added PhICl_2_ (6.0 mmol/L in DMF, 6.0 equiv) dropwise with stirring at −60 °C for 5 min under an argon atmosphere. The reaction mixture was stirred at the same temperature for 30 min, and then it was allowed to warm to 25 °C and stirred for overnight. The solvent and iodobenzene (byproduct) were removed under vacuum. The crude product was purified by silica gel column chromatography (eluent; CHCl_3_) to give [*n*]cycloparaphenylene (*n* = 9, 12, 15) and [Au_2_Cl_2_(dcpm)]. **[9]CPP**; 78% yield. ^1^H NMR (400 MHz, CDCl_3_, r.t.): δ 7.52 (s, 36H, C_6_H_4_). ^13^C{^1^H} NMR (100 MHz, CDCl_3_, r.t.): δ 138.1 (s), 127.5 (s). **[12]CPP**; 78% yield. ^1^H NMR (400 MHz, CDCl_3_, r.t.): δ 7.61 (s, 48H, C_6_H_4_). ^13^C{^1^H} NMR (100 MHz, CDCl_3_, r.t.): δ 138.7 (s), 127.5 (s). **[15]CPP**; 88% yield. ^1^H NMR (400 MHz, CDCl_3_, r.t.): δ 7.67 (s, 60H, C_6_H_4_). ^13^C{^1^H} NMR (100 MHz, CDCl_3_, r.t.): δ 139.0 (s), 127.5 (s).

### General Procedure on NMR Experiments for Determination of the Rate Constants

A solution of [Au_2_Ph_2_(dcpm)] (**Au**_**C**_**-HH**) (1.0 mg, 1.0 μmol) in 0.3 mL of CDCl_3_ (0.001% v/v 1,3,5-tris(trifluoromethyl)benzene) was injected into an NMR test tube and then cooled to −20 °C. To the test tube, a cooled solution of [Au_2_(4-F-C_6_H_4_)_2_(dcpm)] (**Au**_**C**_**–HF**) (1.0 mg, 1.0 μmol) in 0.3 mL of CDCl_3_ (0.001% v/v 1,3,5-tris(trifluoromethyl)benzene) was added. The test tube was inserted to an NMR spectrometer which was precooled to −20 °C. The progress of the reaction was monitored by NMR measurements.

#### Synthesis of CPPs from Two Different Oligophenylene Linkers: Reorganization Approach (Method A)

To a suspension of **Au-*x*** (1.0 equiv) and **Au-*y*** (1.0 equiv) (*x* = 3, *y* = 4 or *x* = 4, *y* = 5) in degassed DMF was stirred for 6 days at 50 °C under an argon atmosphere. After the reaction mixture was allowed to cool to −60 °C, PhICl_2_ (6.0 mmol/L in DMF, 6.0 equiv) was added dropwise with stirring at the same temperature for 5 min. The reaction mixture was stirred at the same temperature for 30 min, and then it was allowed to warm to 25 °C and stirred for 6 h. The solvent and iodobenzene (byproduct) were removed under vacuum. The crude product was purified by silica gel column chromatography (eluent; CHCl_3_) to give a mixture of CPPs as a yellow solid. ^1^H NMR analysis indicated the formation of [*n*]CPPs. **Au-3**/**Au-4** system: [9]CPP (26%), [10]CPP (11%), [11]CPP (17%), and [12]CPP (15%). **Au-4**/**Au-5** system: [12]CPP (13%), [13]CPP (3.0%), [14]CPP (3.8%), and [15]CPP (11%).

#### Synthesis of CPPs from Two Different Oligophenylene Linkers: Social Self-Sorting Approach (Method B)

A mixture of 4,4″-*p*-terphenyldiboronic acid pinacol ester (**L3**) (42.4 mg, 0.088 mmol), 4,4″′-*p*-quaterphenyldiboronic acid pinacol ester (**L4**) (49.0 mg, 0.088 mmol), Cs_2_CO_3_ (12 equiv), and [Au_2_Cl_2_(dcpm)] (**1**) (2.0 equiv) in degassed toluene/H_2_O/EtOH was stirred for 4 days at 50 °C under an argon atmosphere. After the reaction mixture was allowed to cool to room temperature, the precipitate was collected by suction filtration and washed with toluene, H_2_O, EtOH, and then dried in *vacuo*. The mixture of Au complexes was obtained as a dark-green solid, which was used in the next reaction without further purification. To a suspension of the mixture of Au complexes (1.0 equiv) in degassed DMF was added PhICl_2_ (12 mmol/L in DMF, 3.0 equiv) dropwise with stirring at −60 °C for 5 min. The reaction mixture was stirred at the same temperature for 30 min, and then it was allowed to warm to 25 °C and stirred overnight. The solvent and iodobenzene (byproduct) were removed under vacuum. The crude product was purified by silica gel column chromatography (eluent; CHCl_3_) to give a mixture of CPPs as a yellow solid (*R*_f_ = 0.76, 0.95 mg). ^1^H NMR analysis indicated the formation of [*n*]CPPs. **L3**/**L4** system: [9]CPP (1.1%), [10]CPP (1.7%), [11]CPP (2.5%), and [12]CPP (1.0%). **L4**/**L5** system: [12]CPP (12%), [13]CPP (1.0%), [14]CPP (1.5%), and [15]CPP (7.9%).

#### Synthesis of [Au_2_(C_6_H_4_–C_16_H_8_–C_6_H_4_)(Cy_2_PCH_2_PCy_2_)]_3_ (Au-pyr)

A mixture of pinB-C_6_H_4_-C_16_H_8_-C_6_H_4_-Bpin (**Lpyr**)^55^ (243 mg, 0.40 mmol), Cs_2_CO_3_ (792 mg, 2.4 mmol), and [Au_2_Cl_2_(dcpm)] (349 mg, 0.40 mmol) in degassed toluene/H_2_O/EtOH (16/4/4 mL) was stirred for 2 days at 50 °C under an argon atmosphere. After the reaction mixture was allowed to cool to room temperature, the precipitates were collected by suction filtration, washed with toluene (30 mL), H_2_O (20 mL), and EtOH (20 mL), and then dried in *vacuo*. The Au complex, [Au_2_(C_6_H_4_–C_16_H_8_–C_6_H_4_)(Cy_2_PCH_2_PCy_2_)]_3_ (**Au-pyr**), was obtained as an off-white solid (383 mg, 0.11 mmol, 83%).

^1^H NMR and ^31^P NMR spectra suggest that the two isomers are exchanged slower than the NMR timescale (*C*_*2*_/*D*_*3*_ = 1:1) (see Figure S18 for detail). Data for the dynamic mixture of *C*_2_- and *D*_3_-isomers of **Au-pyr**; ^1^H NMR (400 MHz, CDCl_3_, r.t.): δ 8.39 (s, 12H, C_16_H_8_ for *C*_*2*_), 8.07 (s, 12H, C_16_H_8_ for *C*_*2*_), 7.84 (s, 12 H, C_16_H_8_ for *D*_*3*_), 7.76 (s, 24 H, C_6_H_4_ for *C*_*2*_), 7.46 (br, 24 H, C_6_H_4_ for *D*_*3*_), 7.10 (s, 12H, C_16_H_8_ for *D*_*3*_), 2.36–2.04 (br, 54H, C_6_H_11_, CH_2_), 2.02–1.86 (br, 24H, C_6_H_11_), 1.84–1.48 (br, 12H, C_6_H_11,_ overlapping with signal of H_2_O), 1.45–1.23 (br, 48H, C_6_H_11_). ^31^P{^1^H} NMR (161 MHz, CDCl_3_, r.t.): δ 48.1 (s, *D*_*3*_), 47.9 (s, *C*_*2*_). Anal. Calcd for C_159_H_186_Au_6_P_6_ 5H_2_O: C, 53.72; H, 5.56. Found: C, 53.52; H, 5.37.

#### Synthesis of Pyrene-Containing Nanohoop, (Pyr-3)

To a suspension of [Au_2_(C_6_H_4_–C_16_H_8_–C_6_H_4_)(Cy_2_PCH_2_PCy_2_)]_3_ (**Au-pyr**) (350 mg, 0.10 mmol) in degassed CH_2_Cl_2_ (100 mL) was added PhICl_2_ (10 mmol/L in CH_2_Cl_2_, 30 mL, 0.30 mmol) dropwise with stirring at −80 °C for 20 min under an argon atmosphere. The reaction mixture was stirred at the same temperature for 30 min, and then it was allowed to warm to 25 °C and stirred for 14 h. After the solvent was removed under vacuum, the crude product was purified by silica gel column chromatography (eluent; CHCl_3_) to give pyrene-containing nanohoop, (**Pyr-3**) (*R*_f_ = 0.87, 62.1 mg, 59 μmol, 59%) as a pale-yellow solid, and [Au_2_Cl_2_(dcpm)] (*R*_f_ = 0.15, 220 mg, 25 mmol, 84%) as a white solid. ^1^H NMR (400 MHz, CDCl_3_, r.t.): δ 8.26 (s, 12H, C_16_H_8_), 7.94 (s, 12H, C_16_H_8_), 7.78 (d, 12 H, *J* = 8.4 Hz, C_6_H_4_), 7.61 (d, 12 H, *J* = 8.4 Hz, C_6_H_4_). ^13^C{^1^H} NMR (101 MHz, CDCl_3_, r.t.): δ 139.0 (s), 138.7 (s), 137.1 (s), 131.7 (s), 128.0 (s), 127.8 (s), 127.7 (s), 124.0 (s), 123.8 (s). HRMS (MALDI-TOF, DCTB) *m*/*z*: calcd for C_84_H_48_, 1056.3751 [*M*]^+^; found, 1056.3760.

#### Synthesis of Pyrene-Containing Nanohoops (**Pyr-1**, **Pyr-2**, and **Pyr-3**) by Reorganization Method

A suspension of **Au-4** (133 mg, 40 μmol) and **Au-pyr** (139 mg, 40 μmol) in degassed CHCl_3_ (80 mL) was stirred for 2 days at 50 °C under an argon atmosphere. After the reaction mixture was allowed to cool to −60 °C, PhICl_2_ (24 mmol/L in CHCl_3_, 10 mL, 0.24 mmol) was added dropwise with stirring at the same temperature for 5 min. The reaction mixture was stirred at the same temperature for 30 min, and then it was allowed to warm to 25 °C and stirred for 2 days. After the solvent was removed under vacuum, the crude product was purified by silica gel column chromatography (eluent: CHCl_3_) to give a mixture of nanohoops as a yellow solid (52.8 mg) and [Au_2_Cl_2_(dcpm)] as a white solid (152 mg, 0.17 mmol, 73%). ^1^H NMR analysis indicated the formation of [12]CPP, **Pyr-1**, **Pyr-2**, and **Pyr-3** in ca. 16, 29, 15, and 6.7% yields over 2 steps, respectively (the NMR yield of [12]CPP is not precise because the signal overlaps with other CPP derivatives). A portion of the mixture (19.4 mg) was purified by PGPC (eluent: CHCl_3_), to give **Pyr-1** (2.69 mg, 2.8 μmol) and **Pyr-2** (2.51 mg, 2.5 μmol) in a pure form. Data for **Pyr-1**: ^1^H NMR (400 MHz, CDCl_3_, r.t.): δ 8.28 (s, 4H, C_16_H_8_), 7.99 (s, 4H, C_16_H_8_) 7.79 (d, 4H, *J* = 8.6 Hz, C_6_H_4_), 7.64–7.59 (m, 36H, C_6_H_4_). ^13^C{^1^H} NMR (101 MHz, CDCl_3_, r.t.): δ 139.3 (s), 138.7 (m), 137.5 (s), 131.9 (s), 129.2 (s), 128.3 (s), 127.9 (s), 127.8 (s), 127.7 (s), 127.5 (m), 124.1 (s). HRMS (MALDI-TOF, Dithranol) *m*/*z*: calcd for C_76_H_48_, 960.3775 [*M*^+^]; found: 960.3751. Data for **Pyr-2**: ^1^H NMR (400 MHz, CDCl_3_, r.t.): δ 8.28 (s, 4H, C_16_H_8_), 8.25 (s, 4H, C_16_H_8_) 7.97 (s, 8H, C_16_H_8_), 7.80 (d, 4H, *J* = 8.7 Hz, C_6_H_4_), 7.76 (d, 4H, *J* = 8.6 Hz, C_6_H_4_), 7.63–7.54 (m, 24H, C_6_H_4_). ^13^C{^1^H} NMR (101 MHz, CDCl_3_, r.t.): δ 139.4 (s), 139.1 (s), 138.9 (s), 138.8 (s), 138.8 (s), 138.5 (s), 138.5 (s), 137.5 (s), 137.2 (s), 131.9 (s), 131.8 (s), 129.2 (s), 128.4 (s), 128.3 (s), 128.2 (s), 128.0 (s), 127.8 (s), 127.8 (s), 127.7 (s), 127.6 (s), 127.4 (s), 125.4 (s), 124.1 (s), 124.1 (s), 124.1 (s), 123.9 (s). HRMS (MALDI-TOF, dithranol) *m*/*z*: calcd for C_80_H_48_, 1008.3751 [*M*^+^]; found, 1008.3748.

## Supporting Information Available

Experimental procedures, NMR spectra, theoretical studies, and X-ray crystallography data (PDF)

## Accession Codes

CCDC 2108728 (**Au-3**), 2108731 (**Au-4*D***_**3**_), 2108730 (**Au-4*C***_**2**_), 2108732 (**Au**_**C**_**-FF**), 2108733 (**Au**_**P**_**-FF**), and 2108729 (**Au**_**C**_**-HH**) contain the supplementary crystallographic data for this paper. These data can be obtained free of charge via www.ccdc.cam.ac.uk/data_request/cif, by emailing data_request@ccdc.cam.ac.uk, or by contacting The Cambridge Crystallographic Data Centre, 12 Union Road, Cambridge CB2 1EZ, UK; fax: +44 1223 336033.
