# Supplementary material for: Dynamic Au–C σ-Bonds Leading to an Efficient Synthesis of [n]Cycloparaphenylenes (n = 9–15) by Self-Assembly
Source: JACS Au. 2022 Jul 11;2(8):1857–68. doi: 10.1021/jacsau.2c00194 (PMC9400051; doi:10.1021/jacsau.2c00194)
Supplement: Supplementary file 1 — au2c00194_si_001.pdf [file au2c00194_si_001.pdf]

Supporting Information of

Dynamic Au–C σ-Bonds Leading to an Efficient Synthesis of [*n*]Cycloparaphenylenes (n = 9 – 15) by a Self-Assembly

Yusuke Yoshigoe,*[a] Yohei Tanji,[a] Yusei Hata,[a] Kohtaro Osakada,[b] Shinichi Saito,[a] Eiichi Kayahara,[c] Shigeru Yamago,[c] Yoshitaka Tsuchido,*[a] and Hidetoshi Kawai*[a]

[a] Department of Chemistry, Faculty of Science, Tokyo University of Science, 1–3 Kagurazaka, Shinjuku-ku, Tokyo 162-8601, Japan

[b] Laboratory for Chemistry and Life Science, Institute of Innovative Research, Tokyo Institute of Technology, 4259, Nagatsuta, Midori-ku, Yokohama 226-8503, Japan

[c] Institute for Chemical Research, Kyoto University, Uji, Kyoto 611-0011, Japan

Contents

S1 General.

S2. Synthesis and characterization

S3. Kinetics study

S4. Synthesis of CPPs from two different oligophenylene linkers

S5. Crystallographic study

S6. Computational details

S7. References

S1. General

All manipulations were carried out under an argon atmosphere using standard Schlenk techniques. The ^{1}H, $^{13}C\{^{1}H\}$, $^{19}F\{^{1}H\}$ and $^{31}P\{^{1}H\}$ NMR spectra were recorded on a Bruker Biospin AVANCE NEO 400 (400 MHz), Bruker Biospin AVANCE II DPX-400 (400 MHz), Bruker ascend™ 400 (400 MHz), or JEOL ECZ400S (400 MHz). The chemical sifts in ^{1}H and $^{13}C\{^{1}H\}$ NMR were referenced with respect to residual peaks of the solvents used (^{1}H NMR; δ 7.26 for $CDCl_3$, δ 6.00 for $C_2D_2Cl_4$. ^{13}C NMR; δ 77.16 for $CDCl_3$, δ 73.70 for $C_2D_2Cl_4$). The peak positions of the $^{31}P\{^{1}H\}$ NMR spectra were referenced to external 85% H_3PO_4 (δ 0) in deuterated solvents, and of the $^{19}F\{^{1}H\}$ NMR spectra were referenced to external $BF_3 \cdot OEt_2$ (δ 0) in deuterated solvents or internal 1,3,5-tris(trifluoromethyl)benzene (δ -63.5). Deuterated solvents were purchased from Wako, Kanto, or ISOTEC and used as received. IR spectra were taken on a JASCO FT/IR-4600 (ATR). Elemental analyses were performed using a J-science JM10. The high resolution mass spectrometry (HRMS) were measured in positive ion mode on a JEOL JMS-S3000 SpiralTOF (MALDI-TOF), or a Fourier transformation-ion cyclotron resonance-mass spectrometer, Bruker solariX (FT-ICR-MS) equipped with 7 tesla superconductive magnet by using a matrix-assisted laser desorption/ionisation (MALDI) ion source. Samples were prepared from a THF solution by mixing a sample (1 mg/mL) and a matrix (dithranol or *trans*-2-[3-(4-*tert*-butylphenyl)-2-methyl-2-propenylidene]malononitrile) (10 mg/mL) in a 1:1 ratio. The purification of Preparative Gel Permeation Chromatography (PGPC) was carried out on LC-908 with JAIGEL-1HH+2HH columns eluted with $CHCl_3$. The GCMS analysis was carried out on a SHIMADZU GCMS-QP2010 SE coupled with AOC-20i auto-sampler, and a COLUMN, UA1 MS/HT, 30M x 0.25 MM ID x 0.25 UM capillary column, (30 m x 0.25 mm i.d.,

0.25 μm). The initial temperature of column was 50 °C held for 1 min. and was programmed to 300 °C at 10 °C/min., then held for 5 min. at 300 °C. The sample injection volume was 1 μL in AcOEt. Helium was used as carrier gas at a flow rate of 1.7 mL/min.

The commercially available reagents, $HAuCl_4$ $4H_2O$ (Tanaka), tetrahydrothiophene (TCI), bis(dicyclohexylphosphino)methane (Wako), bis(diphenylphosphino)methane (TCI), 4,4''-dibromo-*p*-terphenyl (TCI), B_2pin_2 (ChemICHIBA), 2-(4-bromophenyl)-2,3-dihydro-1*H*-naphtho[1,8-*de*][1,3,2]diazaborine (TCI), phenyl boronic acid (TCI), 4-fluorophenyl boronic acid (BLD Pharm), phenyl-*d*5boronic acid (Wako), 4,4'-diphenylene diboronic acid (TCI), $Pd(OAc)_2$ (Aldrich), $Pd(P^tBu_3)_2$ (Wako), SPhos (Kanto), CsF (Aldrich), Cs_2CO_3 (Wako), KOAc (Kanto), pinacol (Kanto), 1,2,4,5-tetrabromobenzene (TCI), 1,3,5-tris(trifluoromethyl)benzene (TCI), and iodobenzene (TCI) were used as received. $PhICl_2$ was prepared from the oxidation of iodobenzene by Cl_2, which was generated *in situ* from the reaction of 5% NaClO (commercial household bleach) aq. with conc. HCl.[S1]

S2. Synthesis and Characterization

4,4''-Terphenyldiboronic acid pinacol ester (**L3**) was prepared by the Miyaura bolylation of 4,4''-dibromoterphenyl with B_2pin_2.[S2] 4,4'''-Quaterphenyldiboronic acid pinacol ester (**L4**) and 4,4''''-quinquephenyldiboronic acid pinacol ester (**L5**) were synthesized *via* an ring extension from **L2** and **L3** by Suzuki coupling reaction with two molar amounts of Br-C_6H_4-B(dan) (dan = 1,8-diaminonaphthalene)[S3], followed by the exchange of the protective groups under acidic condition[S4] (Scheme S1).

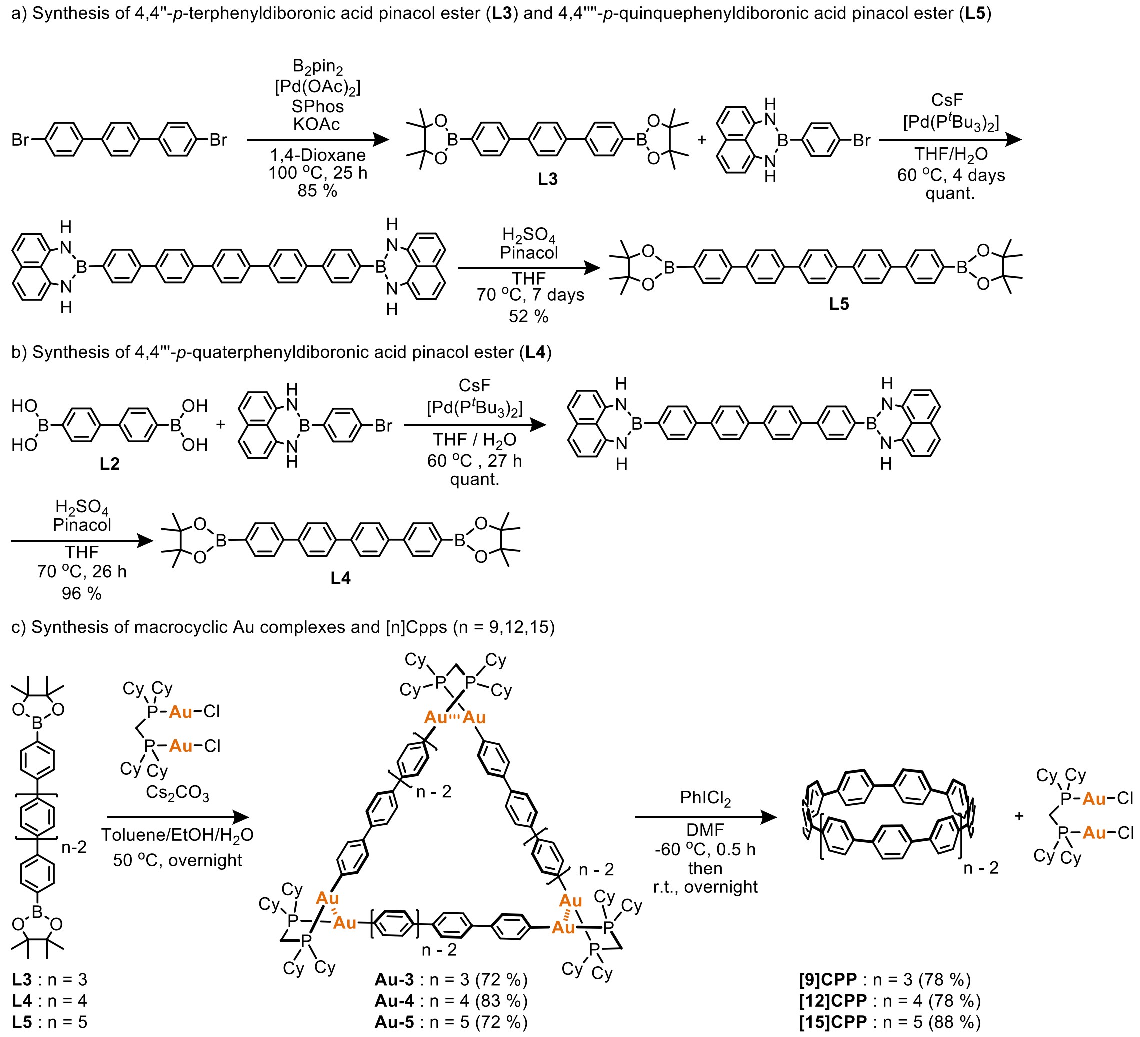

Scheme S1. Synthetic routes for oligophenyldiboronic acids (**L3**, **L4**, **L5**) and [*n*]CPPs (n = 9, 12, 15) via the macrocyclic Au complexes.

Synthesis of [AuCl(tht)] (tht = tetrahydrothiophene).[S5]

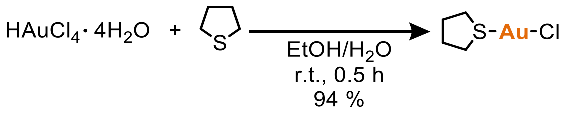

To a EtOH (39 mL) and H_2O (9 mL) solution of $HAuCl_4$·$4H_2O$ (4.98 g, 12 mmol) was added tetrahydrothiophene (2.22 g, 25 mmol) at ambient temperature. The reaction mixture was stirred for 0.5 hour at the same temperature. A white precipitate was formed during the reaction, which was collected by suction filtration and washed with EtOH (30 mL). The residue was dried under vacuum. [AuCl(tht)] (3.66 g, 11 mmol, 94%) was obtained as a white solid.

Synthesis of [Au_2Cl_2(dcpm)] (1).[S6]

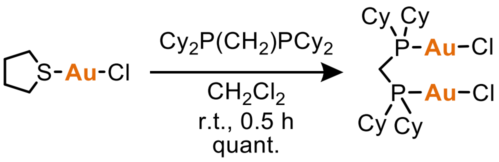

To a CH_2Cl_2 solution (50 mL) of [AuCl(tht)] (3.19 g, 10 mmol) was added bis(dicyclohexylphosphino)methane (dcpm) (2.09 g, 5.1 mmol) at ambient temperature. After the reaction mixture was stirred for 30 min, hexane (150 mL) was added to the mixture. The precipitate was collected by suction filtration to give [Au_2Cl_2(dcpm)] (**1**) (4.33 g, 5.0 mmol, quant.) as a white solid. ^{1}H NMR (500 MHz, $CDCl_3$, r.t.): δ 2.21-2.18 (br, 4H, C_6H_{11}), 2.08 (t, 2H, J = 10.3 Hz), 2.05-1.97 (br, 8H, C_6H_{11}), 1.96-1.88 (br, 8H C_6H_{11}), 1.79-1.71 (br, 4H, C_6H_{11}), 1.54-1.21 (br, 20H, C_6H_{11}). ^{31}P{^{1}H} NMR (202 MHz, $CDCl_3$, r.t.): δ 44.1 (s).

Synthesis of [Au_2Cl_2(dppm)] (1').[S7]

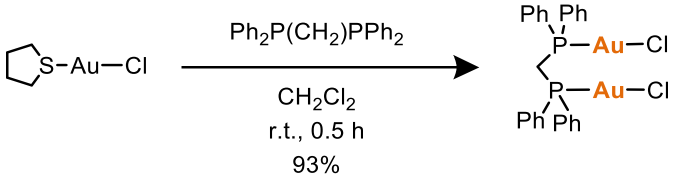

[Au_2Cl_2(dppm)] (**1'**) was synthesized referred to the literature procedure of the synthesis of [Au_2Cl_2(dcpm)] (**1**) as follows. To a CH_2Cl_2 solution (5 mL) of [AuCl(tht)] (324 mg, 1.0 mmol) was added bis(diphenylphosphino)methane (dppm) (192 mg, 0.5 mmol) at ambient temperature. After the reaction mixture was stirred for 30 min, Et_2O (50 mL) was added to the mixture. The precipitate was collected by suction filtration to give [Au_2Cl_2(dppm)] (**1'**) (396 mg, 0.47 mmol, 93%) as a white solid. Purity was confirmed by comparison of ^{1}H NMR spectrum of the product with a reported spectrum.[S7] ^{1}H NMR (400 MHz, $CDCl_3$, r.t.): δ 7.74–7.63 (m, 8H, Ar-H), 7.57–7.49 (m, 4H, Ar-H), 7.48–7.40 (m, 8H, Ar-H), 3.65 (t, J = 11.3 Hz, 2H, CH_2); ^{31}P{^{1}H} NMR (162 MHz, $CDCl_3$, r.t.): δ 26.5.

Synthesis of 4,4''-*p*-terphenyldiboronic acid pinacol ester[S2]

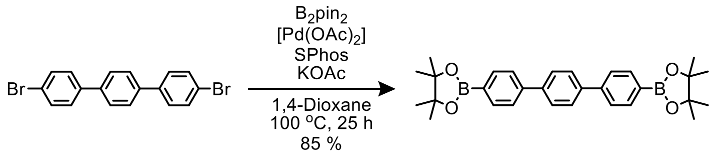

A mixture of 4,4''-dibromo-*p*-terphenyl (1.55 g, 4.0 mmol), bis(pinacolato)diboron (4.05 g, 16 mmol), KOAc (1.98 g, 20 mmol), SPhos (165 mg, 0.40 mmol) and [Pd(OAc)$_2$] (38.5 mg, 0.17 mmol) in degassed 1,4-dioxane (15 mL) was stirred for 25 hours at 100 °C under an argon atmosphere. After the reaction mixture allowed to cool to room temperature, solvent was removed under vacuum. $CHCl_3$ (150 mL) and H_2O (150 mL) was added to the mixture. The aqueous layer separated from the organic layer and

extracted with $CHCl_3$ (150 mL x 3). The organic layer combined with the extracts, washed with water and brine, dried over $MgSO_4$, and then filtered. After the filtrate was concentrated, MeOH (50 mL) was added to the filtrate. The precipitate was collected by suction filtration, then dried in *vacuo*. 4,4''-*p*-Terphenyldiboronic acid pinacol ester (1.63 g, 3.4 mmol, 85%) was obtained as a white solid. ^{1}H NMR[S2] (400 MHz, $CDCl_3$, r.t.): δ 7.90 (d, 4H, J = 8.2 Hz, C_6H_4), 7.70 (s, 4H, C_6H_4), 7.66 (d, 4H, J = 8.3 Hz C_6H_4), 1.37 (s, 24H, CH_3).

Synthesis of 1,8-diaminonaphthalene protected 4,4'''-*p*-quaterphenyldiboronic acid.

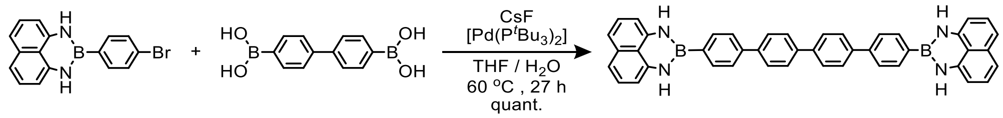

A mixture of 4,4'-*p*-biphenyldiboronic acid (**L2**) (171 mg, 0.71 mmol), 2-(4-bromophenyl)-2,3-dihydro-1*H*-naphtho[1,8-*de*][1,3,2]diazaborine (606 mg, 1.9 mmol), CsF (540 mg, 3.8 mmol) and [Pd(P^{t}Bu$_3$)$_2$] (36.0 mg, 0.070 mmol) in degassed THF/H_2O (5 mL/0.7 mL) was stirred for 27 hours at 60 °C under an argon atmosphere. After the reaction mixture allowed to cool to room temperature, the precipitates were collected by suction filtration and washed with MeOH (10 mL), then dried in *vacuo*. 1,8-Diaminonaphthalene protected 4,4'''-*p*-quaterphenyldiboronic acid (450 mg, 0.71 mmol, quant.) was obtained as a yellow solid. The product was insoluble in various organic solvents, which was used in the next reaction without further purification and characterization.

Synthesis of 4,4'''-*p*-quaterphenyldiboronic acid pinacol ester (L4).

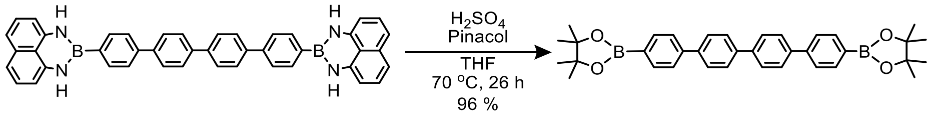

A mixture of 1,8-diaminonaphthalene protected 4,4'''-*p*-quaterphenyldiboronic acid (450 mg, 0.70 mmol), pinacol (1.69 g, 14 mmol) and H_2SO_4 aq (4 M, 2.3 mL, 11 mmol) in THF (10 mL) was stirred for 26 hours at 70 °C. After the reaction mixture allowed to cool to room temperature, H_2O (30 mL) was added to the mixture. The precipitates were collected by suction filtration and washed with MeOH (10 mL), then dried in *vacuo*. 4,4'''-*p*-Quaterphenyldiboronic acid pinacol ester (**L4**) (380 mg, 0.68 mmol, 96%) was obtained as a white solid. ^{1}H NMR (400 MHz, $CDCl_3$, r.t.): δ 7.91 (d, 4H, J = 8.2 Hz, C_6H_4), 7.73 (s, 8H, C_6H_4), 7.67 (d, 4H, J = 8.2 Hz, C_6H_4), 1.37 (s, 24H, CH_3). ^{13}C{^{1}H} NMR (100 MHz, $CDCl_3$, r.t.): δ 143.5 (s), 140.2 (s), 140.0 (s) 135.5 (s), 127.8 (s), 127.6 (s), 126.5 (s), 84.0 (s), 25.0 (s). IR (ATR): ν = 2979, 2931, 1606, 1522, 1361, 1268, 1145, 1093, 1011, 961, 857, 810, 746, 662 cm^{-1}. HRMS (MALDI-TOF, DCTB): m/z: calcd for $C_{36}H_{40}B_2O_4$: 558.3119 [M^+]; found: 558.3099

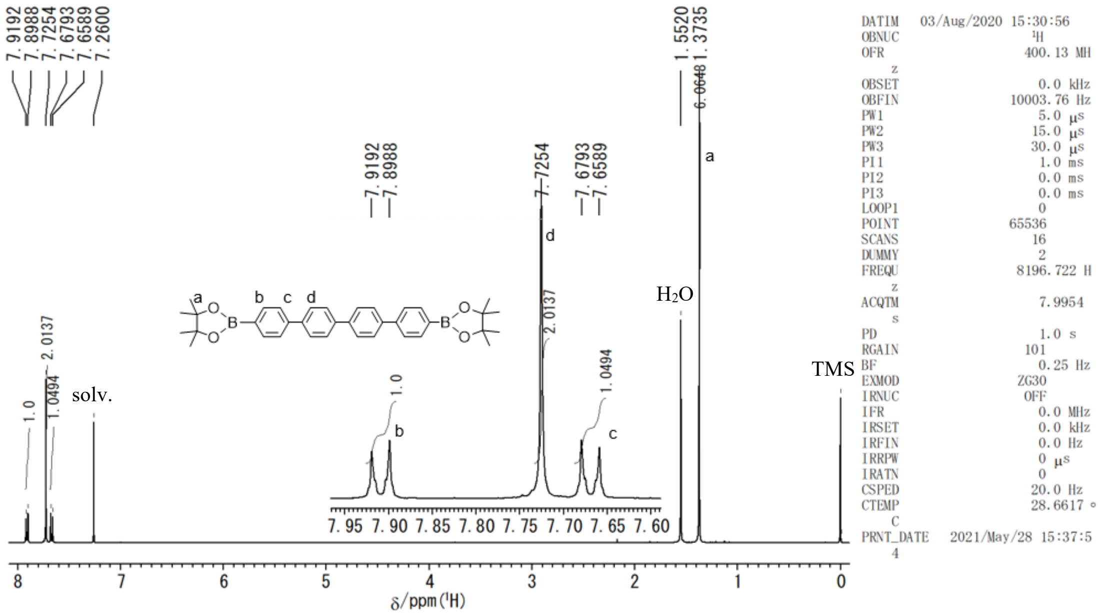

Figure S1. ^{1}H NMR spectrum of **L4** (400 MHz, $CDCl_3$, r.t.).

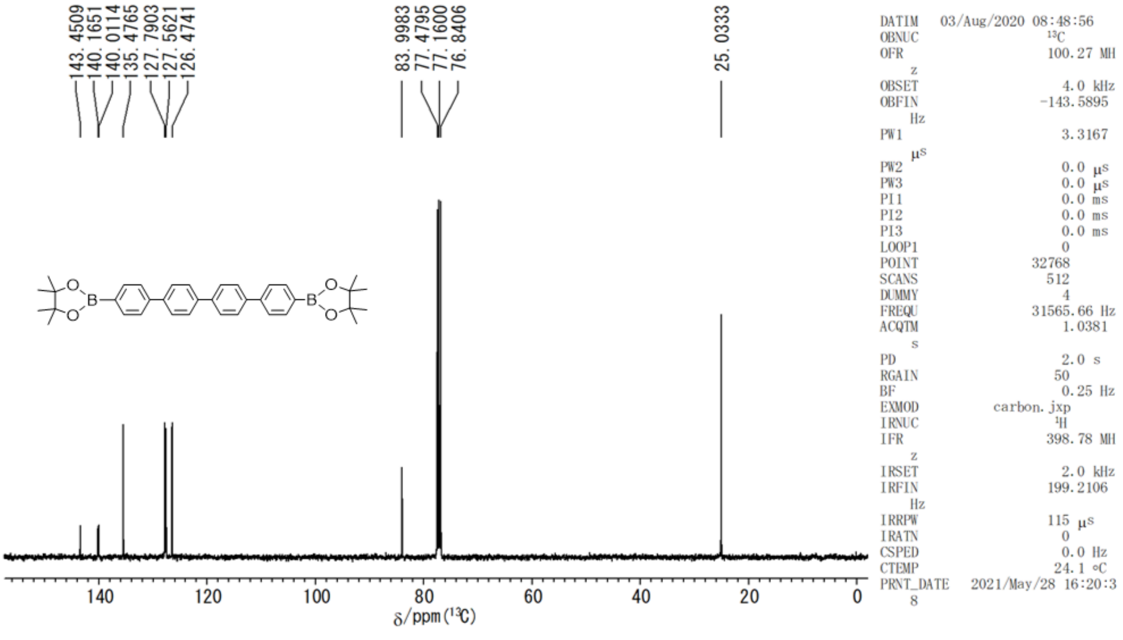

Figure S2. $^{13}C\{^1H\}$ NMR spectrum of **L4** (100 MHz, $CDCl_3$, r.t.).

Synthesis of 1,8-diaminonaphthalene protected 4,4''''-*p*-quinquephenyldiboronic acid.

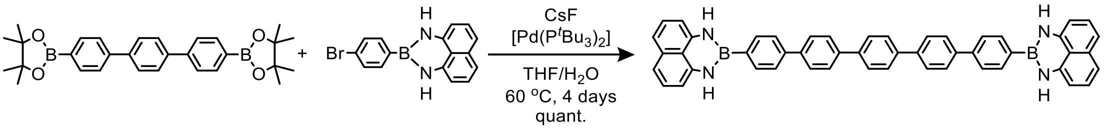

A mixture of 4,4''-*p*-terphenyldiboronic acid pinacol ester (**L3**) (97.5 mg, 0.20 mmol), 2-(4-bromophenyl)-2,3-dihydro-1*H*-naphtho[1,8-*de*][1,3,2]diazaborine (162 mg, 0.50 mmol), CsF (150 mg, 1.1 mmol) and [Pd(P^{t}Bu$_3$)$_2$] (10.3 mg, 0.020 mmol) in degassed THF/H_2O (4 mL/0.8 mL) was stirred for 4 days at 60 °C under an argon atmosphere. After the reaction mixture allowed to cool to room temperature, the precipitates were collected by suction filtration and washed with THF (3 mL), H_2O (3 mL), and EtOH (3 mL), then dried in *vacuo*. 1,8-Diaminonaphthalene protected 4,4''''-*p*-quinquephenyldiboronic acid (148 mg, 0.21 mmol, quant.) was obtained as a green solid. The product was insoluble in various organic solvents, which was used in the next reaction without further purification and characterization.

Synthesis of 4,4''''-*p*-quinquephenyldiboronic acid pinacol ester (L5).

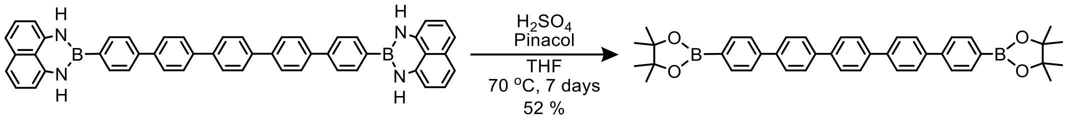

A mixture of 1,8-diaminonaphthalene protected 4,4''''-*p*-quinquephenyldiboronic acid (72.2 mg, 0.10 mmol), pinacol (250 mg, 2.1 mmol) and H_2SO_4 aq. (4 M, 0.4 mL, 1.6 mmol) in THF (6 mL) was stirred for 7 days at 70 °C. After the reaction mixture allowed to cool to room temperature, H_2O (20 mL) was added to the mixture. The precipitates were collected by suction filtration, and then the residue was extracted with $CHCl_3$ (10 mL). The filtrate was concentrated and dried under vacuum. 4,4''''-*p*-

Quinquephenyldiboronic acid pinacol ester (**L5**) (33.2 mg, 0.052 mmol, 52%) was obtained as a white solid.

^{1}H NMR (400 MHz, $CDCl_3$, r.t.): δ 7.91 (d, 4H, J = 8.3 Hz, C_6H_4), 7.76-7.73 (m, 12H, C_6H_4) 7.68 (d, 4H, J = 8.3 Hz, C_6H_4), 1.38 (s, 24 H, CH_3). ^{13}C{^{1}H} NMR (100 MHz, $CDCl_3$, r.t.): δ 143.5 (s), 140.2 (s), 140.0 (s), 139.8 (s), 135.5 (s), 127.8 (s), 127.6 (s), 127.6 (s), 126.5 (s), 84.0 (s), 25.0 (s). IR (ATR): ν = 2979, 2931, 1606, 1362, 1267, 1144, 1093, 855, 812, 660 cm^{-1}. HRMS (MALDI-TOF, Dithranol): m/z: calcd for $C_{42}H_{44}B_2O_4$: 634.3434 [M^+]; found: 634.3402.

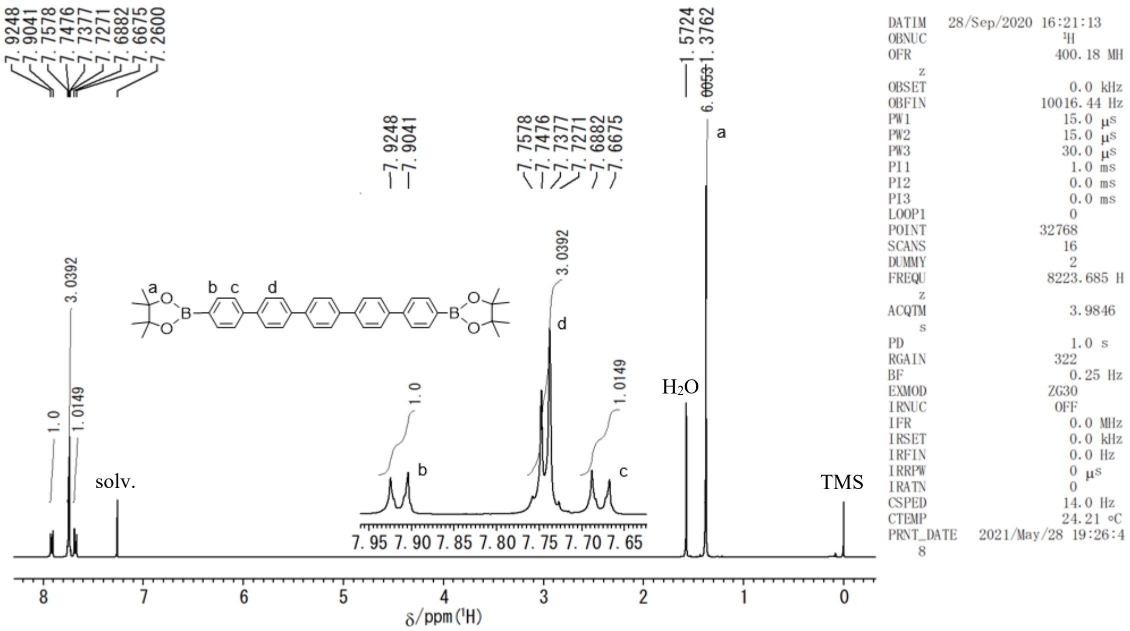

Figure S3. ^{1}H NMR spectrum of **L5** (400 MHz, $CDCl_3$, r.t.).

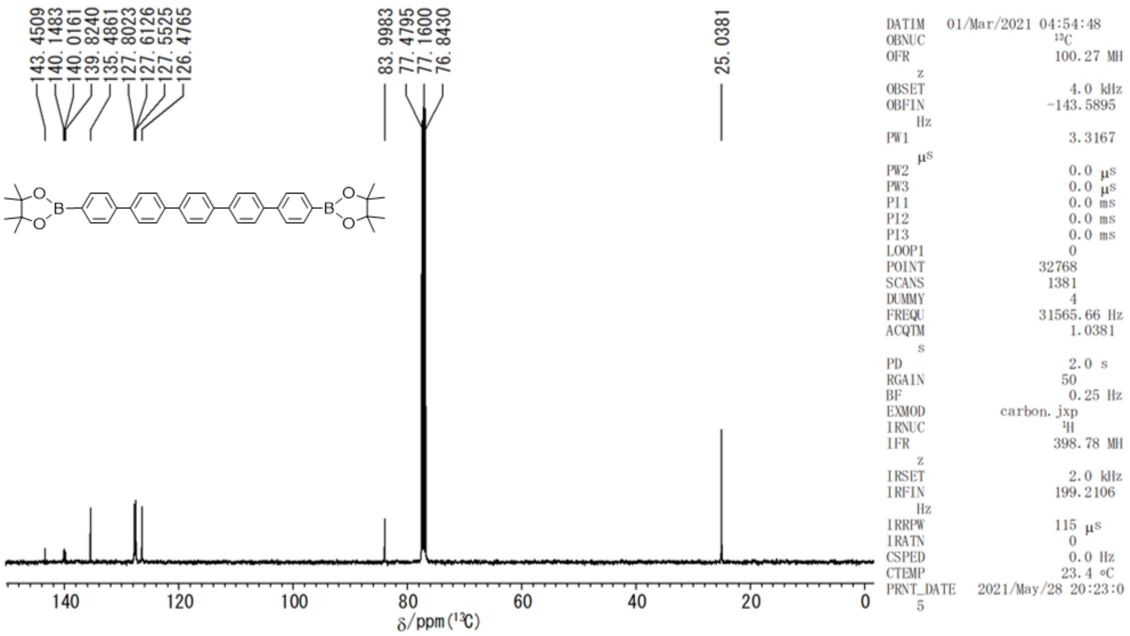

Figure S4. $^{13}C\{^{1}H\}$ NMR spectrum of **L5** (100 MHz, $CDCl_3$, r.t.).

Synthesis of [$Au_2(C_6H_4)_3(Cy_2PCH_2PCy_2)]_3$ (Au-3)

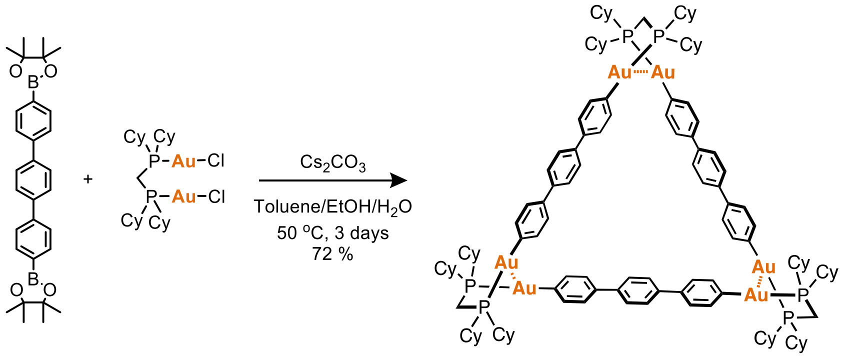

A mixture of 4,4’’-*p*-terphenyldiboronic acid pinacol ester (**L3**) (52.1 mg, 0.11 mmol), Cs_2CO_3 (205 mg, 0.63 mmol) and [Au_2Cl_2(dcpm)] (88.5 mg, 0.10 mmol) in degassed toluene/H_2O/EtOH (4 mL/1 mL/1 mL) was stirred for 3 days at 50 °C under an argon atmosphere. After the reaction mixture allowed to cool to room temperature, the precipitates were collected by suction filtration and washed with EtOH (10 mL), then dried in *vacuo*. The Au complex, [$Au_2(C_6H_4)_3(Cy_2PCH_2PCy_2)]_3$ (**Au-3**), was obtained as

a white solid (73.9 mg, 0.024 mmol, 72%). ^{1}H NMR (399 MHz, $C_2D_2Cl_4$, r.t.): δ 7.65 (s, 12H, C_6H_4), 7.64-7.59 (m, 12 H, C_6H_4), 7.49 (d, 12H, J = 7.8 Hz, C_6H_4) 2.30-2.21 (br, 12H, C_6H_{11}), 2.14-2.07 (br, 18H, C_6H_{11} and CH_2), 1.97-1.91 (br, 24H, C_6H_{11}), 1.79-1.74 (br, 12H, C_6H_{11}), 1.70-1.59 (br, 24H, C_6H_{11}, overlapping with signal of H_2O), 1.57-1.46 (br, 12H, C_6H_{11}), 1.38-1.31 (br, 36 H, C_6H_{11}). $^{13}C\{^{1}H\}$ NMR (100 MHz, $C_2D_2Cl_4$, r.t.): δ 174.0 (t, J = 57.4 Hz), 140.3 (s), 139.9 (s), 136.6 (s), 126.9 (s), 125.2 (s), 35.2 (t, J = 14.9 Hz), 29.8 (s), 29.0 (s), 26.7 (d, J = 20.4 Hz), 25.8 (s). $^{31}P\{^{1}H\}$ NMR (161 MHz, $C_2D_2Cl_4$, r.t.): δ 48.2 (s). IR (ATR): ν = 2922, 2848, 1442, 1355, 1006, 795, 756, 511 cm^{-1}. Anal. Calcd for $C_{129}H_{174}Au_6P_6$: C, 50.10; H, 5.67. Found: C, 50.58; H, 5.77. HRMS (FT-ICR MALDI-TOF, DCTB): m/z: calcd. for $C_{129}H_{174}Au_6P_6$+H: 3093.0141 [M+H]$^+$; found: 3093.0170.

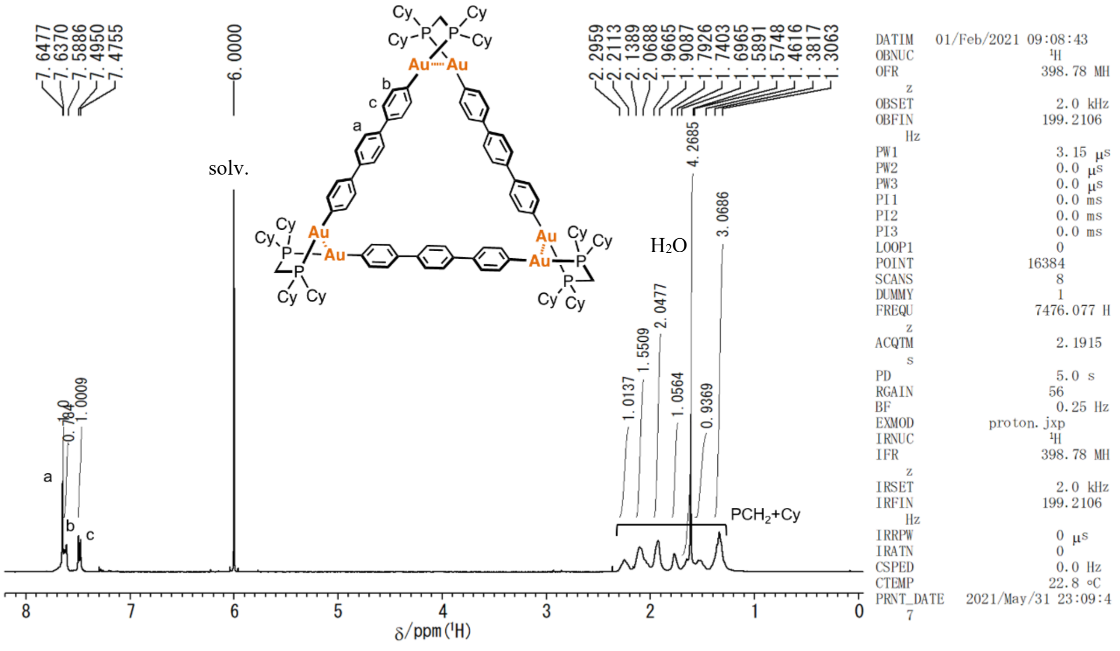

Figure S5. ^{1}H NMR spectrum of **Au-3** (399 MHz, $C_2D_2Cl_4$, r.t.).

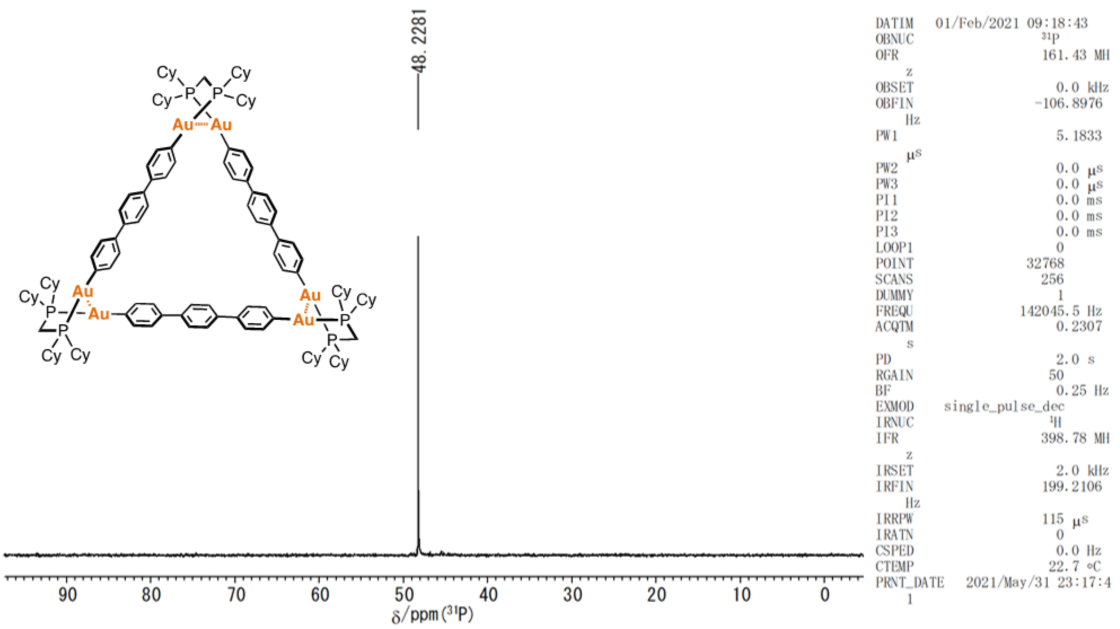

Figure S6. $^{31}P\{^{1}H\}$ NMR spectrum of **Au-3** (161 MHz, $C_2D_2Cl_4$, r.t.).

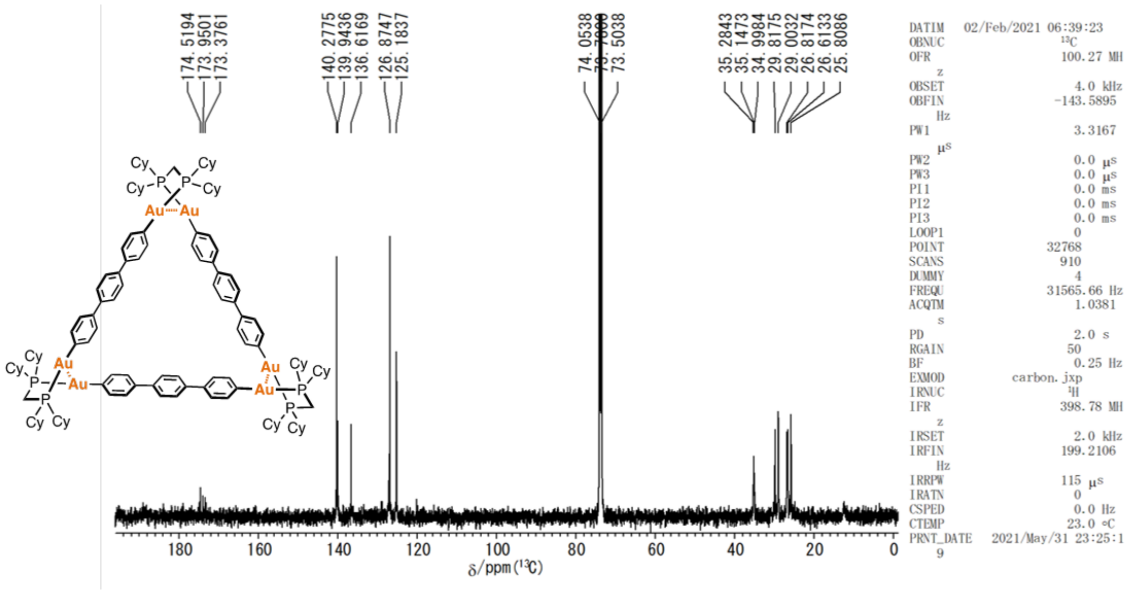

Figure S7. $^{13}C\{^{1}H\}$ NMR spectrum of **Au-3** (100 MHz, $C_2D_2Cl_4$, r.t.).

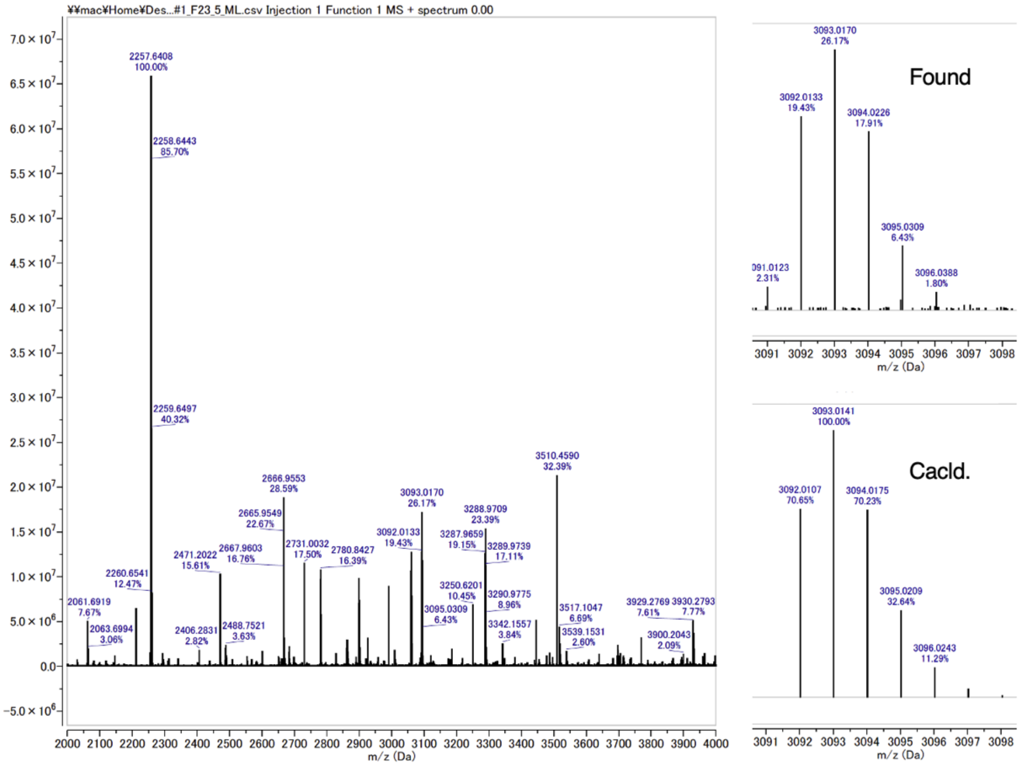

Figure S8. HRMS spectrum of **Au-3** (FT-ICR MALDI-TOF, DCTB).

Transmetalation of [$Au_2Cl_2(Ph_2PCH_2PPh_2)$] (1') with L3

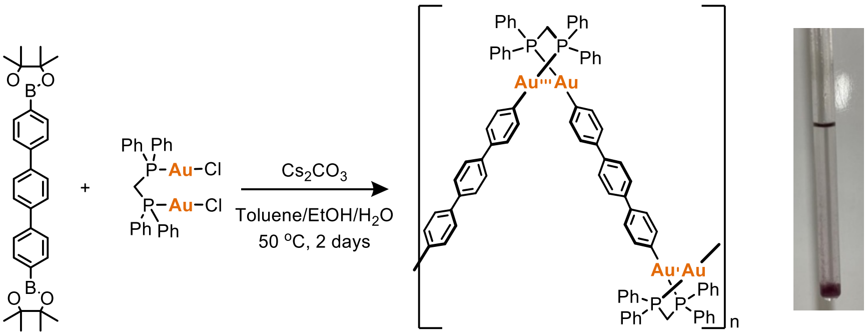

A mixture of 4,4''-*p*-terphenyldiboronic acid pinacol ester (**L3**) (48.2 mg, 0.10 mmol), Cs_2CO_3 (166 mg, 0.51 mmol) and [Au_2Cl_2(dppm)] (**1'**) (85.2 mg, 0.10 mmol) in degassed toluene/H_2O/EtOH (4 mL/1 mL/1 mL) was stirred for 2 days at 50 °C under an argon atmosphere. After the reaction mixture allowed to cool to room temperature, the precipitates were collected by suction filtration and washed with toluene/H_2O/EtOH (10

mL/10 mL/10 mL), then dried in *vacuo*. The resulting purple solid (69.7 mg) was partially soluble in $C_2D_2Cl_4$. The ^{1}H NMR spectrum (Figure S9) showed broad peaks and the ^{31}P{^{1}H} NMR spectrum (Figure S10) showed two singlets at 32.4 and 34.4 ppm with very low intensities, suggesting the formation of linear or undesired macrocyclic oligomers.

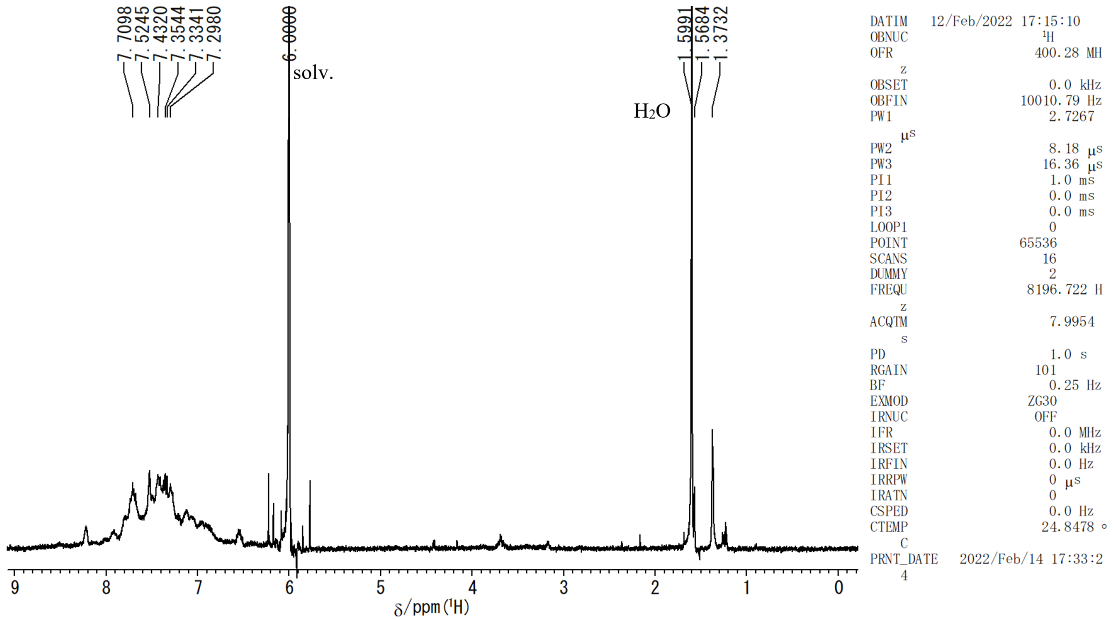

Figure S9. ^{1}H NMR spectrum of the crude product (400 MHz, $C_2D_2Cl_4$, r.t.).

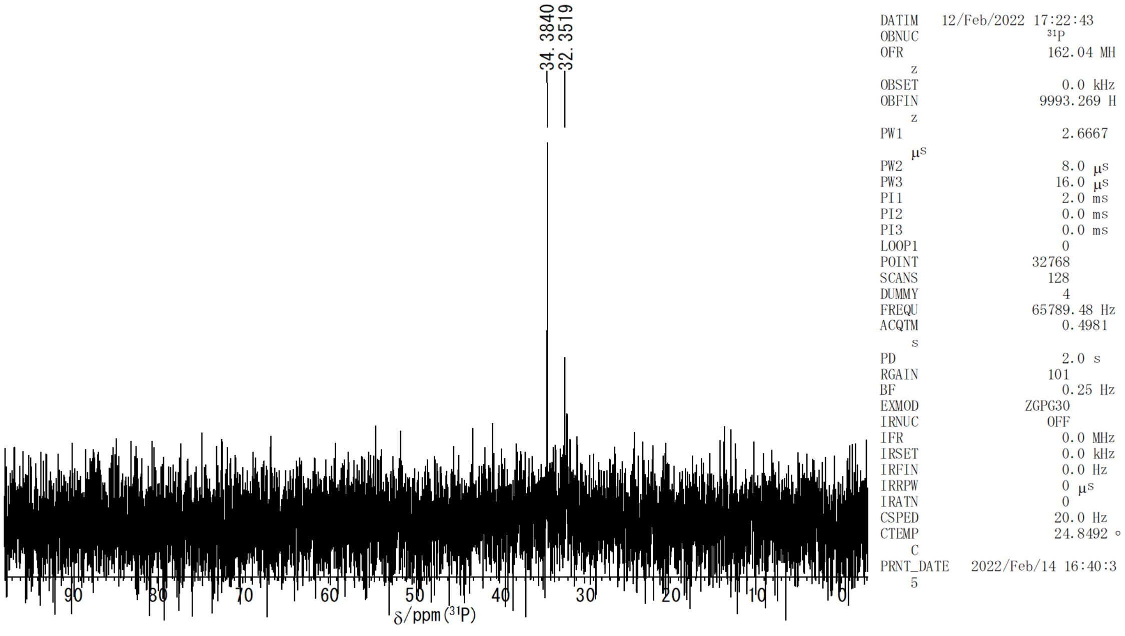

Figure S10. ^{31}P{^{1}H} NMR spectrum of the crude product (162 MHz, $C_2D_2Cl_4$, r.t.).

Synthesis of $[Au_2(C_6H_4)_4(Cy_2PCH_2PCy_2)]_3$ (Au-4)

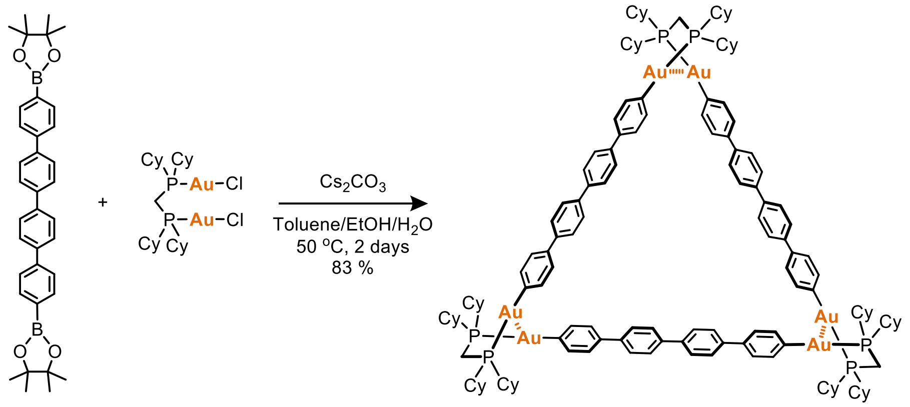

A mixture of 4,4'''-*p*-quaterphenyldiboronic acid pinacol ester (280 mg, 0.50 mmol), Cs_2CO_3 (815 mg, 2.5 mmol) and $[Au_2Cl_2(dcpm)]$ (438 mg, 0.50 mmol) in degassed toluene/H_2O/EtOH (12 mL/3 mL/3 mL) was stirred for 2 days at 50 °C under an argon atmosphere. After the reaction mixture allowed to cool to room temperature, the precipitates were collected by suction filtration and washed with toluene (10 mL), H_2O (10 mL), and EtOH (10 mL), then dried in *vacuo*. The Au complex, $[Au_2(C_6H_4)_4(Cy_2PCH_2PCy_2)]_3$ (**Au-4**), was obtained as a white solid (460 mg, 0.14 mmol, 83%). ^1H NMR (399 MHz, $C_2D_2Cl_4$, r.t.): δ 7.70 (s, 24H, C_6H_4), 7.67-7.61 (m, 12H, C_6H_4), 7.50 (d, 12 H, J = 7.6 Hz, C_6H_4), 2.31-2.20 (br, 12H, C_6H_{11}), 2.15-2.07 (br,18H, C_6H_{11} and CH_2), 1.98-1.90 (br, 24H, C_6H_{11}), 1.82-1.75 (br, 12H, C_6H_{11}), 1.71-1.60 (br, 24H, C_6H_{11}, overlapping with signal of H_2O), 1.59-1.46 (br, 12H, C_6H_{11}) 1.40-1.30 (br, 36H, C_6H_{11}). $^{13}C\{^1H\}$ NMR (100 MHz, $C_2D_2Cl_4$, r.t.): δ 174.3 (t, J = 57.7 Hz), 140.7 (s), 140.3 (s), 138.5 (s), 136.4 (s), 127.1 (s), 127.0 (s), 125.2 (s), 35.2 (t, J = 13.7 Hz), 29.8 (s), 29.0 (s), 26.7 (d, J = 21.4 Hz), 25.8 (s). $^{31}P\{^1H\}$ NMR (161 MHz, $C_2D_2Cl_4$, r.t.): δ 48.3 (s). IR (ATR): v = 2924, 2849, 1447, 1218, 1160, 1004, 803, 760, 513 cm^{-1}. Anal. Calcd for $C_{147}H_{186}Au_6P_6$ $6H_2O$: C, 51.39; H, 6.01. Found: C, 51.48; H, 6.13. HRMS (FT-ICR

MALDI-TOF, DCTB): m/z: calcd for $C_{147}H_{186}Au_6P_6$+H: 3321.1080 [*M*+H]$^+$; found: 3321.1061.

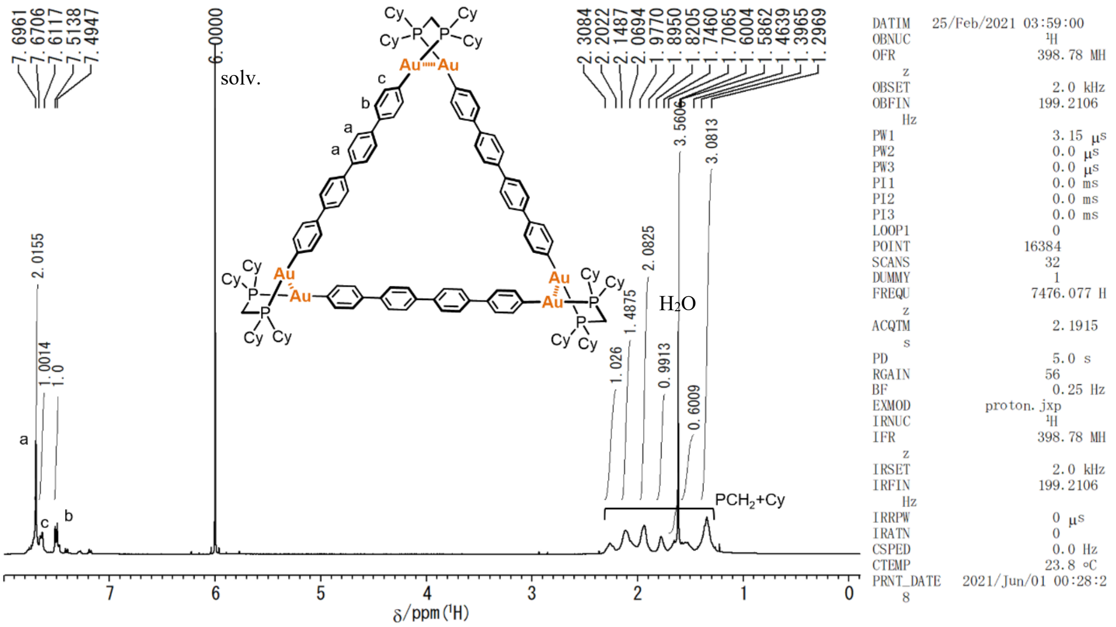

Figure S11. ^{1}H NMR spectrum of **Au-4** (399 MHz, $C_2D_2Cl_4$, r.t.).

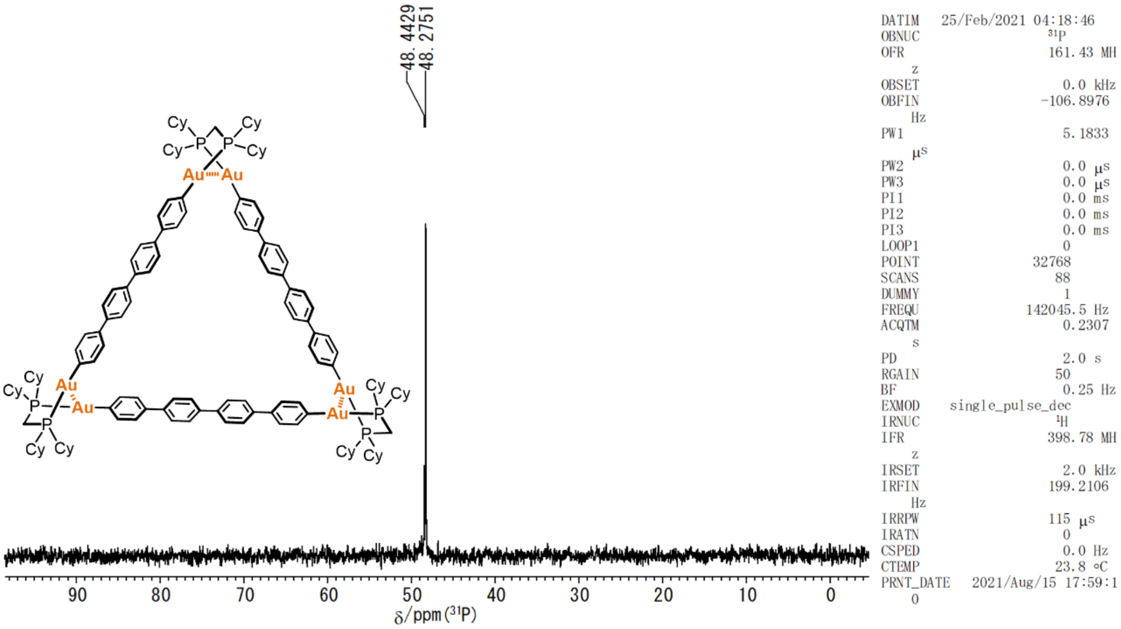

Figure S12. ^{31}P{^{1}H} NMR spectrum of **Au-4** (161 MHz, $C_2D_2Cl_4$, r.t.).

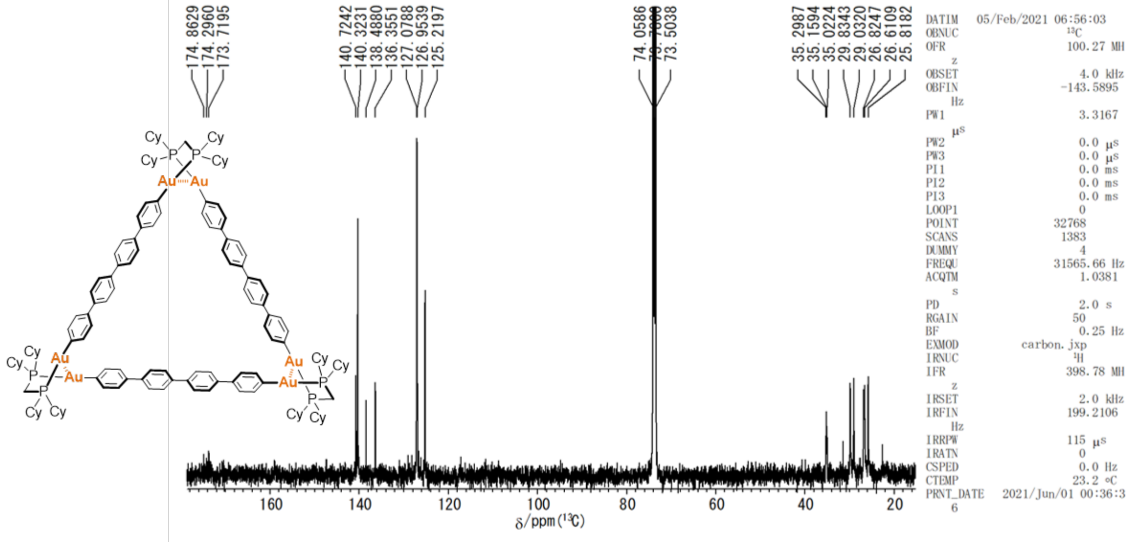

Figure S13. $^{13}C\{^{1}H\}$ NMR spectrum of **Au-4** (100 MHz, $C_2D_2Cl_4$, r.t.).

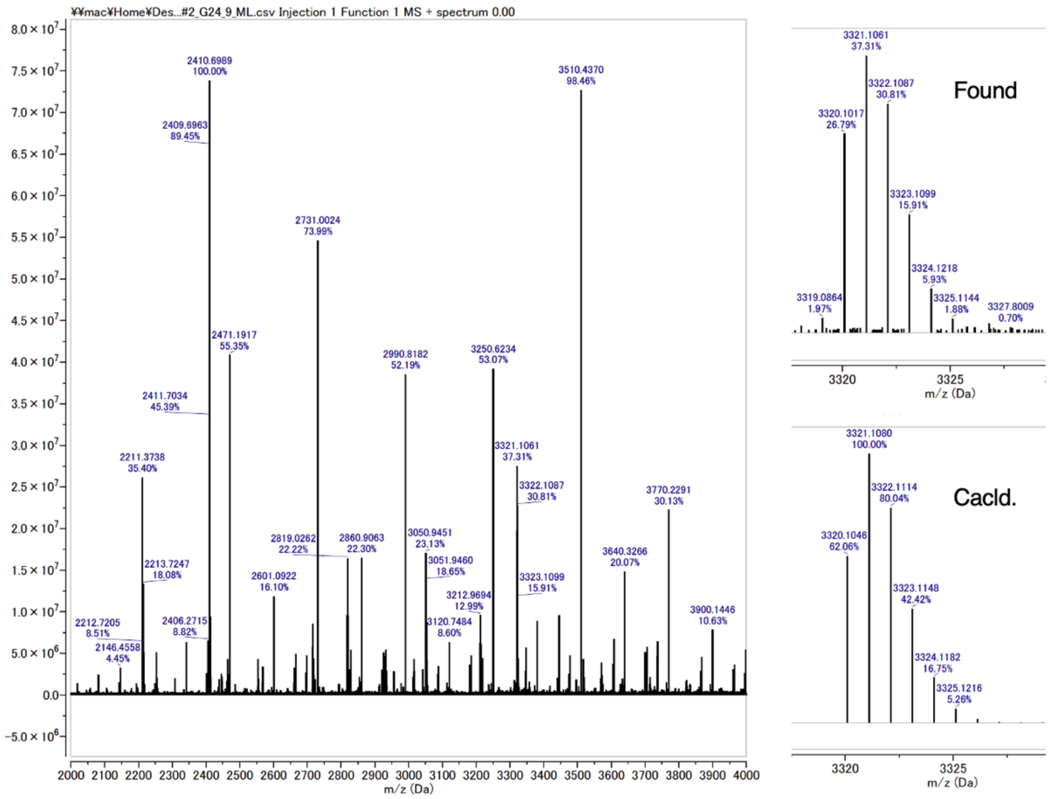

Figure S14. HRMS spectrum of **Au-4** (FT-ICR MALDI-TOF, DCTB).

Synthesis of $[Au_2(C_6H_4)_5(Cy_2PCH_2PCy_2)]_3$ (Au-5)

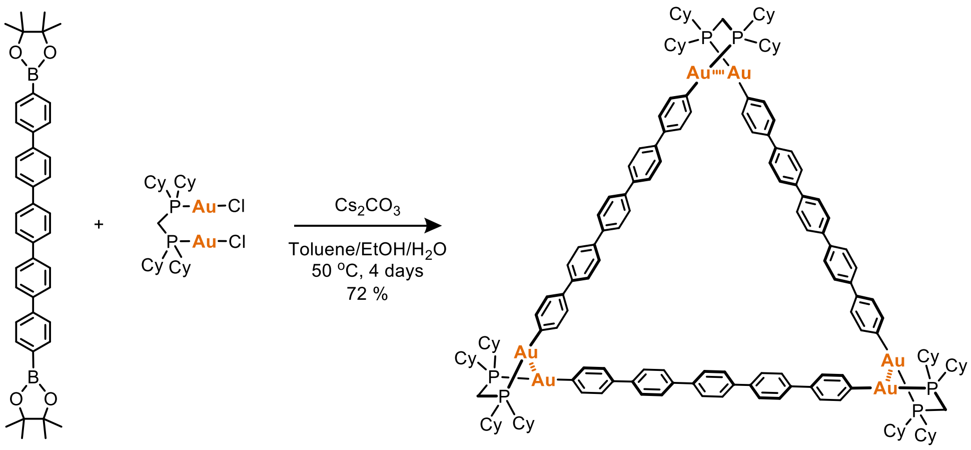

A mixture of 4,4''''-*p*-quinquephenyldiboronic acid pinacol ester (**L5**) (33.2 mg, 0.050 mmol), Cs_2CO_3 (101 mg, 0.31 mmol) and $[Au_2Cl_2(dcpm)]$ (44.7 mg, 0.051 mmol) in degassed toluene/H_2O/EtOH (2 mL/0.5 mL/0.5 mL) was stirred for 4 days at 50 °C under an argon atmosphere. After the reaction mixture allowed to cool to room temperature, the precipitate was collected by suction filtration and washed with H_2O (5 mL), EtOH (10 mL), then dried in *vacuo*. The Au complex, $[Au_2(C_6H_4)_5(Cy_2PCH_2PCy_2)]_3$ (**Au-5**), was obtained as a white solid (43.0 mg, 0.012 mmol, 72%). ^{1}H NMR (399 MHz, $C_2D_2Cl_4$, r.t.): δ 7.76-7.69 (br, 36H, C_6H_4), 7.67-7.59 (br, 12H, C_6H_4), 7.55-7.48 (br, 12 H, C_6H_4), 2.21-2.02 (br, 30H, C_6H_{11}, CH_2), 1.98-1.91 (br, 24H, C_6H_{11}), 1.81-1.75 (br, 12H, C_6H_{11}), 1.68-1.56 (br, 24H, C_6H_{11}, overlapping with signal of H_2O), 1.40-1.29 (br, 48H, C_6H_{11}). ^{31}P{^{1}H} NMR (161 MHz, $C_2D_2Cl_4$, r.t.): δ 48.3 (s). IR (ATR): ν = 2924, 2850, 1480, 1446, 1006, 801, 756, 512 cm^{-1}. Anal. Calcd for $C_{165}H_{198}Au_6P_6$ $3H_2O$: C, 55.00; H, 5.71. Found: C, 55.12; H, 6.04. HRMS (FT-ICR MALDI-TOF, DCTB): m/z: calcd for $C_{165}H_{198}Au_6P_6$+H: 3549.2019 [M+H]$^+$; found: 3549.2084.

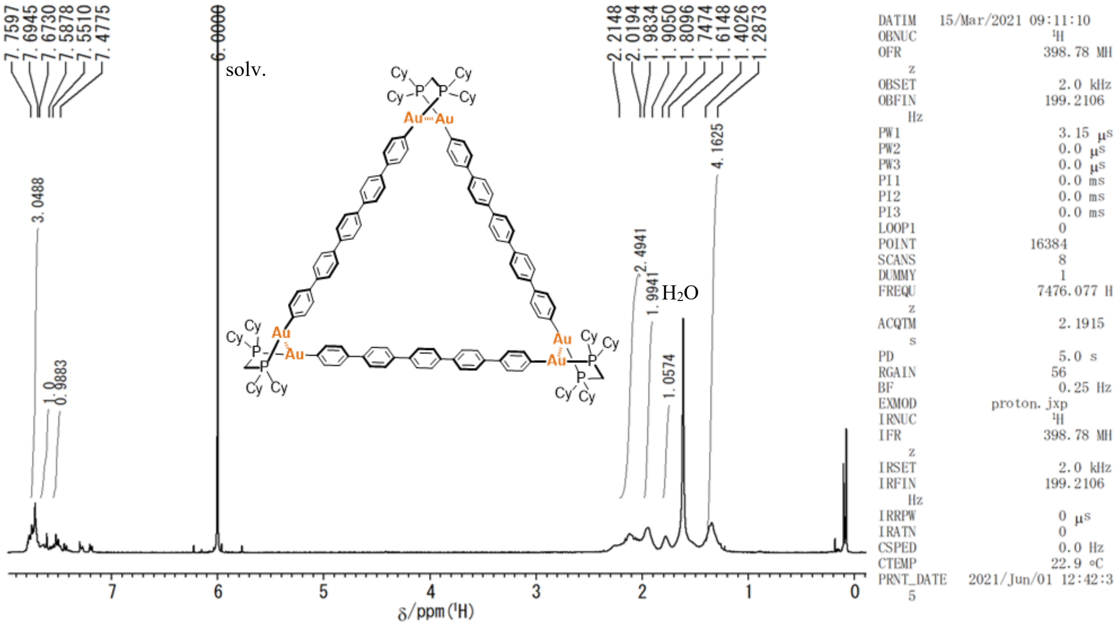

Figure S15. ^{1}H NMR spectrum of **Au-5** (dynamic mixture of C_2- and D_3-isomers) (399 MHz, $C_2D_2Cl_4$, r.t.).

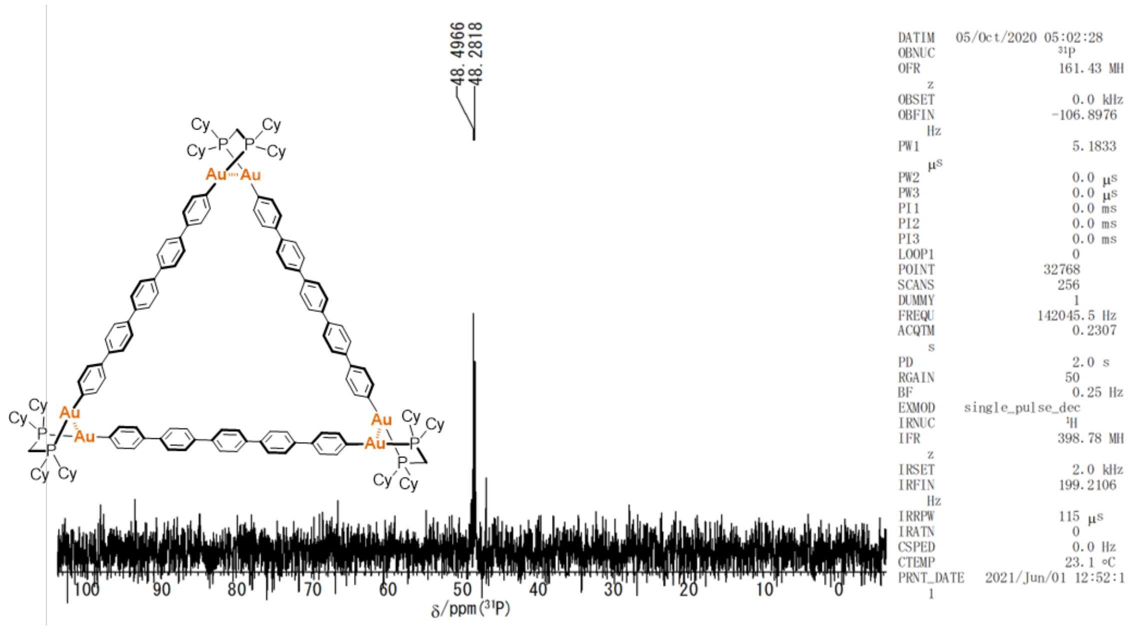

Figure S16. $^{31}P\{^{1}H\}$ NMR spectrum of **Au-5** (dynamic mixture of C_2- and D_3-isomers) (161 MHz, $C_2D_2Cl_4$, r.t.).

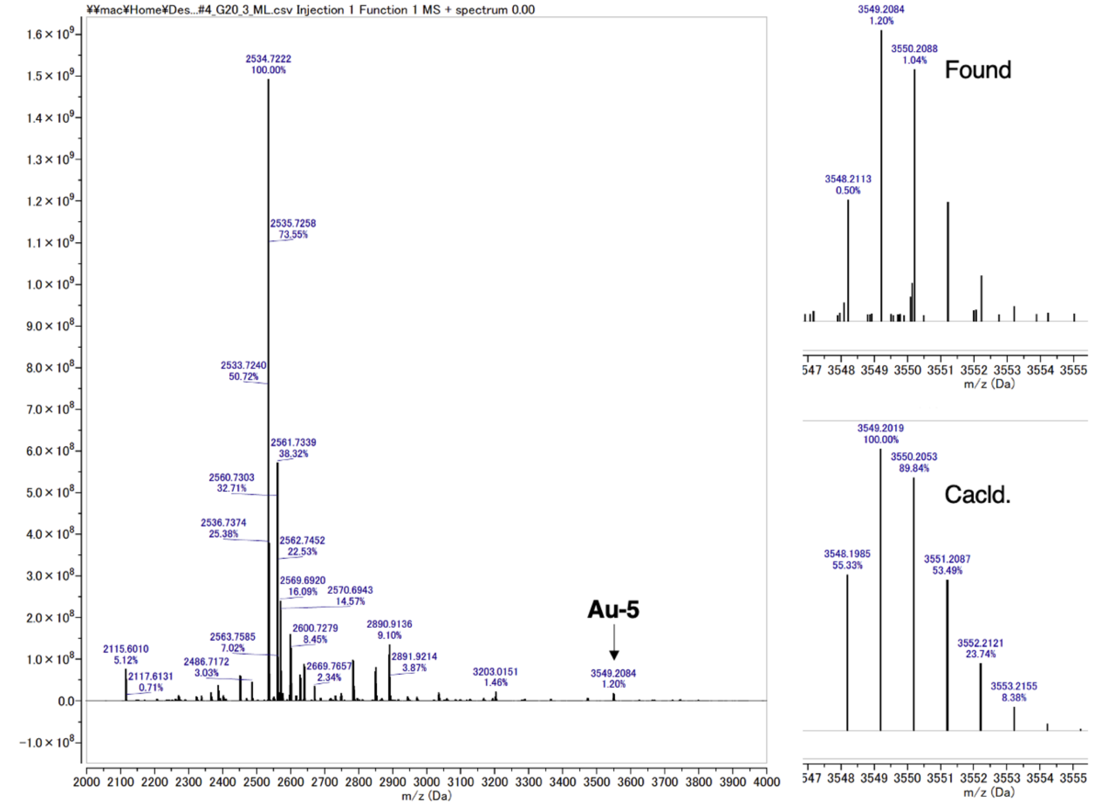

Figure S17. HRMS spectrum of **Au-5** (FT-ICR MALDI-TOF, DCTB).

The $^{31}P\{^{1}H\}$ NMR spectrum of **Au-3** showed one singlet signal at 48.2 ppm as same as **Au-2** in our previous study,[S15] while **Au-4** and **Au-5** appeared two singlet ^{31}P signals at 48.2 and 48.5 ppm ($C_2D_2Cl_4$, 25 °C) (Figure S18a). Based on the crystallographic results (See Figure S75), these signals would be assigned as two stereoisomers (C_2 and D_3, Figure S18b) of triangular Au complexes exchanged slower than the NMR time scale. By comparing the two stereoisomers from polymorphic crystals of **Au-4**, the D_3-isomers has a shorter Au-Au distance than the C_2-isomers, suggesting that the D_3-isomers is more thermodynamically stable. However, D_3-isomers adopt a bent conformation of the phenylene linkers, which seems to be difficult to form it in the case of short oligophenylene linkers. These isomers would be distinguished by observing the ^{31}P NMR spectra of Au complex with longer phenylene linker, **Au-4** and **Au-5**.

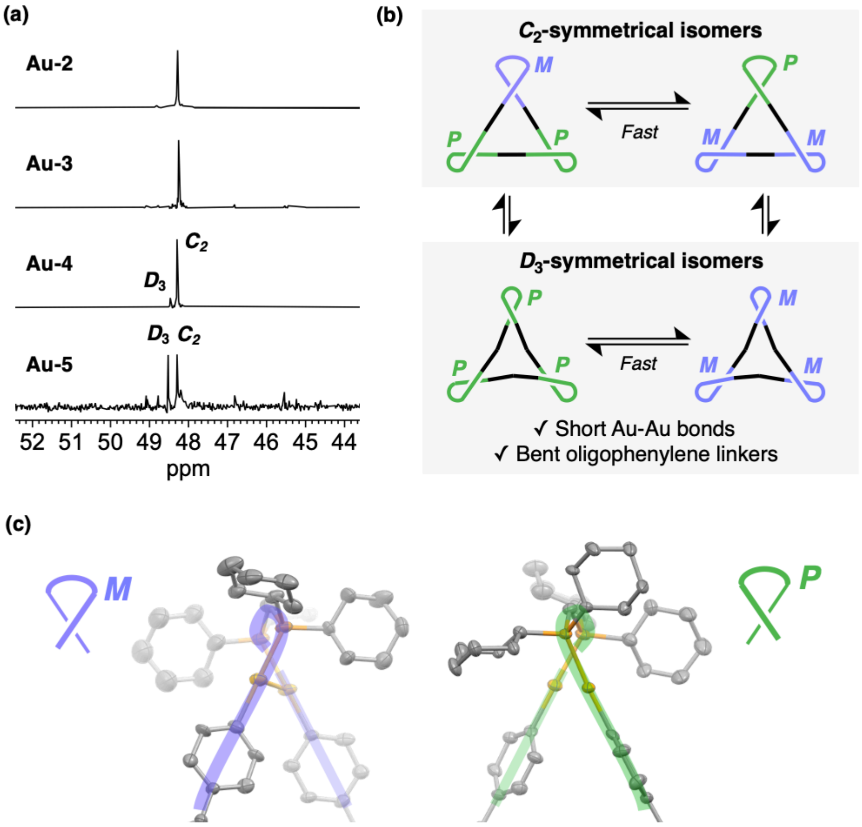

Figure S18. (a) Stacked $^{31}P\{^1H\}$ NMR spectra of macrocyclic Au complexes (161 MHz, $C_2D_2Cl_4$, r.t.). (b) Illustration of possible four stereoisomers of Au complexes. (c) Illustration of axial chirality (*P* and *M* helicity) in Au_2P_2C units at the corner of the complex.

It is noted that the oxidative chlorination of these gold complexes yielded corresponding [*n*]CPPs with the high selectivity, and any aromatic by-products were observed in the ^1H NMR spectra of the crude products (Figure S19). These results also supported that the two singlet ^{31}P signals would be assigned stereoisomers of triangular macrocyclic Au complexes, not macrocyclic Au complexes with larger numbers of metal centers and ligands and oligomer complexes with non-cyclic structures.

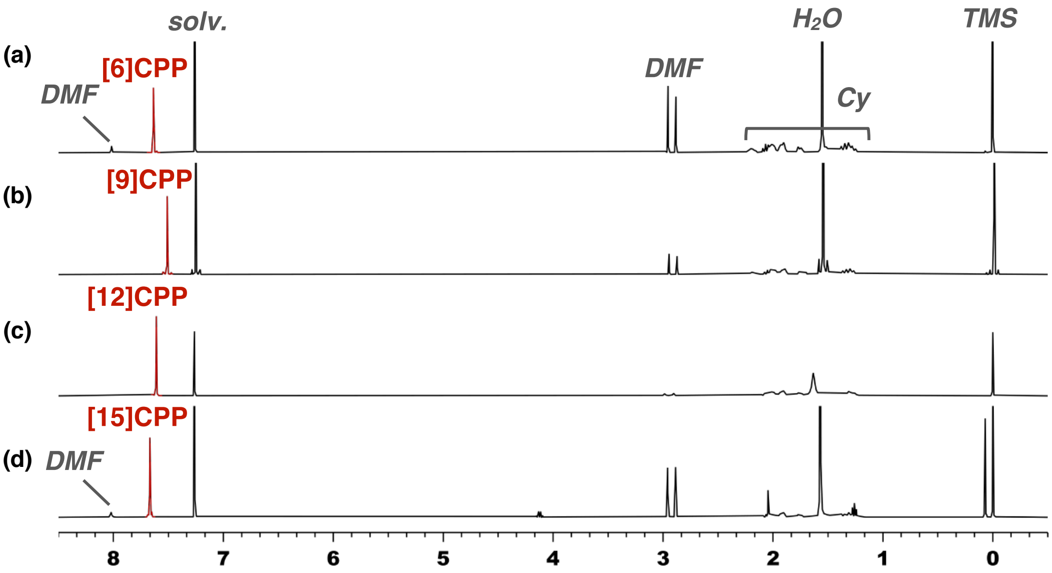

Figure S19. Stacked ^{1}H NMR spectra of crude product after oxidative chlorination of macrocyclic Au complexes (400 MHz, $CDCl_3$, r.t.).

Oxidation of $[Au_2(C_6H_4)_3(Cy_2PCH_2PCy_2)]_3$ (Au-3) by $PhICl_2$.

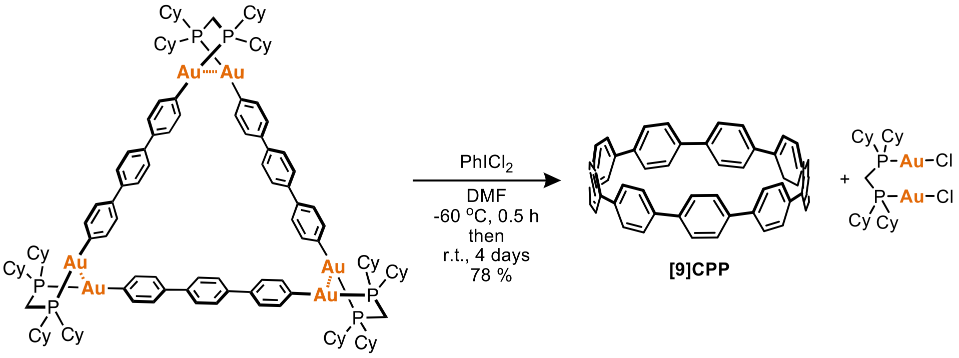

To a suspension of **Au-3** (16.2 mg, 5.2 μmol) in degassed DMF (5 mL) was added $PhICl_2$ (6.0 mmol/L in DMF, 2.5 mL, 15 μmol) dropwise with stirring at –60 ºC for 5 min under an argon atmosphere. The reaction mixture was stirred at the same temperature for 30 min, then it was allowed to warm to 25 ºC and stirred for 4 days. Solvent and iodobenzene (by-product) were removed under vacuum. The crude product was purified by silica gel column chromatography (eluent; $CHCl_3$) to give [9]cycloparaphenylene

([9]CPP) as an yellow solid (R_f = 0.77, 2.80 mg, 4.1 μmol, 78%), and [Au_2Cl_2(dcpm)] as a white solid (R_f = 0.13, 10.7 mg, 12 μmol, 78%). Data for [9]CPP[S8]; ^{1}H NMR (400 MHz, $CDCl_3$, r.t.): *δ* 7.52 (s, 36H, C_6H_4). $^{13}C\{^1H\}$ NMR (100 MHz, $CDCl_3$, r.t.): δ 138.1 (s), 127.5 (s).

Note: Because the laboratory time was limited by the infection of COVID-19, a workup of the reaction was performed after 4 days. The formation of [9]CPP would have been completed in a few hours, as the fluorescence from the product in the reaction mixture was observed.

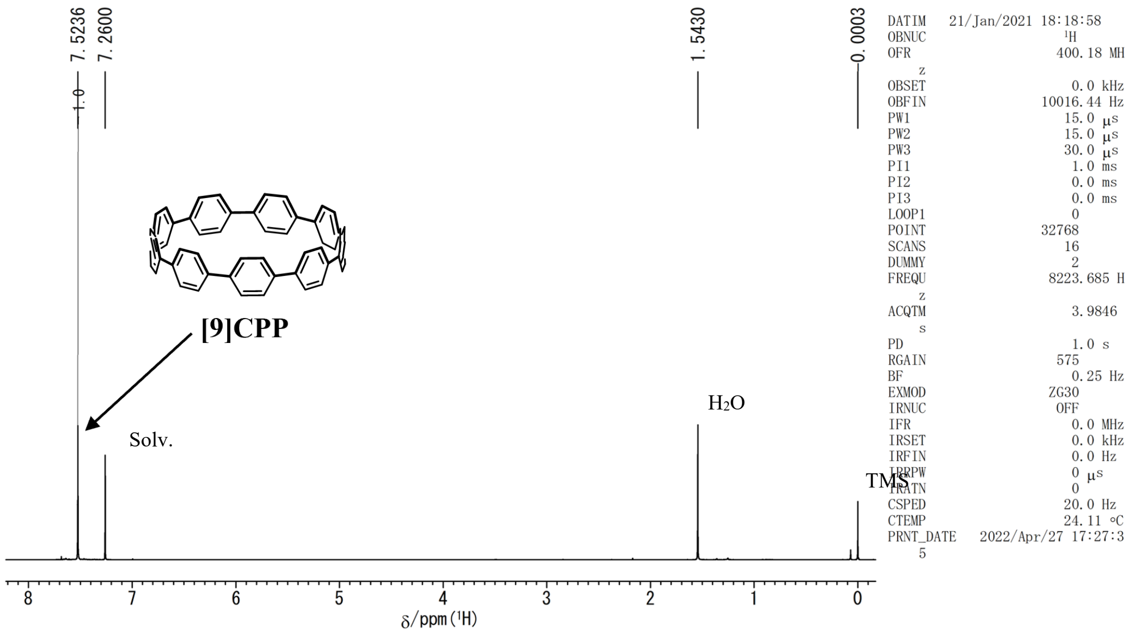

Figure S20. ^{1}H NMR spectrum of isolated [9]CPP (400 MHz, $CDCl_3$, r.t.).

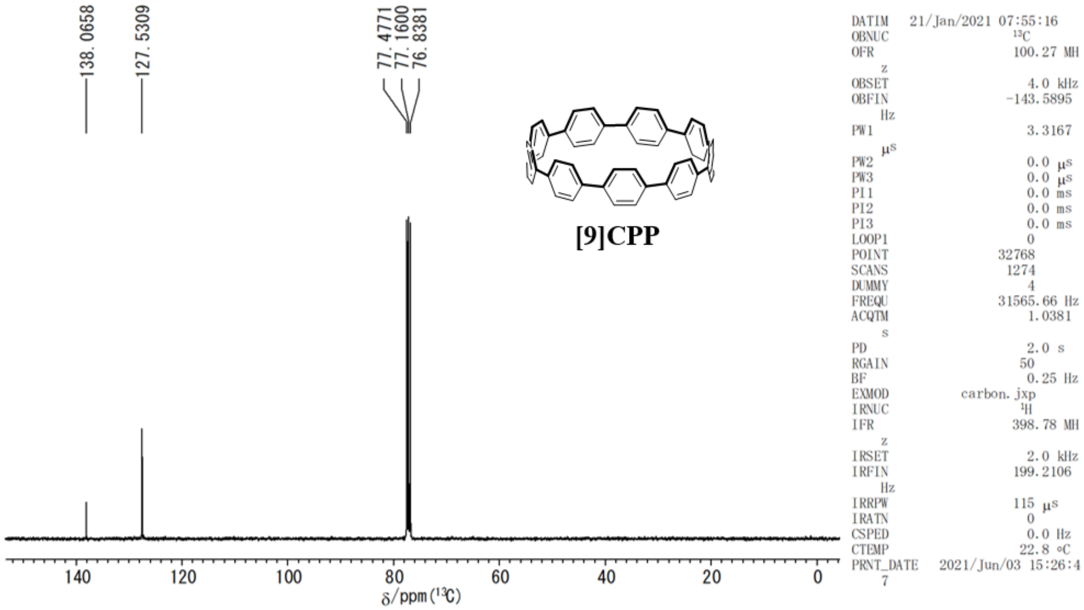

Figure S21. $^{13}C\{^{1}H\}$ NMR spectrum of isolated [9]CPP (100 MHz, $CDCl_3$, r.t.).

Oxidation of $[Au_2(C_6H_4)_4(Cy_2PCH_2PCy_2)]_3$ (Au-4) by $PhICl_2$.

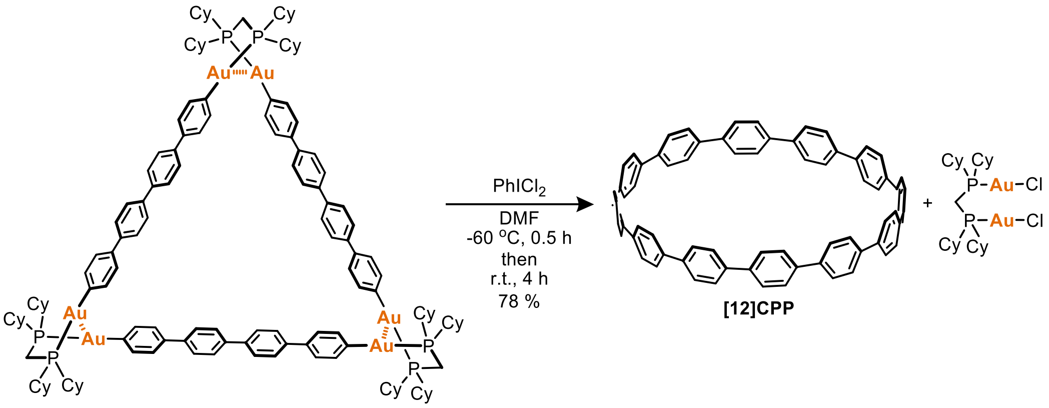

To a suspension of **Au-4** (50.0 mg, 15 μmol) in degassed DMF (20 mL) was added $PhICl_2$ (10 mmol/L in DMF, 10 mL, 0.10 mmol) dropwise with stirring at –60 ºC for 5 min under an argon atmosphere. The reaction mixture was stirred at the same temperature for 30 min, then it was allowed to warm to 25 ºC and stirred for 4 h. Solvent and iodobenzene (by-product) were removed under vacuum. The crude product was purified by silica gel column chromatography (eluent; $CHCl_3$) to give [12]cycloparaphenylene

([12]CPP) as an pale-yellow solid (R_f = 0.87, 10.8 mg, 12 μmol, 78%), and [Au_2Cl_2(dcpm)] as a white solid (R_f = 0.19, 23.6 mg, 27 μmol, 60%). Data for [12]CPP[S8]; ^{1}H NMR (400 MHz, $CDCl_3$, r.t.): δ 7.61 (s, 48H, C_6H_4). $^{13}C\{^1H\}$ NMR (100 MHz, $CDCl_3$, r.t.): δ 138.7 (s), 127.5 (s).

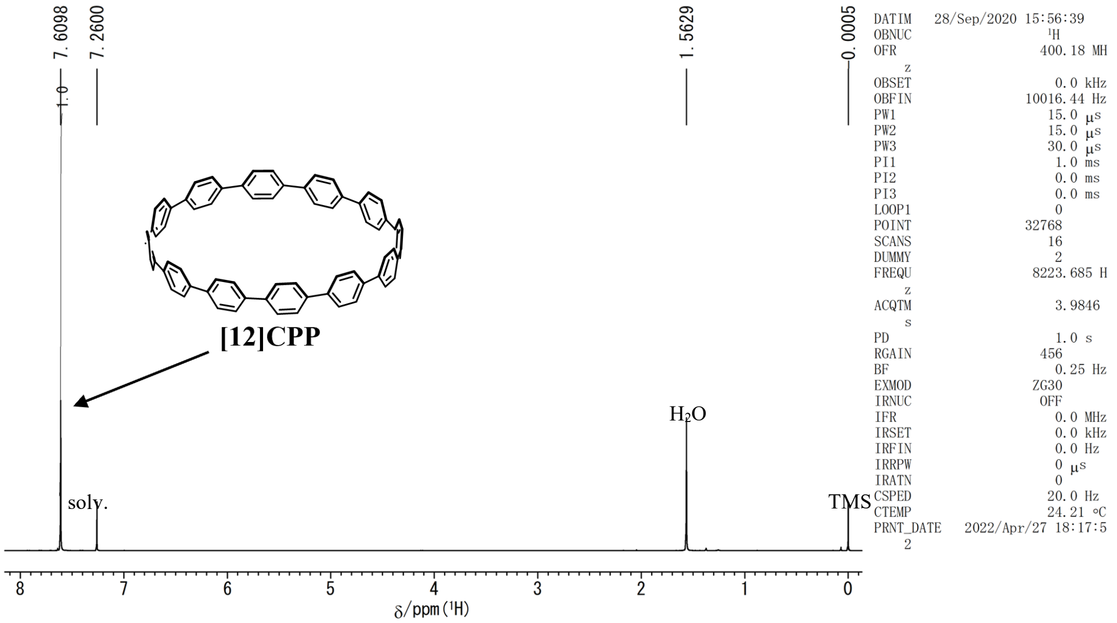

Figure S22. ^{1}H NMR spectrum of isolated [12]CPP (400 MHz, $CDCl_3$, r.t.).

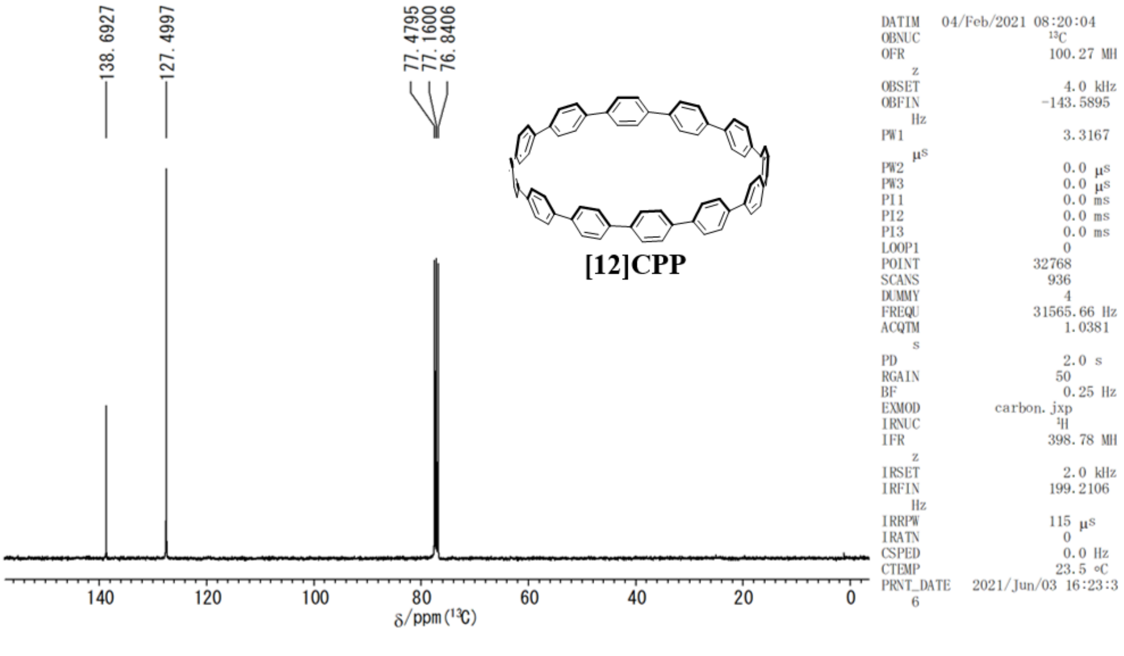

Figure S23. $^{13}C\{^1H\}$ NMR spectrum of isolated [12]CPP (100 MHz, $CDCl_3$, r.t.).

Oxidation of $[Au_2(C_6H_4)_5(Cy_2PCH_2PCy_2)]_3$ (Au-5) by $PhICl_2$.

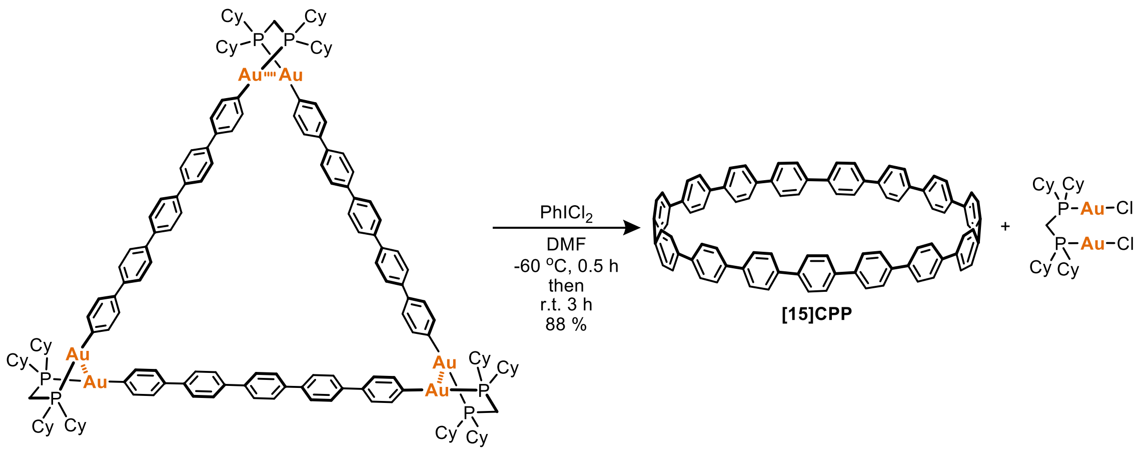

To a suspension of **Au-5** (18.2 mg, 5.1 μmol) in degassed DMF (5 mL) was added $PhICl_2$ (6.0 mmol/L in DMF, 2.5 mL, 15 μmol) dropwise with stirring at –60 ºC for 5 min under an argon atmosphere. The reaction mixture was stirred at the same temperature for 30 min, then it was allowed to warm to 25 ºC and stirred for 4 days. Solvent and iodobenzene (by-product) were removed under vacuum. The crude product was purified by silica gel column chromatography (eluent; $CHCl_3$) to give [15]cycloparaphenylene ([15]CPP) as an white solid (R_f = 0.79, 5.10 mg, 4.5 μmol, 88%). Data for [15]CPP[S14]; ^{1}H NMR (400 MHz, $CDCl_3$, r.t.): δ 7.67 (s, 60H, C_6H_4). $^{13}C\{^1H\}$ NMR (100 MHz, $CDCl_3$, r.t.): δ 139.0 (s), 127.5 (s).

Note: Because the laboratory time was limited by the infection of COVID-19, a workup of the reaction was performed after 4 days. The formation of [15]CPP would have been completed in a few hours, as the fluorescence from the product in the reaction mixture was observed.

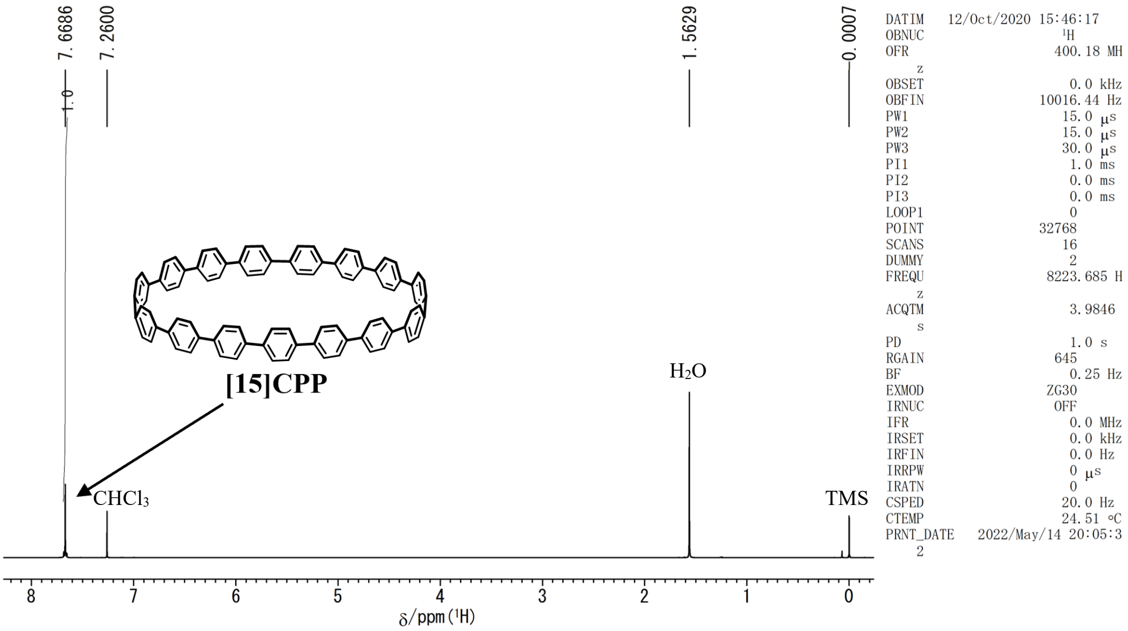

Figure S24. ^{1}H NMR spectrum of isolated [15]CPP (400 MHz, $CDCl_3$, r.t.).

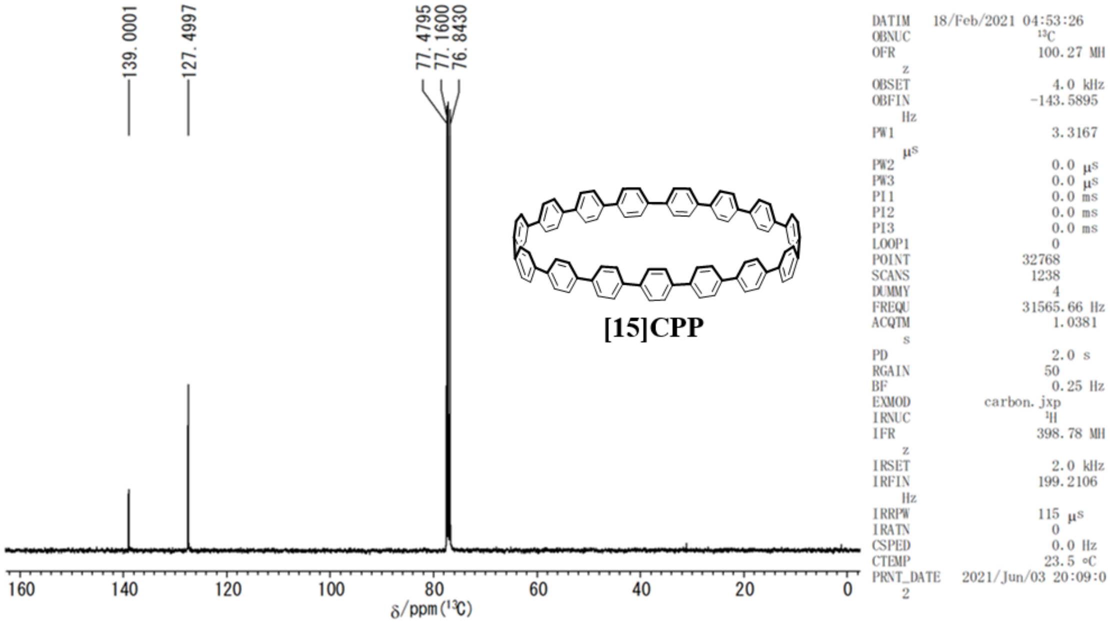

Figure S25. $^{13}C\{^{1}H\}$ NMR spectrum of isolated [15]CPP (100 MHz, $CDCl_3$, r.t.).

Table S1. List of reported synthesis and product yield for [9]CPP

Author	Ref.	Year	Random/ Selective	Number of steps and total yield from commercial reagents
Jasti	S8	2008	Random	5 steps / 0.24%
Jasti	S9	2012	Selective	11 steps / 0.81%
Itami	S10	2011	Random	4 steps / 6.2%
Itami	S11	2012	Selective	6 steps / 5.5%
Yamago	S12	2011	Random	5 steps / 0.9%
**This work**		**2022**	**Selective**	**3 steps / 48%**

Table S2. List of reported synthesis and product yield for [12]CPP

Author	Ref.	Year	Random/ Selective	Number of steps and total yield from commercial reagents
Jasti	S8	2008	Random	5 steps / 1.5%
Jasti	S9	2012	Selective	10 steps / 1.5%
Itami	S13	2009	Selective	6 steps / 9.8%
Itami	S10	2011	Random	4 steps / 12%
Yamago	S12	2011	Selective	4 steps / 3.1%
**This work**		**2022**	**Selective**	**4 steps / 62%**

Table S3. List of reported synthesis and product yield for [15]CPP

Author	Ref.	Year	Random/ Selective	Number of steps and total yield from commercial reagents
Itami	S14	2010	Selective	7 steps / 5.7%
**This work**		**2022**	**Selective**	**5 steps / 29%**

Synthesis of [$Au_2(C_6H_4$-4-F$)_2$(dcpm)] (Au$_C$-FF).

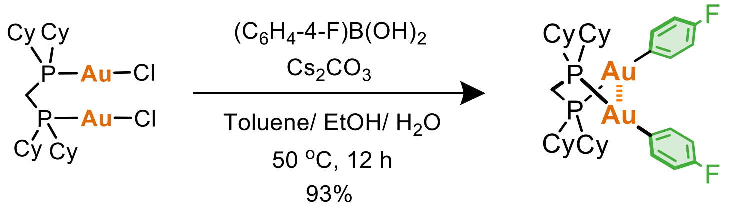

The acyclic Au complex, **Au$_C$-FF**, was synthesized according to the literature[S15] with a slight modification. In a screw capped tube, a mixture of 4-fluorophenylboronic acid (17 mg, 0.12 mmol), Cs_2CO_3 (115 mg, 0.353 mmol) and [Au_2Cl_2(dcpm)] (50 mg, 0.057 mmol) in degassed toluene/ H_2O/ EtOH (2 mL/ 0.5 mL/ 0.5 mL) was stirred for 12 hours at 50 °C under an argon atmosphere. After a mixture was allowed to cool to room temperature, the solvent was evaporated. The residue was dissolved into CH_2Cl_2 (5 mL), and the organic layer was washed with water (5 mL) for 2 times and brine. The organic layer was dried over Na_2SO_4, and filtered off. The filtrate was concentrated to *ca.* 0.5 mL, then poured onto Et_2O (50 mL). The precipitate was collected by suction filtration, then dried in *vacuo*. The desired Au complex **Au$_C$-FF** was obtained as a white solid in 93% yield (53 mg, 0.053 mmol). ^{1}H NMR (400 MHz, $CDCl_3$, r.t.): δ 7.40 (t, 4H, J = 7.6 Hz, AuC_6H_4-4-F), 6.86 (t, 4H, J = 9.1 Hz, AuC_6H_4-4-F), 2.30-2.12 (m, 4H, PCy), 2.12-1.95 (m, 8H, PCy), 2.00 (t, 2H, J = 9.0 Hz, PCH_2P), 1.94-1.80 (m, 8H, P*Cy*), 1.78-1.65 (m, 4H, P*Cy*), 1.65-1.40 (m, 8H, P*Cy*), 1.38-1.15 (m, 12H, P*Cy*); ^{19}F NMR (376 MHz, $CDCl_3$, r.t.): δ -117.6; ^{31}P{^{1}H} NMR (162 MHz, $CDCl_3$, r.t.): δ 47.9; ^{13}C{^{1}H} NMR (100 MHz, $CDCl_3$, r.t.): δ 161.4 (d, J = 242 Hz), 140.6 (d, J = 5.3 Hz), 128.7 (d, J = 81 Hz), 113.7 (dt, J = 17, 3.3 Hz), 35.4 (t, J = 14 Hz), 30.1, 29.2, 26.9 (dt, J = 16, 6.0), 26.0 ; IR (ATR): ν = 2921, 2846, 1569, 1478, 1445, 1369, 1343, 1294, 1270, 1202, 1174, 1155, 1075, 1040, 1016, 1002, 917, 888, 852, 804, 770, 747, 726, 704, 637, 571 cm^{-1}.

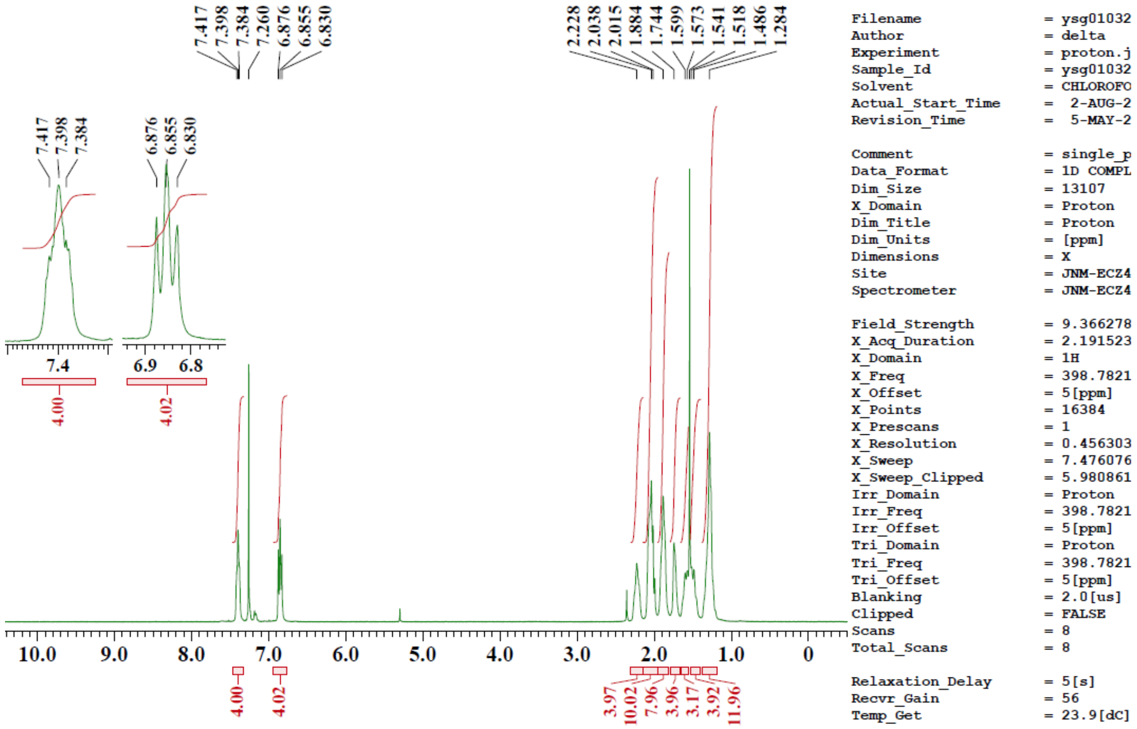

Figure S26. ^{1}H NMR spectrum of **Au$_{C}$-FF** (400 MHz, $CDCl_3$, r.t.).

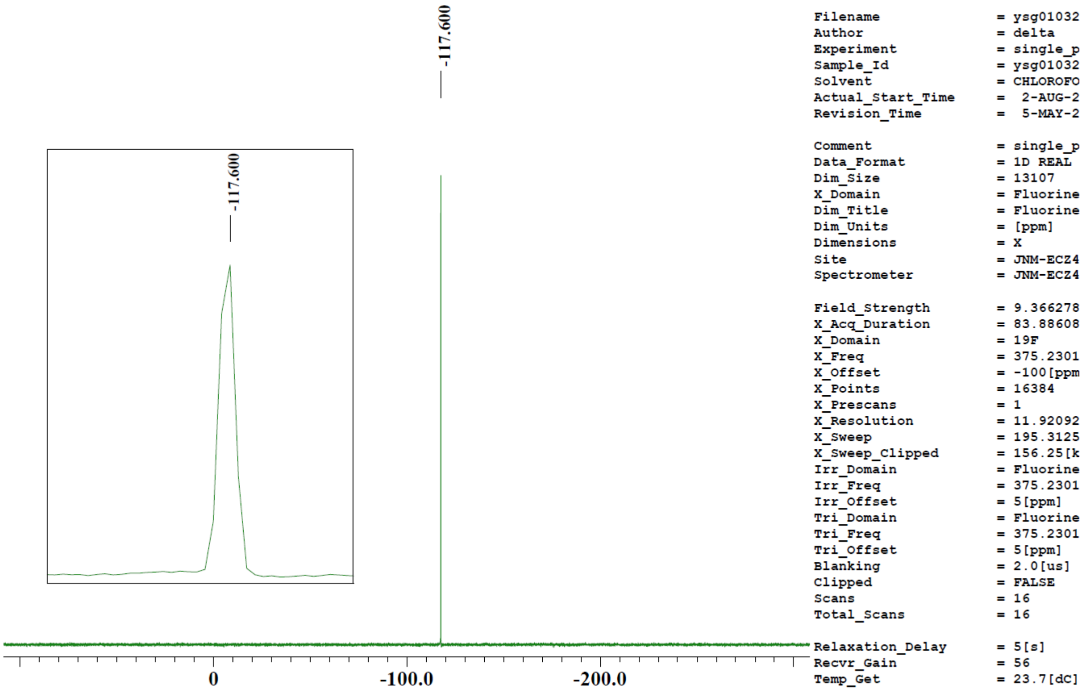

Figure S27. ^{19}F NMR spectrum of **Au$_{C}$-FF** (375 MHz, $CDCl_3$, r.t.).

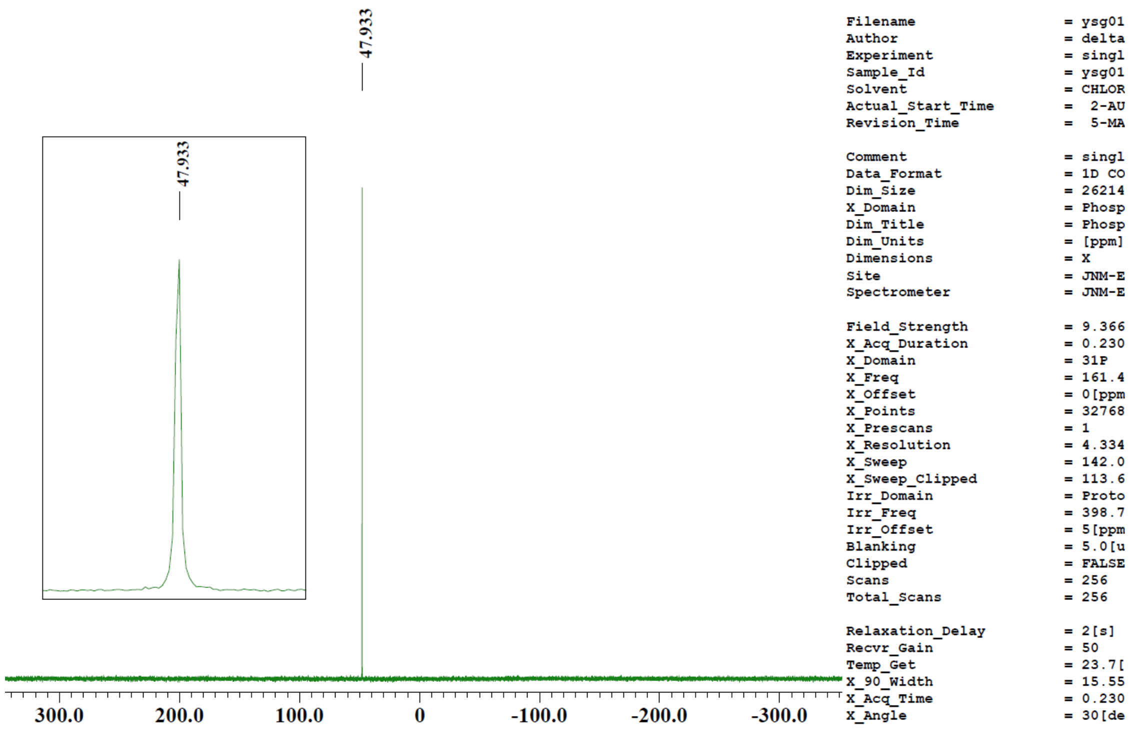

Figure S28. $^{31}P\{^1H\}$ NMR spectrum of **Au$_C$-FF** (161 MHz, $CDCl_3$, r.t.).

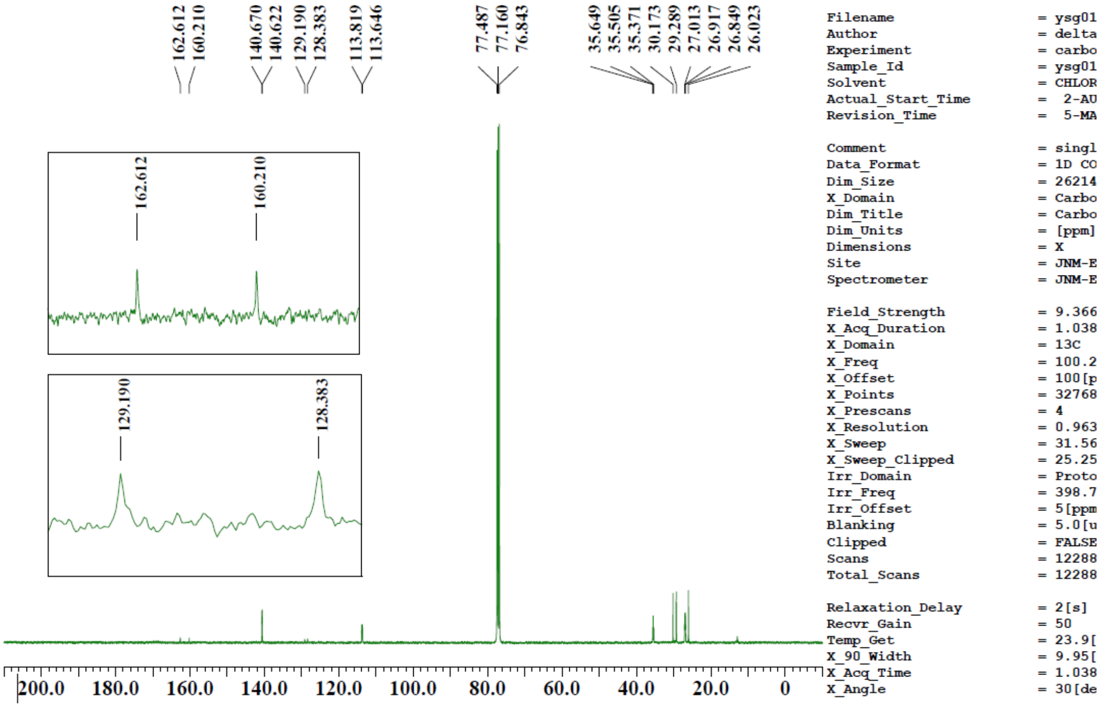

Figure S29. $^{13}C\{^1H\}$ NMR spectrum of **Au$_C$-FF** (100 MHz, $CDCl_3$, r.t.).

Synthesis of [Au_2Ph_2(dcpm)] (Au$_C$-HH).

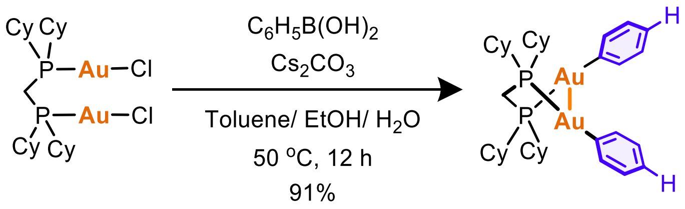

The acyclic Au complex, **Au$_C$-HH**, was synthesized according to the literature[S15] with a slight modification. In a screw capped tube, a mixture of phenylboronic acid (15 mg, 0.12 mmol), Cs_2CO_3 (115 mg, 0.353 mmol) and [Au_2Cl_2(dcpm)] (50 mg, 0.057 mmol) in degassed toluene/ H_2O/ EtOH (2 mL/ 0.5 mL/ 0.5 mL) was stirred for 12 hours at 50 °C under an argon atmosphere. After a mixture was allowed to cool to room temperature, the solvent was evaporated. The residue was dissolved into CH_2Cl_2 (5 mL), and the organic layer was washed with water (5 mL) for 2 times and brine. The organic layer was dried over Na_2SO_4, and filtered off. The filtrate was concentrated to *ca.* 0.5 mL, then poured onto Et_2O (50 mL). The precipitate was collected by suction filtration, then dried in *vacuo*. The desired Au complex **Au$_C$-HH** was obtained as a white solid in 91% yield (50 mg, 0.052 mmol). ^{1}H NMR (400 MHz, $CDCl_3$, r.t.): δ 7.47 (m, 4H, AuC$_6$*H*$_5$), 7.14 (t, 4H, J = 7.4 Hz, AuC$_6$*H*$_5$), 6.96 (t, 2H, J = 7.4 Hz, AuC$_6$*H*$_5$), 2.28-2.18 (m, 4H, PCy), 2.15-2.03 (m, 8H, PCy), 2.01 (t, 2H, J = 9.2 Hz, PC*H*$_2$P), 1.95-1.80 (m, 8H, P*Cy*), 1.78-1.68 (m, 4H, P*Cy*), 1.67-1.40 (m, 8H, P*Cy*), 1.40-1.18 (m, 12H, P*Cy*); ^{31}P{^{1}H} NMR (162 MHz, $CDCl_3$, r.t.): δ 47.7; ^{13}C{^{1}H} NMR (100 MHz, $CDCl_3$, r.t.): δ 174.4 (m), 140.2, 127.2, 125.1, 35.6 (t, J = 14 Hz), 30.2, 29.3, 27.0 (dt, J = 14, 5.7), 26.1 ; IR (ATR): ν = 2920, 2845, 1572, 1444, 1174, 999, 886, 852, 762, 720, 695 cm^{-1}.

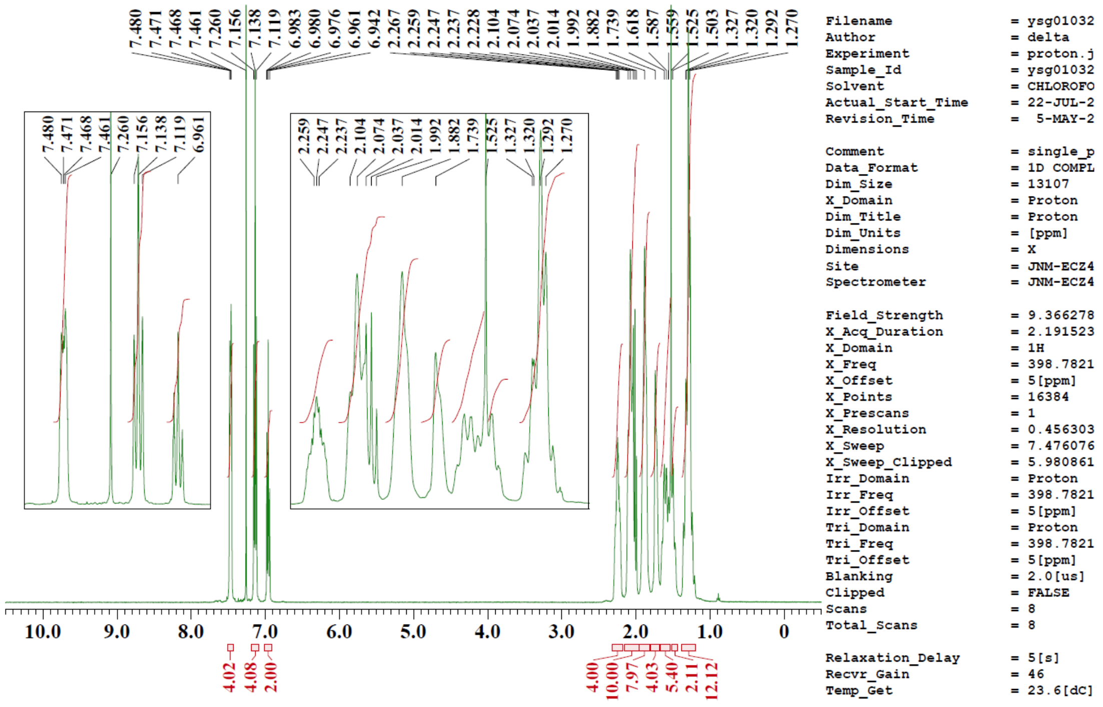

Figure S30. ^{1}H NMR spectrum of **Au$_C$-HH** (400 MHz, $CDCl_3$, r.t.).

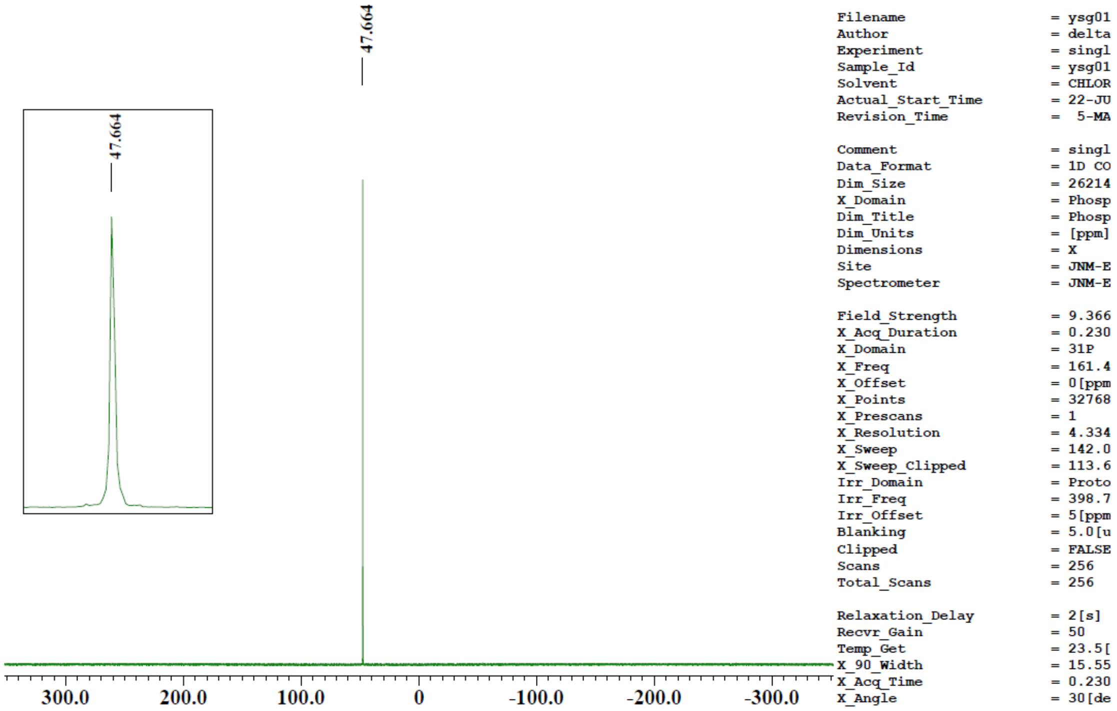

Figure S31. $^{31}P\{^{1}H\}$ NMR spectrum of **Au$_C$-HH** (161 MHz, $CDCl_3$, r.t.).

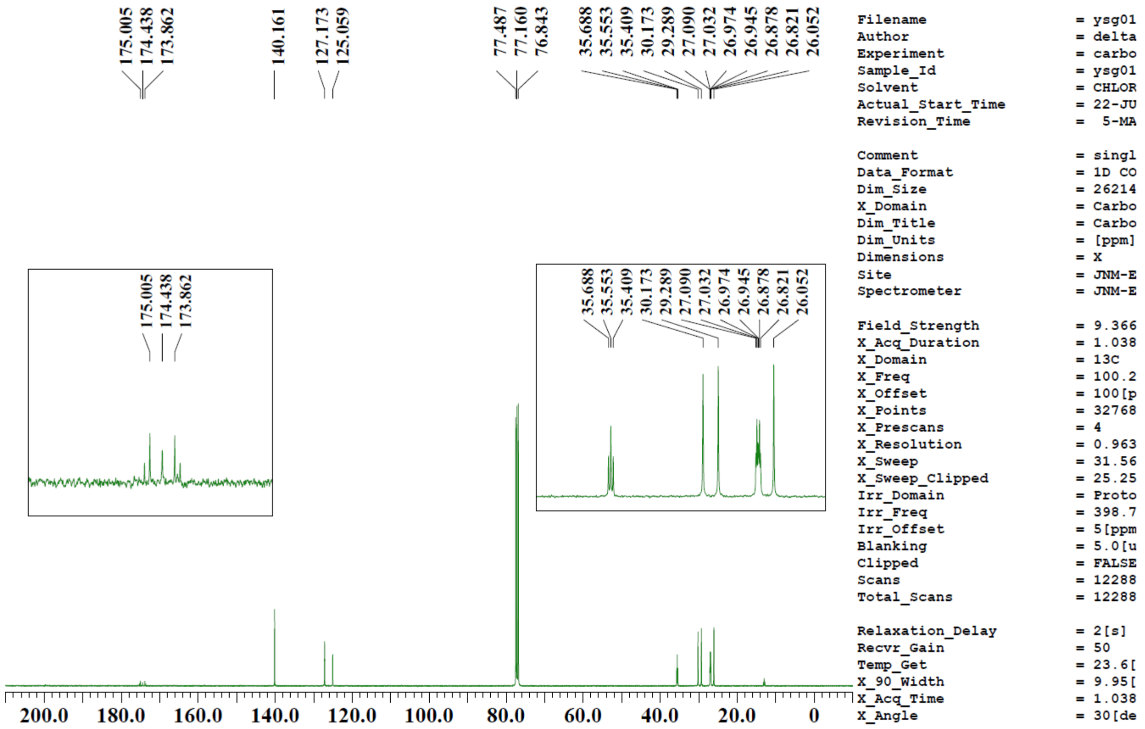

Figure S32. $^{13}C\{^{1}H\}$ NMR spectrum of **Au$_{C}$-HH** (100 MHz, $CDCl_3$, r.t.).

Synthesis of [Au_2Ph_2(dppm)] (AuP-HH).

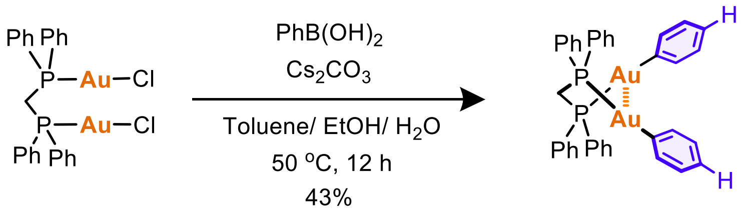

The acyclic Au complex, **AuP-HH**, was synthesized according to the literature[S15] with a slight modification. In a screw capped tube, a mixture of phenylboronic acid (15 mg, 0.12 mmol), Cs_2CO_3 (115 mg, 0.353 mmol) and [Au_2Cl_2(dppm)] (50 mg, 0.059 mmol) in degassed toluene/H_2O/EtOH (2 mL/0.5 mL/0.5 mL) was stirred for 12 hours at 50 °C under an argon atmosphere. After a mixture was allowed to cool to room temperature, the solvent was evaporated. The residue was dissolved into CH_2Cl_2 (5 mL), and the organic layer was washed with water (5 mL) for 2 times and brine. The organic layer was dried over Na_2SO_4, and filtered off. The filtrate was concentrated to *ca.* 0.5 mL, then poured onto Et_2O (50 mL). The precipitate was collected by suction filtration, then dried in *vacuo*. The desired Au complex **AuP-HH** was obtained as a white solid in 43% yield (23 mg, 0.025 mmol). ^{1}H NMR (400 MHz, $CDCl_3$, r.t.): δ 7.77 (m, 8H, AuC_6H_4-4-H and PC_6H_5), 7.46-7.30 (m, 16H, Au$C_6\mathit{H}_4$-4-H and P$C_6\mathit{H}_5$) 7.16 (t, 4H, J = 7.4 Hz, AuC_6H_4-4-*H*), 7.01 (tt, 2H, J = 7.4, 1.3 Hz, Au$C_6\mathit{H}_4$-4-H) 3.62 (t, 2H, J = 9.6, PC*H*$_2$P); ^{31}P{^{1}H} NMR (162 MHz, $CDCl_3$, r.t.): δ 32.30; ^{13}C{^{1}H} NMR (100 MHz, $CDCl_3$, r.t.): δ 172.0 (dd, J = 199, 40 Hz), 140.1, 133.7 (t, J = 7.0 Hz), 130.2 (dd, J = 306, 41 Hz) 131.5 (t, J = 13 Hz), 129.2 (t, J = 5.4 Hz) 127.23 (t, J = 3.2 Hz), 125.4, 30.43 (d, J = 21.5 Hz); IR (ATR): ν = 3049, 1600, 1572, 1481, 1435, 1378, 1332, 1303, 1252, 1185, 1159, 1100, 1055, 1023, 998.0, 923.7, 842.7, 781.0, 736.7, 725.1, 689.4, 617.1, 575.6 cm^{-1}.

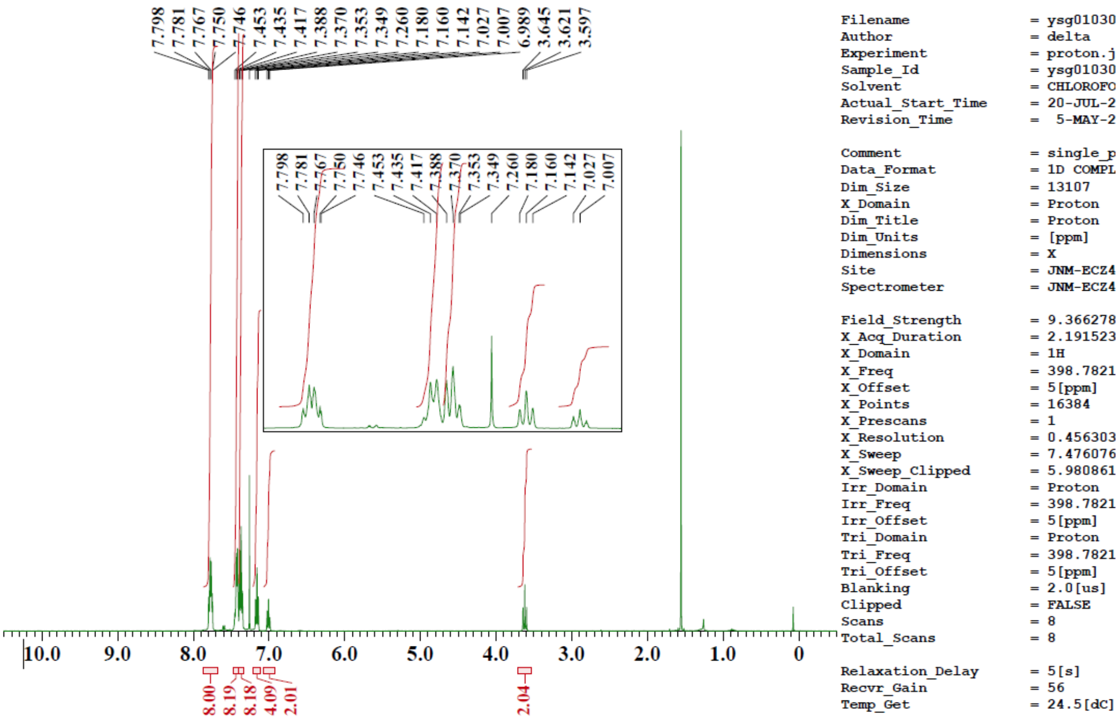

Figure S33. ^{1}H NMR spectrum of **Au$_{P}$-HH** (400 MHz, $CDCl_3$, r.t.).

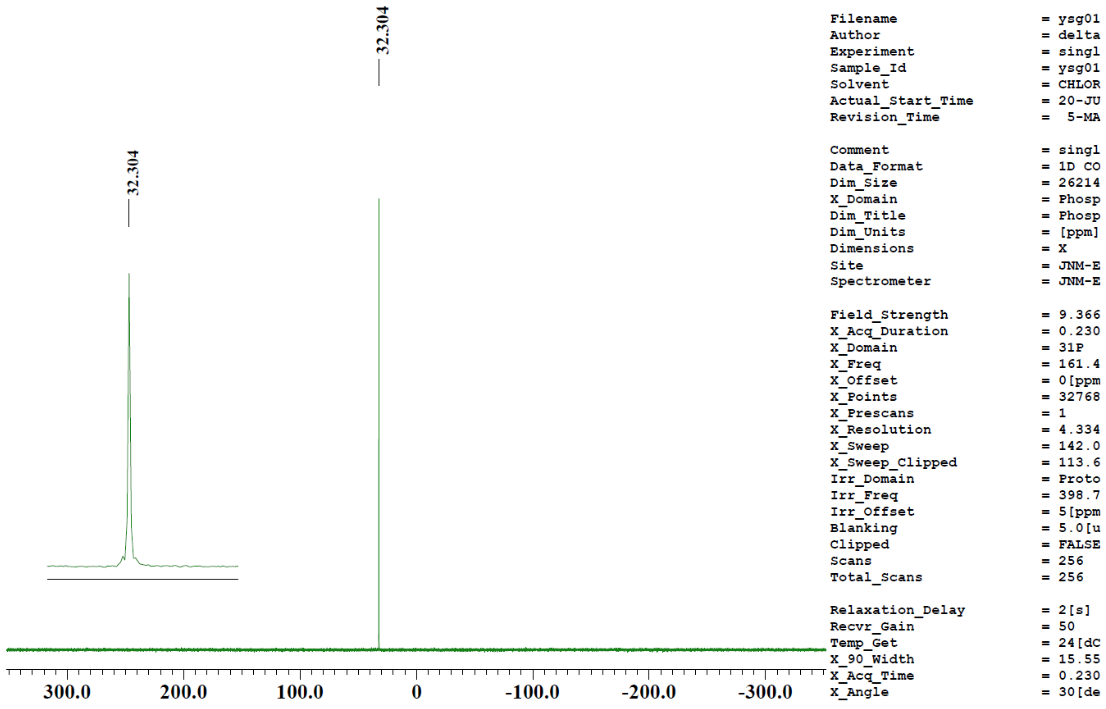

Figure S34. ^{31}P{^{1}H} NMR spectrum of **Au$_{P}$-HH** (161 MHz, $CDCl_3$, r.t.).

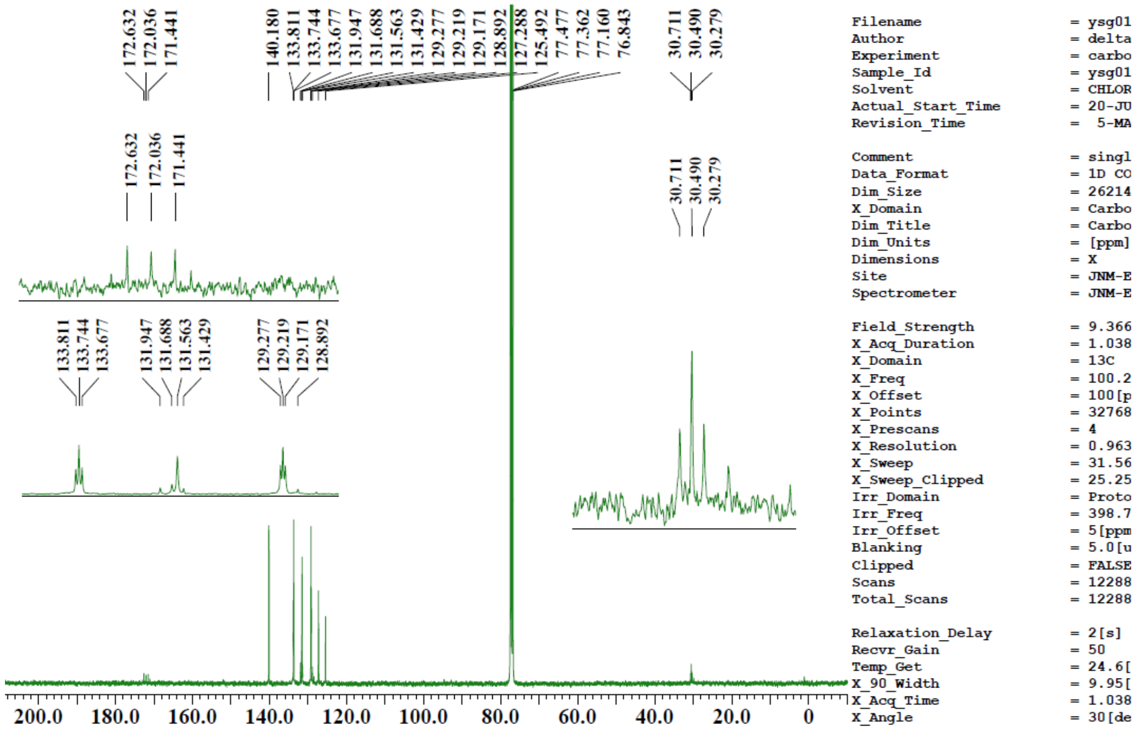

Figure S35. $^{13}C\{^1H\}$ NMR spectrum of **AuP-HH** (100 MHz, $CDCl_3$, r.t.).

Synthesis of [Au_2Ph_2(dppm)] (AuP-FF).

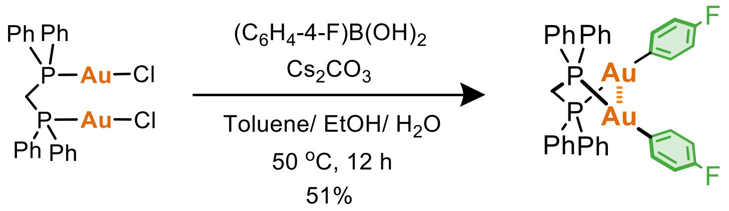

The acyclic Au complex, **AuP-FF**, was synthesized according to the literature[S15] with a slight modification. In a screw capped tube, a mixture of 4-fluorophenylboronic acid (17 mg, 0.12 mmol), Cs_2CO_3 (115 mg, 0.353 mmol) and [Au_2Cl_2(dppm)] (50 mg, 0.059 mmol) in degassed toluene/H_2O/EtOH (2 mL/0.5 mL/0.5 mL) was stirred for 12 hours at 50 °C under an argon atmosphere. After a mixture was allowed to cool to room temperature, the solvent was evaporated. The residue was dissolved into CH_2Cl_2 (5 mL), and the organic layer was washed with water (5 mL) for 2 times and brine. The organic

layer was dried over Na_2SO_4, and filtered off. The filtrate was concentrated to *ca.* 0.5 mL, then poured onto Et_2O (50 mL). The precipitate was collected by suction filtration, then dried in *vacuo*. The desired Au complex **Au$_P$-FF** was obtained as a white solid in 51% yield (29 mg, 0.030 mmol). Purity was confirmed by comparison of ^1H NMR spectrum of the product with a reported spectrum.[S16] ^1H NMR ($CDCl_3$, 400 MHz, r.t.): δ 7.81–7.72 (m, 6H), 7.53–7.41 (m, 4H), 7.40–7.37 (m, 10H), 7.35–7.30 (m, 4H), 6.83 (t, J = 5Hz, 4H), 3.68 (t, J = 10 Hz, 2H). $^{31}P\{^1H\}$ NMR ($CDCl_3$, 162 MHz, r.t.): δ 31.8. ^{19}F NMR ($CDCl_3$, 376 MHz, r.t.): -117.8.

Synthesis of [Au_2Ph_2(dcpm)] (Au$_C$-DD).

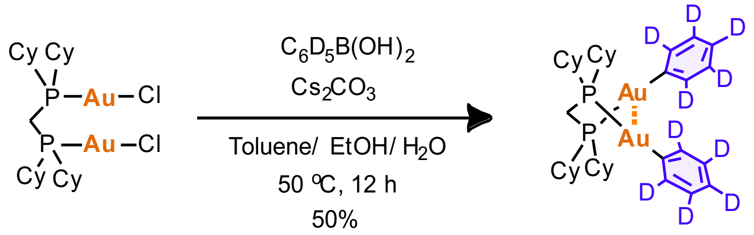

The acyclic Au complex, **Au$_C$-DD**, was synthesized according to the literature[S15] with a slight modification. In a screw capped tube, a mixture of $C_6D_5B(OH)_2$ (15 mg, 0.12 mmol), Cs_2CO_3 (115 mg, 0.353 mmol) and [Au_2Cl_2(dcpm)] (50 mg, 0.056 mmol) in degassed toluene/H_2O/EtOH (2 mL/0.5 mL/0.5 mL) was stirred for 12 hours at 50 °C under an argon atmosphere. After a mixture was allowed to cool to room temperature, the solvent was evaporated. The residue was dissolved into CH_2Cl_2 (5 mL), and the organic layer was washed with water (5 mL) for 2 times and brine. The organic layer was dried over Na_2SO_4, and filtered off. The filtrate was concentrated to *ca.* 0.5 mL, then poured onto Et_2O (50 mL). The precipitate was collected by suction filtration, then dried in *vacuo*. The desired Au complex **Au$_C$-DD** was obtained as a white solid in 50%

yield (27 mg, 0.028 mmol). ^{1}H NMR (400 MHz, $CDCl_3$, r.t.): δ 2.30-2.15 (m, 4H, PCy), 2.13-1.95 (m, 8H, PCy), 2.00 (t, 2H, J = 9.0 Hz, PCH_2P), 1.94-1.79 (m, 8H, P*Cy*), 1.76-1.65 (m, 4H, P*Cy*), 1.66-1.40 (m, 8H, P*Cy*), 1.37-1.17 (m, 12H, P*Cy*); ^{31}P{^{1}H} NMR (162 MHz, $CDCl_3$, r.t.): δ 47.7; ^{13}C{^{1}H} NMR (100 MHz, $CDCl_3$, r.t.): δ 174.1 (m), 139.7 (t, J = 24 Hz), 126.6 (t, J = 24 Hz), 124.5 (t, J = 25 Hz), 35.5 (t, J = 14 Hz), 30.1, 29.3, 26.9 (dt, J = 16, 6.3), 26.0; IR (ATR): v = 2919, 2846, 1444, 1268, 1174, 1000, 761.7, 531.3 cm^{-1}.

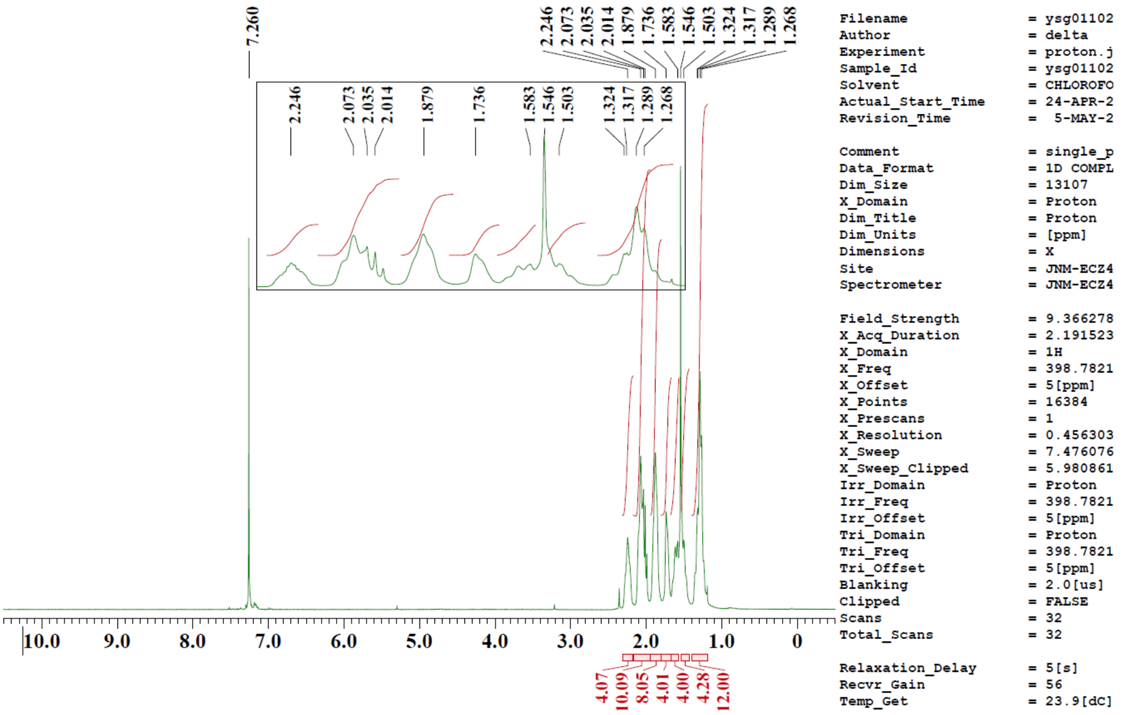

Figure S36. ^{1}H NMR spectrum of **Au$_C$-DD** (400 MHz, $CDCl_3$, r.t.).

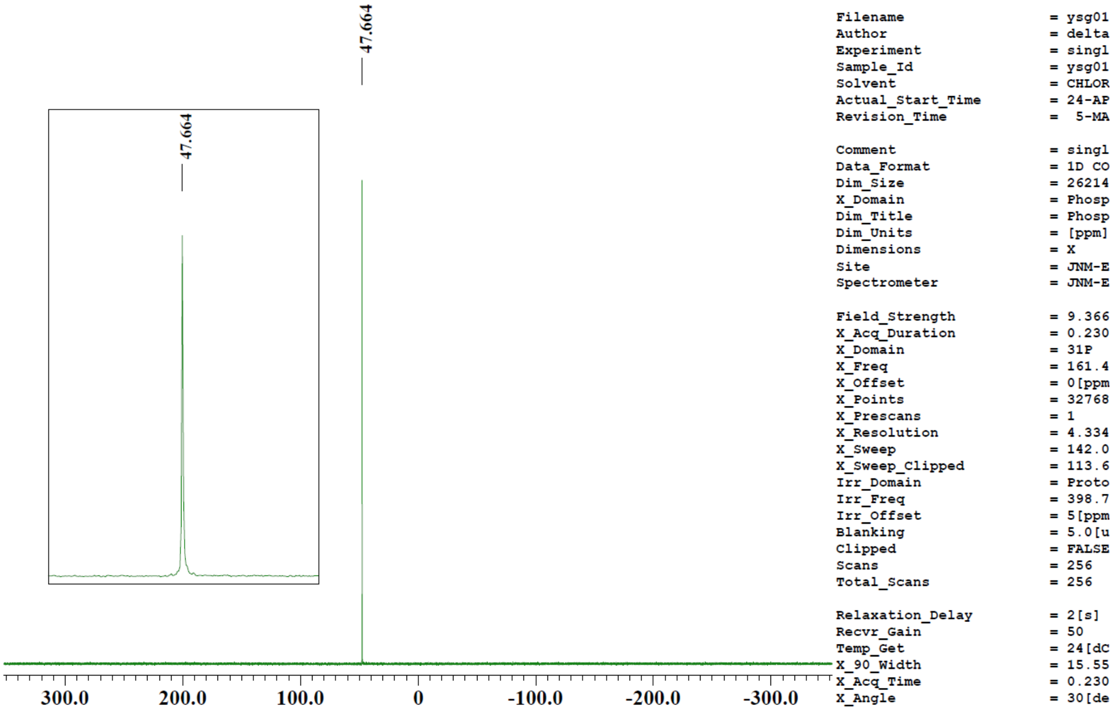

Figure S37. $^{31}P\{^1H\}$ NMR spectrum of **Au$_C$-DD** (161 MHz, $CDCl_3$, r.t.).

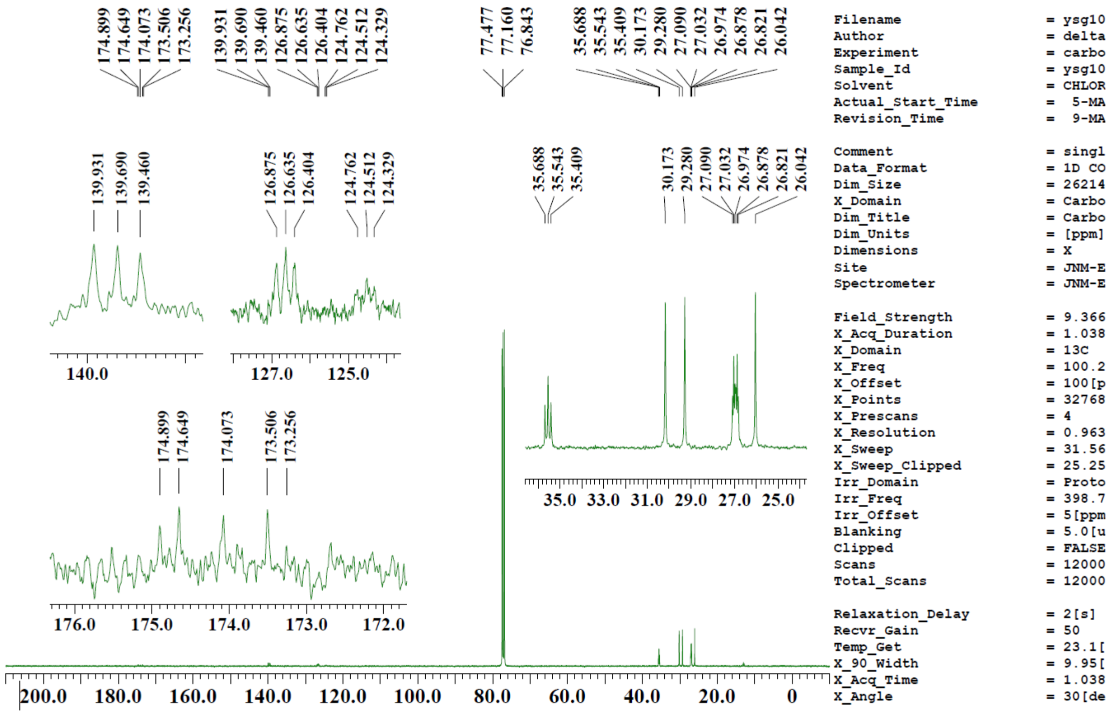

Figure S38. $^{13}C\{^1H\}$ NMR spectrum of **Au$_C$-DD** (100 MHz, $CDCl_3$, r.t.).

Preparation of a mixture of Au$_C$-HH, Au$_C$FF and [$Au_2Ph(C_6H_4$-4-F)(dcpm)] (Au$_C$-HF).

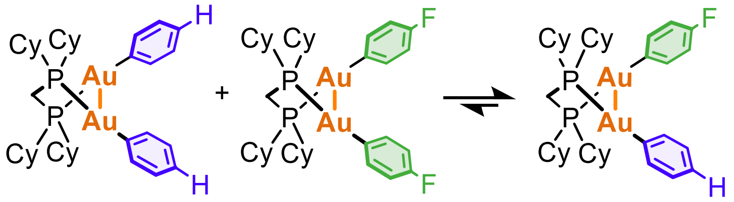

A $CDCl_3$ (1 mL) solution of acyclic Au complexes, **Au$_C$-HH** (2.0 mg, 2.0 μmol) and **Au$_C$-FF** (2.0 mg, 2.0 μmol), was stirred at 25 °C for 4 h. The resulting solution was subjected into the NMR analysis without any purification. The formation of **Au$_C$-HF** was observed in a mixture of the above Au complexes, and any by-product was not observed. The following data shows the chemical shifts of **Au$_C$-HF** extracted from NMR spectra of the mixture. ^{1}H NMR (400 MHz, $CDCl_3$, r.t.): δ 7.50-7.32 (m, 4H, AuC_6*H_4*-4-H), 7.16-7.08 (m, 2H, AuC_6*H_4*-4-H) 7.00-6.90 (m, 1H, AuC_6H_4-4-*H*), 6-89-6.78 (m, 2H , AuC_6*H_4*-4-H) 2.30-2.15 (m, 4H, P*Cy*), 2.13-1.95 (m, 8H, P*Cy*) 2.00 (t, 2H, J = 9.0, PC*H_2*P), 1.95-1.79(m, 8H, P*Cy*) 1.79-1.66 (m, 4H, P*Cy*), 1.65-1.40 (m, 8H, P*Cy*), 1.38-1.15 (m, 12H, P*Cy*); ^{19}F NMR (376 MHz, $CDCl_3$, r.t.): δ -117.8; ^{31}P{^{1}H} NMR (162 MHz, $CDCl_3$, r.t.): δ 47.8; ^{13}C{^{1}H} NMR (100 MHz, $CDCl_3$, r.t.): δ 140.8 (m), 140.2 (d, J = 8.9 Hz), 127.2 (m), 125.1 (m), 113.7 (m), 35.5 (m), 30.2, 29.3 (m), 26.9 (m) 26.8 (m), 26.0; some of peaks could not be obtained because of the equilibrated mixture of the Au complexes, **Au$_C$-HH**, **Au$_C$-FF** and **Au$_C$-HF**.

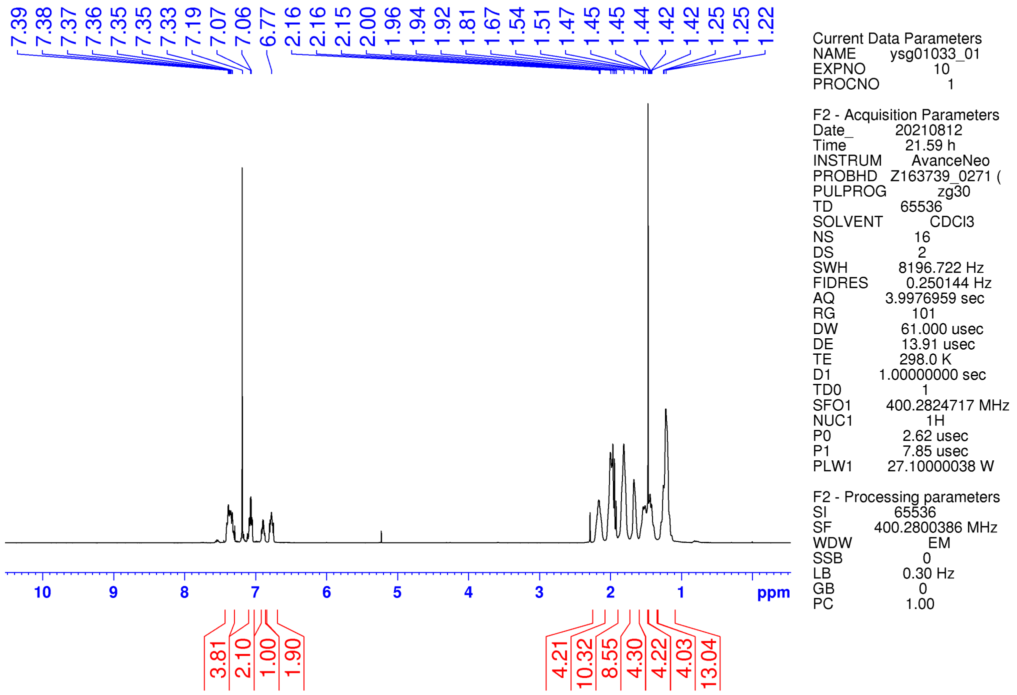

Figure S39. ^{1}H NMR spectrum of the equilibrated mixture of the Au complexes, **Au$_{C}$-HH**, **Au$_{C}$-FF** and **Au$_{C}$-HF** (400 MHz, $CDCl_3$, r.t.).

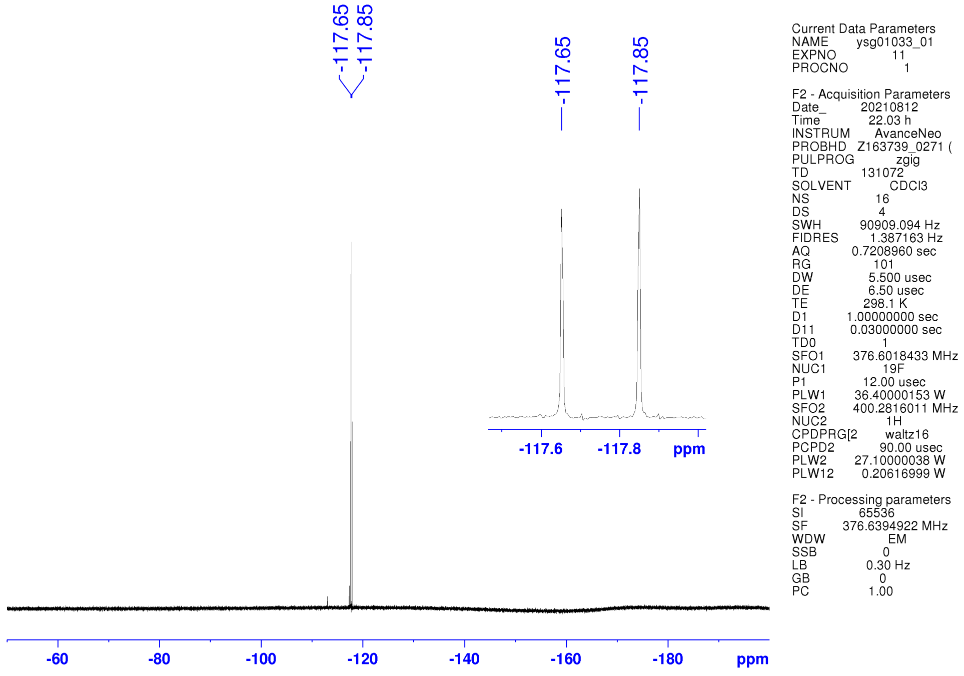

Figure S40. ^{19}F NMR spectrum of the equilibrated mixture of the Au complexes, **Au$_{C}$-HH**, **Au$_{C}$-FF** and **Au$_{C}$-HF** (375 MHz, $CDCl_3$, r.t.).

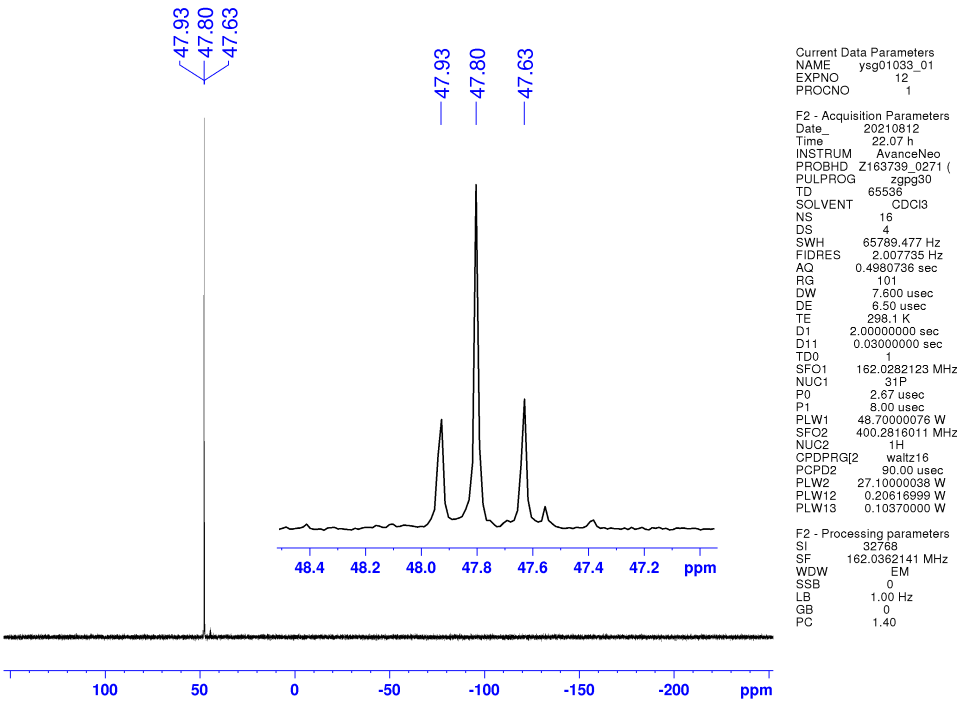

Figure S41. $^{31}P\{^{1}H\}$ NMR spectrum of the equilibrated mixture of the Au complexes, **Au$_C$-HH**, **Au$_C$-FF** and **Au$_C$-HF** (161 MHz, $CDCl_3$, r.t.).

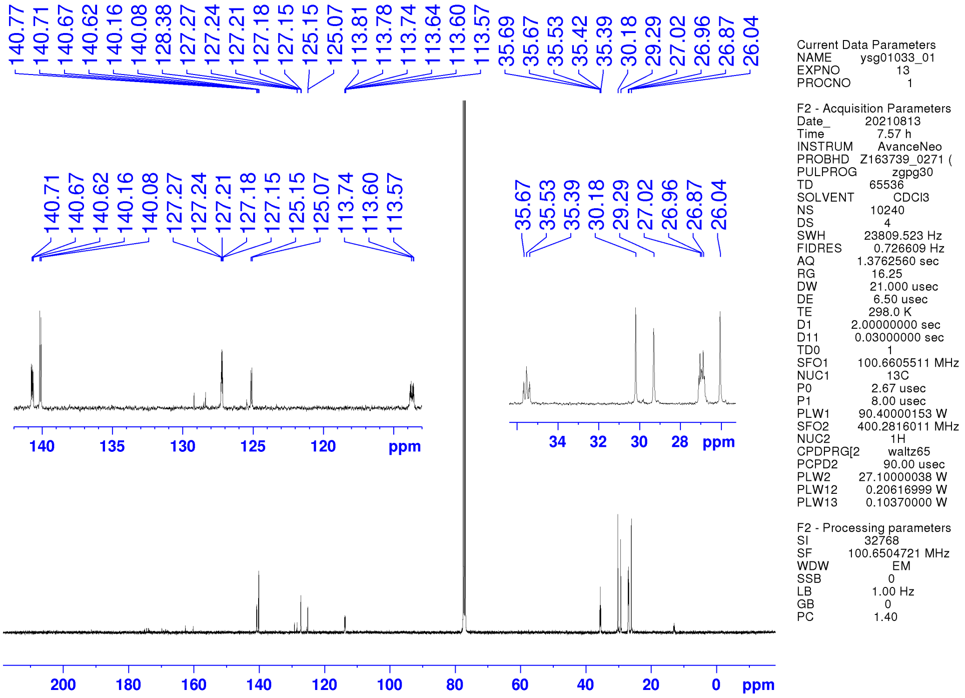

Figure S42. $^{13}C\{^{1}H\}$ NMR spectrum of the equilibrated mixture of the Au complexes, **Au$_C$-HH**, **Au$_C$-FF** and **Au$_C$-HF** (100 MHz, $CDCl_3$, r.t.).

S3. Kinetics Study

Kinetics experiments were performed using NMR techniques. A reported rate constant represents the average of two or more experiments. A stock solution of deuterated solvent, $CDCl_3$, containing 0.001% v/v 1,3,5-tris(trifluoromethyl)benzene as an internal standard, was used. The temperature of the probe was calibrated using a CH_3OH standard. The production of **Au$_C$-HF** was confirmed by comparison to the ^{1}H and ^{19}F NMR chemical shifts.

General Procedure on NMR Experiments for Determination of the Rate Constants.

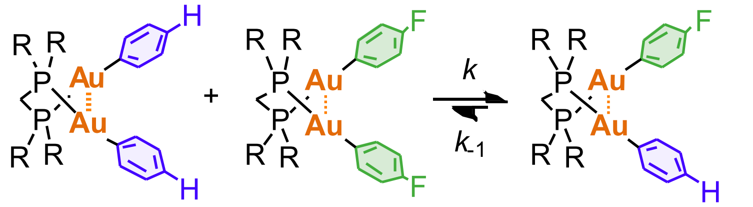

A typical procedure on determination of the rate constants for conproportionation (k_1) and disproportionation (k_{-1}) of the reaction in a mixture of **Au$_C$-HH** and **Au$_C$-FF** is explained in the following section.

A solution of [Au_2Ph_2(dcpm)] (**Au$_C$-HH**) (1.0 mg, 1.0 µmol) in 0.3 mL of $CDCl_3$ (0.001% v/v 1,3,5-tris(trifluoromethyl)benzene) was injected to an NMR test tube, then cooled to -20 °C. To the test tube, a cooled solution of [$Au_2(4\text{-}F\text{-}C_6H_4)_2$(dcpm)] (**Au$_C$-HF**) (1.0 mg, 1.0 µmol) in 0.3 mL of $CDCl_3$ (0.001% v/v 1,3,5-tris(trifluoromethyl)benzene) was added. The test tube was inserted to an NMR spectrometer which was pre-cooled to -20 °C. The progress of the reaction was monitored by NMR measurements.

Summary of Kinetics Study.

Table S4. Rate constants for conproportionation (k_1) and disproportionation (k_{-1}).

Entry	Solvent	Temp. /°C	k_1 /M^{-1}·s^{-1} [a]	k_{-1} /M^{-1}·s^{-1} [a]
1	$CDCl_3$	0	0.27 ± 0.045	(7.7 ± 0.87) × 10^{-2}
2	$CDCl_3$	0	0.33 ± 0.032	(9.5 ± 0.73) × 10^{-2}
		(ave.)	0.30 ± 0.039	(8.6 ± 0.80) × 10^{-2}
3	$CDCl_3$	-10	0.21 ± 0.037	(5.9 ± 0.69) × 10^{-2}
4	$CDCl_3$	-10	0.15 ± 0.013	(3.2 ± 0.18) × 10^{-2}
		(ave.)	0.18 ± 0.025	(4.6 ± 0.87) × 10^{-2}
5	$CDCl_3$	-20	(5.3 ± 0.40) × 10^{-2}	(1.1 ± 0.051) × 10^{-2}
7	$CDCl_3$	-20	(6.0 ± 0.58) × 10^{-2}	(1.1 ± 0.068) × 10^{-2}
8	$CDCl_3$	-20	(9.4 ± 0.94) × 10^{-2}	(1.9 ± 0.12) × 10^{-2}
		(ave.)	(6.9 ± 0.64) × 10^{-2}	(1.4 ± 0.080) × 10^{-2}
9[c]	$CDCl_3$	0	n.r.	n.r.
10[c]	$CDCl_3$	0	n.r.	n.r.
		(ave.)	---	---
11[d]	$CDCl_3$	0	(2.6 ± 0.40) × 10^{-2}	(1.0 ± 0.10) × 10^{-2}
12[d]	$CDCl_3$	0	(2.5 ± 0.30) × 10^{-2}	(1.0 ± 0.075) × 10^{-2}
		(ave.)	(2.6 ± 0.35) × 10^{-2}	(1.0 ± 0.088) × 10^{-2}
13	Acetone-d_6	0	(7.1 ± 2.0) × 10^{-2}	(8.8 ± 2.1) × 10^{-2}
14		0	(6.7 ± 1.1) × 10^{-2}	(7.7 ± 0.9) × 10^{-2}
		(ave.)	(6.9 ± 1.6) × 10^{-2}	(8.3 ± 1.5) × 10^{-2}
15	DMF-d_7	0	n.r.	n.r.
16	DMF-d_7	0	n.r.	n.r.
		(ave.)	--	--
17	DMF-d_7	25	(7.2 ± 1.0) × 10^{-2}	(3.5 ± 0.36) × 10^{-2}
18	DMF-d_7	25	(9.0 ± 1.3) × 10^{-2}	(4.3 ± 0.50) × 10^{-2}
		(ave.)	(8.1 ± 1.2) × 10^{-2}	(3.9 ± 0.43) × 10^{-2}

a. Rate constants (k and k_{-1}) were estimated by linear regression analysis[b]. The corresponding reaction profiles were in good agreement with a reversible second-order reaction model. [S17]

b. The following equation was used for the linear regression analysis.

$$(k_1 - 4k_{-1})Qt = \ln \frac{([\mathbf{Au_C\text{-}FF}]_0 - [\mathbf{Au_C\text{-}FF}]_e)([\mathbf{Au_C\text{-}FF}] - [\mathbf{Au_C\text{-}FF}]_e + Q)}{([\mathbf{Au_C\text{-}FF}] - [\mathbf{Au_C\text{-}FF}]_e)([\mathbf{Au_C\text{-}FF}]_0 - [\mathbf{Au_C\text{-}FF}]_e + Q)}$$

$$\text{where } Q = \frac{1}{K-1}\sqrt{K^2([\mathbf{Au_C\text{-}HH}]_0 - [\mathbf{Au_C\text{-}FF}]_0)^2 + 4([\mathbf{Au_C\text{-}HH}]_0[\mathbf{Au_C\text{-}FF}]_0 K)}$$

$$K = \frac{1}{4}K_e = \frac{1}{4}\frac{[\mathbf{Au_C\text{-}FH}]_e^2}{[\mathbf{Au_C\text{-}HH}]_e[\mathbf{Au_C\text{-}FF}]_e}$$

k_1, k_{-1} : Rate constant ($M^{-1}s^{-1}$)
t : Reaction time (s)
[X] : Concentration (M)
$[X]_0$: Initial concentration (M)
$[X]_e$: Equilibrium concentration (M)
K_e : Equilibrium constant

c. In presence with an extra amount of an ancirally ligand, dcpm (~ 1.0 equiv. of Au complexes).

d. In presence with a trace amount of an ancirally ligand, dcpm (~ 0.05 equiv. of Au complexes).

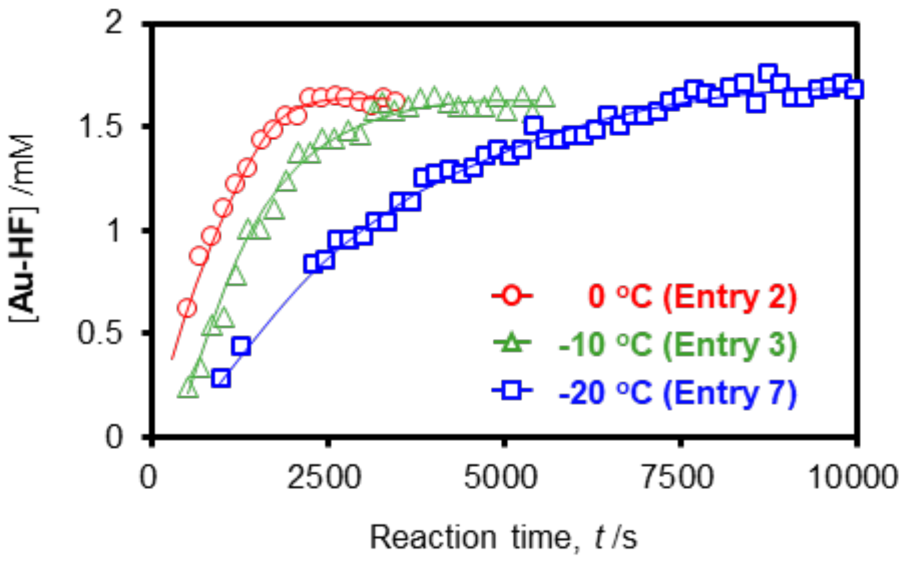

Figure S43. The reaction profiles ([**Au$_C$-HF**] *vs.* *t*) of the reaction in a mixture of **Au$_C$-HH** and **Au$_C$-FF** in $CDCl_3$ at 0, -10 and -20 °C.

We conducted NMR studies of the ligand exchange reaction using an equimolar mixture of [Au_2Ph_2(dcpm)] (**Au$_C$-HH**) and [$Au_2(C_6H_4$-4-F$)_2$(dcpm)] (**Au$_C$-FF**) in presence of an extra amount (Table S4, Entry 9 and 10, ~ 1.0 equiv. of Au complexes) or a trace amount (Table S4, Entry 11 and 12, ~ 0.05 equiv. of Au complexes) of ancillary ligand, dcpm. In case of an extra amount of dcpm, aryl ligand exchange did not occur. In other case of a trace amount of dcpm, the reaction was clearly slowdown that kinetic rates were estimated to be in $k_1 = (2.6 \pm 0.35) \times 10^{-2}$, $k_{-1} = (1.0 \pm 0.088) \times 10^{-2}$, lower than the reaction performed under the same conditions in $CDCl_3$ ($k_1 = 0.30 \pm 0.039$, $k_{-1} = (8.6 \pm 0.80) \times 10^{-2}$).

We also conducted NMR studies of the ligand exchange reaction using an equimolar mixture of [Au_2Ph_2(dcpm)] (**Au$_C$-HH**) and [$Au_2(C_6H_4$-4-F$)_2$(dcpm)] (**Au$_C$-FF**) in the polar solvents such as acetone-d_6 and DMF-d_7 at 0 °C (Table S4). The kinetic rates of comproportionation in acetone-d_6 at 0 °C was estimated to be in $k_1 = (6.9 \pm 1.6)$

× 10^{-2}, k_{-1} = (8.3 ± 1.5) × 10^{-2}, lower than the reaction performed under the same conditions in $CDCl_3$ (k_1 = 0.30 ± 0.039, k_{-1} = (8.6 ± 0.80) × 10^{-2}). The reaction in DMF-d_7 required even higher reaction temperatures than those in the above solvents, and the kinetic rates of the reaction conducted at 25 °C were estimated to be k_1 = (8.1 ± 1.2) × 10^{-2} and k_{-1} = (3.9 ± 0.43) × 10^{-2}. These experiments show that the reaction rates tend to be slower as increasing the solvent polarity or lone-pair donating ability.

The above results are probably due to the formation of a non-reactive complex by the dissociation of one P atom of dcpm stabilized by a polar solvent, and/or by the coordination of the electron-donating molecules (dcpm, or some of solvent molecules) to Au atoms of complex (See Scheme S2).

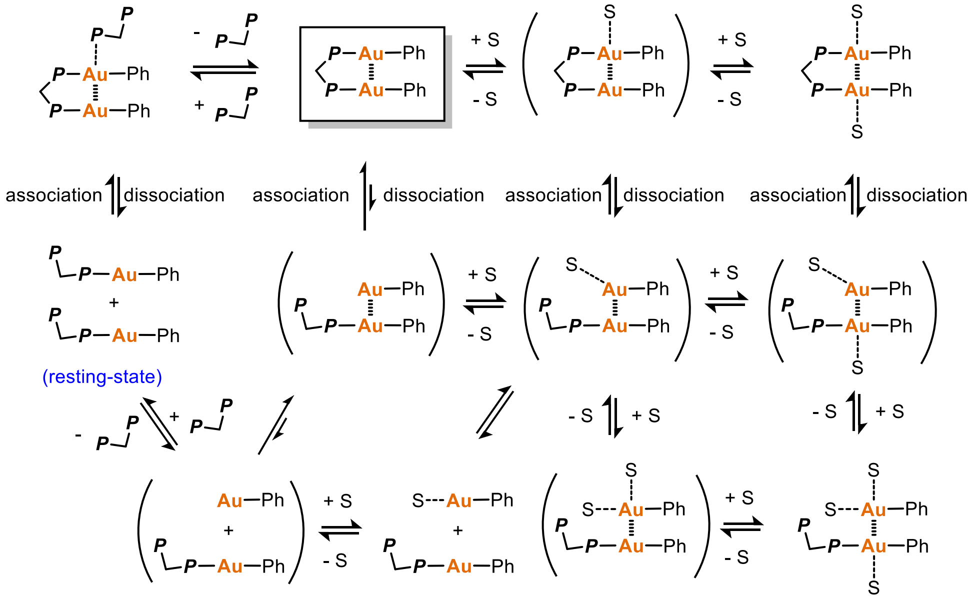

Scheme S2. Possible structures of Au complexes in the solution. Taking into account experimental results shown in the Table S4, complexes indexed by "(resting-state)" are inactive species in the aryl ligand exchange reaction.

NMR Spectra during the Aryl Ligand Exchange Reaction

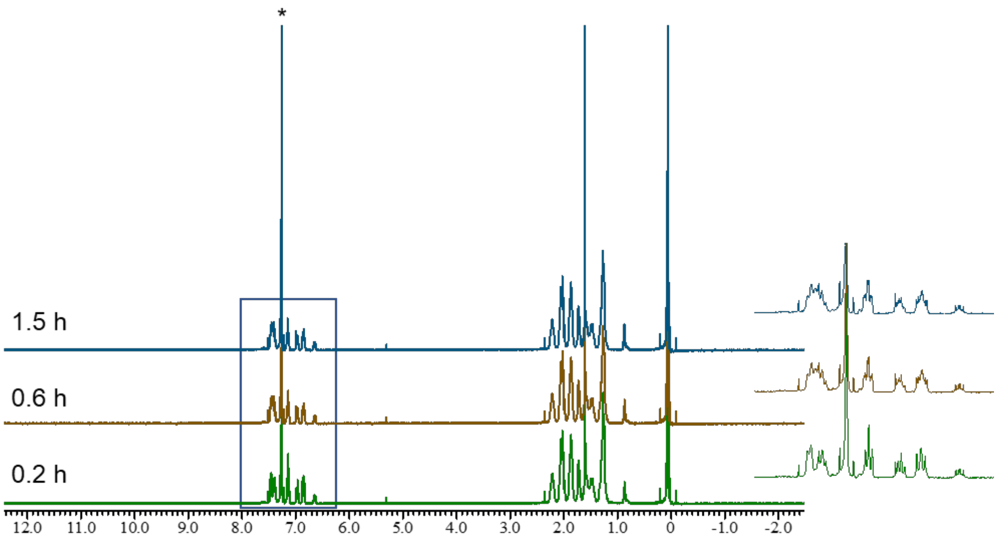

Figure S44. ^{1}H NMR spectra of the reaction mixture of expetiment for entry 1 in Table S4 (400 MHz, $CDCl_3$, 0 °C).

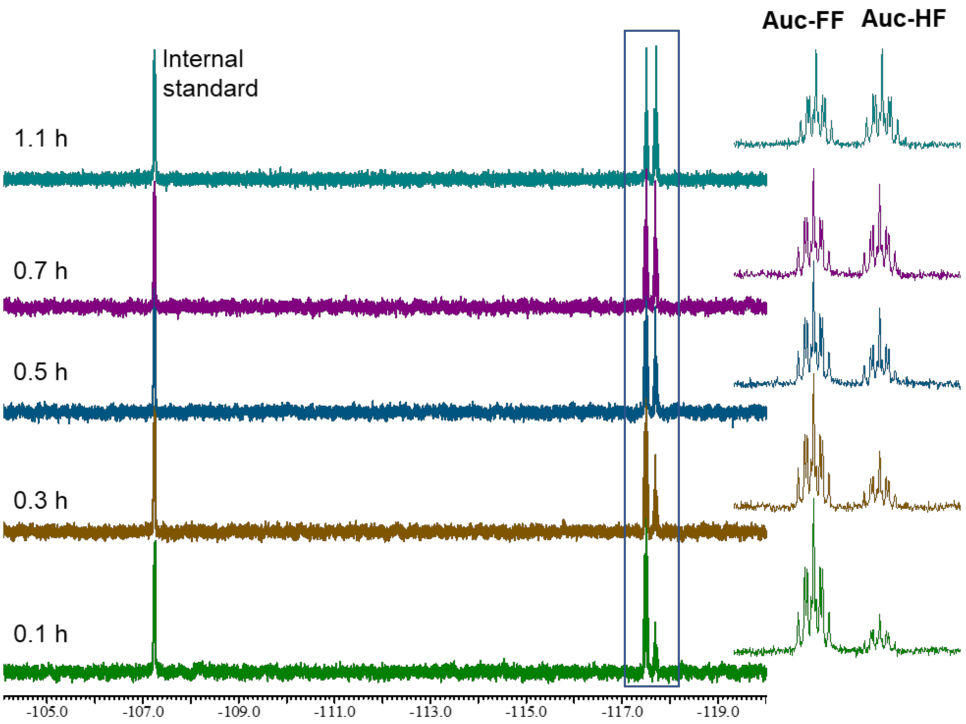

Figure S45. ^{19}F NMR spectra of the reaction mixture of expetiment for entry 1 in Table S4 (375 MHz, $CDCl_3$, 0 °C).

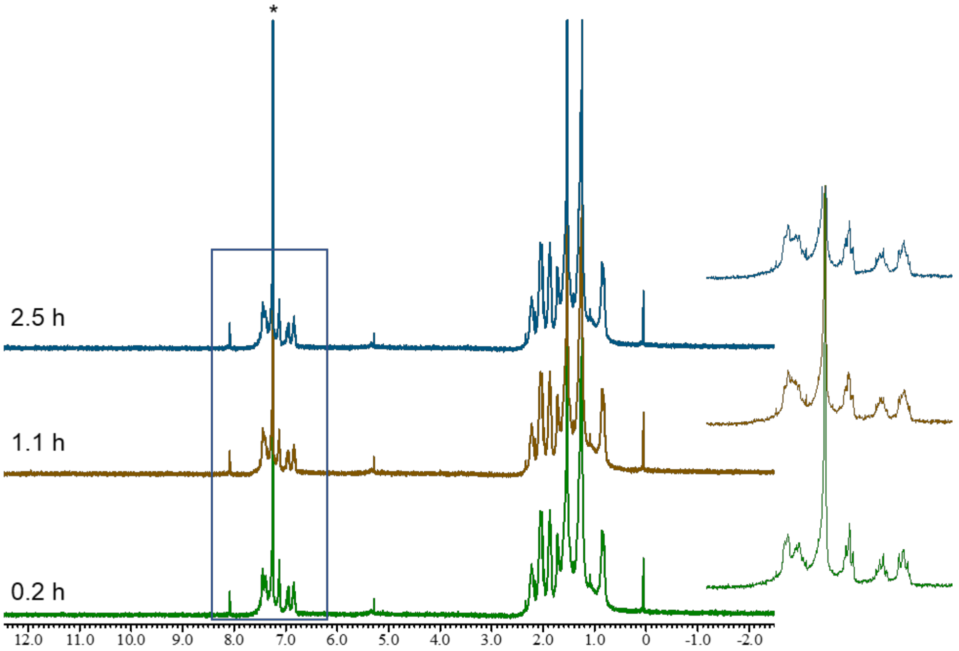

Figure S46. ^{1}H NMR spectra of the reaction mixture of expetiment for entry 3 in Table S4 (400 MHz, $CDCl_3$, -10 ºC).

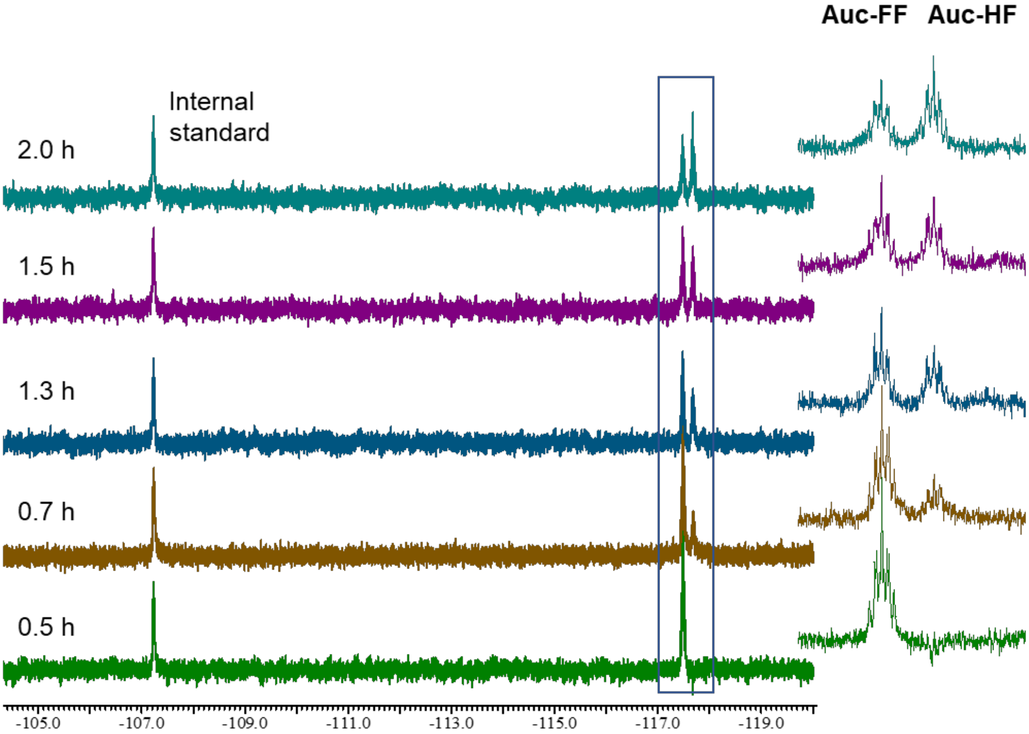

Figure S47. ^{19}F NMR spectra of the reaction mixture of expetiment for entry 3 in Table S4 (375 MHz, $CDCl_3$, -10 ºC).

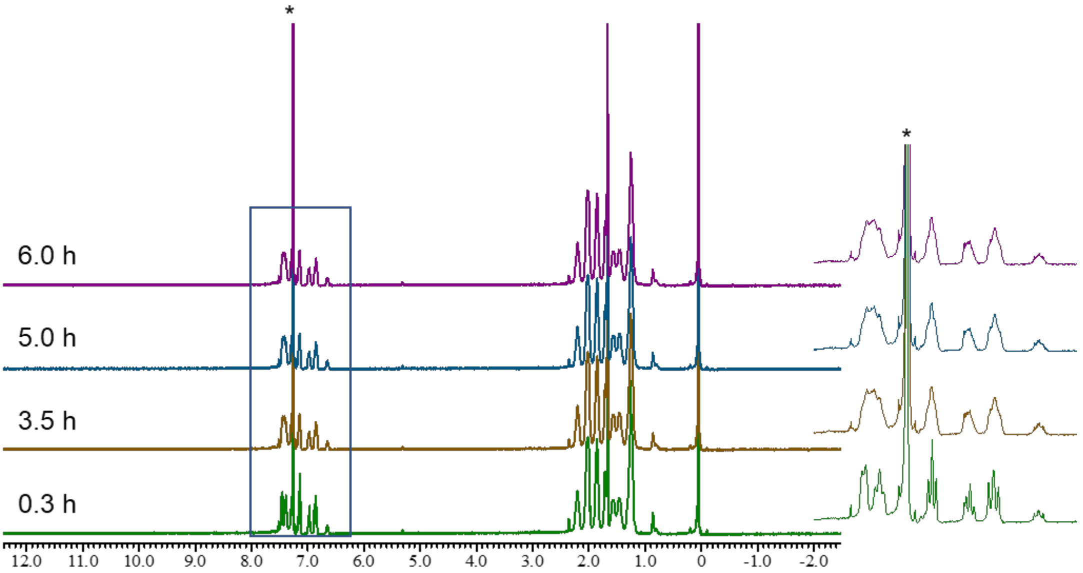

Figure S48. ^{1}H NMR spectra of the reaction mixture of expetiment for entry 5 in Table S4 (400 MHz, $CDCl_3$, -20 °C).

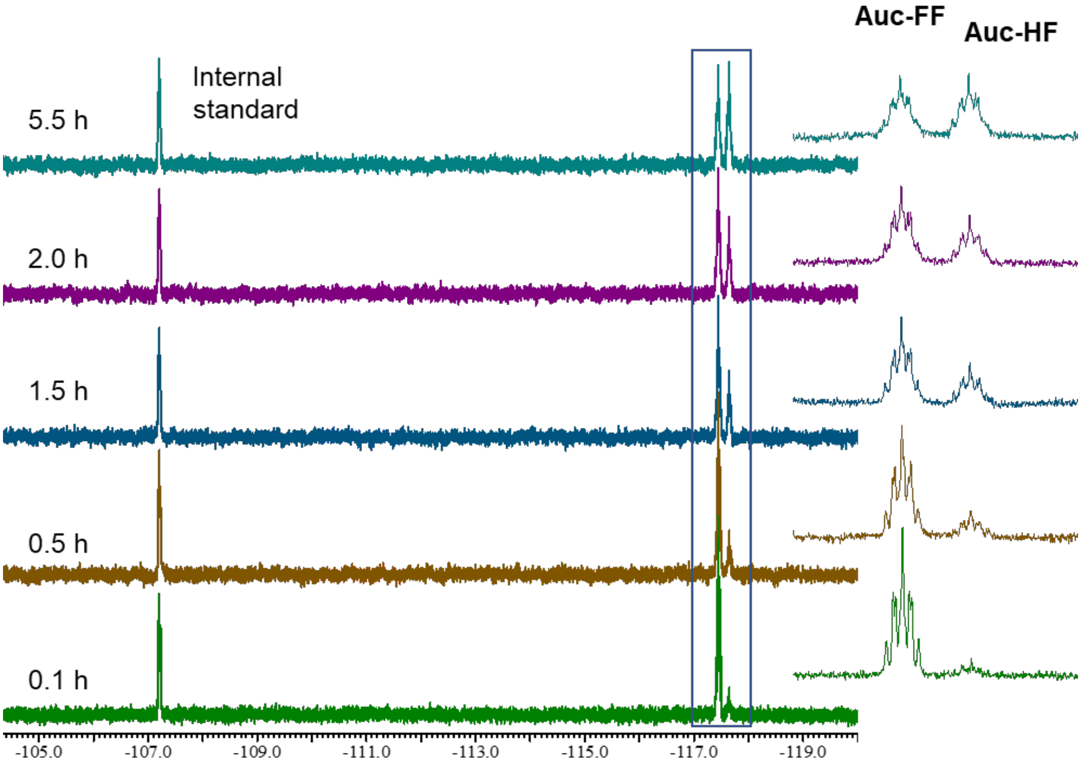

Figure S49. ^{19}F NMR spectra of the reaction mixture of expetiment for entry 5 in Table S4 (375 MHz, $CDCl_3$, -20 °C).

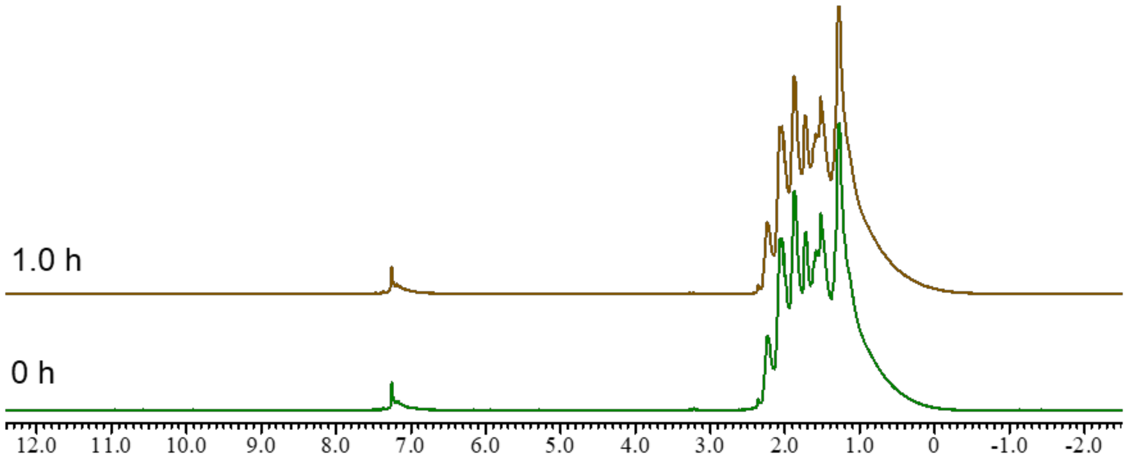

Figure S50. ^{1}H NMR spectra of the reaction mixture of expetiment for entry 11 in Table S4 (400 MHz, $CDCl_3$, 0 °C).

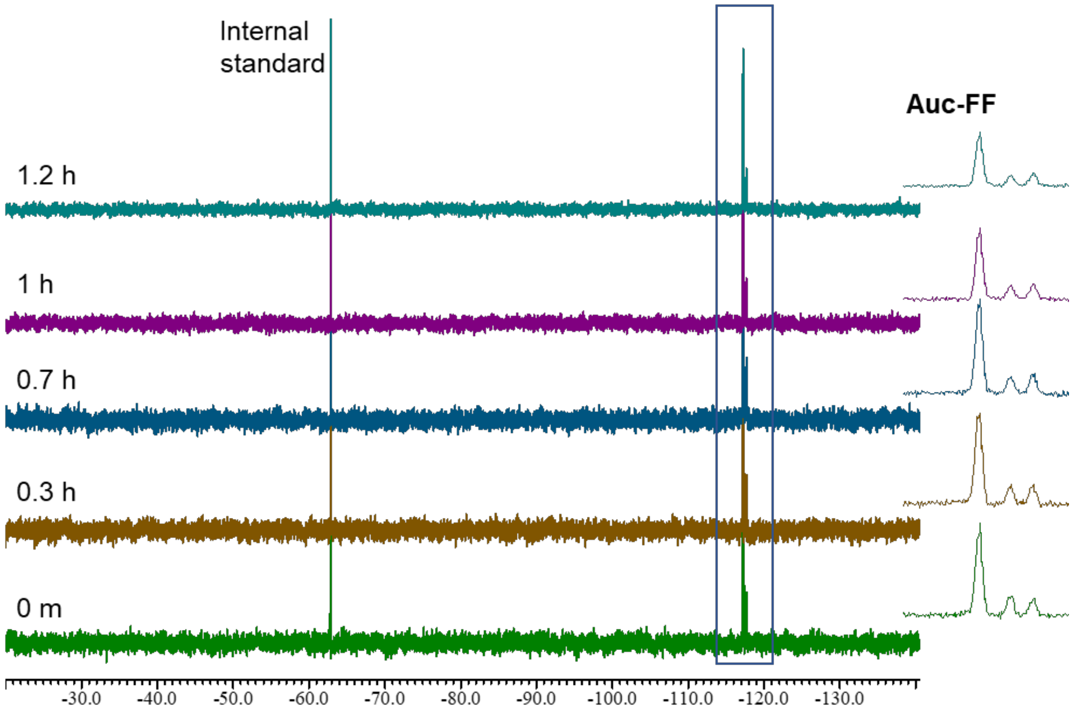

Figure S51. ^{19}F NMR spectra of the reaction mixture of expetiment for entry 9 in Table S4 (375 MHz, $CDCl_3$, 0 °C).

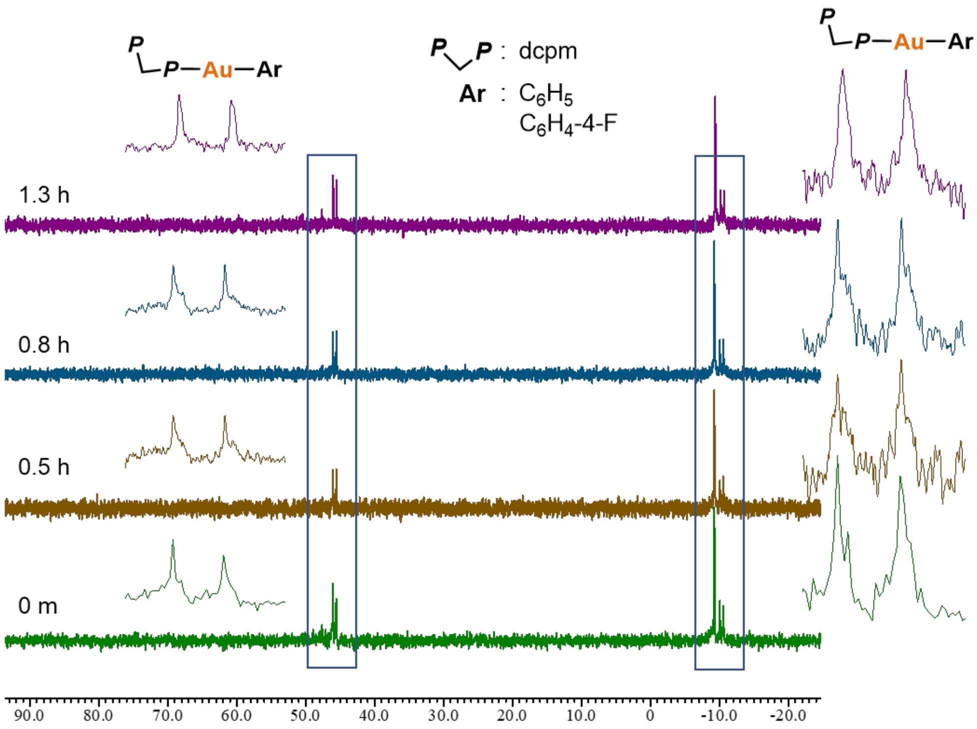

Figure S52. ^{31}P{^{1}H} NMR spectra of the reaction mixture of expetiment for entry 9 in Table S4 (161 MHz, $CDCl_3$, 0 °C). Formation of mononuclear Au complex was obtaind to slowdown desired reaction.

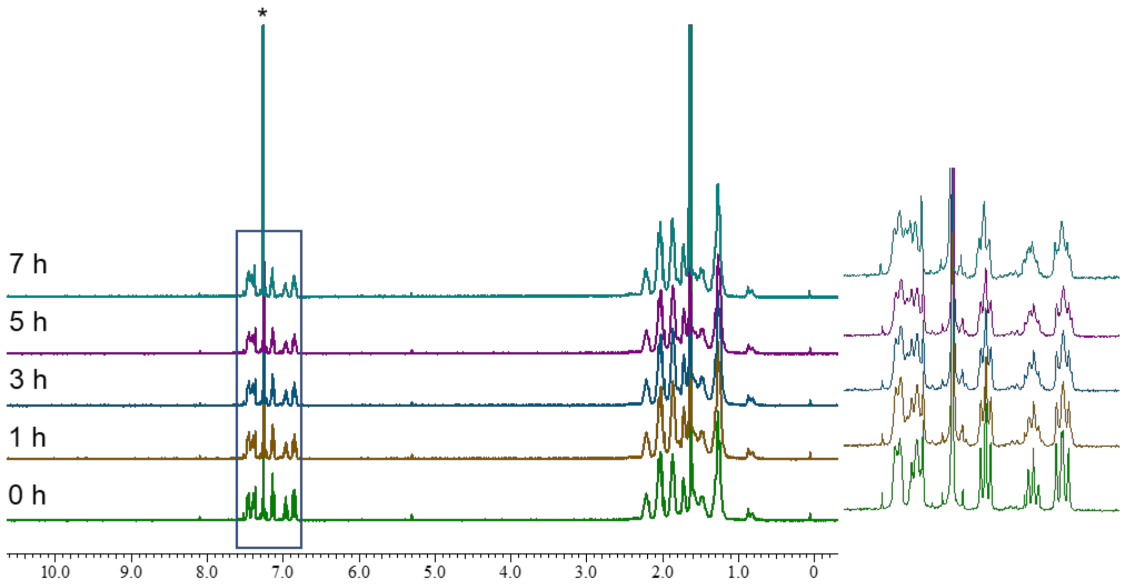

Figure S53. ^{1}H NMR spectra of the reaction mixture of expetiment for entry 11 in Table S4 (400 MHz, Acetone-d_6, 0 °C).

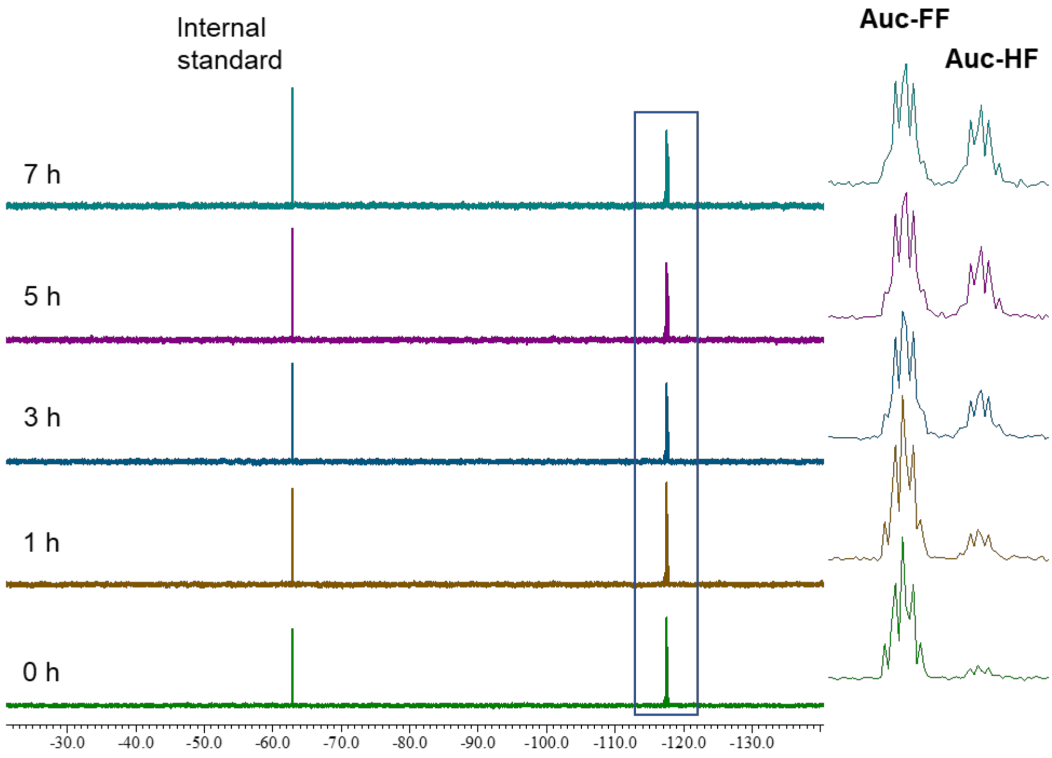

Figure S54. ^{19}F NMR spectra of the reaction mixture of expetiment for entry 11 in Table S4 (375 MHz, Acetone-d_6, 0 °C).

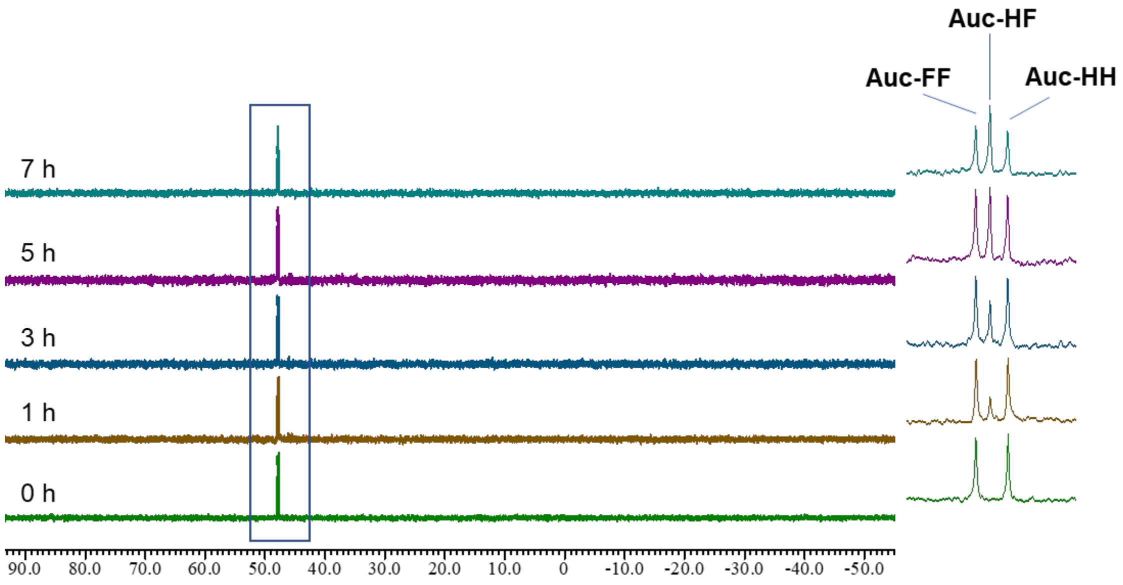

Figure S55. $^{31}P\{^1H\}$ NMR spectra of the reaction mixture of expetiment for entry 11 in Table S4 (161 MHz, Acetone-d_6, 0 °C).

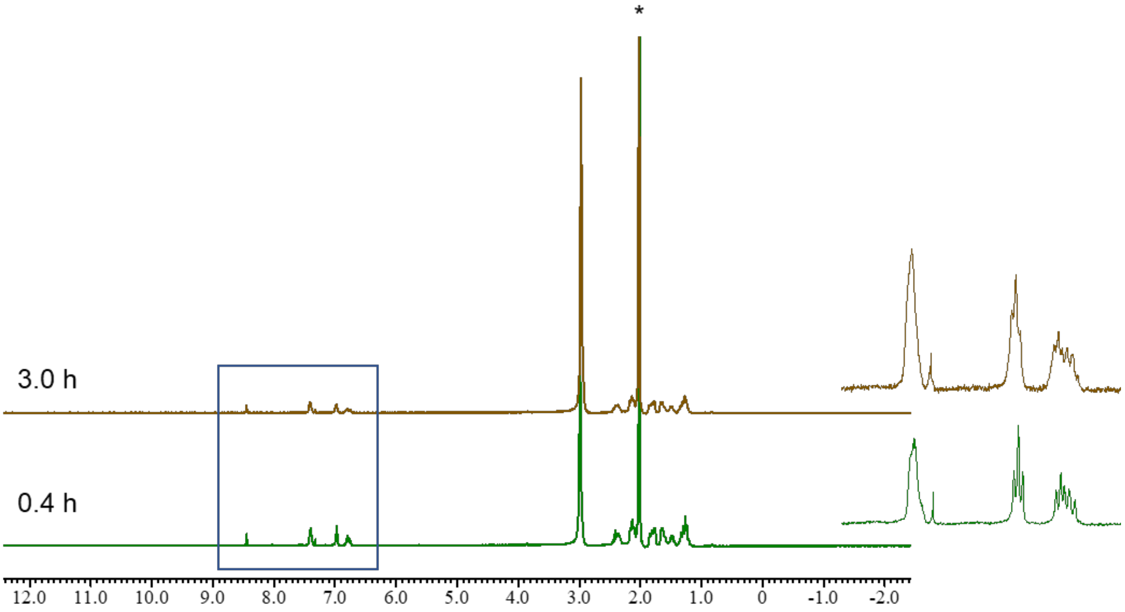

Figure S56. ^{1}H NMR spectra of the reaction mixture of expetiment for entry 13 in Table S4 (400 MHz, DMF-d_7, 0 °C).

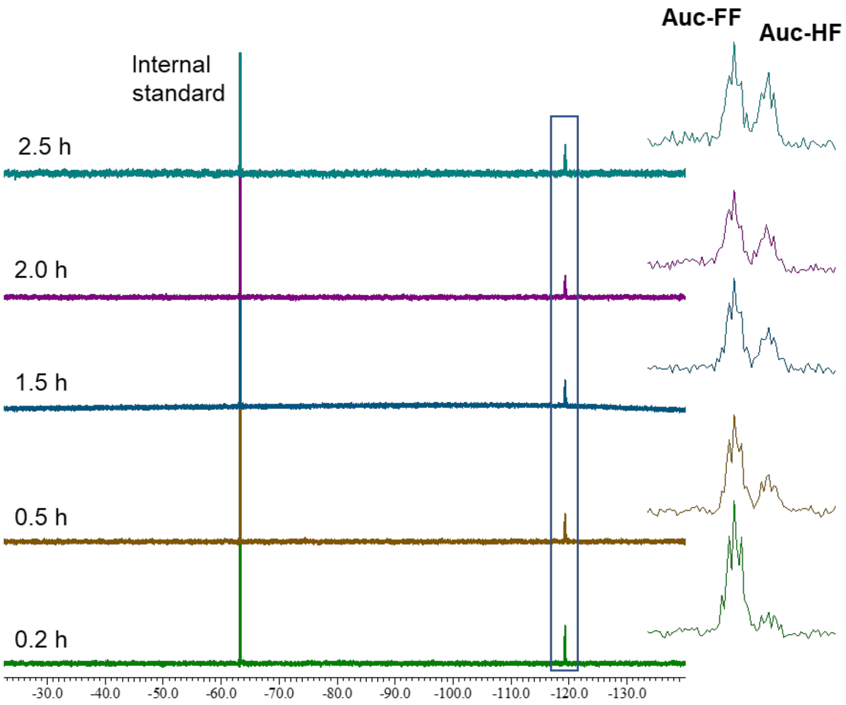

Figure S57. ^{19}F NMR spectra of the reaction mixture of expetiment for entry 3 in Table S4 (375 MHz, DMF-d_7, 0 °C).

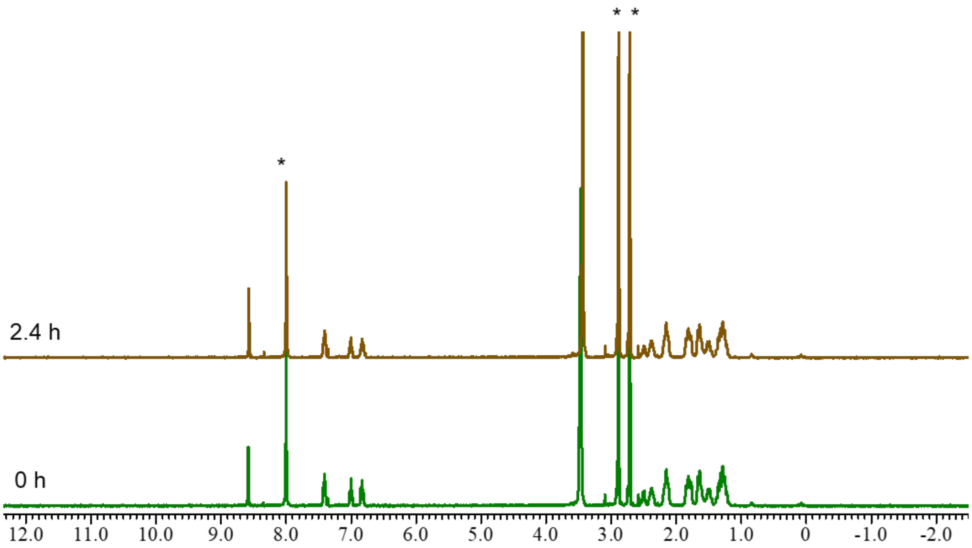

Figure S58. ^{1}H NMR spectra of the reaction mixture of expetiment for entry 15 in Table S4 (400 MHz, DMF-d_7, 0 °C).

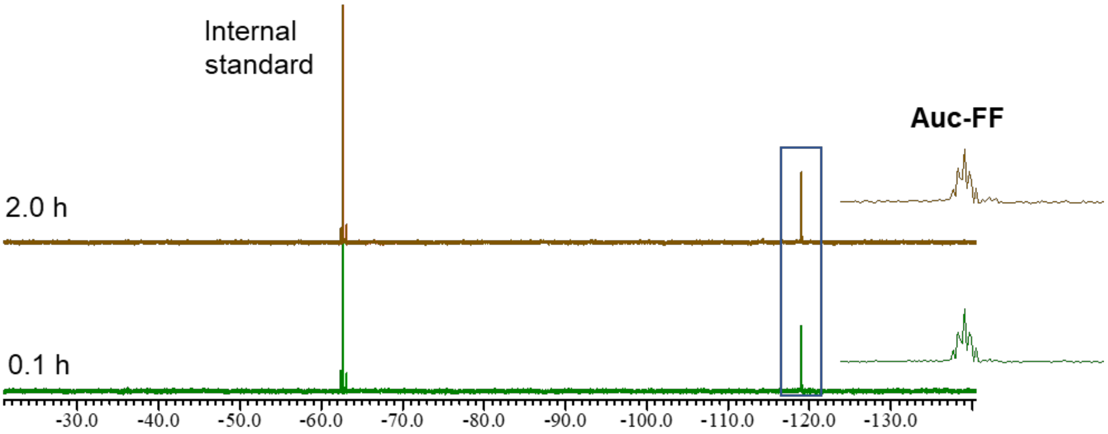

Figure S59. ^{19}F NMR spectra of the reaction mixture of expetiment for entry 15 in Table S4 (375 MHz, DMF-d_7, 0 °C).

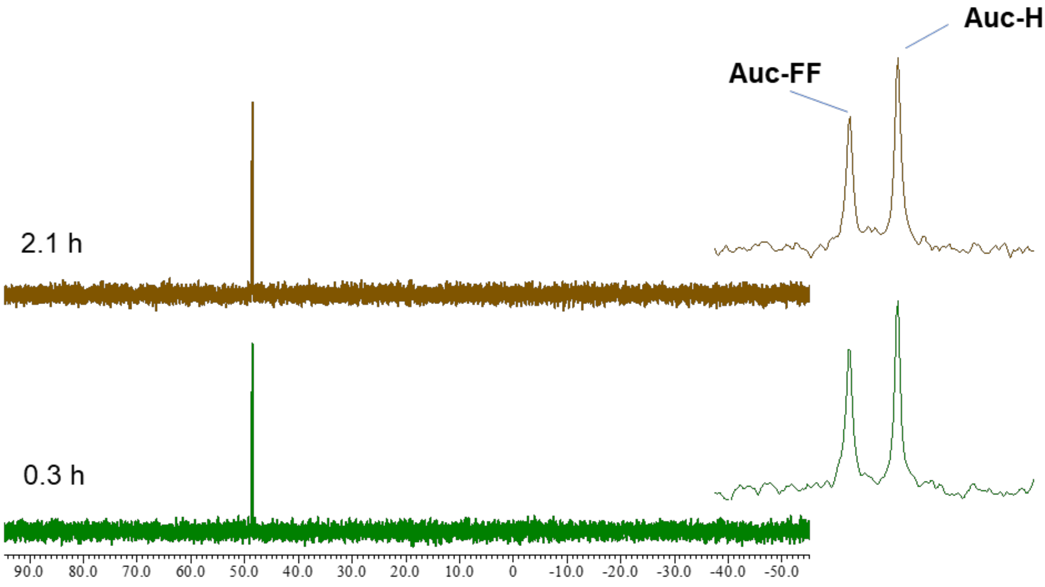

Figure S60. $^{31}P\{^{1}H\}$ NMR spectra of the reaction mixture of expetiment for entry 15 in Table S4 (161 MHz, DMF-d_7, 0 ^{o}C).

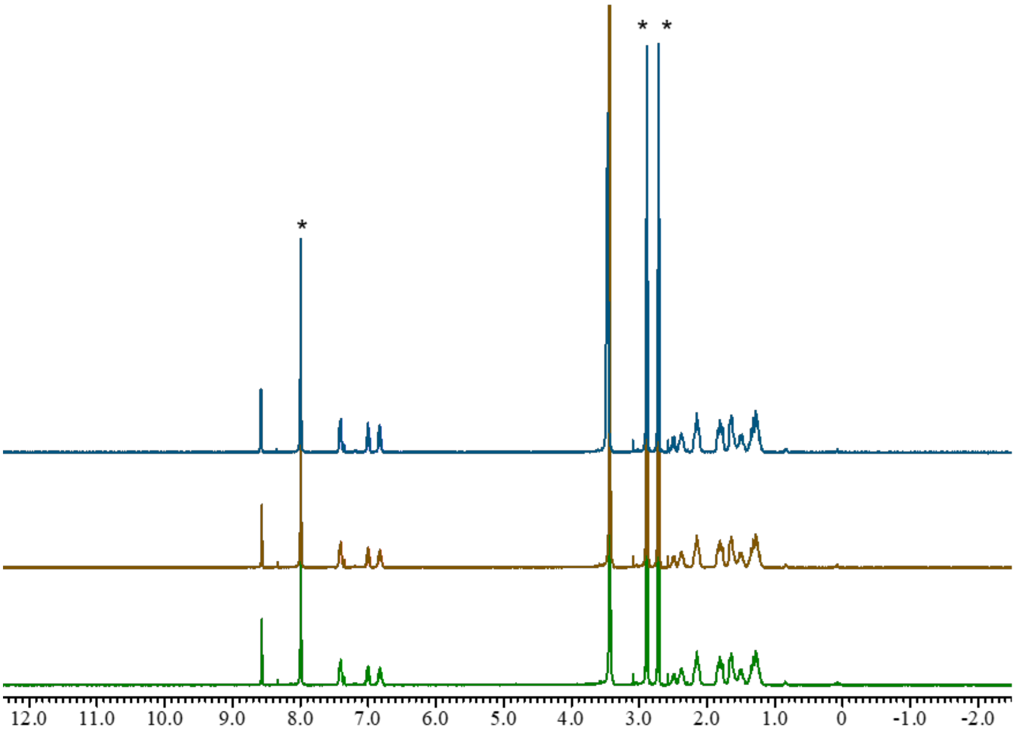

Figure S61. ^{1}H NMR spectra of the reaction mixture of expetiment for entry 17 in Table S4 (400 MHz, DMF-d_7, 25 ^{o}C).

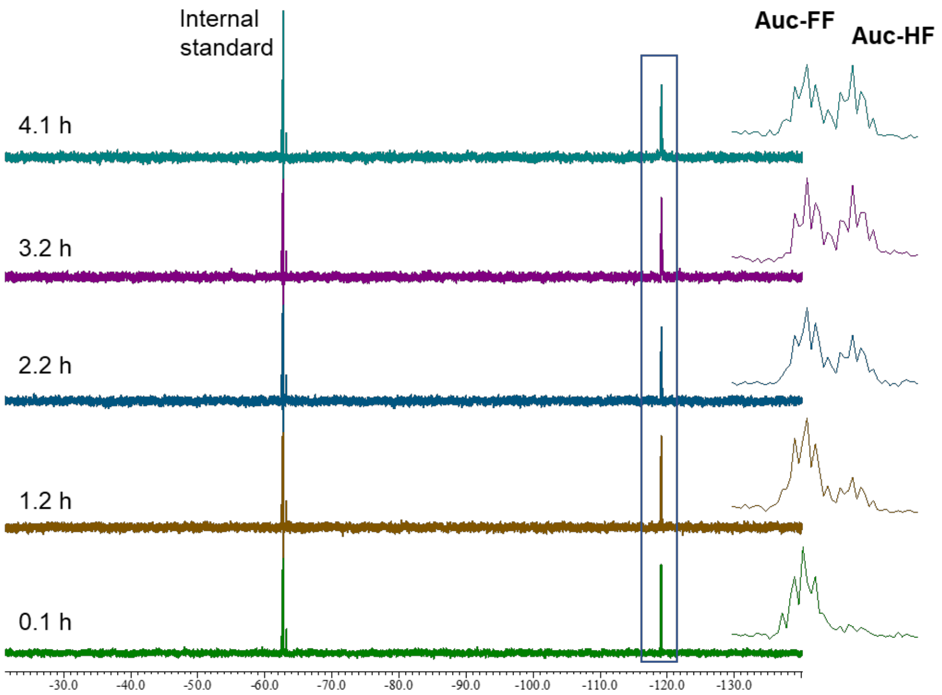

Figure S62. ^{19}F NMR spectra of the reaction mixture of expetiment for entry 17 in Table S4 (375 MHz, DMF-d_7, 25 °C).

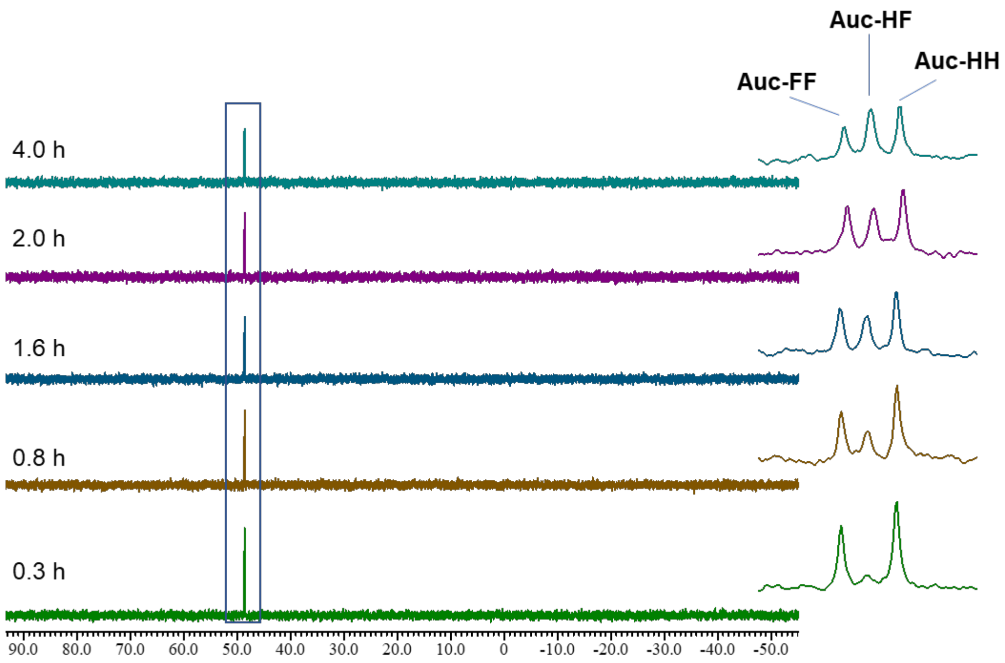

Figure S63. $^{31}P\{^{1}H\}$ NMR spectra of the reaction mixture of expetiment for entry 17 in Table S4 (161 MHz, DMF-d_7, 25 °C).

Eyring–Polanyi Plot

The averaged rate constants (See Table S4) were employed into the Eyring–Polanyi plot[S18] ($\ln(k_1 T^{-1}$ /$M^{-1}s^{-1}K^{-1}$) vs. T^{-1} /K) to calculate thermodynamic parameters (Figure S64).

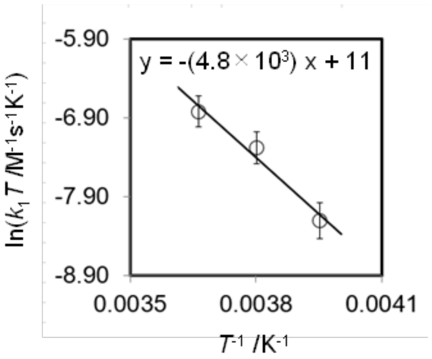

Thermodynamic parameters by Eyring–Polanyi plot		
$\Delta G^{\ddagger}$ /kcal·mol^{-1}	17	(at 25 °C)
$\Delta H^{\ddagger}$ /kcal·mol^{-1}	9.5	
$\Delta S^{\ddagger}$ /cal·mol^{-1}·K^{-1}	-26	

Figure S64. Eyring–Polanyi plot and calculated thermodynamic parameters.

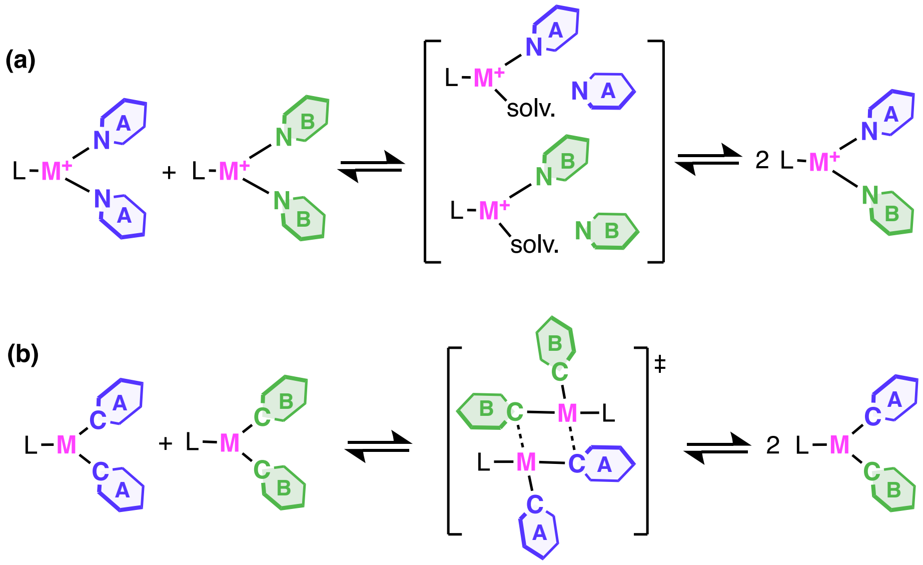

Scheme S3. Intermolecular ligand exchange reaction. (a) reversible bond-cleavage and -formation processes of M–N bonds. (b) homonuclear transmetalation processes of M–C σ bonds (M = Pd, Pt).

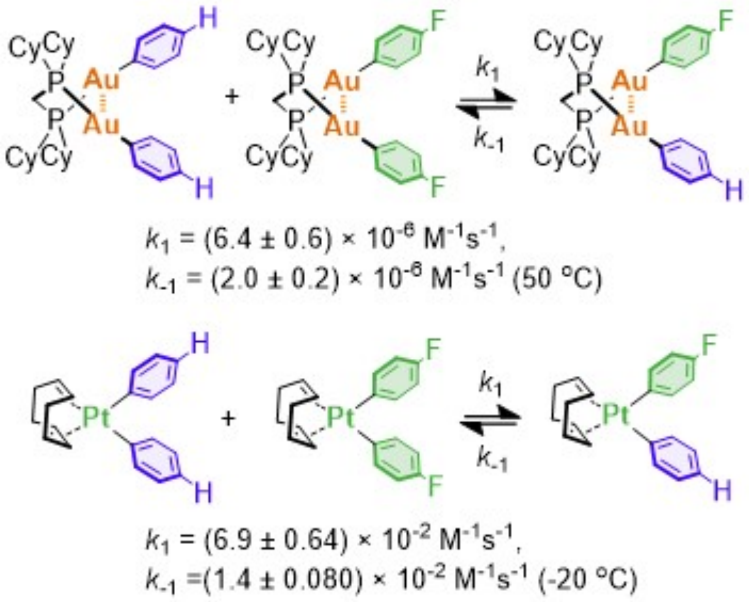

Scheme S4. Intermolecular aryl ligand exchange reaction of Au_2Ph_2(dcpm) and $Au_2(C_6H_4$-4-F$)_2$(dcpm), and the comparable similar reaction of $PtPh_2$(cod) and Pt(C_6H_4-4-F$)_2$(cod).[S19]

Preparation of a mixture of Au$_C$-HH, Au$_C$-DD and [$Au_2Ph(C_6D_5)(dcpm)$] (Au$_C$-HD), followed by the chlorination and reductive elimination to yield biphenyls.

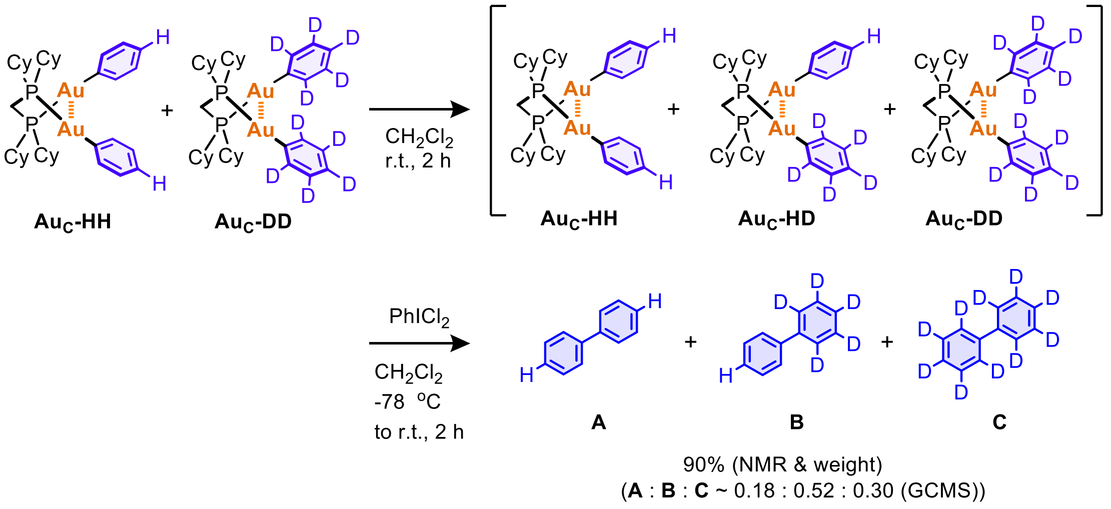

A CH_2Cl_2 (1 mL) solution of acyclic Au complexes, **Au$_C$-HH** (5.0 mg, 5.0 μmol) and **Au$_C$-DD** (5.0 mg, 5.0 μmol), was stirred at room temperature for 2 h. The resulting solution was cooled to -78 °C, and added $PhICl_2$ (6.3 mmol/L in CH_2Cl_2, 1.67 mL, 10 μmol) dropwise with stirring at –78 °C for 15 min. The reaction mixture was stirred at the same temperature for 30 min, then allowed to warm to room temperature, and stirred for 2 h. Solvent and iodobenzene (by-product) were removed under vacuum. Residual Au complexes was removed filtration through a short pad of florisil eluted by MTBE/CH_2Cl_2 (1:1). After removing a solvent, the residue (1.43 mg, 90%) was subjected into NMR experiments and GCMS analysis. The ratio of biphenyls, C_6H_5-C_6H_5 (**A**), C_6H_5-C_6D_5 (**B**) and C_6D_5-C_6D_5 (**C**), was estimated by a comparison of MS pattern with a simulation of isotope distributions of a mixture (**A** : **B** : **C** ~ 0.18 : 0.52 : 0.30) (Figure S65).

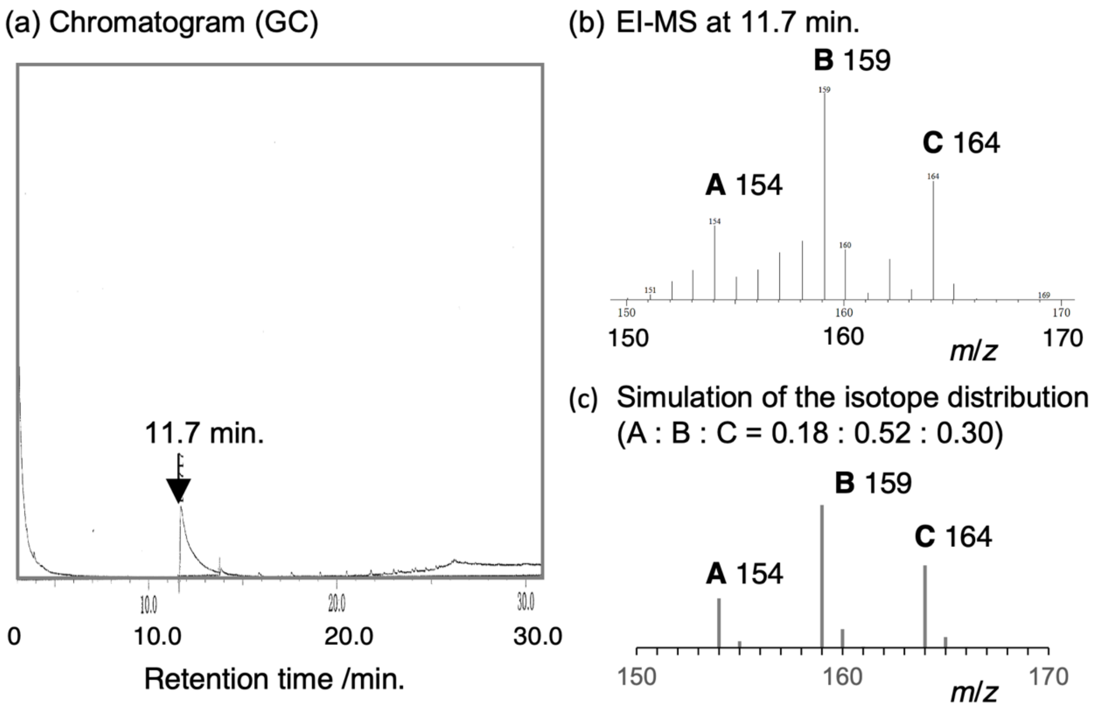

Figure S65. GCMS analysis of a resulting mixture containing **A**, **B**, and **C**: (a) A chromatogram chart of GCMS analysis; (b) A mass spectrum of an outflow mixture around 11.7 min. of retention time of gas chromatogram; (c) A simulated isotope distribution pattern of the mixture composed of **A**, **B**, and **C** in the ratio of 0.18 : 0.52 : 0.30, which is almost in good agreement with the experimental pattern.

S4. Synthesis of CPPs from two different oligophenylene linkers.

Reorganization approach (Method A) in Au-3/Au-4 system.

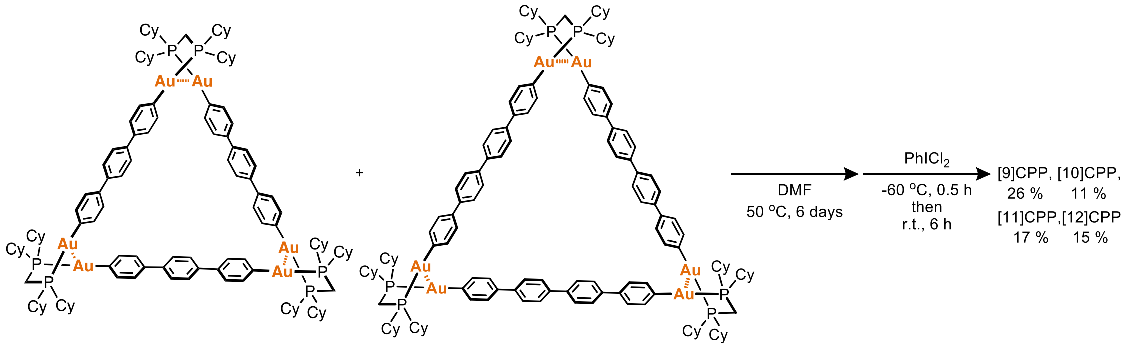

To a suspension of **Au-3** (31.1 mg, 10 µmol) and **Au-4** (33.5 mg, 10 µmol) in degassed DMF (15 mL) was stirred for 6 days at 50 °C under an argon atmosphere. After the reaction mixture allowed to cool to –60 °C, was added $PhICl_2$ (6.0 mmol/L in DMF, 10 mL, 60 µmol) dropwise with stirring at the same temperature for 5 min. The reaction mixture was stirred at the same temperature for 30 min, then it was allowed to warm to 25 °C and stirred for 6 h. Solvent and iodobenzene (by-product) were removed under vacuum. The crude product was purified by silica gel column chromatography (eluent; $CHCl_3$) to give a mixture of [*n*]CPPs as a yellow solid (12.2 mg). ^{1}H NMR analysis indicated the formation of [9]-, [10]-, [11]-, and [12]CPPs in 26%, 11%, 17% and 15% yields over 2 steps, respectively (See Figure S66 and Table S5).

The mixture of [n]CPPs (12.2 mg) was purified by preparative gel permeation chromatography (eluent; $CHCl_3$) giving [9]CPP (3.62 mg, 5.3 µmol, 26%), [10]CPP (1.65 mg, 2.2 µmol, 11%), [11]CPP (3.02 mg, 3.6 µmol, 18%), and [12]CPP (2.35 mg, 2.57 µmol, 13%) in pure forms (See Figure S69).

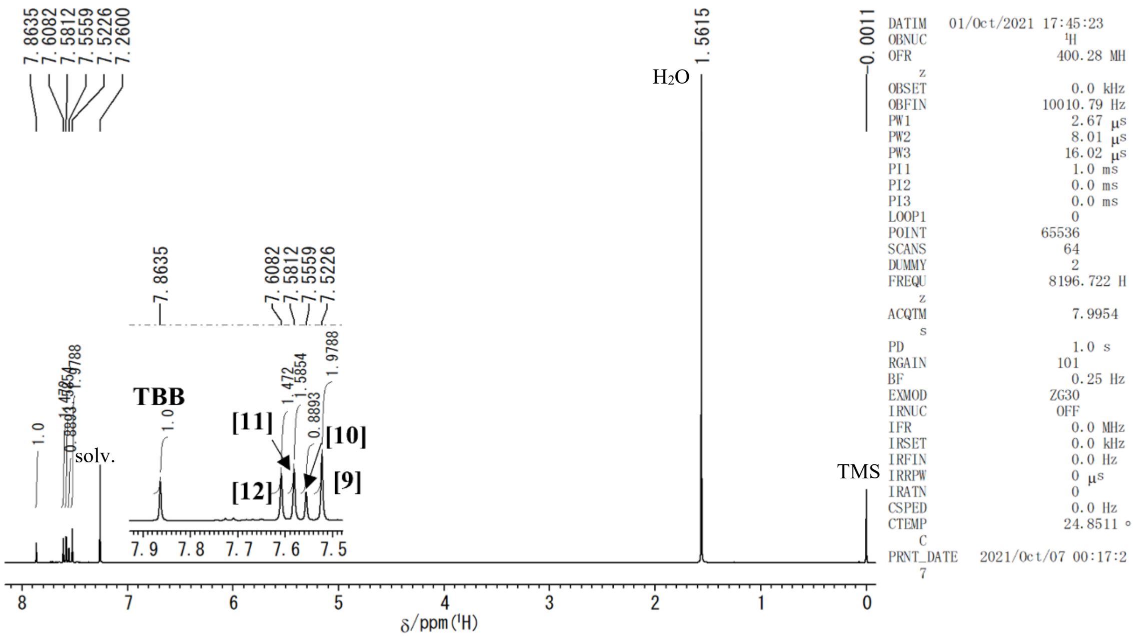

Figure S66. ^{1}H NMR spectrum of the CPP products after purification with internal standard (400 MHz, $CDCl_3$, r.t.).

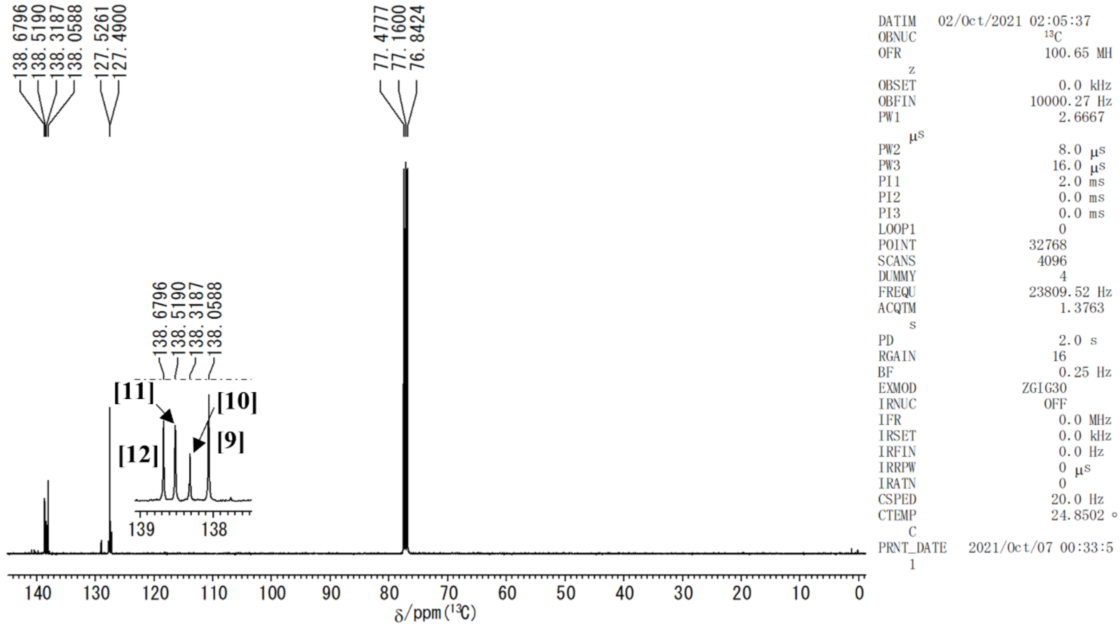

Figure S67. $^{13}C\{^{1}H\}$ NMR spectrum of the CPP products after purification (101 MHz, $CDCl_3$, r.t.).

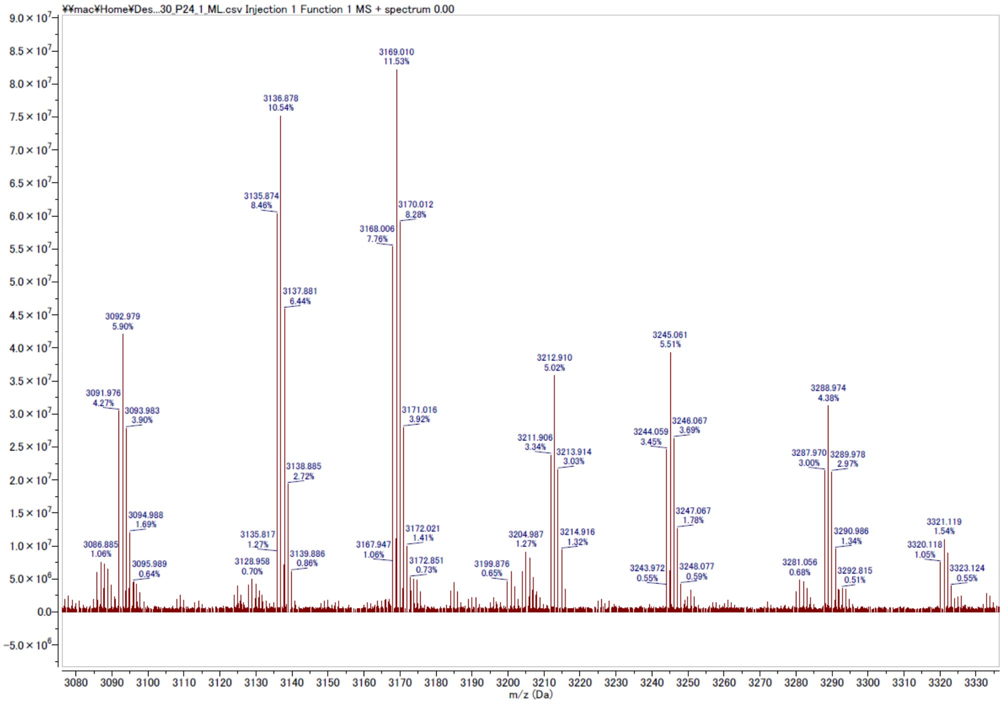

Figure S68. FT-ICR MALDI-TOF MS spectrum of the mixture of different macrocyclic Au complexes, **Au-3** and **Au-4** after mixing at 50 °C in DMF (X = C_2H_6O or Me_2NH).

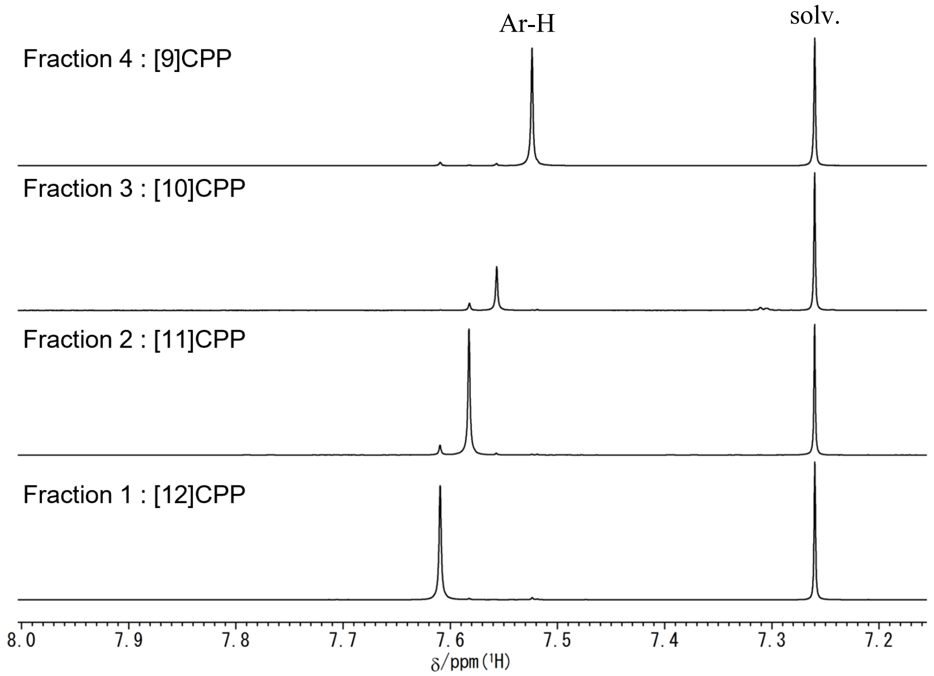

Figure S69. Stacked ^{1}H NMR spectra of [*n*]CPP (*n* = 9, 10, 11, 12) separated from the reaction mixture by PGPC (400 MHz, $CDCl_3$, r.t.).

Table S5. Calculation of NMR yields of CPPs (Method A) in **Au-3**/**Au-4** system

(**A**)	Total amounts of CPP mixtures after oxidative chlorination	12.2 mg
(**B**)	Amounts of **A** contained in the NMR tube	0.73 mg
(**C**)	Amounts of internal standard (TBB) contained in the NMR tube	1.14 mg (2.90 μmol)
(**D**)	NMR signal intensities<br>v.s. TBB (See Figure S66)	[9]CPP: 1.98<br>[10]CPP: 0.889<br>[11]CPP: 1.59<br>[12]CPP: 1.47
(**E**)	Ratio of number of protons in the structure v.s. TBB	[9]CPP: 18<br>[10]CPP: 20<br>[11]CPP: 22<br>[12]CPP: 24
(**F**)	Molar numbers of CPPs in **B**<br>(**C** × **D** ÷ **E**)	[9]CPP: 318 nmol<br>[10]CPP: 129 nmol<br>[11]CPP: 209 nmol<br>[12]CPP: 178 nmol
(**G**)	Molar numbers of CPPs in **A**<br>(**F** × **A** ÷ **B**)	[9]CPP: 5.32 μmol<br>[10]CPP: 2.15 μmol<br>[11]CPP: 3.49 μmol<br>[12]CPP: 2.97 μmol
(**H**)	Theoretical yield	20.1 μmol
(**I**)	NMR yield<br>(**G** ÷ **H** × 100)	**[9]CPP: 26%**<br>**[10]CPP: 11%**<br>**[11]CPP: 17%**<br>**[12]CPP: 15%**

Reorganization approach (Method A) in Au-3/Au-4 system <u>without heating process</u>.

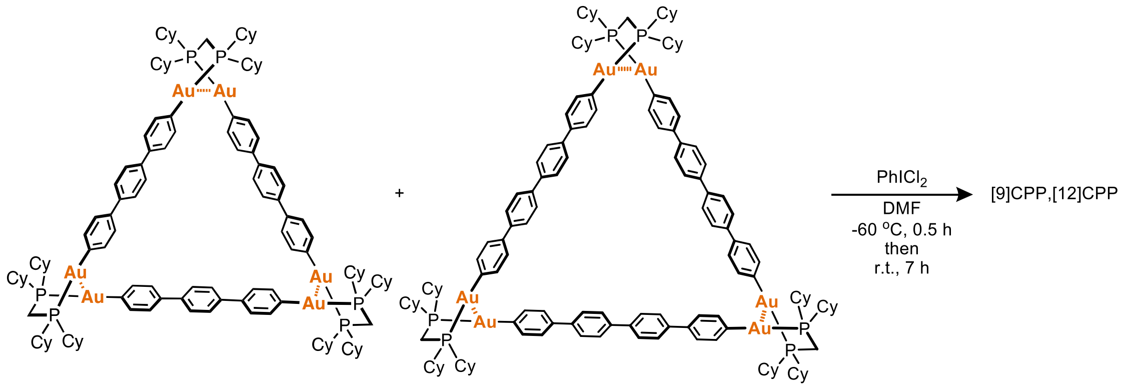

To a suspension of **Au-3** (15.8 mg, 5.1 μmol) and **Au-4** (16.7 mg, 5.0 μmol) in degassed DMF (15 mL) was added $PhICl_2$ (6.3 mmol/L in DMF, 5 mL, 32 μmol) dropwise with stirring at –60 °C for 5 min. The reaction mixture was stirred at the same temperature for 30 min, then it was allowed to warm to 25 °C and stirred for 7 h. Solvent and iodobenzene (by-product) were removed under vacuum. The crude product was obtained as a yellow solid.

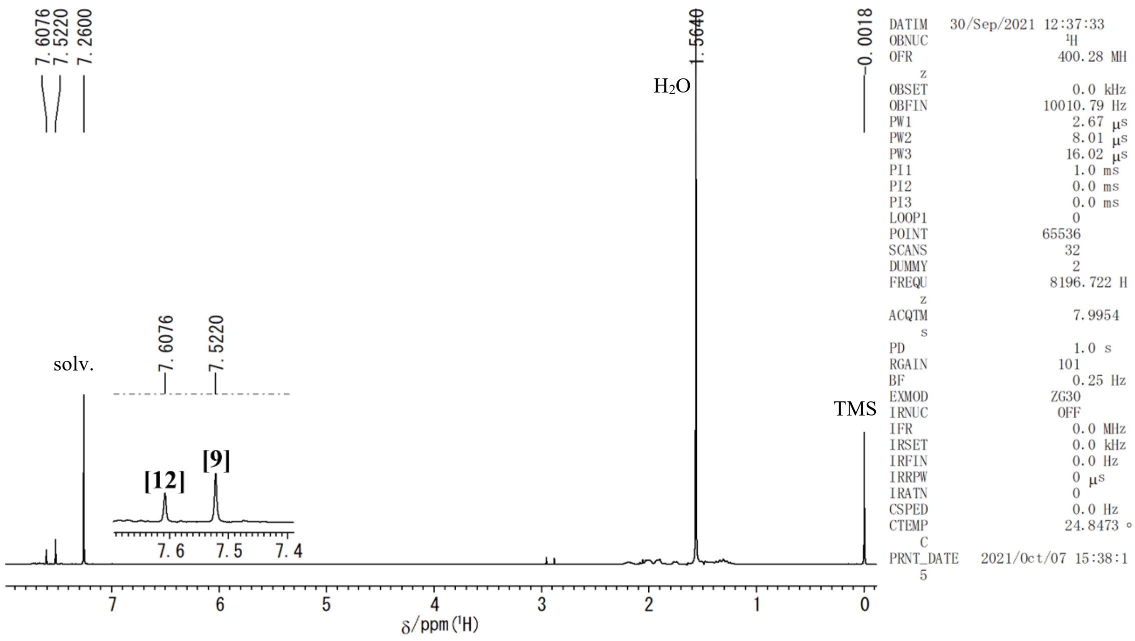

Figure S70. ¹H NMR spectrum of the crude products (400 MHz, $CDCl_3$, r.t.).

Reorganization approach (Method A) in Au-4/Au-5 system.

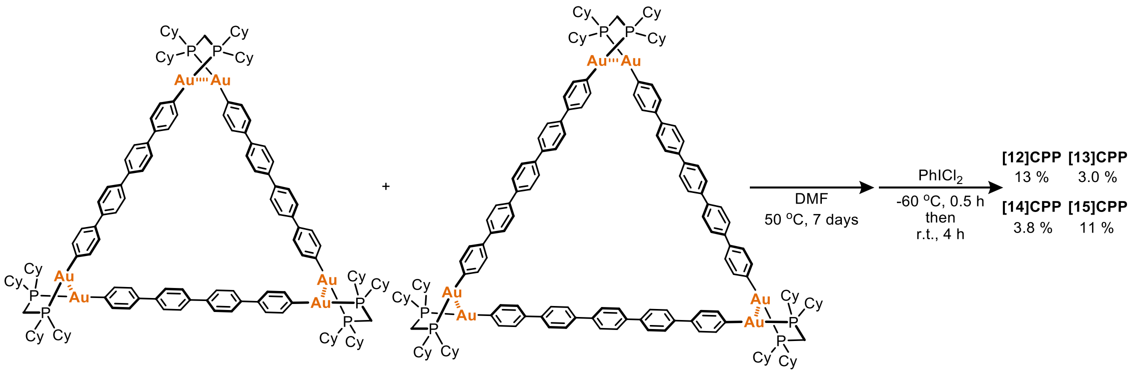

To a suspension of **Au-4** (17.0 mg, 5.1 μmol) and **Au-5** (18.3 mg, 5.1 μmol) in degassed DMF (15 mL) was stirred for 7 days at 50 ºC under an argon atmosphere. After the reaction mixture allowed to cool to –60 ºC , was added $PhICl_2$ (6.3 mmol/L in DMF, 5 mL, 32 μmol) dropwise with stirring at the same temperature for 5 min. The reaction mixture was stirred at the same temperature for 30 min, then it was allowed to warm to 25 ºC and stirred for 4 h. Solvent and iodobenzene (by-product) were removed under vacuum. The crude product was purified by silica gel column chromatography (eluent; $CHCl_3$) to give a mixture of CPPs as a pale yellow solid (4.4 mg). ^{1}H NMR analysis indicated the formation of [12]-, [13]-, [14]-, and [15]CPPs in 13%, 3.0%, 3.8% and 11% yields over 2 steps, respectively.

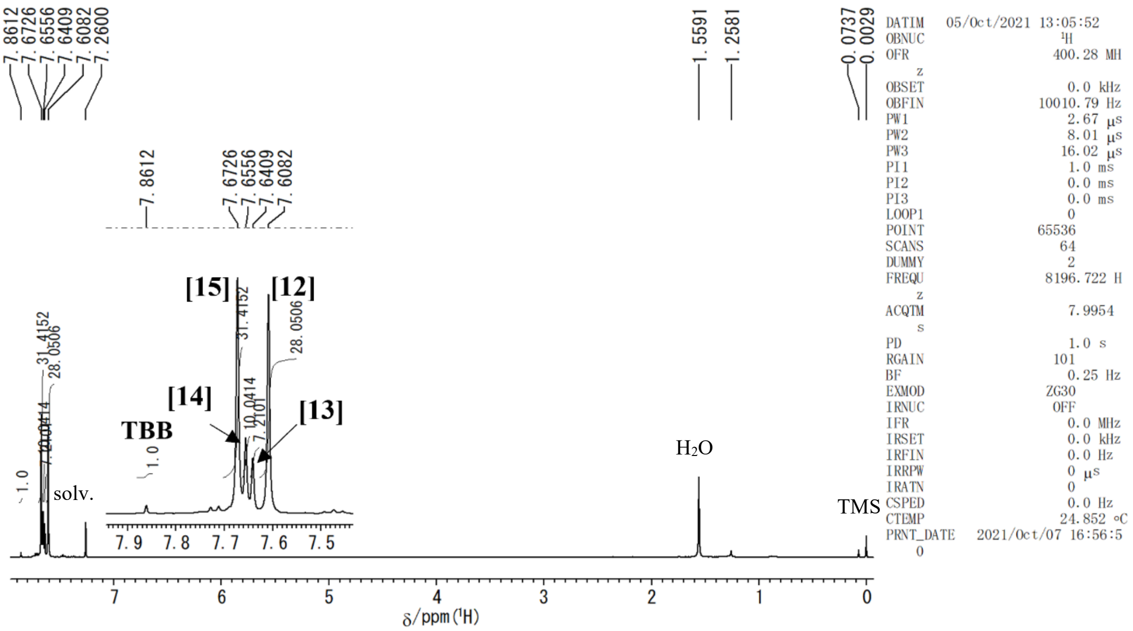

Figure S71. ^{1}H NMR spectrum of the CPP products after purification with internal standard (400 MHz, $CDCl_3$, r.t.).

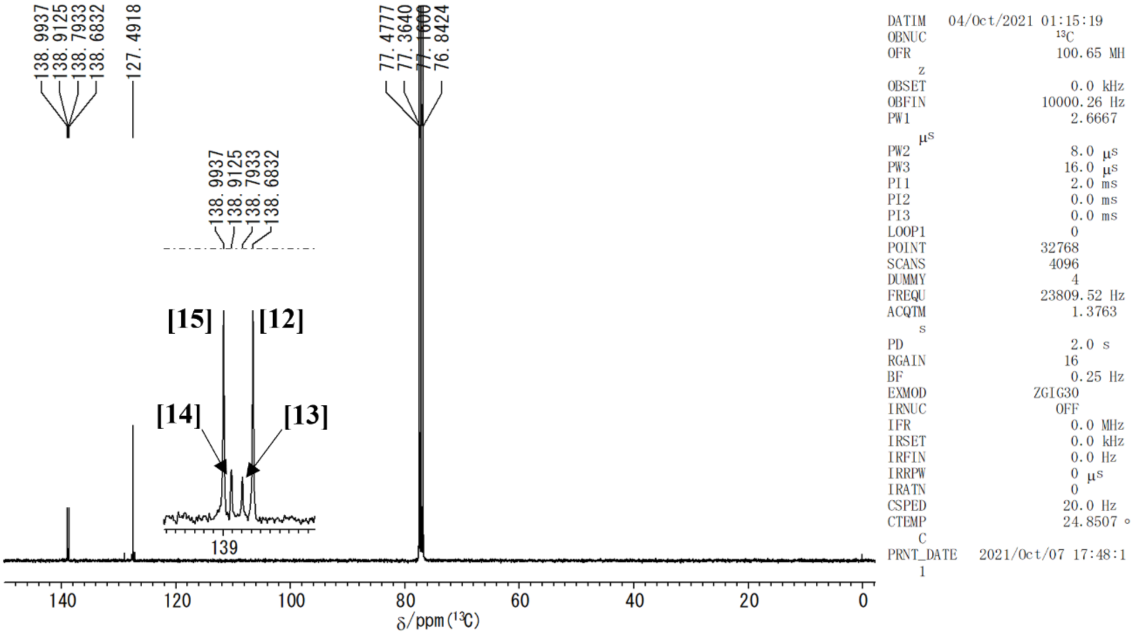

Figure S72. $^{13}C\{^{1}H\}$ NMR spectrum of the CPP products after purification (101 MHz, $CDCl_3$, r.t.).

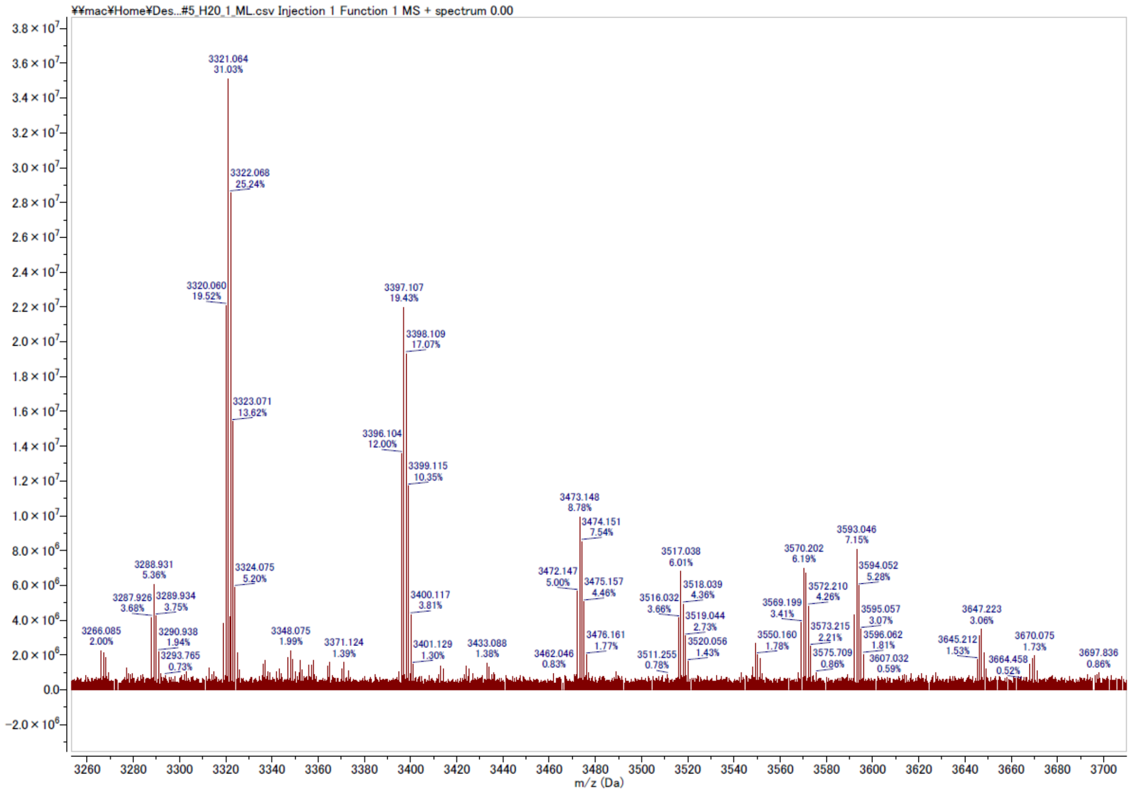

Figure S73. FT-ICR MALDI-TOF MS spectrum of the mixture of different macrocyclic Au complexes, **Au-4** and **Au-5** after mixing at 50 ºC in DMF (X = C_2H_6O or Me_2NH).

Table S6. Calculation of NMR yields of CPPs (Method A) in **Au-4**/**Au-5** system

(**A**)	Total amounts of CPP mixtures after oxidative chlorination	4.4 mg
(**B**)	Amounts of **A** contained in the NMR tube	4.4 mg
(**C**)	Amounts of internal standard (TBB) contained in the NMR tube	0.43 mg (1.09 μmol)
(**D**)	NMR signal intensities v.s. TBB (See Figure S71)	[12]CPP: 28.1<br>[13]CPP: 7.21<br>[14]CPP: 10.0<br>[15]CPP: 31.4
(**E**)	Ratio of number of protons in the structure v.s. TBB	[12]CPP: 24<br>[13]CPP: 26<br>[14]CPP: 28<br>[15]CPP: 30
(**F**)	Molar numbers of CPPs in **B**<br>(**C** × **D** ÷ **E**)	[12]CPP: 1.28 μmol<br>[13]CPP: 0.303 μmol<br>[14]CPP: 0.392 μmol<br>[15]CPP: 1.14 μmol
(**G**)	Molar numbers of CPPs in **A**<br>(**F** × **A** ÷ **B**)	[12]CPP: 1.28 μmol<br>[13]CPP: 0.303 μmol<br>[14]CPP: 0.392 μmol<br>[15]CPP: 1.14 μmol
(**H**)	Theoretical yield	10.2 μmol
(**I**)	NMR yield<br>(**G** ÷ **H** × 100)	**[12]CPP: 13%**<br>**[13]CPP: 3.0%**<br>**[14]CPP: 3.8%**<br>**[15]CPP: 11%**

Reorganization approach (Method A) in Au-4/Au-5 system <u>without heating process</u>.

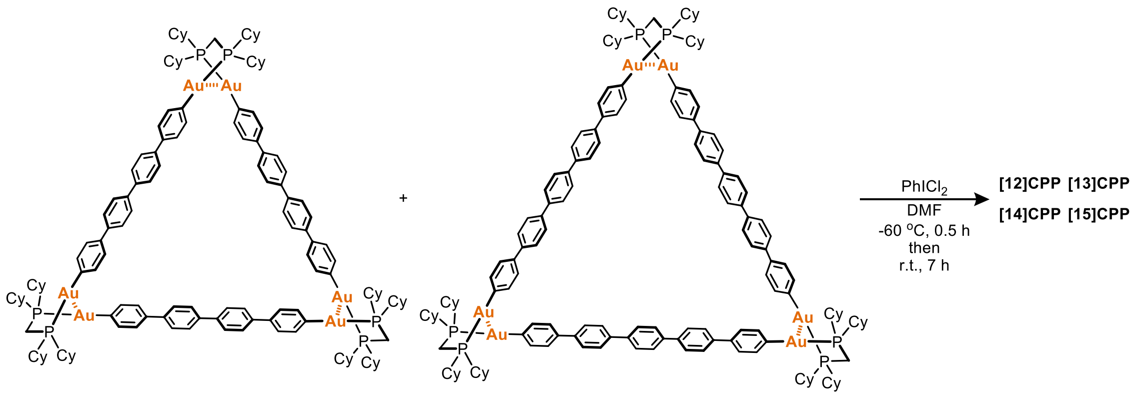

To a suspension of **Au-4** (16.8 mg, 5.0 μmol) and **Au-5** (18.2 mg, 5.1 μmol) in degassed DMF (15 mL) was added $PhICl_2$ (6.3 mmol/L in DMF, 5 mL, 32 μmol) dropwise with stirring at –60 °C for 5 min. The reaction mixture was stirred at the same temperature for 30 min, then it was allowed to warm to 25 °C and stirred for 7 h. Solvent and iodobenzene (by-product) were removed under vacuum. The crude product was obtained as a gray solid.

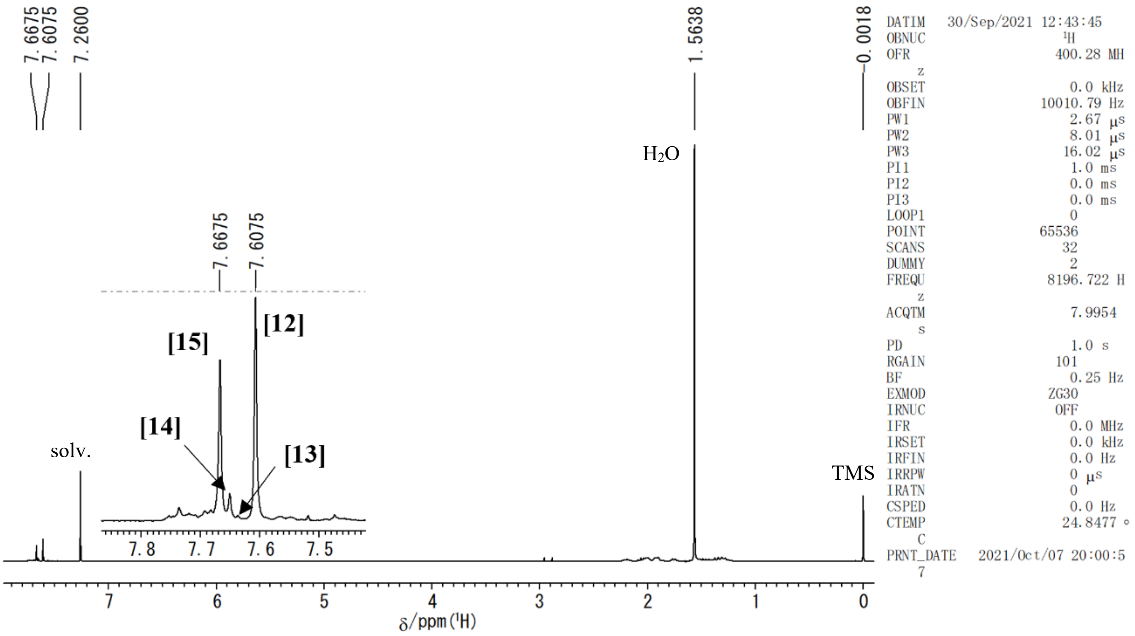

Figure S74. ^{1}H NMR spectrum of the crude products (400 MHz, $CDCl_3$, r.t.).

Social self-sorting approach (Method B) in L3/L4 system.

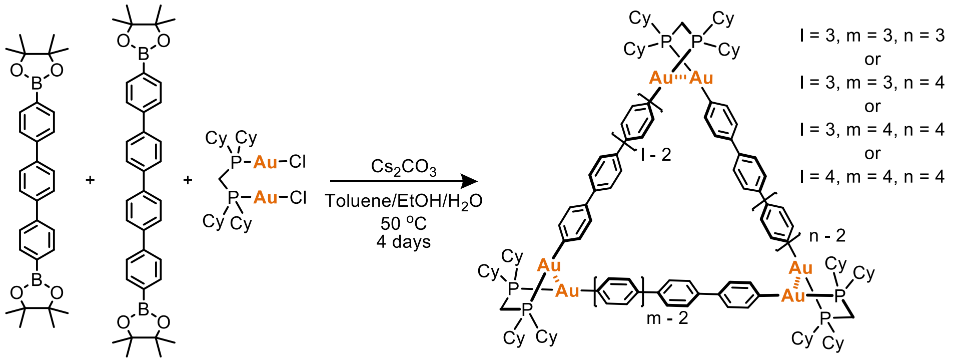

A mixture of 4,4''-*p*-terphenyldiboronic acid pinacol ester (**L3**) (42.4 mg, 0.088 mmol), 4,4'''-*p*-quaterphenyldiboronic acid pinacol ester (**L4**) (49.0 mg, 0.088 mmol), Cs_2CO_3 (369 mg, 1.1 mmol) and [Au_2Cl_2(dcpm)] (**1**) (158 mg, 0.18 mmol) in degassed toluene/H_2O/EtOH (8 mL/2 mL/2 mL) was stirred for 4 days at 50 °C under an argon atmosphere. After the reaction mixture allowed to cool to room temperature, the precipitate was collected by suction filtration and washed with toluene (10 mL), H_2O (10 mL), EtOH (10 mL), then dried in *vacuo*. The mixture of Au complexes (120 mg) was obtained as a dark-green solid, which was used in the next reaction without further purification.

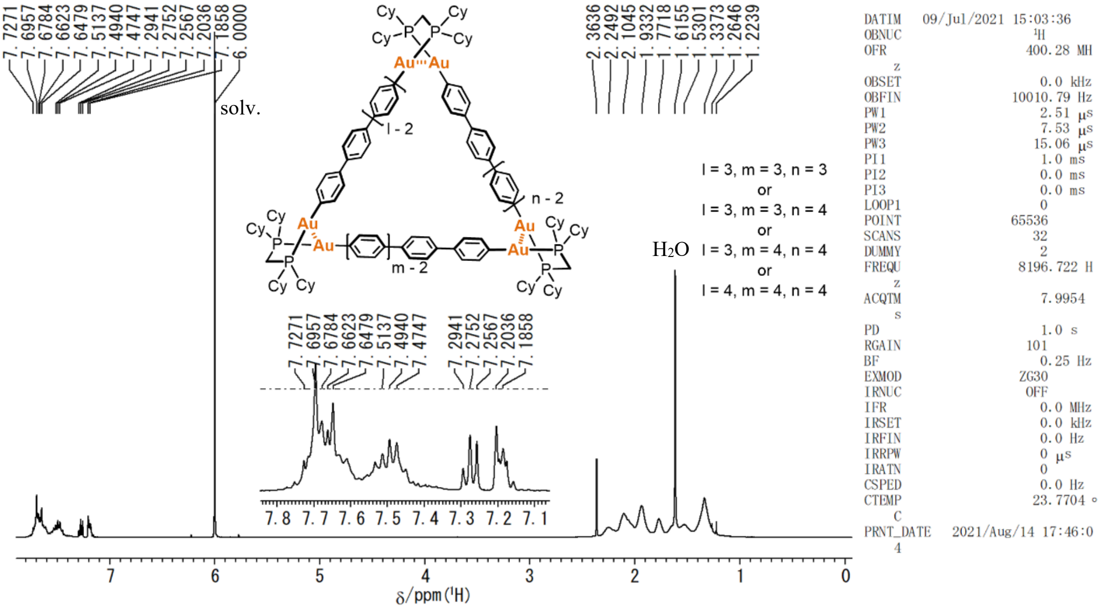

Figure S75. ^{1}H NMR spectrum of mixture of Au complexes from **L3** and **L4** (400 MHz, $C_2D_2Cl_4$, r.t.).

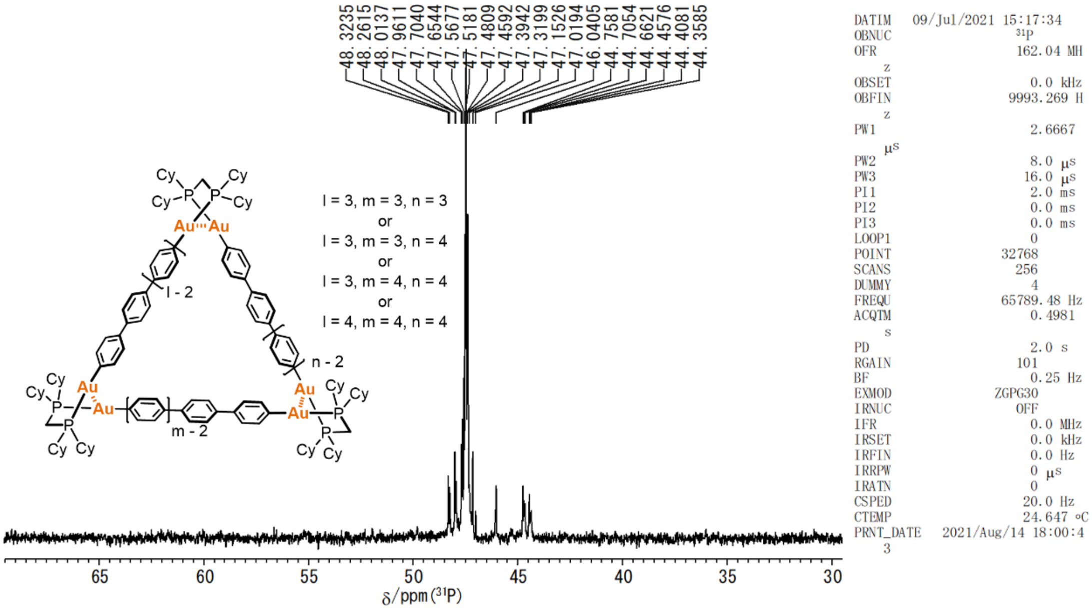

Figure S76. $^{31}P\{^{1}H\}$ NMR spectrum of mixture of Au complexes from **L3** and **L4** (162 MHz, $C_2D_2Cl_4$, r.t.).

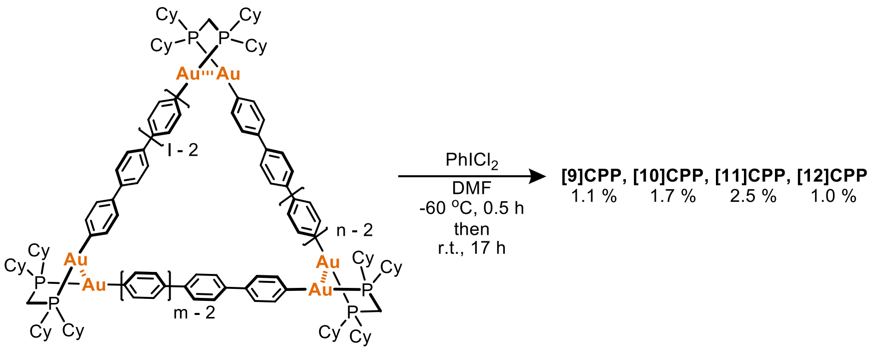

To a suspension of the mixture of Au complexes (40.2 mg) in degassed DMF (5 mL) was added $PhICl_2$ (12 mmol/L in DMF, 9 mL, 0.11 mmol) dropwise with stirring at –60 ºC for 5 min. The reaction mixture was stirred at the same temperature for 30 min, then it was allowed to warm to 25 ºC and stirred for 17 h. Solvent and iodobenzene (by-product) were removed under vacuum. The crude product was purified by silica gel column chromatography (eluent; $CHCl_3$) to give a mixture of CPPs as a yellow solid (R_f = 0.76, 0.95 mg). ^{1}H NMR analysis indicated the formation of [9]-, [10]-, [11]-, and [12]CPPs in 1.1%, 1.7%, 2.5% and 1.0% yields over 2 steps, respectively.

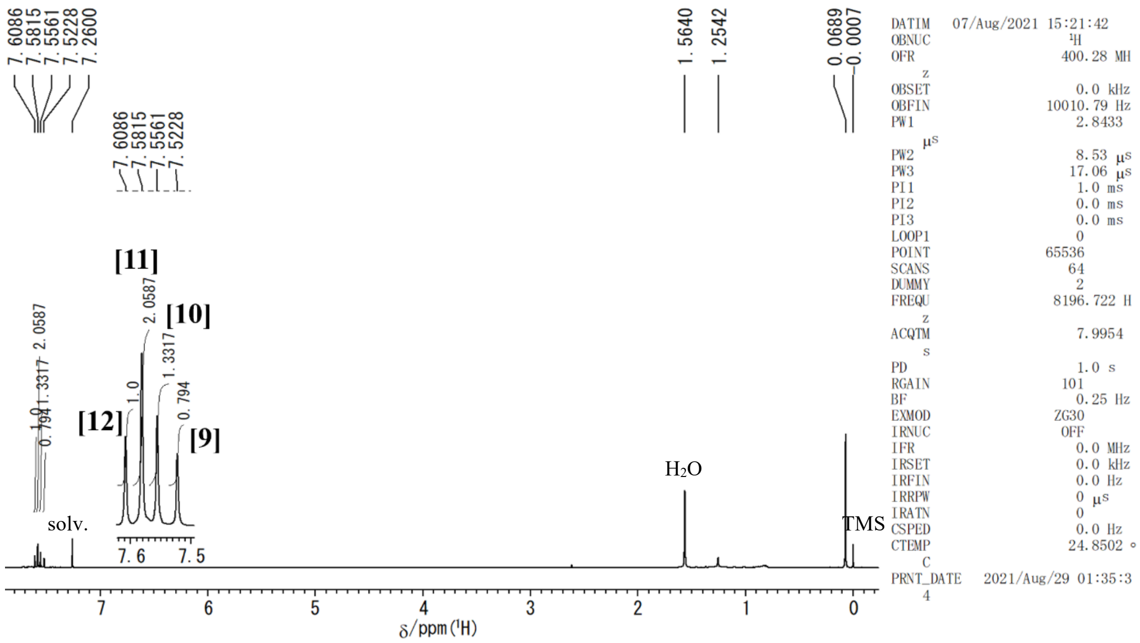

Figure S77. ^{1}H NMR spectrum of the CPP products after silica gel column chromatography (400 MHz, $CDCl_3$, r.t.).

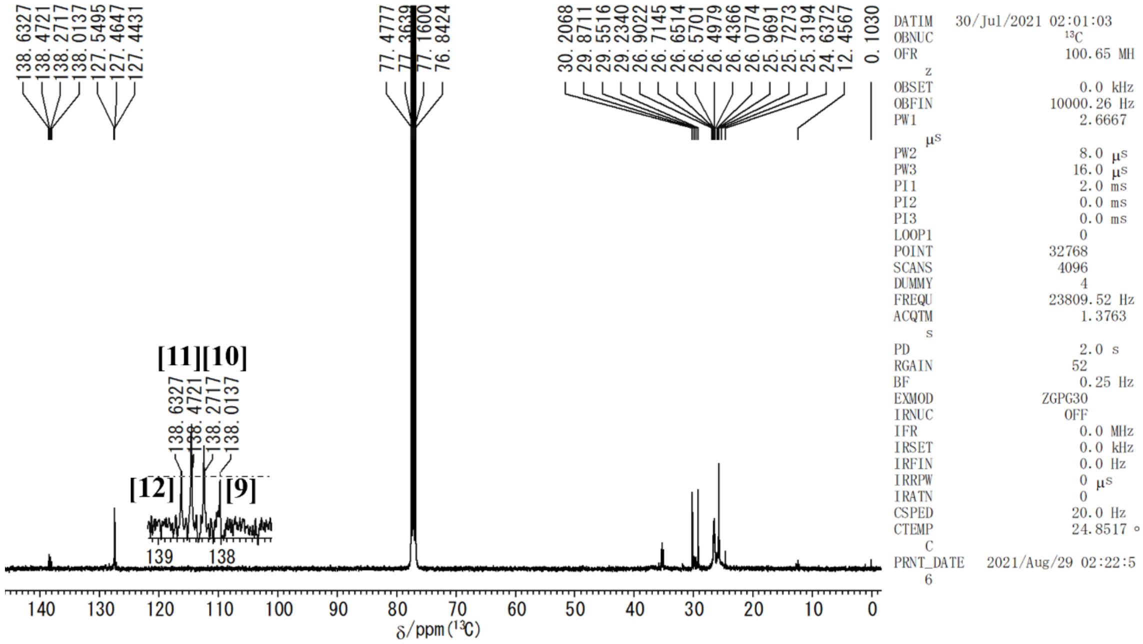

Figure S78. $^{13}C\{^{1}H\}$ NMR spectrum of the CPP products after silica gel column chromatography (101 MHz, $CDCl_3$, r.t.).

Table S7. Calculation of NMR yields of CPPs (Method B) in **L3**/**L4** system

(**A**)	Total amounts of macrocyclic gold complex after transmetalation	120 mg
(**B**)	Amounts of the gold complexes used for the oxidative chlorination	40.2 mg
(**C**)	Total amounts of CPP mixtures after oxidative chlorination	3.0 mg
(**D**)	Amounts of **C** contained in the NMR tube	1.24 mg
(**E**)	Amounts of internal standard (TBB) contained in the NMR tube	0.95 mg (2.41 μmol)
(**F**)	NMR signal intensities v.s. TBB (See Figure S77)	[9]CPP: 0.684<br>[10]CPP: 1.12<br>[11]CPP: 1.77<br>[12]CPP: 0.849
(**G**)	Ratio of number of protons in the structure v.s. TBB	[9]CPP: 18<br>[10]CPP: 20<br>[11]CPP: 22<br>[12]CPP: 24
(**H**)	Molar numbers of CPPs in **D**<br>(**E** × **F** ÷ **G**)	[9]CPP: 91.7 nmol<br>[10]CPP: 135 nmol<br>[11]CPP: 195 nmol<br>[12]CPP: 85.4 nmol
(**I**)	Molar numbers of CPPs in **C**<br>(**H** × **C** ÷ **D**)	[9]CPP: 0.222 μmol<br>[10]CPP: 0.328 μmol<br>[11]CPP: 0.471 μmol<br>[12]CPP: 0.207 μmol
(**J**)	Molar numbers of CPPs when the entire amount of the complexes is used for the oxidative chlorination<br>(**I** × **A** ÷ **B**)	[9]CPP: 0.661 μmol<br>[10]CPP: 0.977 μmol<br>[11]CPP: 1.46 μmol<br>[12]CPP: 0.616 μmol
(**K**)	Theoretical yield relative to [Au_2Cl_2(dcpm)] (**1**)<br>(181 μmol ÷ 3)	58.7 μmol
(**L**)	NMR yield over 2 steps<br>(**J** ÷ **K** × 100)	**[9]CPP: 1.1%**<br>**[10]CPP: 1.7%**<br>**[11]CPP: 2.5%**<br>**[12]CPP: 1.0%**

Social self-sorting approach (Method B) in L4/L5 system.

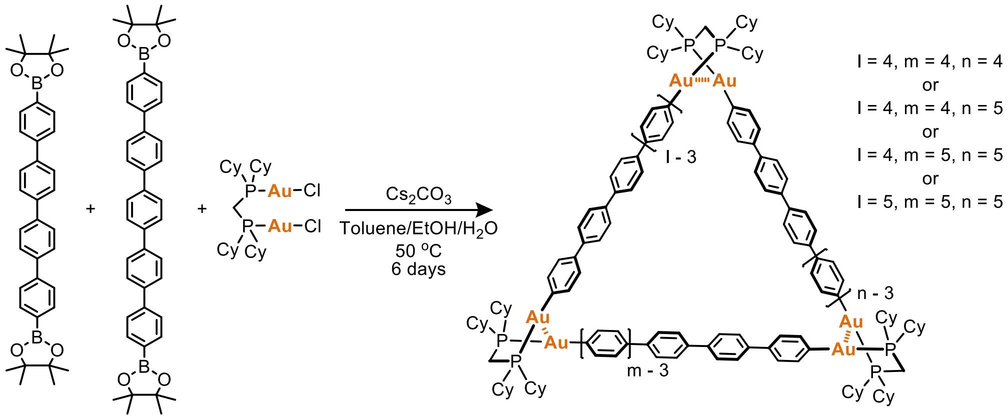

A mixture of 4,4’’’-*p*-quaterphenyldiboronic acid pinacol ester (**L4**) (55.9 mg, 0.10 mmol), 4,4’’’’-*p*-quinquephenyldiboronic acid pinacol ester (**L5**) (63.7 mg, 0.10 mmol), Cs_2CO_3 (408 mg, 1.3 mmol) and [Au_2Cl_2(dcpm)] (**1**) (176 mg, 0.20 mmol) in degassed toluene/H_2O/EtOH (8 mL/2 mL/2 mL) was stirred for 6 days at 50 °C under an argon atmosphere. After the reaction mixture allowed to cool to room temperature, the precipitate was collected by suction filtration and washed with toluene (10 mL), H_2O (10 mL), EtOH (10 mL), then dried in *vacuo*. The mixture of Au complexes (173 mg) was obtained as a white solid, which was used in the next reaction without further purification.

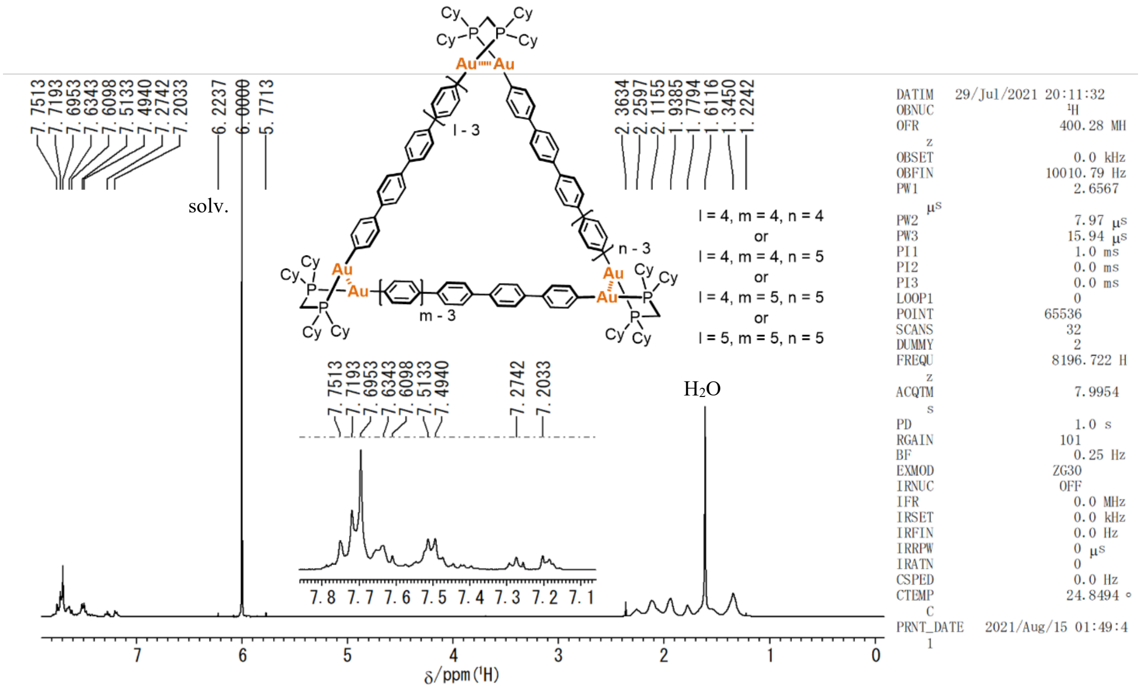

Figure S79. ^{1}H NMR spectrum of mixture of Au complexes from **L4** and **L5** (400 MHz, $CDCl_3$, r.t.).

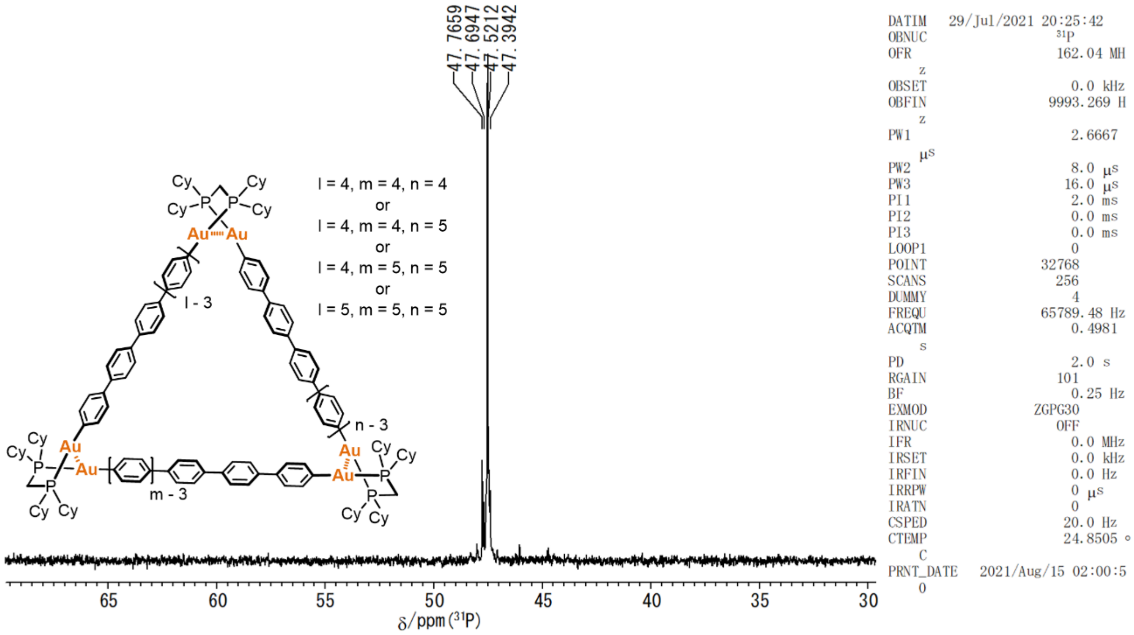

Figure S80. $^{31}P\{^{1}H\}$ NMR spectrum of mixture of Au complexes from **L4** and **L5** (162 MHz, $C_2D_2Cl_4$, r.t.).

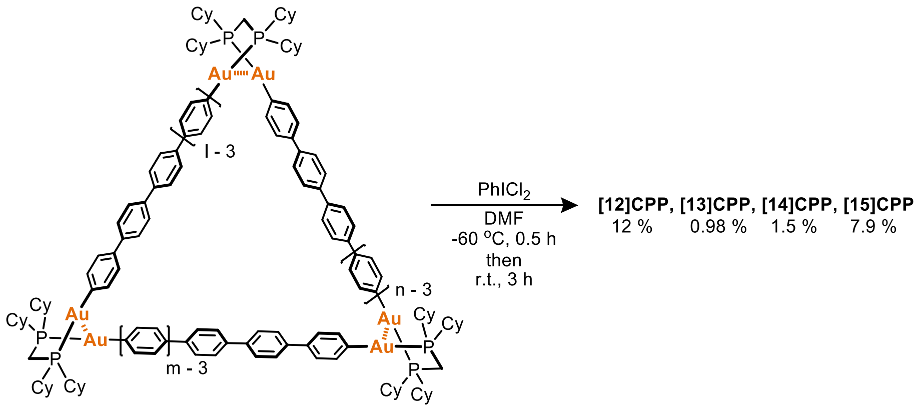

To a suspension of mixture of Au complexes (20.0 mg) in degassed DMF (5 mL) was added $PhICl_2$ (12 mmol/L in DMF, 4 mL, 0.048 mmol) dropwise with stirring at –60 ºC for 5 min. The reaction mixture was stirred at the same temperature for 30 min, then it was allowed to warm to 25 ºC and stirred for 3 h. Solvent and iodobenzene (by-product) were removed under vacuum. The crude product was purified by silica gel column chromatography (eluent; $CHCl_3$) to give a mixture of CPPs as a yellow solid (R_f = 0.74, 2.9 mg). ^{1}H NMR analysis indicated the formation of [12]-, [13]-, [14]-, and [15]CPPs in 12%, 0.98%, 1.5% and 7.9% yields over 2 steps, respectively.

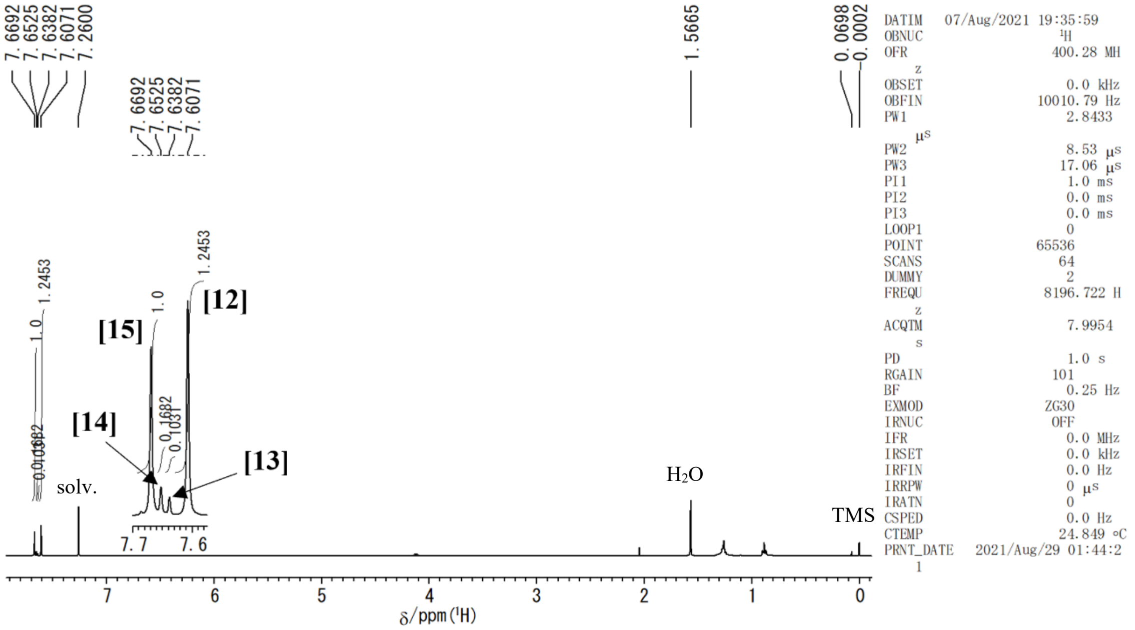

Figure S81. ^{1}H NMR spectrum of the CPP products after purification (400 MHz, $CDCl_3$, r.t.).

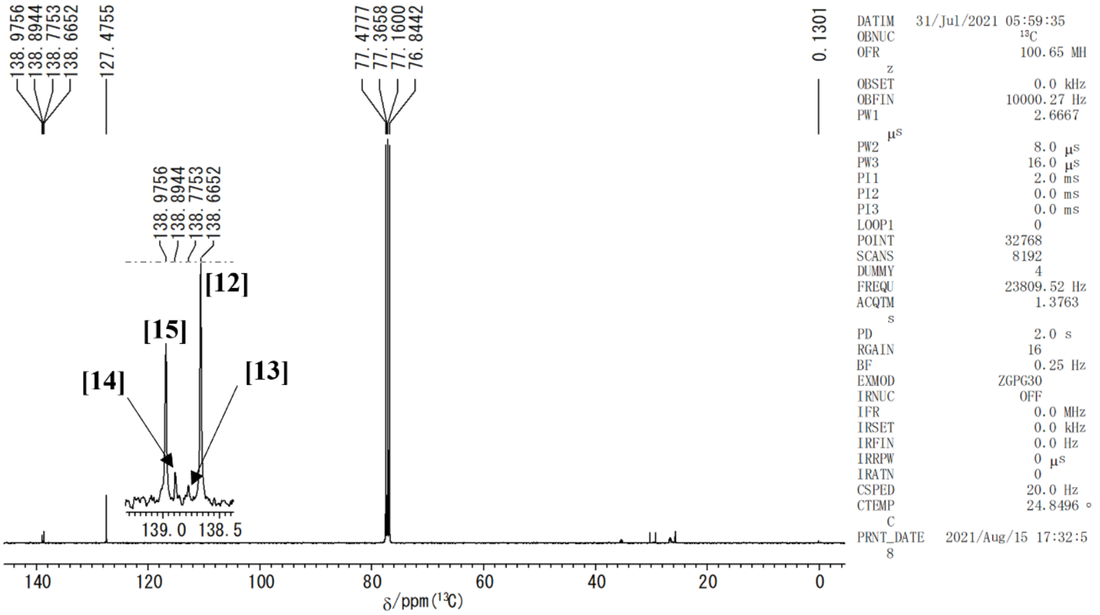

Figure S82. $^{13}C\{^1H\}$ NMR spectrum of the CPP products after purification (101 MHz, $CDCl_3$, r.t.).

Table S8. Calculation of NMR yields of CPPs (Method B) in **L4**/**L5** system

(**A**)	Total amounts of macrocyclic gold complex after transmetalation	173 mg
(**B**)	Amounts of the gold complexes used for the oxidative chlorination	20.0 mg
(**C**)	Total amounts of CPP mixtures after oxidative chlorination	2.9 mg
(**D**)	Amounts of **C** contained in the NMR tube	0.87 mg
(**E**)	Amounts of internal standard (TBB) contained in the NMR tube	1.20 mg (3.05 μmol)
(**F**)	NMR signal intensities<br>v.s. TBB (See Figure S81)	[12]CPP: 2.22<br>[13]CPP: 0.193<br>[14]CPP: 0.309<br>[15]CPP: 1.80
(**G**)	Ratio of number of protons in the structure v.s. TBB	[12]CPP: 24<br>[13]CPP: 26<br>[14]CPP: 28<br>[15]CPP: 30
(**H**)	Molar numbers of CPPs in **D**<br>(**E** × **F** ÷ **G**)	[12]CPP: 281 nmol<br>[13]CPP: 22.7 nmol<br>[14]CPP: 33.6 nmol<br>[15]CPP: 183 nmol
(**I**)	Molar numbers of CPPs in **C**<br>(**H** × **C** ÷ **D**)	[12]CPP: 0.938 μmol<br>[13]CPP: 0.0755 μmol<br>[14]CPP: 0.112 μmol<br>[15]CPP: 0.611 μmol
(**J**)	Molar numbers of CPPs when the entire amount of the complexes is used for the oxidative chlorination<br>(**I** × **A** ÷ **B**)	[12]CPP: 8.12 μmol<br>[13]CPP: 0.654 μmol<br>[14]CPP: 0.970 μmol<br>[15]CPP: 5.29 μmol
(**K**)	Theoretical yield relative to [Au_2Cl_2(dcpm)] (**1**)<br>(201 μmol ÷ 3)	66.7 μmol
(**L**)	NMR yield over 2 steps<br>(**J** ÷ **K** × 100)	**[12]CPP: 12%**<br>**[13]CPP: 0.98%**<br>**[14]CPP: 1.5%**<br>**[15]CPP: 7.9%**

Synthesis of $[Au_2(C_6H_4\text{-}C_{16}H_8\text{-}C_6H_4)(Cy_2PCH_2PCy_2)]_3$ (Au-pyr)

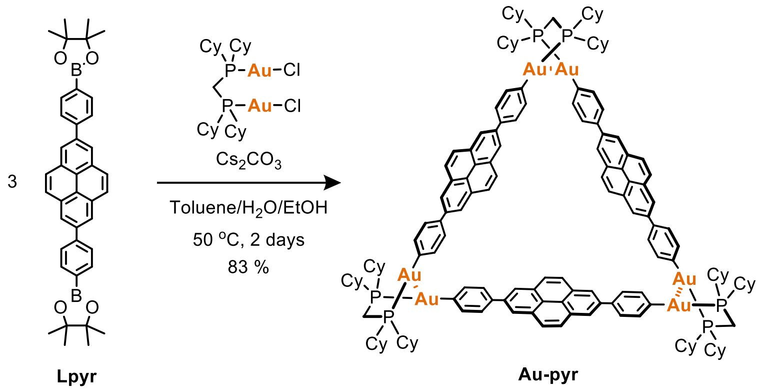

A mixture of pinB-C_6H_4-$C_{16}H_8$-C_6H_4-Bpin (**Lpyr**)[S20] (243 mg, 0.40 mmol), Cs_2CO_3 (792 mg, 2.4 mmol) and $[Au_2Cl_2(dcpm)]$ (349 mg, 0.40 mmol) in degassed toluene/H_2O/EtOH (16 mL/4 mL/4 mL) was stirred for 2 days at 50 °C under an argon atmosphere. After the reaction mixture allowed to cool to room temperature, the precipitates were collected by suction filtration and washed with toluene (30 mL), H_2O (20 mL), and EtOH (20 mL), then dried in *vacuo*. The Au complex, $[Au_2(C_6H_4\text{-}C_{16}H_8\text{-}C_6H_4)(Cy_2PCH_2PCy_2)]_3$ (**Au-pyr**), was obtained as off-white solid (383 mg, 0.11 mmol, 83%).

^{1}H NMR and ^{31}P NMR spectra suggest that the two isomers are exchanged slower than the NMR timescale (C_2 : D_3 = 1 : 1) (see Figure S18 for detail). Data for the dynamic mixture of C_2- and D_3-isomers of **Au-pyr**; ^{1}H NMR (400 MHz, $CDCl_3$, r.t.): δ 8.39 (s, 12H, $C_{16}H_8$ for C_2), 8.07 (s, 12H, $C_{16}H_8$ for C_2), 7.84 (s, 12 H, $C_{16}H_8$ for D_3), 7.76 (s, 24 H, C_6H_4 for C_2), 7.46 (br, 24 H, C_6H_4 for D_3), 7.10 (s, 12 H, $C_{16}H_8$ for D_3), 2.36-2.04 (br, 54H, C_6H_{11}, CH_2), 2.02-1.86 (br, 24H, C_6H_{11}), 1.84-1.48 (br, 12H, C_6H_{11}, overlapping with signal of H_2O), 1.45-1.23 (br, 48H, C_6H_{11}). ^{31}P{^{1}H} NMR (161 MHz, $CDCl_3$, r.t.): δ 48.1 (s, D_3), 47.9 (s, C_2). Anal. Calcd for $C_{159}H_{186}Au_6P_6$ $5H_2O$: C, 53.72; H, 5.56. Found: C, 53.52; H, 5.37.

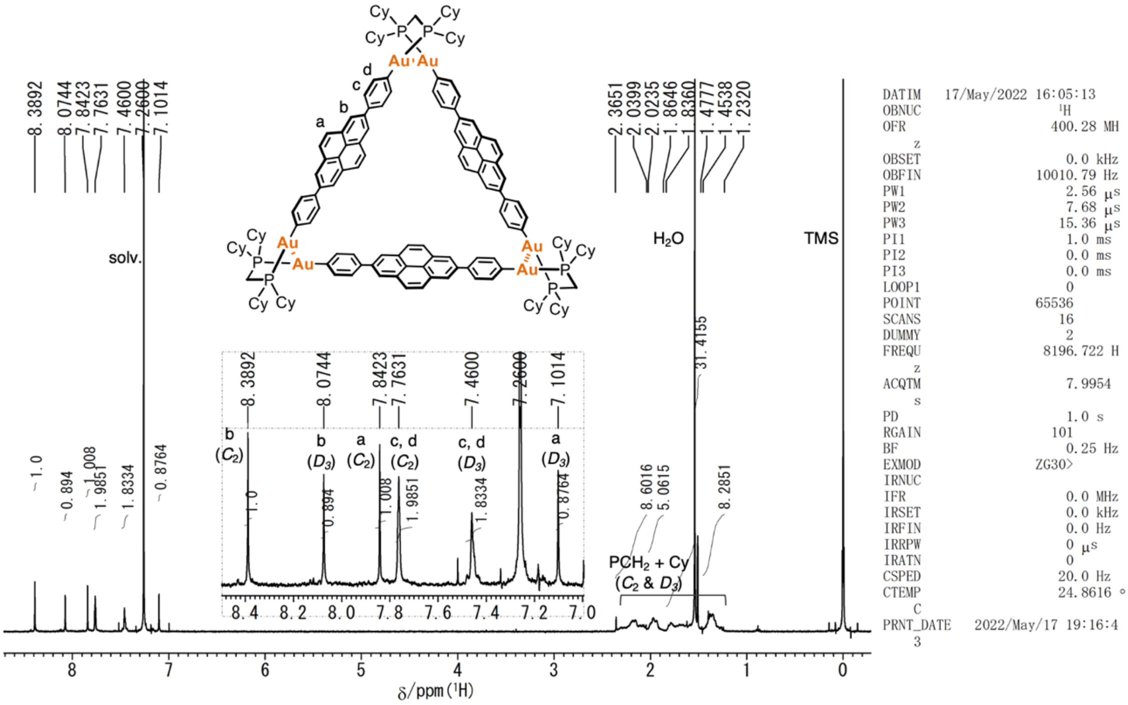

Figure S83. ^{1}H NMR spectrum of **Au-pyr** (dynamic mixture of C_2- and D_3-isomers) (400 MHz, $CDCl_3$, r.t.).

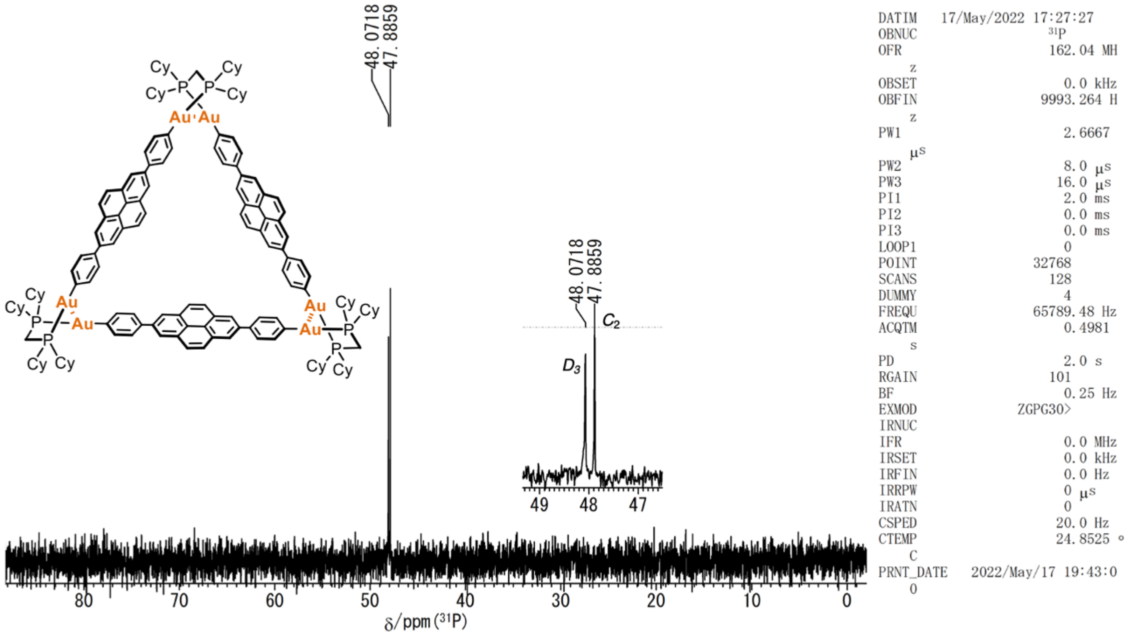

Figure S84. ^{31}P{^{1}H} NMR spectrum of **Au-pyr** (dynamic mixture of C_2- and D_3-isomers) (162 MHz, $CDCl_3$, r.t.).

Synthesis of Pyrene-Containing Nanohoop, $(C_6H_4–C_{16}H_8–C_6H_4)_3$ (Pyr-3)

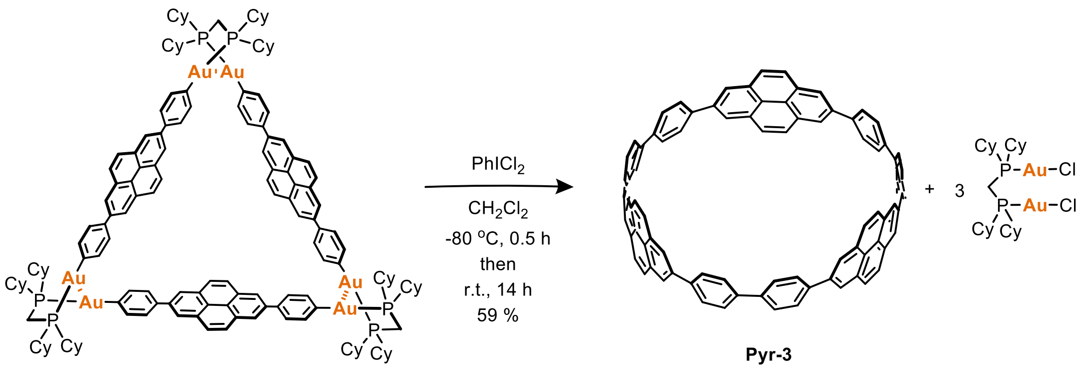

To a suspension of $[Au_2(C_6H_4\text{-}C_{16}H_8\text{-}C_6H_4)(Cy_2PCH_2PCy_2)]_3$ (**Au-pyr**) (350 mg, 0.10 mmol) in degassed CH_2Cl_2 (100 mL) was added $PhICl_2$ (10 mmol/L in CH_2Cl_2, 30 mL, 0.30 mmol) dropwise with stirring at –80 ºC for 20 min under an argon atmosphere. The reaction mixture was stirred at the same temperature for 30 min, then it was allowed to warm to 25 ºC and stirred for 14 h. After the solvent was removed under vacuum, the crude product was purified by silica gel column chromatography (eluent; $CHCl_3$) to give pyrene-containing nanohoop, $(C_6H_4–C_{16}H_8–C_6H_4)_3$ (**Pyr-3**) (R_f = 0.87, 62.1 mg, 59 μmol, 59%) as a pale-yellow solid, and $[Au_2Cl_2(dcpm)]$ (R_f = 0.15, 220 mg, 25 mmol, 84%) as a white solid. ^1H NMR (400 MHz, $CDCl_3$, r.t.): δ 8.26 (s, 12H, $C_{16}H_8$), 7.94 (s, 12H, $C_{16}H_8$), 7.78 (d, 12 H, J = 8.4 Hz, C_6H_4), 7.61 (d, 12 H, J = 8.4 Hz, C_6H_4). $^{13}C\{^1H\}$ NMR (101 MHz, $CDCl_3$, r.t.): δ 139.0 (s), 138.7 (s), 137.1 (s), 131.7 (s), 128.0 (s), 127.8 (s), 127.7 (s), 124.0 (s), 123.8 (s). HRMS (MALDI-TOF, DCTB): m/z: calcd for $C_{84}H_{48}$: 1056.3751 $[M]^+$; found: 1056.3760.

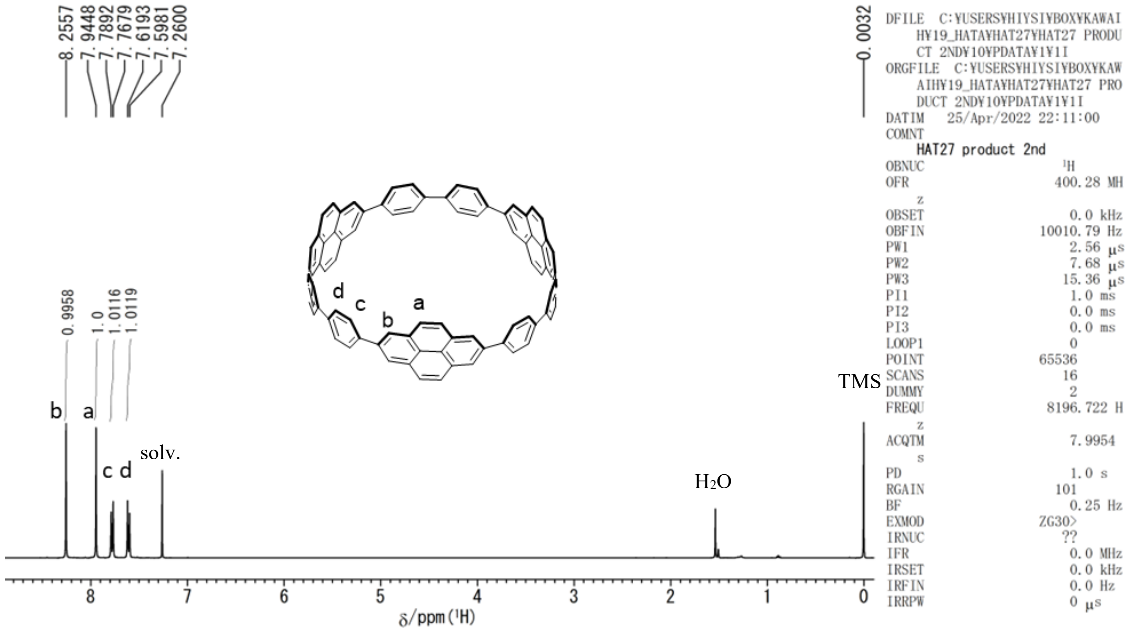

Figure S85. ^{1}H NMR spectrum of **Pyr-3** (400 MHz, $CDCl_3$, r.t.).

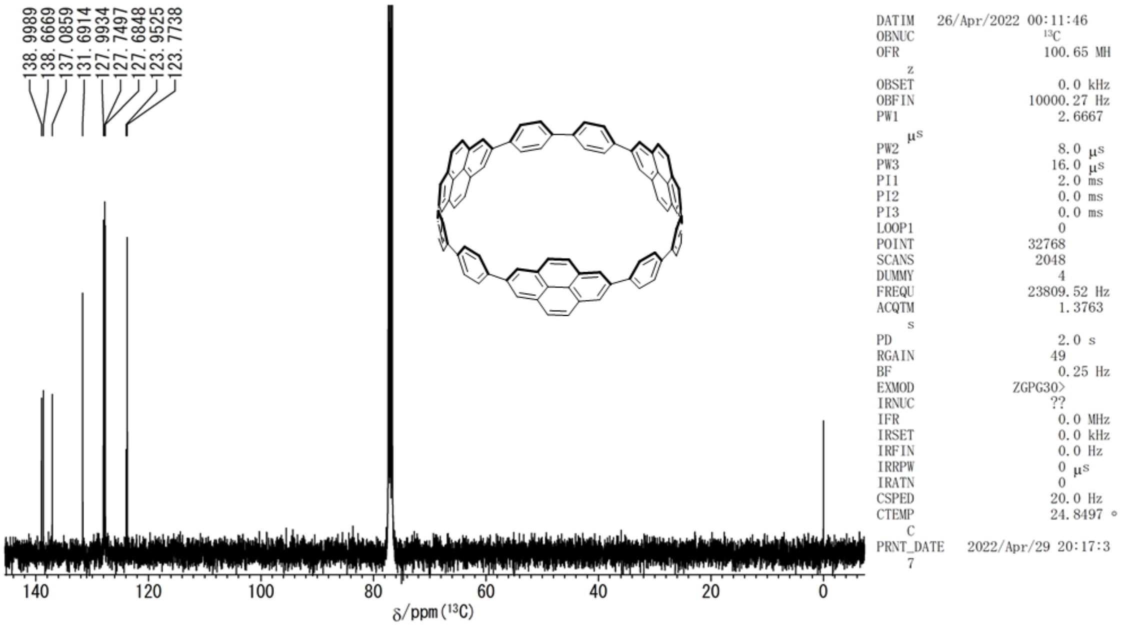

Figure S86. $^{13}C\{^{1}H\}$ NMR spectrum of **Pyr-3** (101 MHz, $CDCl_3$, r.t.).

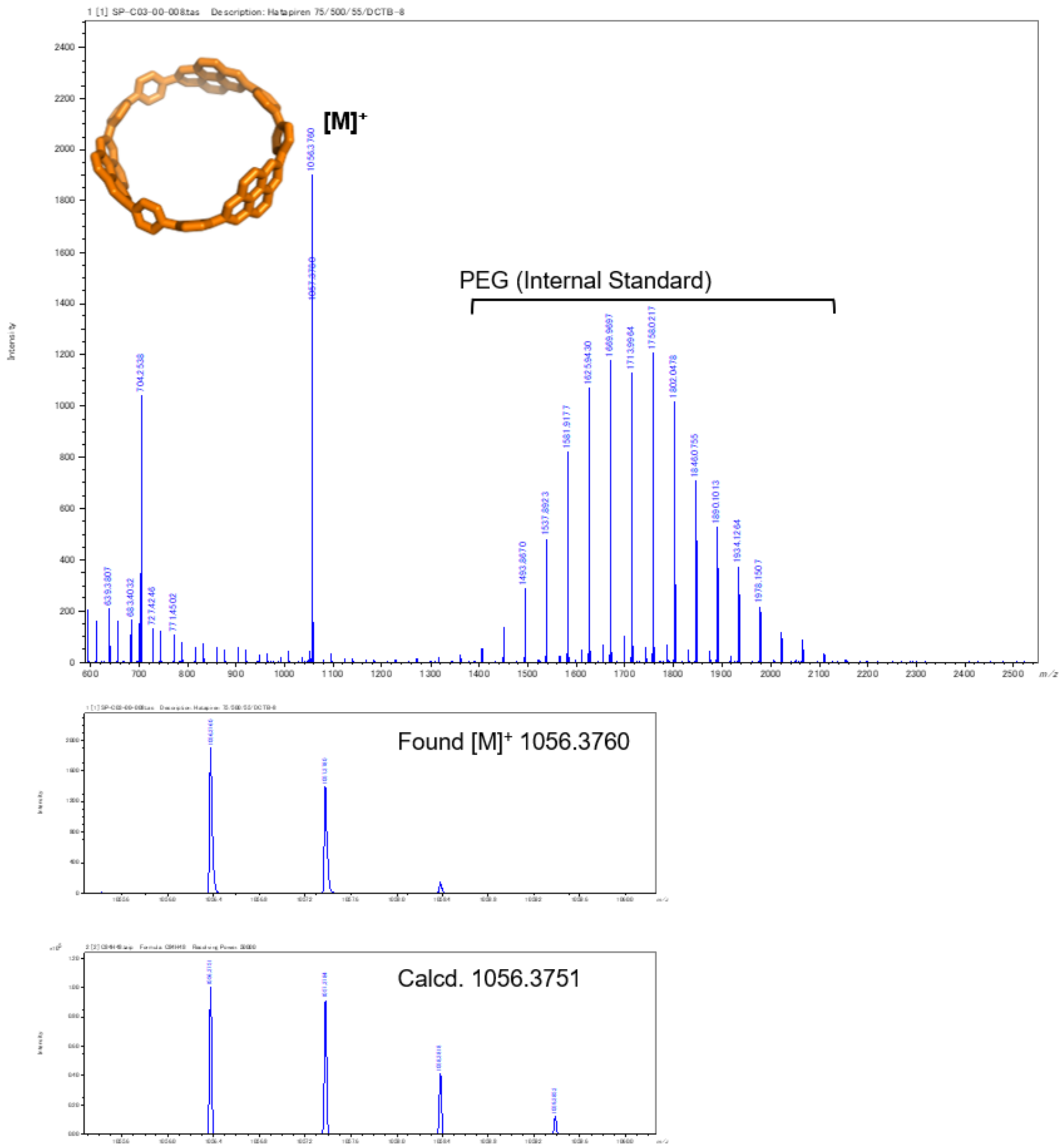

Figure S87. HRMS spectrum of **Pyr-3** (MALDI-TOF, DCTB).

Synthesis of pyrene-containing nanohoops (Pyr-1, Pyr-2, Pyr-3) by reorganization method

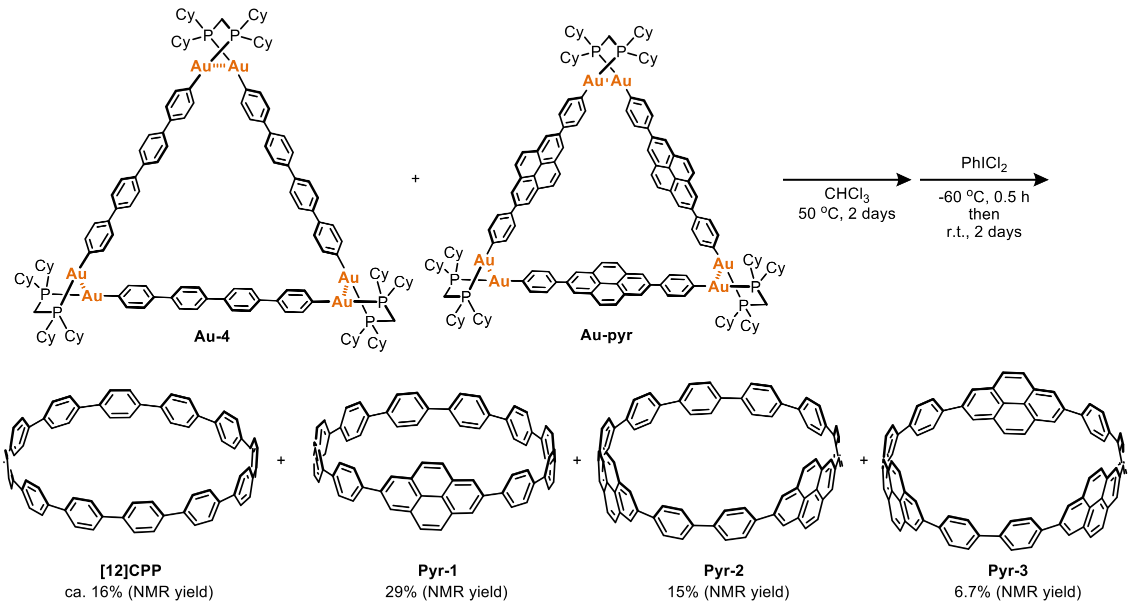

To a suspension of **Au-4** (133 mg, 40 μmol) and **Au-pyr** (139 mg, 40 μmol) in degassed $CHCl_3$ (80 mL) was stirred for 2 days at 50 °C under an argon atmosphere. After the reaction mixture allowed to cool to –60 °C, $PhICl_2$ (24 mmol/L in $CHCl_3$, 10 mL, 0.24 mmol) was added dropwise with stirring at the same temperature for 5 min. The reaction mixture was stirred at the same temperature for 30 min, then it was allowed to warm to 25 °C and stirred for 2 days. After the solvent was removed under vacuum, the crude product was purified by silica gel column chromatography (eluent; $CHCl_3$) to give a mixture of nanohoops as a yellow solid (52.8 mg) and [Au_2Cl_2(dcpm)] as a white solid (152 mg, 0.17 mmol, 73%). ^{1}H NMR analysis indicated the formation of [12]CPP, **Pyr-1**, **Pyr-2**, and **Pyr-3** in ca. 16%, 29%, 15% and 6.7% yields over 2 steps, respectively (The NMR yield of [12]CPP is not precise because the signal overlaps with other CPP derivatives.).

A portion of the mixture (19.4 mg) was purified by preparative gel permeation chromatography (eluent; $CHCl_3$) to give **Pyr-1** (2.69 mg, 2.8 μmol), **Pyr-2** (2.51 mg, 2.5 μmol) in a pure form. Data for **Pyr-1**: ^{1}H NMR (400 MHz, $CDCl_3$, r.t.): δ 8.28 (s, 4H, $C_{16}H_8$), 7.99 (s, 4H, $C_{16}H_8$) 7.79 (d, 4H, J = 8.6 Hz, C_6H_4), 7.64-7.59 (m, 36H, C_6H_4). $^{13}C\{^{1}H\}$ NMR (101 MHz, $CDCl_3$, r.t.): δ 139.3 (s), 138.7 (m), 137.5 (s), 131.9 (s), 129.2 (s), 128.3 (s), 127.9 (s), 127.8 (s), 127.7 (s), 127.5 (m), 124.1 (s). HRMS (MALDI-TOF, Dithranol): m/z: calcd for $C_{76}H_{48}$: 960.3775 [M^+]; found: 960.3751. Data for **Pyr-2**: ^{1}H NMR (400 MHz, $CDCl_3$, r.t.): δ 8.28 (s, 4H, $C_{16}H_8$), 8.25 (s, 4H, $C_{16}H_8$) 7.97 (s, 8H, $C_{16}H_8$), 7.80 (d, 4H, J = 8.7 Hz, C_6H_4), 7.76 (d, 4H, J = 8.6 Hz, C_6H_4), 7.63-7.54 (m, 24H, C_6H_4). $^{13}C\{^{1}H\}$ NMR (101 MHz, $CDCl_3$, r.t.): δ 139.4 (s), 139.1 (s), 138.9 (s), 138.8 (s), 138.8 (s), 138.5 (s), 138.5 (s), 137.5 (s), 137.2 (s), 131.9 (s), 131.8 (s), 129.2 (s), 128.4 (s), 128.3 (s), 128.2 (s), 128.0 (s), 127.8 (s), 127.8 (s), 127.7 (s), 127.6 (s), 127.4 (s), 125.4 (s), 124.1 (s), 124.1 (s), 124.1 (s), 123.9 (s). HRMS (MALDI-TOF, Dithranol): m/z: calcd for $C_{80}H_{48}$: 1008.3751 [M^+]; found: 1008.3748.

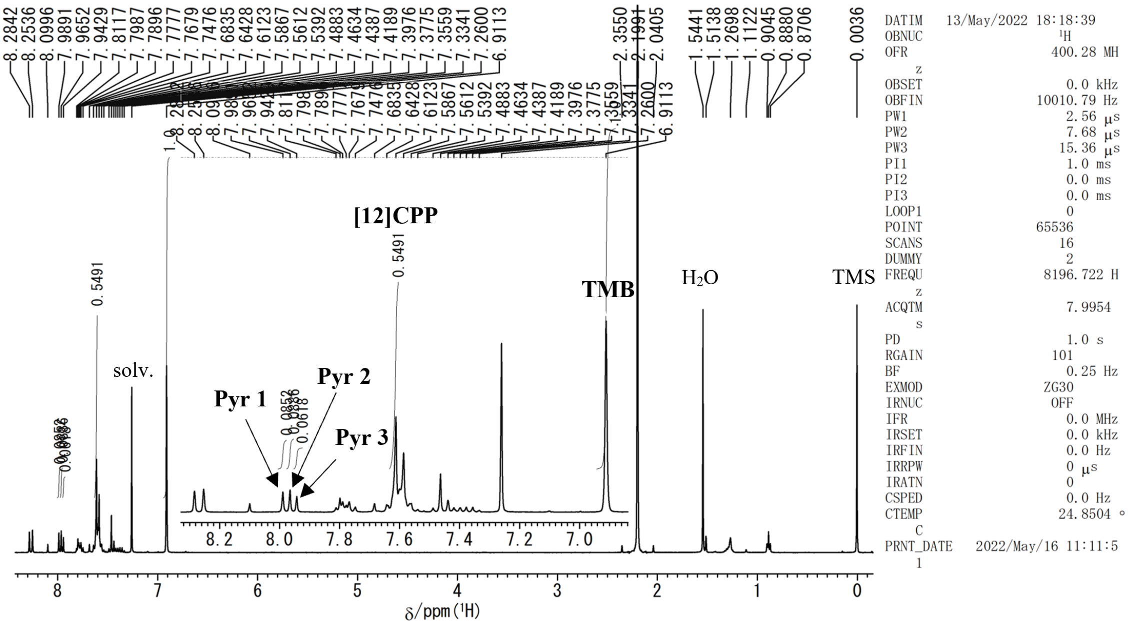

Figure S88. ^{1}H NMR spectrum of the mixture of nanohoops after purified by silica gel column chromatography with internal standard, 1,2,4,5-tetramethylbenzene (TMB) (400 MHz, $CDCl_3$, r.t.).

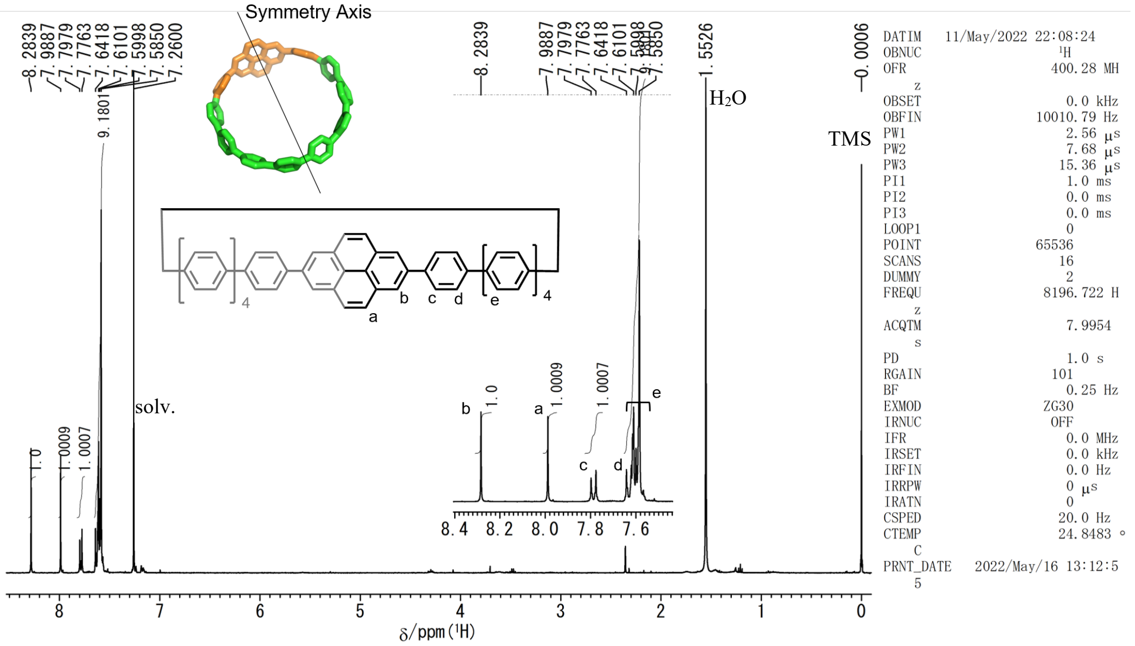

Figure S89. ^{1}H NMR spectrum of **Pyr-1** (400 MHz, $CDCl_3$, r.t.).

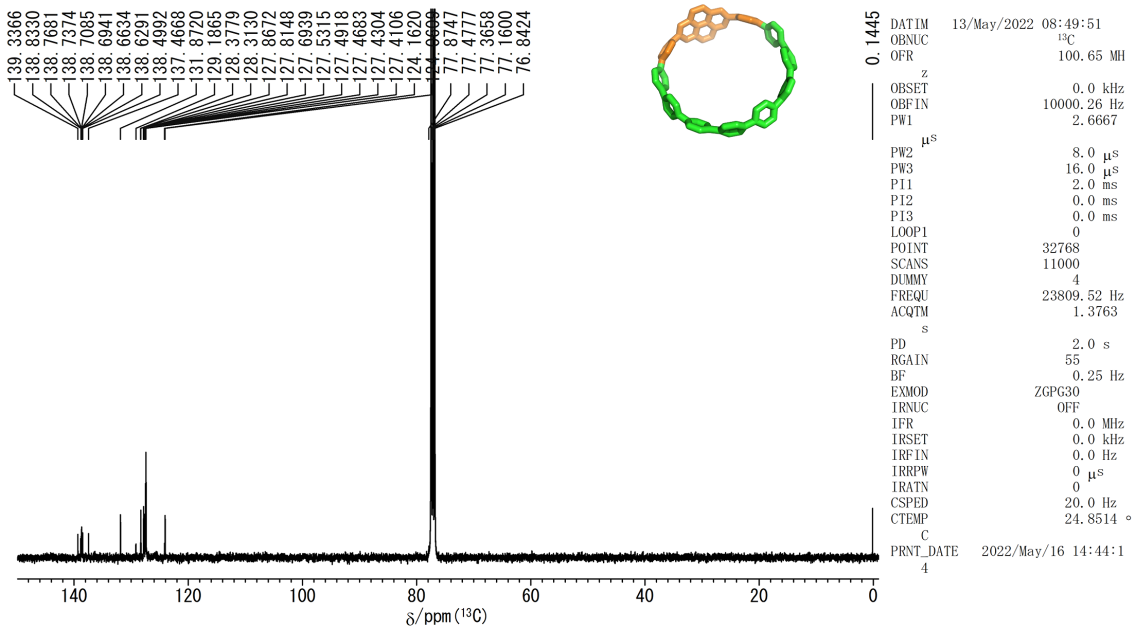

Figure S90. $^{13}C\{^{1}H\}$ NMR spectrum of **Pyr-1** (101 MHz, $CDCl_3$, r.t.).

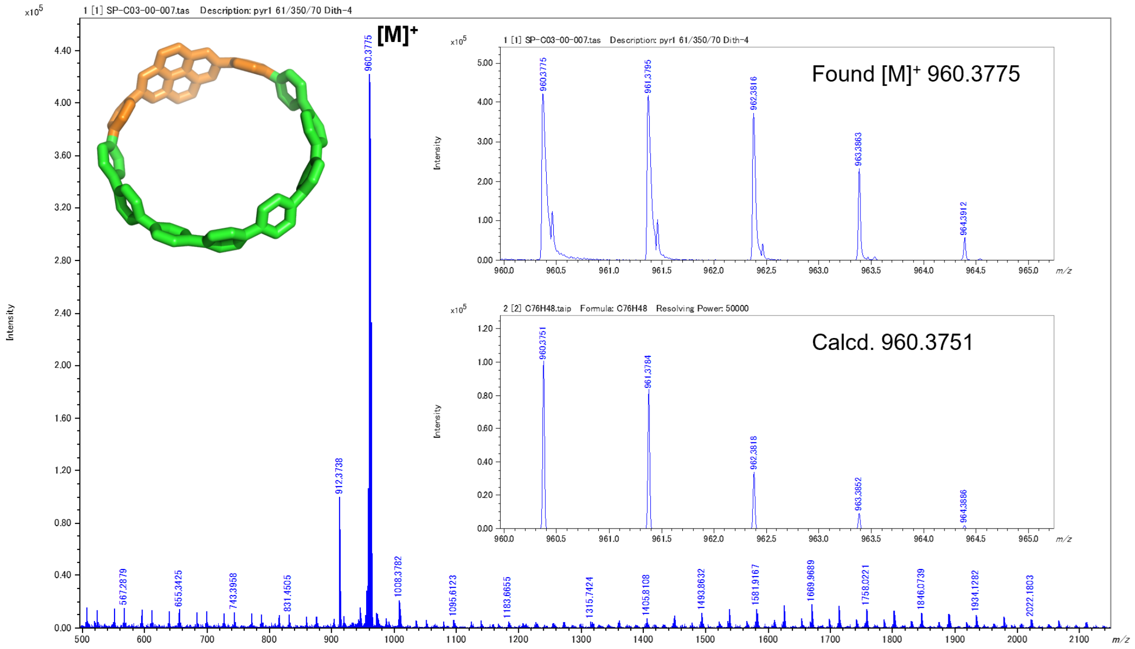

Figure S91. HRMS spectrum of **Pyr-2** (MALDI-TOF, Dithranol).

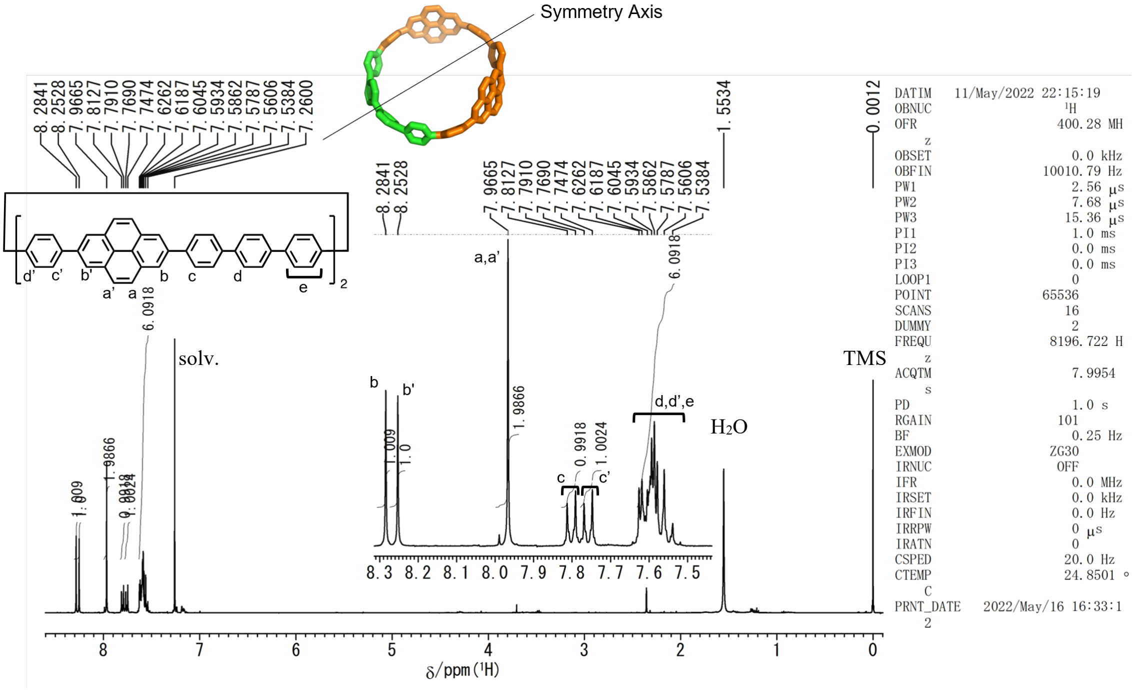

Figure S92. ^{1}H NMR spectrum of **Pyr-2** (400 MHz, $CDCl_3$, r.t.).

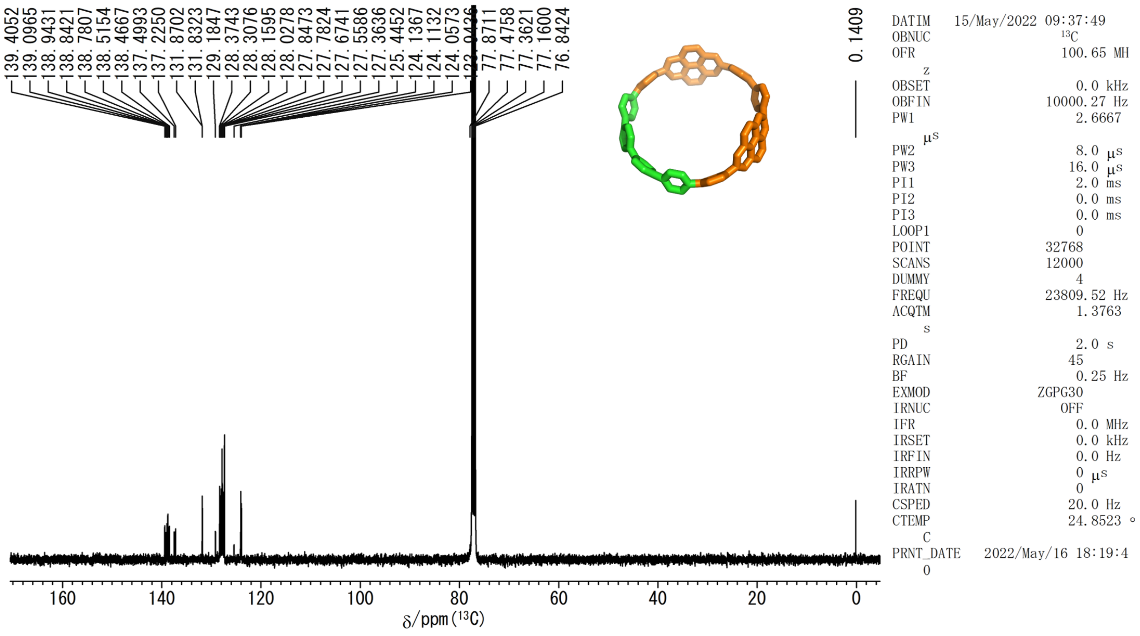

Figure S93. $^{13}C\{^{1}H\}$ NMR spectrum of **Pyr-2** (101 MHz, $CDCl_3$, r.t.).

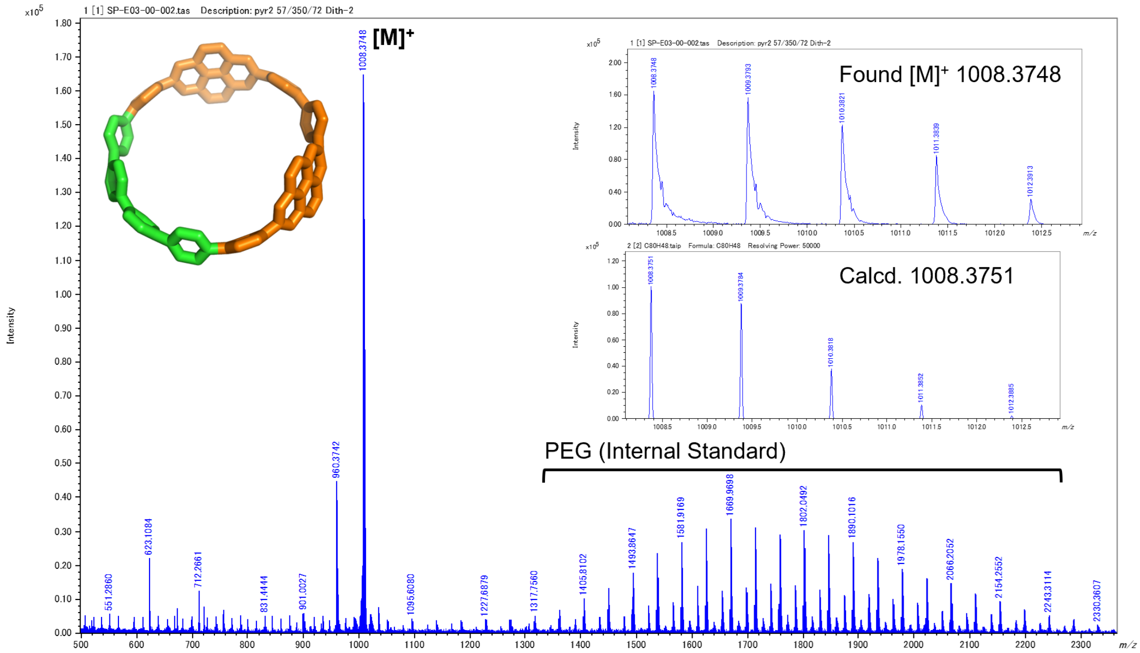

Figure S94. HRMS spectrum of **Pyr-2** (MALDI-TOF, Dithranol).

Table S9. Calculation of NMR yields of pyrene-containing CPP derivatives

(**A**)	Total amounts of CPP mixture after oxidative chlorination	52.8 mg
(**B**)	Amounts of **A** contained in the NMR tube	0.97 mg
(**C**)	Amounts of internal standard (TMB) contained in the NMR tube	1.35 mg (10.1 μmol)
(**D**)	NMR signal intensities<br>v.s. TMB (See Figure S88)	[12]CPP: 0.549[a]<br>**Pyr-1**: 0.0852<br>**Pyr-2**: 0.0886<br>**Pyr-3**: 0.0618
(**E**)	Ratio of number of protons in the structure v.s. TBB	[12]CPP: 24<br>**Pyr-1**: 2<br>**Pyr-2**: 4<br>**Pyr-3**: 6
(**F**)	Molar numbers of CPPs in **B**<br>(**C** × **D** ÷ **E**)	[12]CPP: ca. 230 nmol[a]<br>**Pyr-1**: 428 nmol<br>**Pyr-2**: 223 nmol<br>**Pyr-3**: 104 nmol
(**G**)	Molar numbers of CPPs in **A**<br>(**F** × **A** ÷ **B**)	[12]CPP: ca. 12.5 μmol[a]<br>**Pyr-1**: 23.3 μmol<br>**Pyr-2**: 12.1 μmol<br>**Pyr-3**: 5.64 μmol
(**H**)	Theoretical yield	80 μmol
(**I**)	NMR yield<br>(**G** ÷ **H** × 100)	**[12]CPP: ca. 16%**[a]<br>**Pyr-1: 29%**<br>**Pyr-2: 15%**<br>**Pyr-3: 6.7%**

[a] The signal intensity of [12]CPP is not precise because the signal overlaps with other CPP derivatives.

S5. Crystallographic study

Single crystals of Au complexes suitable for X-ray crystallography were obtained by vapor diffusion of CH_3CN into $C_2H_2Cl_4$ solutions of Au complexes (**Au-3**, **Au-4**), or by liquid/liquid diffusion of hexane and CH_2Cl_2 solution of Au complexes (**Au$_C$-FF**, **Au$_P$-FF** and **Au$_C$-HH**). The single X-ray structure determination was performed on a Rigaku XtaLAB Synergy-DW diffractometer or a Bruker D8 QUEST diffractometer. Due to very small size of the crystals, the X-ray structure of polymorphic **Au-4** with C_2-symmetry was solved using the structure analysis tool "What Is This".[S21] A numerical absorption correction (μ) was applied. The structure was solved by direct methods and refined by the full-matrix least-squares method on F^2 with anisotropic temperature factors for non-hydrogen atoms.[S22,S23] All the hydrogen atoms were located at the calculated positions and refined with riding. The disordered cyclohexyl and phenylene groups were restricted by DFIX and SIMU for **Au-3** and **Au-4**, or by ISOR and RIGU for **Au$_C$-FF**. The disordered solvent molecules were removed by SQUEEZE program for **Au-3** and **Au-4**. ORTEP drawings of Au complexes are shown in Figures S95 and S96, and crystallographic data collection and refinement information are listed in Tables S10 and S11.

Single-crystal X-ray structures that contain the supplementary crystallographic data for this paper can be obtained free of charge from the Cambridge Crystallographic Data Centre at www.ccdc.cam.ac.uk/data_request/cif.

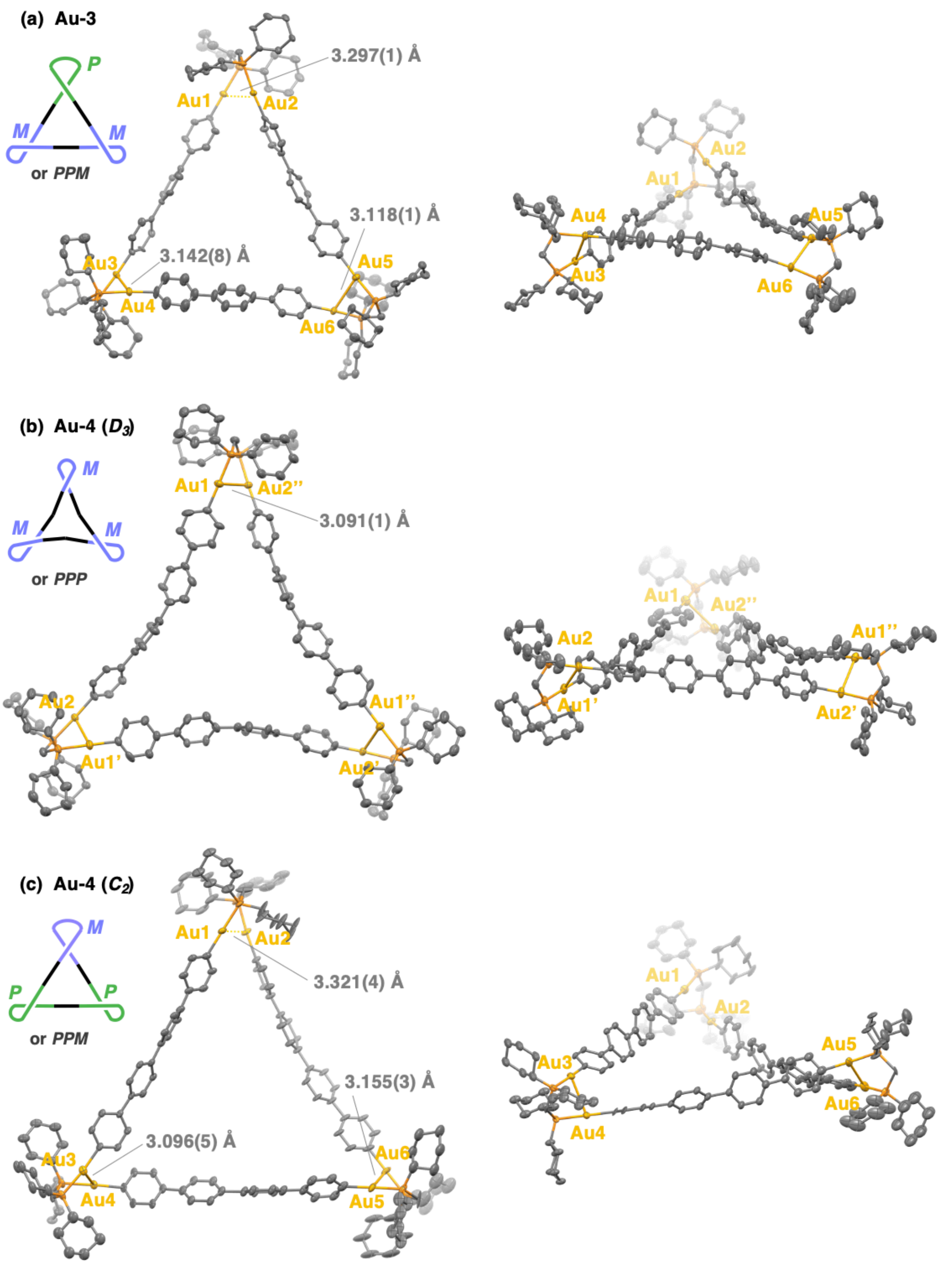

Figure S95. ORTEP drawing of (a) **Au-3** with C_2 symmetry, (b) **Au-4** with D_3 symmetry, and (c) **Au-4** with C_2 symmetry (30 % level of probability). Left: top view, Right: side view. Hydrogen atoms are omitted for clarity.

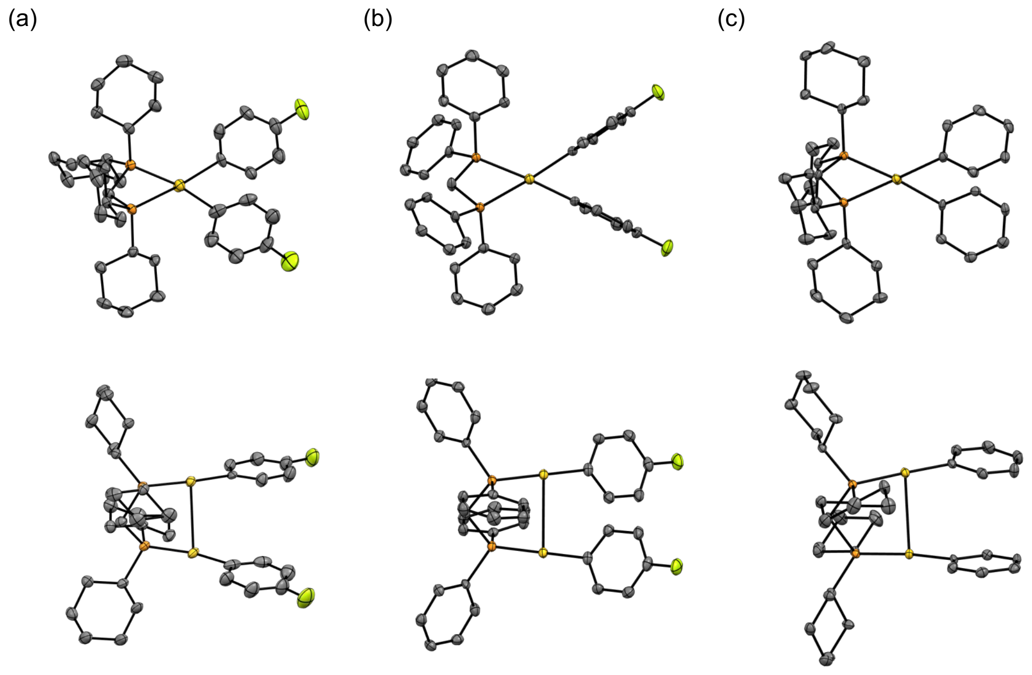

Figure S96. ORTEP drawing of (a) **Au$_C$-FF**, (b) **Au$_P$-FF**, and (c) **Au$_C$-HH** (30 % level of probability). Hydrogen atoms and solvent molecules are omitted for clarity.

Table S10. Crystal data and structure refinement for macrocyclic Au complexes, **Au-3** and polymorphs of **Au-4**.

Compound	**Au-3**	**Au-4** ***D*₃**	**Au-4** ***C*₂**
CCDC No.	2108728	2108731	2108730
Solvent system	$C_2H_2Cl_4$/MeCN	$C_2H_2Cl_4$/MeCN	$C_2H_2Cl_4$/MeCN
Color	Colorless	Colorless	Colorless
Formula	$C_{129}H_{174}Au_6P_6$	$C_{49}H_{62}Au_2P_2$	$C_{147}H_{185}Au_6P_6$
F_w	3092.29	1106.86	3319.56
Crystal size /mm	0.02 x 0.02 x 0.01	0.02 x 0.01 x 0.01	0.01 x 0.01 x 0.01
Crystal system	triclinic	trigonal	triclinic
Space group	*P*-1 (No. 2)	*R*-3 (No. 148)	*P*-1 (No. 2)
a /Å	17.3161(5)	24.9449(10)	16.7415(10)
b /Å	21.8509(6)	24.9449(10)	24.4204(15)
c /Å	22.0959(6)	41.743(5)	25.1552(13)
α /°	116.891(3)	90	116.612(5)
β /°	95.435(2)	90	92.217(5)
γ /°	100.908(3)	120	90.424(5)
V /Å^3	7164.8(4)	22494(3)	9184.4(10)
Z	2	18	2
D_c /g cm^{-3}	1.433	1.471	1.200
No. of reflections measured	98287	26536	19012
No. of unique reflections	29007	9794	16110
No. observations ($I > 2\sigma(I)$)	17383	5409	8661
R	0.0909	0.0960	0.1937
R_w	0.2329	0.2640	0.4366
GOF on F^2	1.019	1.076	1.453

Table S11. Crystal data and structure refinement for acyclic Au complexes, **Au$_C$-FF**, **Au$_P$-FF** and **Au$_C$-HH**.

Compound	**Au$_C$-FF**	**Au$_P$-FF**	**Au$_C$-HH**
CCDC No.	2108732	2108733	2108729
Solvent system	CH_2Cl_2/*n*-hexane	CH_2Cl_2/*n*-hexane	CH_2Cl_2/*n*-hexane
Color	Colorless	Colorless	Colorless
Formula	$C_{37}H_{54}Au_2F_2P_2$	$C_{37}H_{30}Au_2F_2P_2$	$C_{37}H_{56}Au_2P_2$
F_w	992.67	968.48	956.69
Crystal size /mm	0.05 x 0.01 x 0.01	0.01 x 0.01 x 0.01	0.02 x 0.02 x 0.0.2
Crystal system	triclinic	monoclinic	triclinic
Space group	*P*-1 (No. 2)	*C2/c* (No.15)	*P*-1 (No.2)
a /Å	10.7380(6)	16.1863(15)	10.8481(5)
b /Å	12.4998(7)	10.7246(15)	10.8810(5)
c /Å	14.9946(2)	18.106(2)	15.6144(7)
α /^{o}	99.814(2)	90	80.817(1)
β /^{o}	96.734(2)	98.432(6)	87.266(2)
γ /^{o}	114.407(2)	90	80.851(1)
V /Å^3	1765.99(17)	3109.1(7)	1795.86(14)
Z	2	4	2
D_c /g cm^{-3}	1.867	2.069	1.769
No. of reflections measured	21552	5076	11819
No. of unique reflections	8778	4025	8993
No. observations ($I > 2\sigma(I)$)	7574	3734	8511
R	0.0448	0.0463	0.0469
R_w	0.1328	0.1857	0.1341
GOF on F^2	1.065	1.162	1.056

S6. Computational Details

All calculations were carried out with Gaussian 16 Rev. B. Program package[S24]. The geometries of Au complexes, **Au$_C$-HH** and **Au$_P$-HH**, were optimized by M06[S25] functional in conjunction with 6-31g[S26] basis set for C, H, P, and LANL2TZ(f)[S27] basis set for Au. Frontier orbitals of Au complexes were computed by the same level of theory.

Optimized Geometries of Au complexes

Au$_C$-HH

Au	-1.32154	1.03818	0.59564
P	-1.48926	0.01003	-1.55236
C	-0.76035	-1.70234	-1.56568
H	-0.28164	-1.89893	-2.53076
C	-0.69633	0.95136	-2.96054
C	-3.22775	-0.30561	-2.13013
P	0.47158	-1.93062	-0.19323
Au	1.81596	0.01074	0.12668
C	-0.64084	-2.37075	1.22703
C	1.40216	-3.45654	-0.68990
H	-1.57160	-2.43341	-1.45105
C	-1.13223	1.82095	2.48301
C	-0.05418	2.66755	2.78787
C	0.15934	3.14126	4.08013
C	-0.70710	2.78235	5.10874
C	-1.78822	1.95084	4.83215
C	-1.99523	1.47979	3.53697
H	0.64555	2.95348	2.00172
H	1.01107	3.79106	4.28117
H	-0.54170	3.14893	6.12024
H	-2.47484	1.66755	5.62902
H	-2.85108	0.82989	3.34553
C	2.98230	1.69645	0.27227
C	3.22694	2.33481	1.49923
C	3.97260	3.50912	1.57645
C	4.50220	4.08078	0.42321

C	4.28587	3.46376	-0.80493
C	3.53981	2.28892	-0.87310
H	2.81324	1.91456	2.41671
H	4.13797	3.98157	2.54399
H	5.08118	5.00049	0.48174
H	4.69958	3.89727	-1.71493
H	3.39373	1.82094	-1.84970
C	-4.01542	-1.07606	-1.06530
C	-3.94627	1.00347	-2.46904
C	-6.16001	-0.02266	-1.84742
C	-5.38545	0.74920	-2.90761
C	-5.45303	-1.32873	-1.50885
H	-3.52724	-2.03169	-0.82289
H	-5.44911	-1.98139	-2.39699
H	-5.99460	-1.87223	-0.72446
H	-6.23818	0.59239	-0.93693
H	-4.01427	-0.48355	-0.13414
H	-7.18620	-0.21603	-2.18405
H	-3.15731	-0.92147	-3.04630
H	-5.37989	0.17148	-3.84620
H	-5.87902	1.70300	-3.13197
H	-3.94283	1.64929	-1.57428
H	-3.40820	1.55105	-3.25501
C	-0.70091	2.44637	-2.60914
C	0.72170	0.50927	-3.33113
C	1.25600	2.85103	-4.12028
C	1.28791	1.36643	-4.46131
C	-0.15670	3.28749	-3.75703
H	-1.70868	2.78020	-2.32682
H	-0.81511	3.17773	-4.63530
H	-0.17654	4.34892	-3.48113
H	1.92095	3.04344	-3.26271
H	-0.07170	2.59174	-1.71462
H	1.64262	3.44298	-4.95949
H	-1.34593	0.78644	-3.84039
H	0.69679	1.19215	-5.37582

H	2.31267	1.04231	-4.68573
H	1.37213	0.59441	-2.44325
H	0.74194	-0.54617	-3.63642
C	2.20959	-3.19434	-1.96634
C	2.33410	-3.89768	0.44414
C	3.96671	-4.83493	-1.22608
C	3.16932	-5.10913	0.04201
C	3.04214	-4.41070	-2.35968
H	1.54797	-2.91421	-2.79803
H	2.36765	-5.24571	-2.61019
H	3.61633	-4.18986	-3.26825
H	4.69204	-4.02936	-1.03040
H	2.87543	-2.33181	-1.79176
H	4.54933	-5.71883	-1.51444
H	0.66880	-4.25828	-0.88945
H	2.49979	-5.96815	-0.12835
H	3.83512	-5.39155	0.86700
H	3.00053	-3.05596	0.69953
H	1.75909	-4.12461	1.35184
C	-1.24450	-3.77384	1.17697
C	-0.00844	-2.06935	2.58854
C	-1.64804	-3.65877	3.65695
C	-1.03441	-2.26407	3.69960
C	-2.27037	-3.94591	2.29481
H	-1.71171	-3.97094	0.19939
H	-3.10620	-3.24788	2.12152
H	-2.69449	-4.95796	2.26526
H	-0.86325	-4.40732	3.85589
H	-0.44981	-4.52793	1.29967
H	-2.39711	-3.77546	4.45029
H	-1.46561	-1.64227	1.10794
H	-1.82571	-1.50590	3.57994
H	-0.57154	-2.07054	4.67505
H	0.85642	-2.73098	2.75972
H	0.37179	-1.03750	2.59972

Au$_P$-HH

Au	0.16792	-1.70783	0.71769
P	-1.61228	-0.24186	1.29276
C	-2.31647	0.56575	-0.22480
H	-2.76381	1.53421	0.02796
C	-1.11799	1.17357	2.32840
C	0.15403	1.16505	2.90682
H	0.81790	0.31663	2.73910
C	0.58633	2.24967	3.66475
H	1.58623	2.24012	4.09164
C	-0.24632	3.34803	3.84488
H	0.09516	4.19875	4.42999
C	-1.51704	3.36482	3.27273
H	-2.16911	4.22342	3.41497
C	-1.95100	2.28353	2.51649
H	-2.95013	2.30242	2.08031
C	-3.09939	-0.98470	2.05184
C	-3.67357	-0.53902	3.24240
H	-3.24471	0.31059	3.77029
C	-4.79273	-1.18611	3.76008
H	-5.23423	-0.83560	4.69003
C	-5.34240	-2.27557	3.09447
H	-6.21582	-2.77848	3.50267
C	-4.76710	-2.73054	1.90995
H	-5.18598	-3.59104	1.39386
C	-3.64571	-2.09447	1.39553
H	-3.17835	-2.46692	0.48147
P	-0.99668	0.83264	-1.50565
Au	1.12069	1.33302	-0.53948
C	-1.04401	-0.70541	-2.48177
C	0.16055	-1.36196	-2.74696
H	1.09905	-0.93485	-2.39321
C	0.16217	-2.58075	-3.41888
H	1.10394	-3.09638	-3.58900
C	-1.03766	-3.14666	-3.83275
H	-1.03734	-4.10339	-4.34985

C	-2.24389	-2.49448	-3.58205
H	-3.18235	-2.93575	-3.90960
C	-2.24823	-1.28088	-2.90632
H	-3.19646	-0.77679	-2.71712
C	-1.73251	2.15523	-2.53124
C	-2.16516	1.97043	-3.84439
H	-2.08935	0.99007	-4.31077
C	-2.68813	3.04288	-4.56247
H	-3.02103	2.89281	-5.58680
C	-2.78276	4.29933	-3.97556
H	-3.19117	5.13431	-4.53987
C	-2.34294	4.49168	-2.66774
H	-2.40168	5.47639	-2.21032
C	-1.81173	3.42768	-1.95192
H	-1.44062	3.58462	-0.93694
H	-3.11116	-0.07487	-0.62499
C	1.76150	-2.85749	0.12543
C	3.06700	-2.36588	0.28402
C	4.17286	-3.06113	-0.20068
C	4.00142	-4.27719	-0.85501
C	2.71721	-4.79030	-1.01746
C	1.61513	-4.08741	-0.53447
H	3.22756	-1.40940	0.78376
H	5.17139	-2.64518	-0.06710
H	4.86274	-4.82345	-1.23526
H	2.57303	-5.74462	-1.52305
H	0.61787	-4.50331	-0.68513
C	2.94727	1.76457	0.29568
C	4.09998	1.04321	-0.05564
C	5.33354	1.30238	0.53814
C	5.45067	2.29995	1.50103
C	4.32582	3.03651	1.86042
C	3.09536	2.77041	1.26370
H	4.03430	0.24924	-0.80102
H	6.20684	0.72007	0.24689
H	6.41285	2.50337	1.96738

H	4.40750	3.82486	2.60821
H	2.22662	3.35771	1.56574

S7. References

[S1] M. S. Carle, G. K. Shimokura, G. K. Murphy, *Eur. J. Org. Chem.* **2016**, 3930–3933.

[S2] M. R. Talipov, A. Boddeda, Q. K. Timerghazin, R. Rathore, *J. Phys. Chem.* **2014**, *118,* 21400–21408.

[S3] H. Noguchi, K. Hojo, M. Suginome, *J. Am. Chem. Soc.* **2007**, *129,* 758–759.

[S4] T. Tani, Y. Sawatsugawa, Y. Sano, Y. Hirataka, N. Takahashi, S. Hashimoto, T. Sugiura, T. Tsuchimoto, *Adv. Synth. Catal.* **2019**, *361*, 1815–1834.

[S5] B. Kemper, Y. R. Hristova, S. Tacke, L. Stegemann, L. S. Bezouwen, M. C. A. Stuart, J. Klingauf, C. A. Strassertbd, P. Besenius, *Chem. Commun.* **2015**, *51*, 5253–5256.

[S6] M. J. Irwin, L. M. Rendina, J. J. Vittal, R. J. Puddephatt, *Chem. Commun.* **1996**, 1281–1282.

[S7] (a) H. Schmidbaur, A. Wohlleben, F. Wagner, O. Orama, G. Huttner, *Chem. Ber.* **1977**, *110*, 1748–1754; (b) M.-C. Brandys, M.-C. Jennings, R. J. Puddephatt, *J. Chem. Soc.*, *Dalton Trans*. **2000**, 4601–4606.

[S8] R. Jasti, J. Bhattacharjee, J. B. Neaton, C. R. Bertozzi, *J. Am. Chem. Soc.* **2008**, *130*, 17646–17647.

[S9] E. R. Darzi, T. J. Sisto, R. Jasti, *J. Org. Chem*. **2012**, *77*, 6624–6628.

[S10] Y. Ishii, S. Matsuura, Y. Segawa, K. Itami, *Org. Lett.* **2014**, *16*, 2174–2176.

[S11] Y. Ishii, Y. Nakanishi, H. Omachi, S. Matsuura, K. Matsui, H. Shinohara, Y. Segawa, K. Itami, *Chem. Sci*. **2012**, *3*, 2340–2345.

[S12] T. Iwamoto, Y. Watanabe, Y. Sakamoto, T. Suzuki, S. Yamago, *J. Am. Chem. Soc.* **2011**, *133*, 8354–8361.

[S13] H. Takaba, H. Omachi, Y. Yamamoto, J. Bouffard, K. Itami, *Angew. Chem. Int. Ed.* **2009**, *48*, 6112–6116.

[S14] H. Omachi, S. Matsuura, Y. Segawa, K. Itami, *Angew. Chem. Int. Ed.* **2010**, *49*, 10202–10205.

[S15] Y. Tsuchido, R. Abe, T. Ide, K. Osakada, *Angew. Chem. Int. Ed.* **2020**, *59*, 22928 –22932.

[S16] W. J. Wolf, M. S. Winston, F. D. Toste, *Nat. Chem.* **2014**, *6*, 159–164.

[S17] Z. G. Szabó in *Comprehensive Chemical Kinetics* vol 2. (Eds.: C. H. Bamford, and C. H. F. Tipper), Elsevier: Amsterdam, **1969**, p. 43.

[S18] H. Eyring, M. Polanyi *Über Einfache Gasreaktionen. Z. Phys. Chem. B* **1931**, *12*, 279–311; H. Eyring, *J. Chem. Phys.* **1935**, *3*, 107–115.

[S19] Y. Yoshigoe, S. Suzaki, K. Osakada, *Chem. Lett.* **2014**, *43*, 1337–1339.

[S20] Ikemoto, K.; Fujita, M; Too, P. C.; Tnay, Y. L.; Sato, S; Chiba, S.; Isobe, H., Synthesis and Structures of π-Extended [*n*]Cyclo-para-phenylenes (*n* = 12, 16, 20) Containing *n*/2 Nitrogen Atoms. *Chem. Lett.* **2016**, *45* (6), 658-660.

[S21] T. Matsumoto, A. Yamano, T. Sato, J. D. Ferrara, F. J. White, M. Meyer, *J. Chem. Crystallogr.* **2020**, *51*, 438–450.

[S22] (a) G. M. Sheldrick, *Acta. Cryst.* **2015**, *C71*, 3; (b) G. M. Sheldrick, *Acta Cryst. A64*, **2008**, 112.

[S23] O. V. Dolomanov, L. J. Bourhis, R. J. Gildea, J. A. K. Howard, H. Puschmann, *J. Appl. Cryst.* **2009**, *42*, 339.

[S24] Gaussian 16, Revision B.01, M. J. Frisch, G. W. Trucks, H. B. Schlegel, G. E. Scuseria, M. A. Robb; J. R. Cheeseman; G. Scalmani, V. Barone, G. A. Petersson, H. Nakatsuji, X. Li, M. Caricato, A. V. Marenich, J. Bloino, B. G. Janesko, R. Gomperts, B. Mennucci, H. P. Hratchian, J. V. Ortiz, A. F. Izmaylov, J. L. Sonnenberg, D. Williams-Young, F. Ding, F. Lipparini, F. Egidi, J. Goings, B. Peng, A. Petrone, T. Henderson, D.

Ranasinghe, V. G. Zakrzewski, J. Gao, N. Rega, G. Zheng, W. Liang, M. Hada, M. Ehara, K. Toyota, R. Fukuda, J. Hasegawa, M. Ishida, T. Nakajima, Y. Honda, O. Kitao, H. Nakai, T. Vreven, K. Throssell, J. A. Montgomery Jr., J. E. Peralta, F. Ogliaro, M. J. Bearpark, J. J. Heyd, E. N. Brothers, K. N. Kudin, V. N. Staroverov, T. A. Keith, R. Kobayashi, J. Normand, K. Raghavachari, A. P. Rendell, J. C. Burant, S. S. Iyengar, J. Tomasi, M. Cossi, J. M. Millam, M. Klene, C. Adamo, R. Cammi, J. W. Ochterski, R. L. Martin, K. Morokuma, O. Farkas, J. B. Foresman, D. J. Fox, Gaussian, Inc., Wallingford CT, **2016**

[S25] M06: (a) P. J. Hay, W. R. Wadt, *J. Chem. Phys.* **1985**, *82*, 270-283; (b) W. R. Wadt, P. J. Hay, *J. Chem. Phys.* **1985**, *82*, 284-298; (c)P. J. Hay, W. R. Wadt, *J. Chem. Phys.* **1985**, *82*, 299-310.

[S26] 6-31g: (a) R. Ditchfield, W. J. Hehre, J. A. Pople, *J. Chem. Phys.* **1971**, *54*, 724-728; (b) W. J. Hehre, R. Ditchfield, J. A. Pople, *J. Chem. Phys.* **1972**, *56*, 2257-2260.

[S27] LANL2TZ(f): (a) M. M. Francl, W. J. Pietro, W. J. Hehre, J. S. Binkley, D. J. DeFrees, J. A. Pople, M. S. Gordon, *J. Chem. Phys.* **1982**, *77*, 3654-3665; (b) L. E. Roy, P. J. Hay, R. L. Martin, *J. Chem. Theory Comput.* **2008**, *4*, 1029-1031.
